# Mitochondrial heteroplasmy in vertebrates using ChIP-sequencing data

**DOI:** 10.1186/s13059-016-0996-y

**Published:** 2016-06-27

**Authors:** Thomas Rensch, Diego Villar, Julie Horvath, Duncan T. Odom, Paul Flicek

**Affiliations:** European Molecular Biology Laboratory, European Bioinformatics Institute, Wellcome Genome Campus, Hinxton, Cambridge CB10 1SD UK; Cancer Research UK Cambridge Institute, University of Cambridge, Robinson Way, Cambridge, CB2 0RE UK; Biological and Biomedical Sciences, North Carolina Central University, Durham, NC 27707 USA; North Carolina Museum of Natural Sciences, Raleigh, NC 27601 USA; Wellcome Trust Sanger Institute, Wellcome Genome Campus, Hinxton, Cambridge CB10 1SA UK

**Keywords:** Heteroplasmy, Chromatin immunoprecipitation sequencing (ChIP-seq), mitochondrial DNA (mtDNA), Mitochondrion, Vertebrates

## Abstract

**Background:**

Mitochondrial heteroplasmy, the presence of more than one mitochondrial DNA (mtDNA) variant in a cell or individual, is not as uncommon as previously thought. It is mostly due to the high mutation rate of the mtDNA and limited repair mechanisms present in the mitochondrion. Motivated by mitochondrial diseases, much focus has been placed into studying this phenomenon in human samples and in medical contexts. To place these results in an evolutionary context and to explore general principles of heteroplasmy, we describe an integrated cross-species evaluation of heteroplasmy in mammals that exploits previously reported NGS data. Focusing on ChIP-seq experiments, we developed a novel approach to detect heteroplasmy from the concomitant mitochondrial DNA fraction sequenced in these experiments.

**Results:**

We first demonstrate that the sequencing coverage of mtDNA in ChIP-seq experiments is sufficient for heteroplasmy detection. We then describe a novel detection method for accurate detection of heteroplasmies, which also accounts for the error rate of NGS technology. Applying this method to 79 individuals from 16 species resulted in 107 heteroplasmic positions present in a total of 45 individuals. Further analysis revealed that the majority of detected heteroplasmies occur in intergenic regions.

**Conclusion:**

In addition to documenting the prevalence of mtDNA in ChIP-seq data, the results of our mitochondrial heteroplasmy detection method suggest that mitochondrial heteroplasmies identified across vertebrates share similar characteristics as found for human heteroplasmies. Although largely consistent with previous studies in individual vertebrates, our integrated cross-species analysis provides valuable insights into the evolutionary dynamics of mitochondrial heteroplasmy.

**Electronic supplementary material:**

The online version of this article (doi:10.1186/s13059-016-0996-y) contains supplementary material, which is available to authorized users.

## Background

Mitochondrial DNA (mtDNA) forms a circular molecule, which is located in the mitochondrial matrix [1]. In mammals, mtDNA is ~16.5 kb long and contains 37 genes [2]. For the most part, mtDNA either codes for proteins or for ribosomal RNAs and transfer RNAs, except for a 1 kb stretch known as the control region, which contains one origin of replication and both origins of transcription [3, 4]. Several identical mtDNA copies (between 2 and 10 in humans) are present in each individual mitochondrion, which means a single cell can contain hundreds to thousands of copies of mtDNA [2, 4]. The mtDNA was the first part of the human genome to be sequenced and to this day is one of the most studied segments of DNA in humans and in many other species [2, 4]. In addition to its high copy number, the mutation rate of mtDNA is significantly higher than that of nuclear DNA [3]. These properties make it common for an individual to have more than one mtDNA variant: this phenomenon is known as heteroplasmy [5] and has been observed and studied in many species and contexts.

In humans, hundreds of diseases are linked to point mutations in the mitochondrial genome [6], suggesting that a fraction of human mitochondrial mutations may be pathogenic. Many of these mutations exist in a heteroplasmic state and the extent of the disease symptoms vary according to the proportion of the deleterious allele [7]. Such diseases include many metabolic diseases, age-related neurodegenerative diseases such as Alzheimer’s and Parkinson’s, as well as several types of cancer [6, 8–11]. Research in fields such as population genetics and forensics has also focused on heteroplasmy as a way to investigate aspects of inheritance [12]. Although heteroplasmic positions have also been observed in other mammalian species [13–16], studies of the phenomenon in other taxa have mostly been conducted for genetic barcoding or to investigate molecular evolution and generally focused on small controlled datasets [17–21]. Cross-species comparisons have been reported in a limited number of closely related species, such as different types of bees [22–24].

Heteroplasmy was first reported in 1983 [25] and has been detected with a variety of methods including Sanger capillary sequencing [26] and pyrosequencing [27]. However, these sequencing methods are expensive and slow, which limited the number of studied samples. More recently, next-generation sequencing (NGS) has been used to study mitochondrial heteroplasmy with high-throughput data and several computational approaches for heteroplasmy detection have been developed [28–33]. The main challenge in using NGS data to detect heteroplasmies is sequencing errors, which tend to be location-specific and thus can be confused with heteroplasmies. To avoid such biases, criteria for NGS-based heteroplasmy detection were developed using PhiX genome simulations and establishing different quality thresholds to identify heteroplasmic positions [28]. Since the heteroplasmy detection power increases with coverage, recent studies employing high coverage sequencing (>1000×) have adapted these criteria [29] (e.g. more lenient thresholds) as well as developed advanced probabilistic models to detect micro-heteroplasmies (i.e. positions with a minor allele ratio below 2–5 %) [30, 34]. In this study, we focus on detecting a higher level of heteroplasmy (>15 %) using a modified version of the established criteria [28].

Previous heteroplasmy studies used targeted mtDNA sequencing. In most cases mtDNA was extracted from whole blood or buccal tissue, although recently a few studies have investigated a range of tissues [35, 36]. More recently, mitochondrial heteroplasmy has been assayed using data from many whole-genome sequencing studies including from the 1000 Genomes Project [37] and in other NGS datasets, such as exome- and RNA-sequencing (RNA-seq) [38, 39]. It is currently unknown whether genomic enrichment assays such as Chromatin immunoprecipitation followed by high-throughput sequencing (ChIP-seq) have suitable characteristics for mitochondrial heteroplasmy detection including relatively uniform coverage and appropriate sequencing depth, although some assays such as ATAC-sequencing (ATAC-seq) are known to include a high fraction of mtDNA reads.

As the cost of sequencing continues to drop, the quantity of datasets being generated and stored is rapidly increasing. Among the benefits of public availability of sequencing experiments is their use to efficiently answer research questions not explored at the time of data generation. Here, we exploit a combination of previously generated and novel datasets resulting from ChIP-seq experiments to perform heteroplasmy detection across a range of vertebrate species. Although mtDNA is of the order of 0.1 % of all DNA in a cell [40], the high copy number of the circular mitochondrial genome generally leads to it being sequenced many times in ChIP-seq experiments, resulting in a significant proportion of ChIP-seq reads covering the mtDNA [41–43].

We first confirm the prevalence of mtDNA in published ChIP-seq data and show that mtDNA coverage is suitable for heteroplasmy detection. We then apply a novel heteroplasmy detection method to a collection of both novel (see “Methods”) and previously published ChIP-seq datasets comprising a total of 79 individuals from 16 species. Our findings provide several insights into the dynamics of mtDNA heteroplasmy over a large portion of the mammalian phylogeny.

## Results

### Large mammalian dataset

We gathered ChIP-seq data from five previously published studies [44–48] and performed new transcription factor (TF) and histone modification ChIP-seq experiments (see “Methods”) on a selection of samples that were used in the aforementioned papers. We selected published ChIP-seq datasets (signal and input files) from large cross-species comparison studies to mitigate batch effects. The combined data cover a wide range of species spanning the mammalian clade including primates, rodents, and domesticated animals such as dogs, cats, and cattle, as well as chicken as an out-group vertebrate species. Most of these samples come from liver tissue, but some consist of lymphoblastoid cell lines. After analysis (see below and “Methods”), we identified a core set of 16 species for comparison – *Homo sapiens* (human), *Macaca mulatta* (macaque), *Chlorocebus aethiops sabaeus* (vervet), *Callithrix jacchus* (marmoset), *Otolemur garnettii* (bushbaby), *Mus musculus domesticus* (mouse), *Rattus norvegicus* (rat), *Heterocephalus glaber* (naked mole-rat), *Oryctolagus cuniculus* (rabbit), *Bos taurus* (cattle), *Delphinus delphis* (dolphin), *Sus scrofa* (pig), *Canis familiaris* (dog), *Mustela putorius furo* (ferret), *Sarcophilus harrisii* (Tasmanian devil), and *Gallus gallus* (chicken).

### ChIP-sequencing data for heteroplasmy detection

A ChIP-seq study generally consists of two experiments that each result in short read sequencing data files [49]. The first is commonly known as the signal file and contains reads resulting from the ChIP experiment, which when mapped to the target genome produces read clusters (peaks) identifying genomic locations where the proteins targeted by the ChIP antibody were bound. The second data file is a control experiment consisting of a similar process, but without the immunoprecipitation step, and is used to control for biased genome-wide read coverage arising from preferential sonication of open chromatin [50]. Thus, the control data generally contain reads that map to the entire genome, with few expected enriched regions. Due to the high copy number of the mtDNA, reads within the mitochondrial genome are sequenced many times in both signal and control ChIP-seq experiments. We observed significant read coverage in both data files, even though binding peaks within mtDNA were not detected (see “Methods”).

**Fig. 1:**
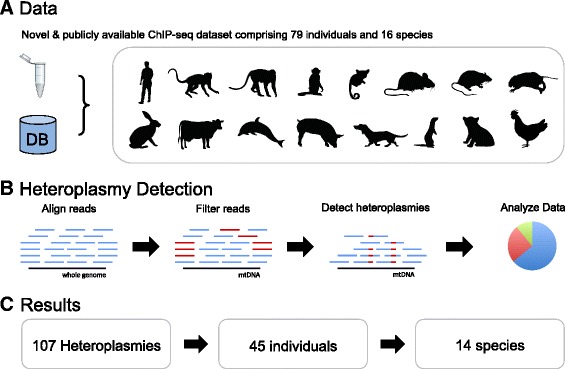
Heteroplasmy detection *workflow*. The raw read files obtained from the ChIP-seq experiments were first aligned to the respective reference genomes. The aligned reads were then pre-processed, filtering out duplicate reads and extracting reads mapping with a high quality score to the mtDNA. The heteroplasmy detection algorithm was then used across the samples. Finally, we analyzed the genomic properties of heteroplasmic positions across vertebrates

**Fig. 2:**
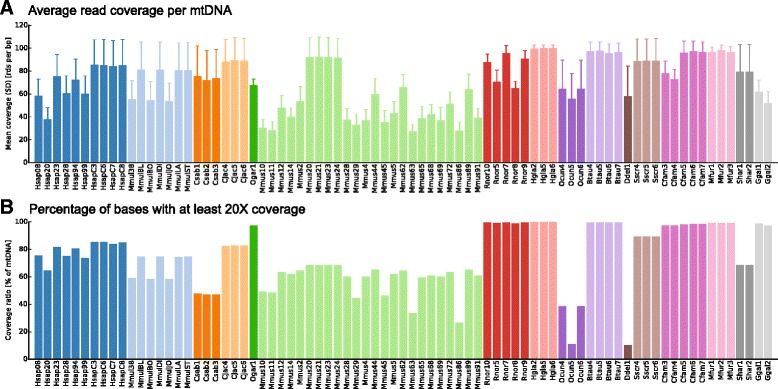
mtDNA read coverage per individual. **a** The mean read coverage per mtDNA base pair for each analyzed individual, colored per species (the *error bars* represent the standard deviation). **b** The fraction of mtDNA base pairs covered by at least 20 reads (our heteroplasmy detection cutoff) also colored by species

We used ChIP-seq data originally generated to map various histone modifications and TFs such as CEBPA and FOXA1, as well as the input/control data for each experiment (Fig. 1). We merged all experiments corresponding to the same individual, which provides better total coverage and acted as technical replicates (i.e. sequencing of the same biological tissue) for the mtDNA detection experiment. When combined in this way, coverage for each individual was relatively high and homogenous for almost all species, with average mitochondrial coverage above 50× and coverage ratio above 70 % (Fig. 2). As previously reported [28], such coverage levels are adequate to detect high-level heteroplasmies with high specificity. Detailed coverage data for each individual are available in the supplementary materials (Additional file 1: Figure S1). For six species in the original published studies forming our collected dataset, but not included in the 16 core species (*Monodelphis domestica*, *Cavia porcellus*, *Tupia belangeri*, *Balaenoptera borealis*, *Mesoplodon bidens*, and *Lagenorhynchus albirostris*), we observed very low rates of uniquely mapping reads on mtDNA. *C. porcellus* (guinea pig) and *T. belangeri* (tree shrew) have highly fragmented genome assemblies that may hinder accurate mtDNA read mapping, and *M. domestica* (opossum) is known to have a significantly increased number of NUMTS (nuclear mitochondrial DNA sequences), which may also have affected the number of uniquely mtDNA mapping reads [51]. Samples from *Balaenoptera borealis* (sei whale), *Mesoplodon bidens* (Sowerby’s beaked whale), and *Lagenorhynchus albirostris* (white-beaked dolphin) were all mapped to the closely related *Tursiops truncatus* (common bottlenose dolphin) species’ genome, a process that also yielded few uniquely mapping reads to the mtDNA except for *Delphinus delphis* (short-beaked common dolphin), see Fig. 2. Since coverage in these species was insufficient for heteroplasmy detection, we excluded them from further analysis. Finally, we also discarded a *Mus musculus* (mouse) individual for which the coverage ratio fell below 10 %.

### Heteroplasmy detection algorithm

We adapted a previously published heteroplasmy detection methodology [28] for the specific characteristics of ChIP-seq data (see below and “Methods”). Briefly, this method is based on a set of criteria to be checked for each mtDNA base pair. In addition to quality thresholds, the algorithm requires a minimum number of reads to be present on each strand. Verification of heteroplasmies on both strands avoids location-specific errors that may arise from sequencing errors, since it is uncommon for these to occur at the same location on each strand [28]. Distinguishing characteristics of our method include aligning with BWA [52] (instead of assembling the reads) and a parameter set optimized for ChIP-seq data. This parameter set is generally more stringent than in previous reports [28, 29] and includes a higher base quality threshold (base quality >23) and an added minimum coverage threshold (20 reads). We also increased the minimum heteroplasmy level to 15 % (minor allele frequency). Although these changes to the algorithm result in lower expected sensitivity, we do so to optimize specificity from the generally less homogenous sequencing coverage in ChIP-seq samples, compared to that observed in targeted mtDNA resequencing.

Nucleotide repeats present in low complexity regions strongly hinder sequencing quality over those locations. While previous studies have excluded these positions from their analysis, these regions are also more likely to harbor heteroplasmies due to error-prone polymerase activity and limited DNA repair in the mitochondrion. The human mitochondrial annotation database MITOMAP [53] lists several positions as heteroplasmic within these regions, of which we find nine (see “Methods”) with our detection method. Since the detection parameters are very stringent, we decided to keep the repetitive regions in our analysis.

In addition to liver samples, we also applied our detection algorithm to ChIP-seq data from several primate species’ lymphoblastoid cell lines (LCLs) [46]. We observed that 33 % of them expressed more than 25 heteroplasmic positions, which we assume might be due to genomic instability in the immortalized cell lines that could have arisen from a high passage number of the cells [54]. Our results from two *Felis catus* (cat) samples also exhibited a surprisingly high number of heteroplasmies in both individuals (Additional file 2: Table S1). Furthermore, almost all of the positions detected in one cat individual were present in the other, which may be due to low genetic diversity in the source population or the two individuals being siblings. Other possibilities are that some contamination occurred in the process or that cat may have a specific pattern of NUMTS that impedes analysis. For these reasons, we do not include the primate LCLs or the cat data in the core set of species or the remaining comparative analysis in the paper.

**Fig. 3:**
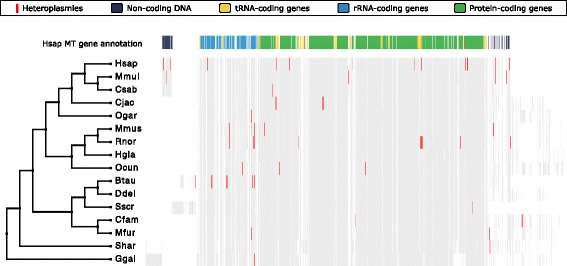
Heteroplasmies in 16 species. Detected heteroplasmy in *H. sapiens* (human), *M. mulatta* (macaque), *C. sabaeus* (vervet), *C. jacchus* (marmoset), *O. garnettii* (bushbaby), *M. musculus* (mouse), *R. norvegicus* (rat), *H. glaber* (naked mole-rat), *O. cuniculus* (rabbit), *B. taurus* (cattle), *D. delphis* (dolphin), *S. scrofa* (pig), *C. familiaris* (dog), *M. putorius furo* (ferret), *S. harrisii* (Tasmanian devil), and *G. gallus* (chicken) displayed in *red* on the mtDNA multiple alignment with the associated evolutionary tree. The human gene annotation displayed at the *top* of the figure shows RNA and protein-coding genes as well as non-coding regions

After the filtering above, our final dataset included comparative heteroplasmy results from liver samples of 79 individuals across 16 species. For these, we found 107 positions in 45 individuals across 14 species (Fig. 3; Additional file 2: Table S2). A total of 57 % of the individuals express heteroplasmy. Our estimate is higher than initial NGS-based reports of human heteroplasmy [28], but consistent with recent reports on high-coverage datasets [34, 35], which also showed that liver tissue has one of the highest relative number of heteroplasmies compared to other human tissues [35]. In fact, we find heteroplasmic positions in every species except *Heterocephalus glaber* (naked mole-rat) and *Delphinus delphis* (short-beaked common dolphin).

### Heteroplasmic positions present in multiple individuals

**Fig. 4:**
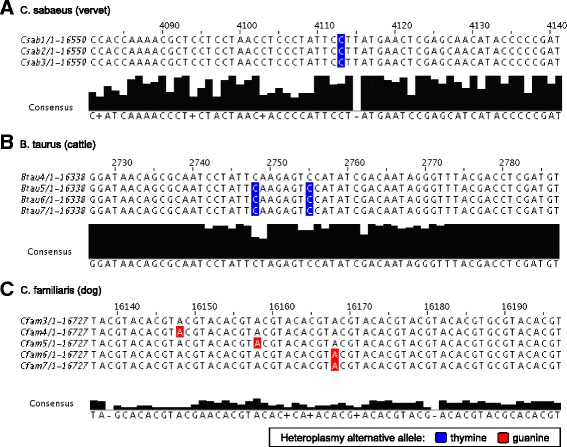
Sequence context of multiple heteroplasmies present in three species (*Chlorocebus sabaeus*, *Bos Taurus*, and *Canis familiaris*). Heteroplasmic positions are colored according to the alternative allele nucleotide (*blue*: thymine, *red*: guanine). The consensus sequence of the multiple alignment is displayed below each species sequences

Several positions occur in more than one individual of the same species (Fig. 4; Additional file 2: Table S2), a phenomenon that has previously been observed in humans and attributed in part to differential mutation rates across the mtDNA sequence [28]. Since heteroplasmies in humans are mostly located at positions with high relative mutation rate [28, 34] and mutation rate patterns are shared across individuals, such positions are more likely to exist in a heteroplasmic state in more than one individual. Similar differential mutation rate patterns are likely in other species and thus the shared positions we observe may also have high mutation rates. Likewise, we asked whether this phenomenon could be linked to sequence conservation, but the heteroplasmic positions occurring in more than one individual do not show evidence of conservation bias (Fig. 4). That some individuals may be closely related via their breeding history is another possible explanation.

### Read coverage of the heteroplasmic positions

The average number of reads supporting each of our observed heteroplasmic position is 60 (SD 25), which is significantly higher than our 20 read threshold (Additional file 1: Figure S2). Indeed, there are no observed positions with read coverage of exactly 20 reads and only three positions with coverage of 21 reads. Based on the observed coverage distribution, our detection parameters including the coverage threshold appear to be conservative.

**Fig. 5:**
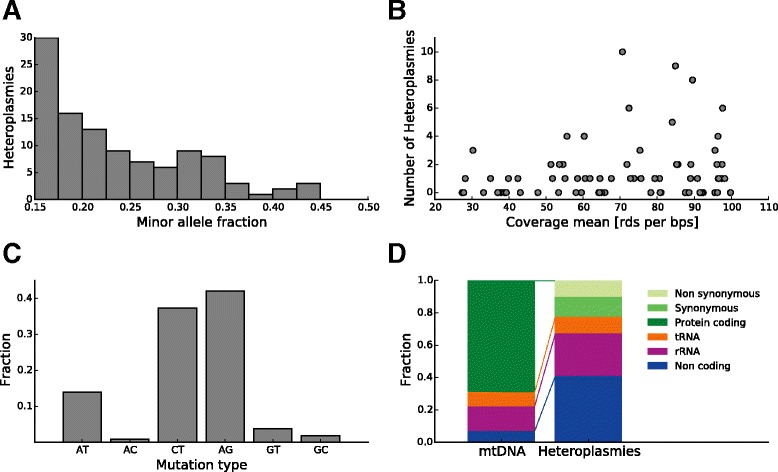
Characteristics of heteroplasmies. **a** The minor allele fraction of the detected heteroplasmies (heteroplasmy level). Most positions are detected close to the detection threshold of 15 %. **b** The number of heteroplasmies plotted against the mean coverage for each analyzed individual. There is a minor correlation in the data (Pearson’s *r* = 0.17), however there is essentially no correlation for individuals with a mean coverage of more than 40 (Pearson’s *r* = 0.05). **c** The mutational spectrum of the heteroplasmies detected is similar to the spectrum of mutations reported in MITOMAP (*X* ^2^ *p* = 0.07). **d** The genomic location of heteroplasmies is strongly biased and significantly different to the repartition of mtDNA elements

For each individual, we compared the average coverage across the mtDNA to the number of observed heteroplasmic positions. We found a minor correlation across the entire distribution (Pearson’s *r* = 0.17) (Fig. 5a), but for individuals with high average coverage (>40 reads per mtDNA position), there is essentially no correlation between coverage and number of observed heteroplasmies (Pearson’s *r* = 0.05). This result further suggests that our chosen coverage threshold is sufficient for robust detection of high-level heteroplasmies. As expected, the heteroplasmy level distribution is highest at 15 % minor allele frequency corresponding to the threshold level (Fig. 5c). Previous studies also report that the majority of heteroplasmic positions occur at the lowest minor allele level [28, 34].

### Heteroplasmy mutation spectrum analysis

The transition-transversion rate is strongly biased in the mitochondrion [55]. We observe a transition-transversion ratio of Ts/Tv = 3.86 in our results across all species, which is similar to the ratio found in the MITOMAP database (Ts/Tv = 2.95, Fisher’s exact test, *p* = 0.31). In fact, the full mutational spectrum we observe in our multi-species dataset (Fig. 5b) is similar to that observed in the MITOMAP database (*X*^*2*^ test, *p* = 0.07). Additionally, the fact that the most common Illumina sequencing errors (AC and GT transversions [56]) are rare in our observed multi-species mutation spectrum strongly suggests that we have few false positives due to sequencing errors in our set of heteroplasmic positions.

### Genomic location of the detected heteroplasmies

As shown in Figs. 3 and 5d, out of the 107 heteroplasmic positions found, 44 are in intergenic regions with most of these in the hyper-variable D-loop and only five in other mitochondrial intergenic regions. Of the remaining positions, 39 are located in non-protein-coding genes (28 in ribosomal RNA (rRNA) genes and 11 in tRNA genes) and 24 positions are located in protein-coding genes. Of the protein-coding gene changes, 13 are synonymous variations meaning they do not affect the amino acid used in the translated protein, while there are 11 non-synonymous variants in six species. However, most of the observed amino acid changes (7 out of 11) are between residues with similar biochemical properties. In four cases, the observed changes from isoleucine to threonine and from valine to alanine are modifications between hydrophobic and hydrophilic amino acids.

The relative paucity we observed for heteroplasmies in non-coding regions of the mitochondrial genome may be partly due to negative selection acting on heteroplasmy positions within mitochondrial genes [33].

### Heteroplasmic positions associated with disease

For humans, about 5 % of mtDNA positions are associated with disease [53]. Using the MITOMAP annotations, five positions (15 %) among the human heteroplasmies we find are disease-associated. This is more than the proportion of positions associated with disease in MITOMAP, but comparable to the previously observed proportion in humans from a set of five Eurasian populations (11.8 %, Fisher’s exact test, *p* = 0.99) [28]. We then considered whether a similar proportion of positions in other species could be considered deleterious. Since there are no MITOMAP-type databases for the other species listing disease associations, we assigned heteroplasmic positions in other species to their orthologous human positions (see “Methods”). For the 43 positions in other species that could be confidently assigned an orthologous position on the human mitochondria, two (4.7 %) were associated with disease (13,882 in Rnor5 – *Rattus norvegicus* and 1068 in Btau4 – *Bos taurus*) based on human MITOMAP annotations. These correspond to a synonymous protein-coding gene mutation in *Rattus norvegicus* and an rRNA mutation in *Bos Taurus*. Although we do not assume that disease associations observed in humans will be maintained at orthologous positions in other species, the observed rate is indistinguishable from the baseline disease associated rate in the MITOMAP database (Fisher’s exact test, *p* = 0.99). Therefore, these results suggest that the distribution of functionally relevant mitochondrial mutations is similar across the species we studied. This observation may reflect a comparable mitochondrial disease burden across mammals.

### Sanger sequencing and pyrosequencing validation of heteroplasmies

To validate the mitochondrial heteroplasmies identified in ChIP-seq datasets with an independent method, we selected 34 heteroplasmic positions randomly from the total 107 we detected (see Additional file 2: Table S3). This validation set includes positions with a range of minor allele frequencies and comprises 12 species. For these, we performed Sanger sequencing on mtDNA amplicons to confirm the presence of two sequence variants at each heteroplasmic position (see “Methods”). We validated heteroplasmies with high confidence in 14 positions. A further seven positions showed some evidence for heteroplasmy, while we did not detect any heteroplasmy in the remaining 14 positions (41 %). Because of the relatively low sensitivity of Sanger sequencing for detection of single-nucleotide variants [57], we performed further validations using pyrosequencing as an alternative method. We selected five of the previously tested positions we could not validate by Sanger sequencing and an additional 13 positions (Additional file 2: Tables S3 and S5). Pyrosequencing data supported heteroplasmies predicted from ChIP-seq data for all of the positions previously tested by Sanger sequencing and 11 out of the 13 (85 %) newly tested positions. For three of the newly validated positions, the identities of minor and major allele were reversed in pyrosequencing compared to the ChIP-seq based detection, which may be due to indels or other discrepancies between ChIP-seq libraries and mtDNA samples used for pyrosequencing. In sum, these results indicate that our stringent criteria for heteroplasmy detection in ChIP-seq experiments largely identify true mitochondrial heteroplasmies that can be independently validated by Sanger sequencing or pyrosequencing of mtDNA samples.

Along with the validation of heteroplasmic sites identified in ChIP-seq data, we also performed pyrosequencing of the same positions in different individuals of the corresponding species for which our NGS method did not detect heteroplasmies. Supporting the stringency of our computational approach, in a number of cases pyrosequencing data also detected low levels of heteroplasmy in individuals where ChIP-seq based identification was negative (see Additional file 2: Tables S3 and S5).

## Discussion

### Detected heteroplasmic positions

We find an average number of 2.4 heteroplasmic positions per individual with heteroplasmy. Although this is higher than previously found in humans [28, 29], two recent studies show that liver tissue has an increased amount of heteroplasmies compared to other tissues, and most previous studies were performed with whole blood and buccal tissue samples [35, 36]. Our detection method also extends prior approaches by not discarding heteroplasmic positions within low complexity regions (see “Results”), while these were not considered in previous studies. Moreover, we find most heteroplasmic positions with minor allele ratios close to our 15 % threshold (Fig. 5c). This is consistent with previous studies, which also found most heteroplasmies at their respective thresholds [28, 34], and suggests that additional heteroplasmies exist beyond the threshold that we used. Finally, since we used relatively stringent detection criteria, it is likely that the heteroplasmic positions we identified are in fact a subset of all heteroplasmies present in the samples. We hypothesize that the apparent absence of heteroplasmies in *Heterocephalus glaber* (naked mole-rat) and *Delphinus delphis* (short-beaked common dolphin) is due to profiling of only a few individuals in these species (one individual of *D. delphis* and three of *H. glaber*).

The genomic location of heteroplasmies across vertebrates is also consistent with previous findings [28, 34, 36] with the majority of positions occurring in the non-coding control region which is the most polymorphic segment of the mtDNA. In fact, most of the heteroplasmies are located in non-coding regions or (structural) RNA genes in which modifications of single nucleotide bases are expected to only have minor effects [58]. Furthermore, heteroplasmies located in protein-coding genes, genome elements that are highly conserved [59], are almost exclusively either synonymous mutations or result in biochemically similar amino acids.

### Validation of heteroplasmies identified in ChIP-seq datasets

Through a combination of Sanger sequencing and pyrosequencing, we carried out validation experiments for a total of 47 heteroplasmies predicted from ChIP-seq data. While Sanger sequencing alone only validated 20 out of 34 interrogated positions (59 %), a fraction of the undetected positions could still be true heteroplasmies given the low sensitivity of this method. Further validation using pyrosequencing as a more sensitive method further validated heteroplasmies that were negative in Sanger sequencing (5 out of 5 tested), as well as a large fraction of newly tested heteroplasmy positions detected at low minor allele frequencies in ChIP-seq data (11 out of 13, 85 %). These results strongly suggest that most of the heteroplasmies we identified in ChIP-seq data are true heteroplasmies that can be validated in mtDNA samples. However, given the stringency of our detection method (with a minor allele threshold of 15 %), it remains likely that we are underestimating the full extent of heteroplasmy positions in each sample. In this regard, mtDNA samples from individuals for which a particular heteroplasmy could not be detected based on our ChIP-seq method often showed low levels of heteroplasmy in pyrosequencing.

### Number of different mtDNA genomes

When an individual expresses one heteroplasmic position, it is likely that only two variants of the mtDNA genome exist within its cells. However, when individuals express more than one heteroplasmic position, which is the case for 21 individuals in this study (see Additional file 2: Table S2), we cannot determine the underlying number of mtDNA genome variants. Estimating this number could be possible using the heteroplasmy minor allele fractions, however this would require a precise evaluation of the heteroplasmy level. This might be feasible with high coverage data but is not realistic with the data presented here. Further, none of the detected heteroplasmic positions has more than two alleles. Although our analysis revealed a handful of positions that have one sequencing read containing an additional third base, such potential third alleles never fulfill the criteria to be determined as alternative minor alleles. In sum, our results cannot discriminate the underlying number of mtDNA genomes. Emerging technologies that promise to provide significantly longer reads would help in identifying combinations of mutations on the same mtDNA molecule.

### MtDNA coverage in ChIP-seq data

It is well-known that the mtDNA is sequenced many times due to its high copy number and that significant amounts of mtDNA reads are found in most NGS generated datasets including in ChIP-sequencing data for which mtDNA coverage has even been used as a control for background noise [41]. However, since most ChIP-seq studies focus on binding events in the nuclear DNA and use other control methods, reads mapping to mtDNA have been discarded as mitochondrial contamination. Here we demonstrate that mtDNA coverage in ChIP-seq samples is in fact so high that it may be used similarly to targeted mtDNA shotgun sequencing. We first studied the coverage of each ChIP-seq file independently and observed that it was consistently deeper in the control files compared to the TF binding files, which also had higher mtDNA coverage than the histone ChIP-seq files (Additional file 1: Figure S3). Low mtDNA coverage in ChIP-seq for histone marks is expected, since histones are not used to pack mtDNA in the mitochondrion [5]. Although the investigated TFs do not show signs of binding on the mtDNA (see “Methods”), it has been reported that several TFs that are active in the nucleus can also bind the mtDNA [43] and such weak binding events could explain the high coverage found in our experiments. However, due to the even read coverage observed, it seems most likely, at least for the factors investigated here, that the presence of mtDNA in ChIP-sequencing data is a technical and not biological phenomenon. Overall, after merging the different files the coverage level and ratio were relatively high and comparable to the data from Li et al. [28], on which we based our detection algorithm. On the other hand, coverage ratio was variable between species, ranging from the entire mtDNA to as little as 10 %. This caveat restricts the conclusions we can draw about the genomic location of heteroplasmies within individual species, since some portions of the mtDNA genome are not covered in every individual. However, although we were limited in our ability to conduct some analyses, such as detailed cross-species comparisons of heteroplasmic positions, we focused on extracting information from our dataset as a whole to obtain conclusive results.

### ChIP-seq datasets are useful data sources for the study of heteroplasmy

We have shown that it is possible to make use of ChIP-sequencing data that were originally collected for other purposes to explore mitochondrial heteroplasmies across a wide variety of mammalian and vertebrate species, by taking advantage of the high number of sequenced mtDNA reads in these experiments. We hope our general approach will encourage the further study of mtDNA and other biological questions using valuable existing datasets. For instance, making use of data arising from other profiling-sequencing methods such as RNA-seq or ATAC-seq might be very useful, as demonstrated by a recent study of the mitochondrial transcriptome [38]. However, making use of RNA-seq data to explore mitochondrial heteroplasmy would require caution, as some RNA-DNA differences (RDDs) have been reported in the mitochondrial genome [60]. Finally, although we adapted a detection method appropriate for our datasets of relatively low-coverage and variable homogeneity, higher coverage and/or more advanced methods for heteroplasmy detection [30, 34] could potentially be valuable for a wider variety of existing data types. For example, higher coverage datasets will likely enable new methods to explore lower levels of heteroplasmy in ChIP-seq data. Furthermore, having demonstrated that ChIP-seq coverage of the mtDNA can be substantial and allows for the study of heteroplasmy, it is likely that ChIP-seq data can be used to perform other mtDNA studies in fields such as population genetics, forensics, and in some areas of medical research. There is a current trend of creating large genotype-phenotype datasets for ChIP-seq and RNA-seq data and using these datasets to explore phenotype associations to heteroplasmies could prove extremely valuable to further our knowledge of the phenomenon.

## Conclusions

### Heteroplasmies show consistent characteristics

Our study shows that mitochondrial heteroplasmy displays similar characteristics across vertebrate species, including genomic location and mutation spectrum across the vertebrate species tree. As might be expected, our results strongly suggest that previous heteroplasmy findings established in humans are valid for all mammals and possibly all vertebrates. In addition to this, our results also support recent findings that heteroplasmy is more prevalent in liver compared to other tissues [35, 36, 61]. Our results also suggest that any new understanding about heteroplasmies will likely apply across the mammalian clade. It is clear, both in our findings and in previous work, that mutation rates vary significantly between positions, meaning some positions are more likely to exist as heteroplasmies than others. Although there is limited information, on a molecular level, describing the functional impacts these heteroplasmies may have, our results suggest that those functional impacts are likely to be similar across many different mammalian species and that many species may be effective model organisms for understanding the biology of heteroplasmy.

## Methods

### ChIP-seq data

We used a combination of newly generated and previously published ChIP-seq data as described here.

We performed ChIP-seq experiments for CEBPA, H3K4me1, H3K4me3, H3K27ac, and total histone H3 on a collection of liver samples from multiple species: CEBPA in nine species (*C. jacchus*, *C. familiaris*, *C. porcellus*, *F. catus*, *H. glaber*, *M. domestica*, *M. furo*, *O. cuniculus*, and *T. belangeri)*, H3K4me1 in 20 species (*B. borealis*, *B. taurus*, *C. jacchus*, *C. familiaris*, *C. porcellus*, *C. sabaeus*, *D. delphis*, *F. catus*, *H. glaber*, *L. albirostris*, *M. mulatta*, *M. bidens*, *M. domestica*, *M. furo*, *O. cuniculus*, *O. garnettii*, *R. norvegicus*, *S. harrisii*, *S. scrofa*, and *T. belangeri*), H3K4me3 in three species (*C. jacchus*, *O. garnettii*, and *O. garnettii*), H3K27ac in six species (*H. glaber*, *M. domestica*, *O. garnettii*, *S. harrisii*, *S. scrofa*, and *T. belangeri*), and total histone H3 in eight species (*D. delphis*, *H. glaber*, *M. mulatta*, *M. domestica*, *O. garnettii*, *S. harrisii*, *S. scrofa*, and *T. belangeri*). Additional details are listed in Additional file 2: Table S4.

Wherever possible, livers from young adult males were used. Tissues from eight species were excess from routine euthanasia procedures including from individuals sacrificed during maintenance of research colonies. Five species were purchased commercially from slaughterhouses, for example. Specialty conservation programs including zoos, the Duke Lemur Center, or Cetacean Strandings surveillance often collect tissues for research purposes, and we obtained seven species’ tissues from these efforts. With the exception of the *Lagenorhynchus albirostris* sample, cetacean tissues were from live stranded individuals that died on the beach and were in a freshly dead condition at the time of post-mortem.

Our ArrayExpress [62] submission, E-MTAB-3933, has complete sample meta-data, raw sequencing files, and detailed experimental protocols. Briefly, chromatin immunoprecipitation was carried out from 0.1–0.5 g of liver tissue, using antibodies against H3K4me3 (millipore 05-1339), H3K27ac (abcam ab4729), H3K4me1 (abcam ab8895), total histone H3 (abcam ab1791), or CEBPA (Santa Cruz Biotechnology sc-9314). Histone mark ChIP experiments were performed with automated 96-well protocols in an Agilent Bravo liquid handling robot [63]. A manual version of the protocol was used for CEBPA experiments to allow for higher chromatin input.

The previously published data obtained from [44–48] was obtained from the ArrayExpress database (see “Supporting Data” for accession numbers). We performed the preprocessing and aligning of the reads (see below) so only raw read files (in FASTQ format) were downloaded. Details of the datasets are as follows: HNF4A and CEBPA ChIP-seq data for the liver tissue of five species (*H. sapiens*, *M. musculus*, *C. familiaris*, *M. domestica*, and *G. gallus*) [44]; CTCF, SA1, NRSF/REST, and H2AK5ac ChIP-seq data for the liver tissue of six species (*H. sapiens*, *M. mulatta*, *M. musculus*, *R. norvegicus*, *C. familiaris*, and *M. domestica*) [45]; CTCF and YY1 ChIP-seq data for LCLs of seven species (*H. sapiens*, *P. troglodytes*, *G. gorilla*, *P. pygmaeus*, *M. mulatta*, *P. hamadryas*, and *S. oedipus*), and YY1 liver tissue ChIP-seq data for two species (*H. sapiens* and *M. musculus*) [46]; CEBPA, FOXA1, ONECUT1, and HNF4A ChIP-seq data for the liver tissue of five species (*H. sapiens*, *M. mulatta*, *M. musculus*, *R. norvegicus*, and *C. familiaris*) [47]; H3K4me3 and H3K27ac ChIP-seq data for the liver tissue of 21 species (*H. sapiens*, *M. mulatta*, *C. sabeus*, *C. jacchus*, *M. musculus*, *R. norvegicus*, *C. porcellus*, *H. glaber*, *O. cuniculus*, *T. belangeri*, *B. taurus*, *D. delphis*, *L. albirostris*, *B. borealis*, *M. bidens*, *S. scrofa*, *C. familiaris*, *F. catus*, *M. furo*, *M. domesticus*, and *S. harrisii*) [48]; and CEBPA, H3K4me1, H3K27ac, and total Histone H3 ChIP-seq data for the liver tissue of 21 species (*M. mulatta*, *C. sabeus*, *C. jacchus*, *M. musculus*, *R. norvegicus*, *C. porcellus*, *H. glaber*, *O. cuniculus*, *T. belangeri*, *B. taurus*, *D. delphis*, *L. albirostris*, *B. borealis*, *M. bidens*, *S. scrofa*, *C. familiaris*, *F. catus*, *M. furo*, *M. domesticus*, *S. harrisii*, and *O. garnettii*) (see “Methods”). Experimental details regarding all of these experiments are described in detail in their respective publications, as well as in the protocols in ArrayExpress.

### Pre-processing and read alignment

Raw sequencing reads (FASTQ files) were aligned to the whole genome of their respective species obtained from Ensembl (v. 81) [64]. The human samples were aligned to the human reference genome used in the 1000 Genomes Project (GRCh37) [65]. Some species were aligned to closely related species’ genomes. Specific assemblies and files used for alignment are listed in Additional file 2: Table S4. Finally, the samples of species, for which a full genome was not available (*O. garnettii*, *H. glaber*, and *M. furo*), were aligned to the reference mitochondrial mtDNA sequence obtained from the NCBI Nucleotide database [66] (Additional file 2: Table S4). All of the raw read files were aligned using BWA (Burrow-Wheeler Aligner) [52] with default parameters. The aligned read files in BAM format were then merged per individual using SAMtools merge [67]. Next, we removed duplicate reads with SAMtools rmdup (keeping only the read with the highest mapping quality score per set of coordinates). Finally, reads aligning to the mtDNA with a mapping quality score of at least 20 were extracted using SAMtools view (parameter q = 20).

### Heteroplasmy detection and data analysis

The heteroplasmy detection algorithm as well as the analysis performed in this study were implemented in Python 2.7 [68] using the following scientific packages, SciPy [69], Pandas [70], Matplotlib [71], and Pysam (a SAMtools wrapper) and all the code is available in the supplemental data (Additional file 3). Our algorithm processes BAM files, scanning through the entire mitochondrial genome. For each base, it retrieves the set of reads covering that base. This read set is first filtered according to two criteria: (1) reads that have a Phred quality score lower than 23 at the position are discarded; and (2) reads for which any of the 5 neighboring base pairs (both directions) has a lower quality than 15 are discarded. Three further criteria are used to call a heteroplasmy on the filtered read set: (1) at least 20 reads should be present in the set; (2) the minor allele (if it exists) should be present on least 15 % of the reads; and (3) the minor allele should be present on at least two reads of each strand.

### Heteroplasmy validation

MtDNA was extracted from 20 mg of flash-frozen liver tissue from each individual of interest, using a protocol adapted from Ahmad et al. [72]. Tissue samples were homogenized in 1 mL homogenization buffer (100 mM Tris-HCl pH 7.4, 250 mM sucrose, 10 mM EDTA) in a Precellys 24 homogenizer, with conditions 5000^–3^ × 30^–30^ and tubes CK14 (Bertin Technologies). Nuclei and cellular debris were removed by centrifugation (1500 g for 10 min at 4 °C), and the supernatant was centrifuged at 10,000 g for 10 min at 4 °C to obtain a crude mitochondrial pellet. Mitochondria were suspended in 480 uL of high salt buffer (Tris HCl 10 mM pH 7.6, 10 mM KCl, 10 mM MgCl2, 0.4 M NaCl, and 2 mM EDTA) plus 75 uL 10 % SDS, and incubated at 55 °C for 10 min for protein denaturation and solubilization. Proteins were precipitated by the addition of 200 uL 6 M NaCl and centrifugation at 11,300 g for 20 min. Finally, the supernatant containing mtDNA was precipitated with two volumes of 100 % ethanol, centrifuged for 10 min at 10,000 g at 4 °C, and washed twice with 70 % ethanol. The dried mtDNA pellet was resuspended in 100 uL EB buffer (Qiagen), quantified, and diluted to a final concentration of 100 ng/uL.

PCR primers were designed to amplify two independent mtDNA fragments (400-1000 bp) spanning each heteroplasmic position. A total of 100 ng of mtDNA were used as a template in a 50 uL reaction with Kapa HiFi PCR master mix (Kapa Biosystems) and the following conditions: 95 °C for 3 min; 20 cycles of 98 °C for 20s, 60 °C for 30 s, 72 °C for 1 min, 72 °C for 5 min, 4 °C and hold. Sanger sequencing was performed on each amplicon with primers proximal to the heteroplasmy, using at least two different primers per amplicon (Additional file 2: Table S3). Heteroplasmies were considered as robustly validated if both alleles could be clearly detected in the chromatograms of more than 50 % of successful Sanger sequencing reactions (14 positions). For an additional six positions, the minor allele could be detected at lower levels and frequencies, typically in one to three of the reactions (Additional file 2: Table S3).

PCR and sequencing primers for pyrosequencing assays were designed using PyroMark Assay Design 2.0. Each assay was designed to target mitochondrial regions of interest in each species. Primers were designed to ensure high specificity and optimal sequencing length as indicated by their PyroMark quality score. Final PCR reactions included 1.5 units of MyTaq™ HS DNA Polymerase (Bioline), 5× MyTaq Reaction Buffer, 0.27 uM of each forward and reverse primer, 10 ng of sample DNA and molecular grade water to a final volume of 50 uL reaction volume. PCR reactions were carried out on an MJ PTC 225 tetrad, 96-well block, in triplicate for each sample including a positive and negative control. PCR cycling conditions were as follows for all assays excluding assay 18; 95 °C for 1 min initial denaturation; 35 cycles of 95 °C for 15 s denaturation, 63 °C for 15 s primer annealing, and 72 °C for 10 s extension. Assay 18 cycled with an annealing temperature of 50 °C. After a quality check via Agarose Gel Electrophoresis, pyrosequencing was set up and performed on the PSQMA96 machine as per the PyroMark Gold Q96 Reagents Handbook (Qiagen). Following the sequencing runs analysis was performed using PyroMark ID 1.0 via Allele Quantification. Pyrosequencing primer sequences used for each assay can be found in Additional file 2: Table S5.

### Coverage and genomic context analysis

The mean coverage per individual was calculated using SAMtools depth, which provides the coverage for each base pair. The coverage ratio was calculated as the fraction of mtDNA bases that are covered by at least 20 reads. The resulting data are visible in Fig. 2. To assign the genomic context of each heteroplasmic position, the Ensembl Variant Effect Predictor [73] was used. For three species (*C. jacchus*, *M. furo*, and *O. garnettii*), annotations were not available and annotations of closely related species were used (*M. mulatta*, *C. familiaris*, and *M. mulatta*, respectively). The resulting data are visible in Fig. 5d.

### Heteroplasmy visualization

PRANK [74] with the genomic model was used to generate a multiple alignment of each species’ mtDNA. A trimmed species tree extracted from the Ensembl (v. 81) species tree was used as a guide tree. The human mtDNA gene annotation was downloaded from Ensembl (v. 81) BioMart. Jalview [75] was used to visualize heteroplasmic positions for every species displayed on the previously generated alignment (Fig. 3). Jalview was also used to display heteroplasmies in their sequence context (Fig. 4).

### Low complexity regions

There are five low complexity regions in the human mtDNA (66 to 71, 303 to 309, 514 to 523, 12,418 to 12,425, and 16,184 to 16,193) [18]. To count the number of heteroplasmic positions occurring within these regions across all species, we used the PRANK generated multiple mtDNA alignment to map the non-human heteroplasmic positions to the orthologous human coordinates.

### Disease-associated positions

Human positions were directly compared to MITOMAP annotations to identify potential disease associations. For other species, orthologous human positions for each heteroplasmy were first identified using the UCSC Batch Coordinate Conversion (liftover) tool [76] and then compared to the MITOMAP annotations.

### ChIP-seq protein binding assay

We performed peak detection with the Model-based analysis of ChIP-seq (MACS) tool [77] for a range of experiments covering nine species (*H. sapiens*, *M. mulatta*, *C. jacchus*, *M. musculus*, *R. norvegicus*, *O. cuniculus*, *C. familiaris*, *F. catus*, and *G. gallus*) and four different proteins (CEBPA, CTCF, FOXA1, and YY1). No peaks where reported on the mtDNA genome in all cases.

### Human contamination test

To test if the detection method was to subject false positives by cross-species contamination, we simulated human contamination *in silico* by creating a mixture of *H. sapiens* (human) and *R. norvegicus* (rat) data. By adding random human sequencing reads to a rat sequence file, which did not contain any heteroplasmies, we created mixture files containing 1 % and 10 % of human DNA. No heteroplasmies were detected by our method in these artificially contaminated files.

## Supporting data

All the data used in this study are accessible on ArrayExpress, under the following accession numbers.

New data from this study:

- CEBPA, H3K4me1, H3K27ac, and Histone3 total ChIP-seq data: **E-MTAB-3933**

Previously published data:

- HNF4A and CEBPA ChIP-seq data [44]: **E-TABM-722**
- CTCF, SA1, NRSF/REST and H2AK5ac ChIP-seq data [45]: **E-MTAB-437**
- CTCF and YY1 ChIP-seq data [46]: **E-MTAB-1511**
- CEBPA, FOXA1, ONECUT1, and HNF4A ChIP-seq data [47]: **E-MTAB-1509**
- H3K4me3 and H3K27ac ChIP-seq [48]: **E-MTAB- 2633**
- Direct links to raw data and other supporting information are available from http://www.ebi.ac.uk/research/flicek/publications/FOG17.

## Additional files

Additional file 1: Supplementary figures S1–S3. **Figure S1.** Detailed coverage data for all individuals. **Figure S2.** Read coverage of heteroplasmic positions. **Figure S3.** Coverage data of different ChIP-seq data files. (PDF 20125 kb) Additional file 2: Supplementary tables S1–S4. **Table S1.** Detected heteroplasmies of excluded individuals. **Table S2.** Detected heteroplasmies. **Table S3.** Sanger sequencing and pyrosequencing validation results. **Table S4.** Additional details for each file. **Table S5.** Raw pyrosequencing data. (XLS 352 kb) Additional file 3: Heteroplasmy detection algorithm and analysis in Python. (ZIP 115 kb)
