# Supplementary material for: Mitochondrial heteroplasmy in vertebrates using ChIP-sequencing data
Source: Genome Biol. 2016 Jun 27;17:139. doi: 10.1186/s13059-016-0996-y (PMC4922064; doi:10.1186/s13059-016-0996-y)
Supplement: Additional file 1: — Supplementary figures S1–S3. Figure S1. Detailed coverage data for all individuals. Figure S2. Read coverage of heteroplasmic positions. Figure S3. Coverage data of different ChIP-seq data files. (PDF 20125 kb) [file 13059_2016_996_MOESM1_ESM.pdf]

Supplementary file 1

Contents:

Figure S1: Detailed coverage data for all individuals (pages 2-118)

Figure S2. Read coverage of heteroplasmic positions. (page 119)

Figure S3. Coverage data of different ChIP-seq data files. (page 120)

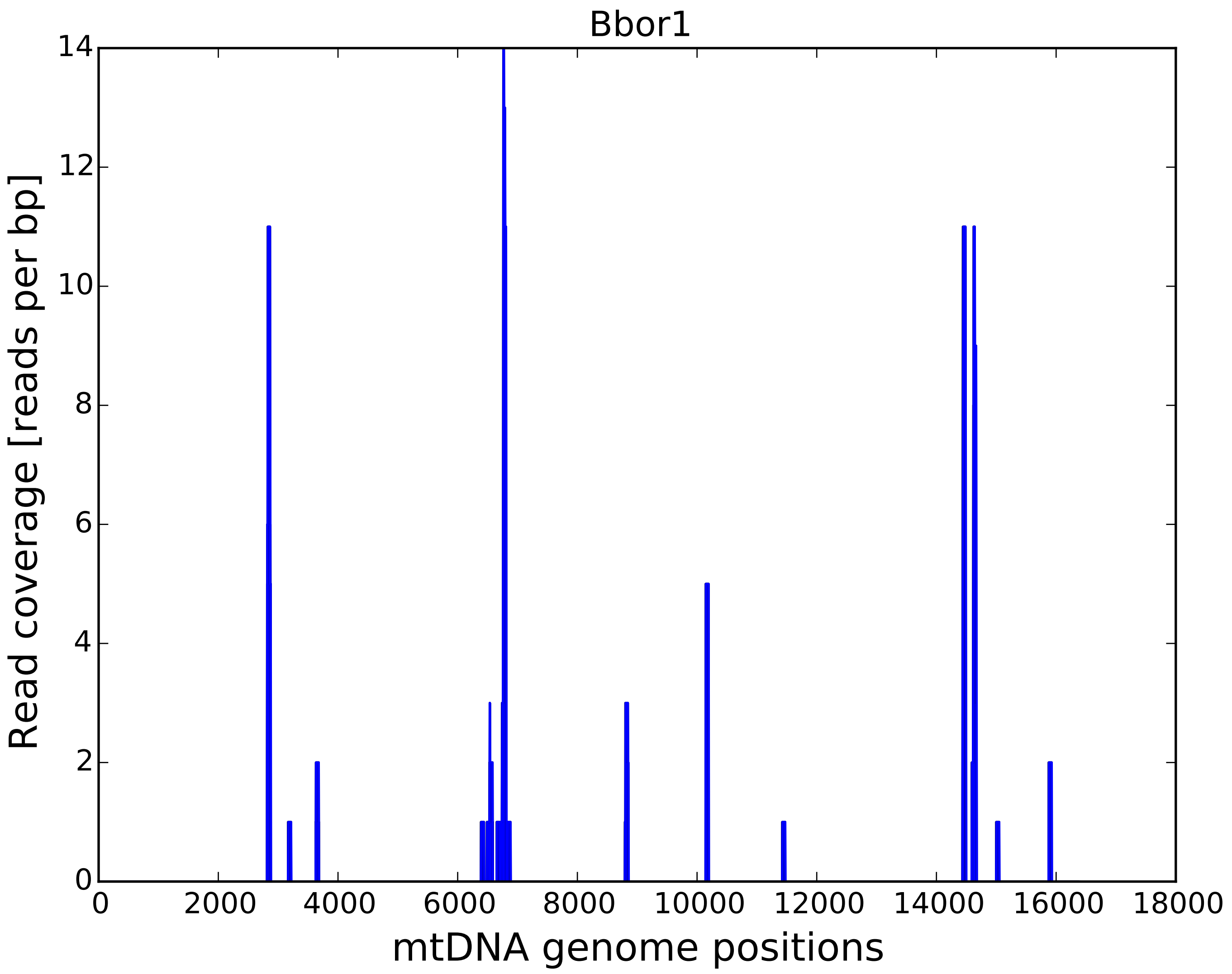

Bbor1
Read coverage [reads per bp]
mtDNA genome positions
0
2
4
6
8
10
12
14
0
2000
4000
6000
8000
10000
12000
14000
16000
18000

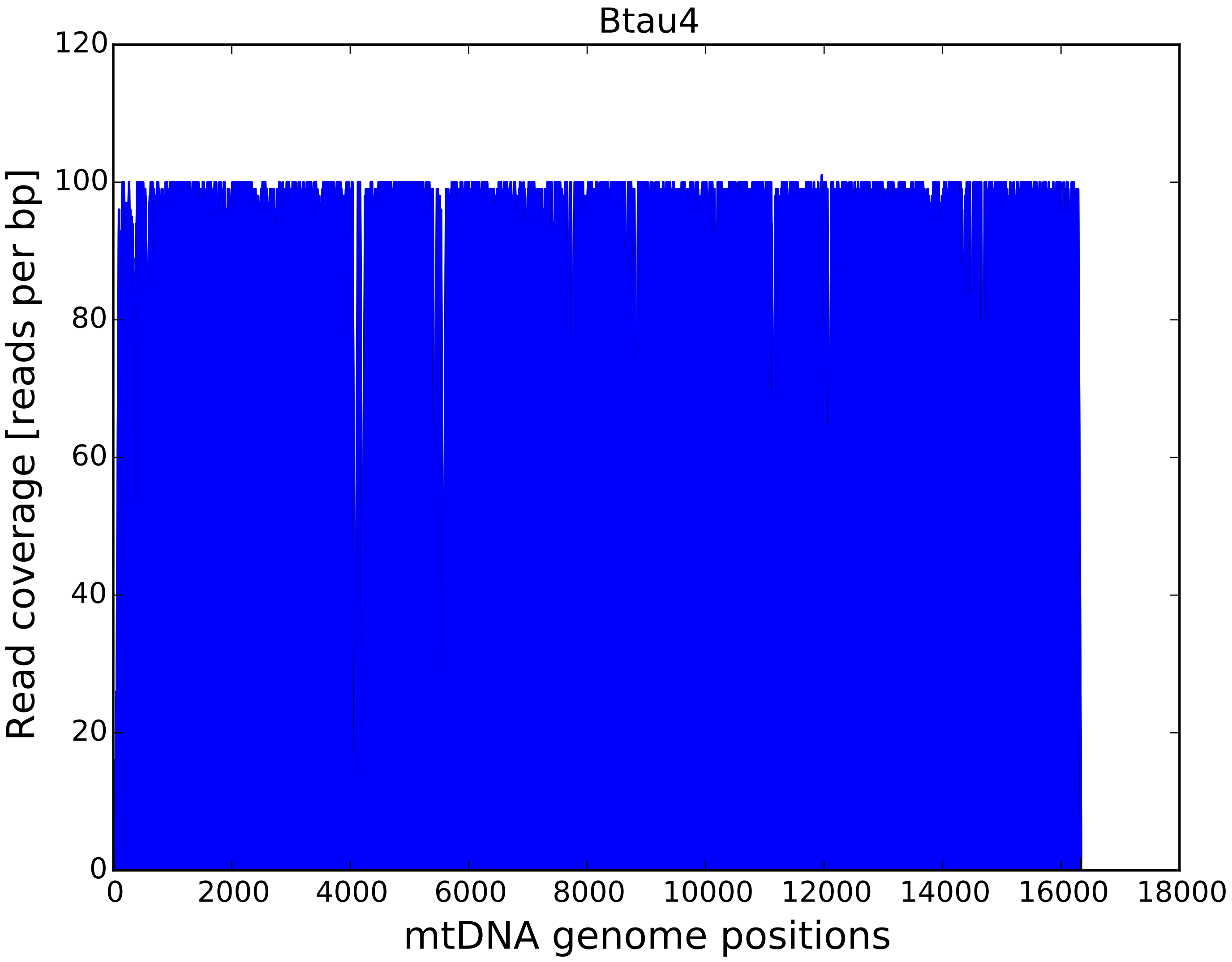

Btau4
Read coverage [reads per bp]
mtDNA genome positions
0
20
40
60
80
100
120
0
2000
4000
6000
8000
10000
12000
14000
16000
18000

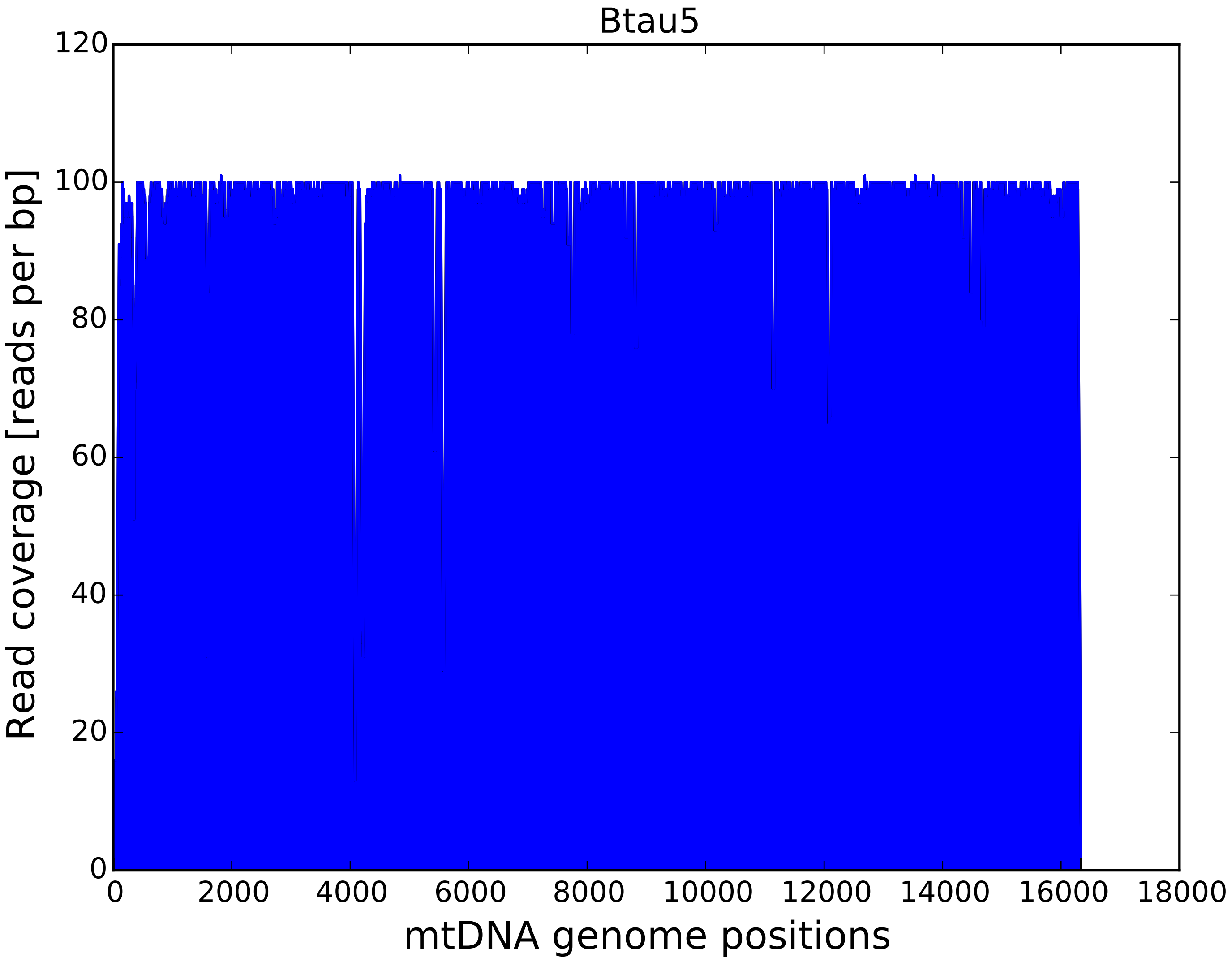

Btau5
Read coverage [reads per bp]
mtDNA genome positions
0
20
40
60
80
100
120
0
2000
4000
6000
8000
10000
12000
14000
16000
18000

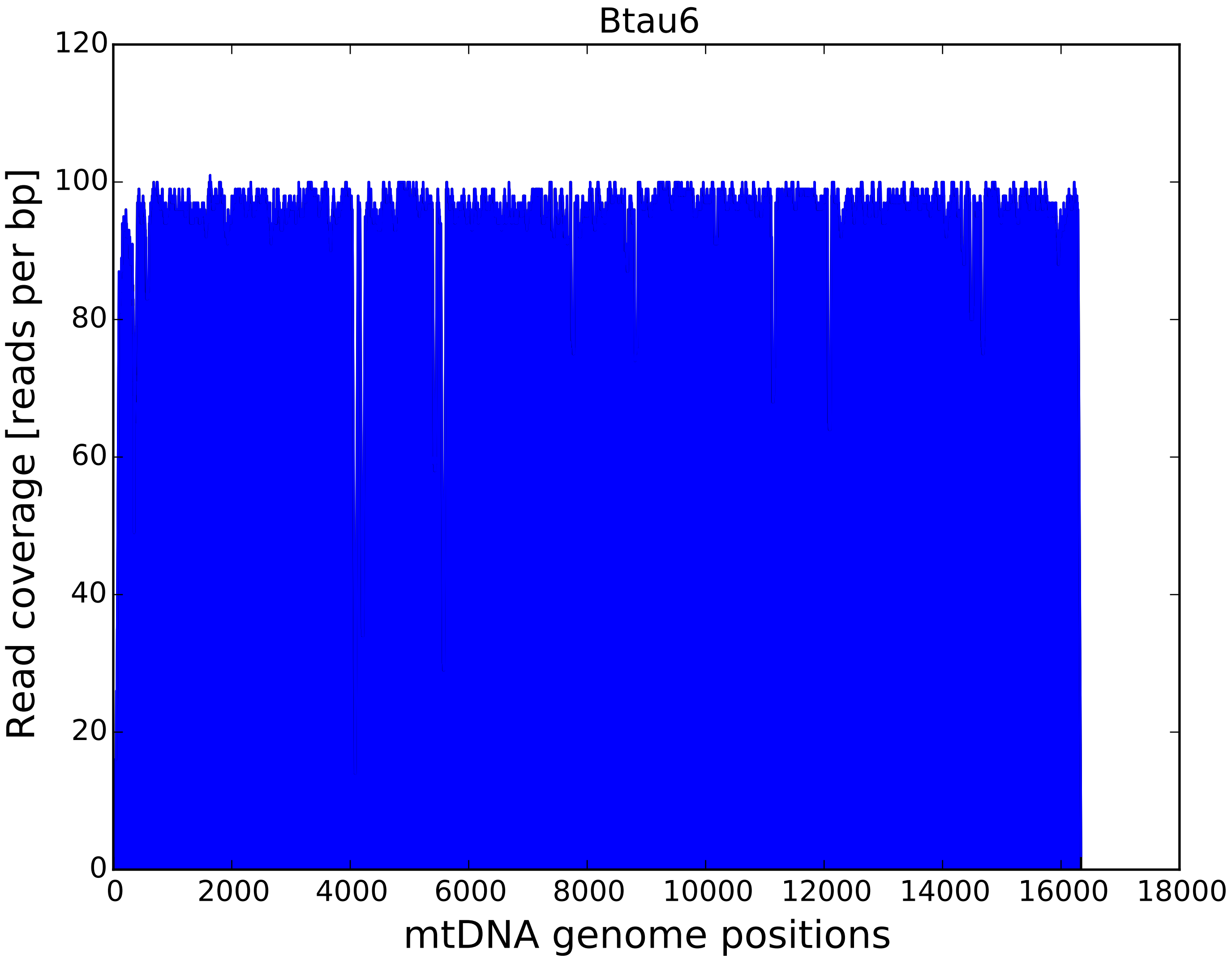

Btau6
Read coverage [reads per bp]
mtDNA genome positions
0
20
40
60
80
100
120
0
2000
4000
6000
8000
10000
12000
14000
16000
18000

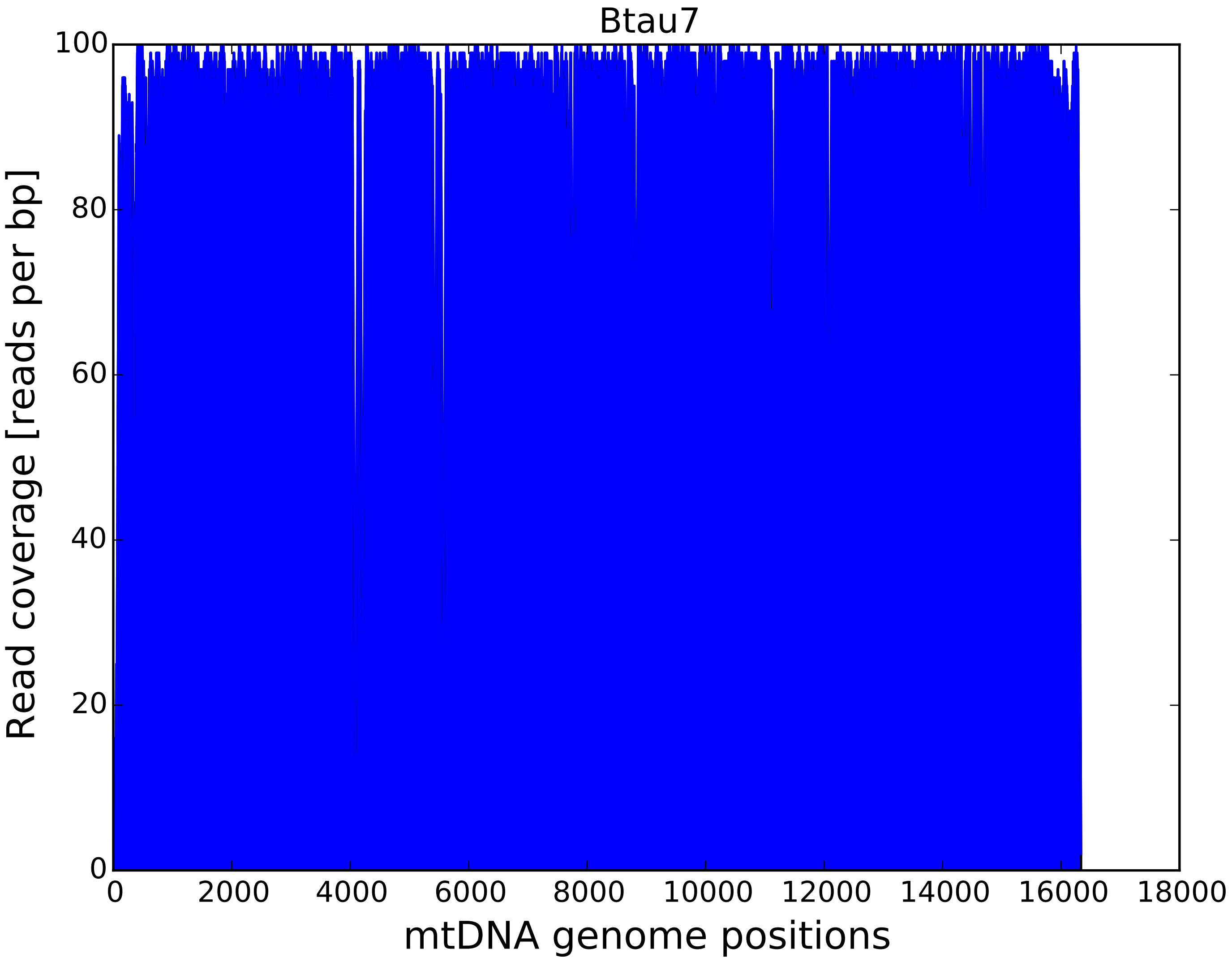

Btau7
Read coverage [reads per bp]
mtDNA genome positions
0
20
40
60
80
100
0
2000
4000
6000
8000
10000
12000
14000
16000
18000

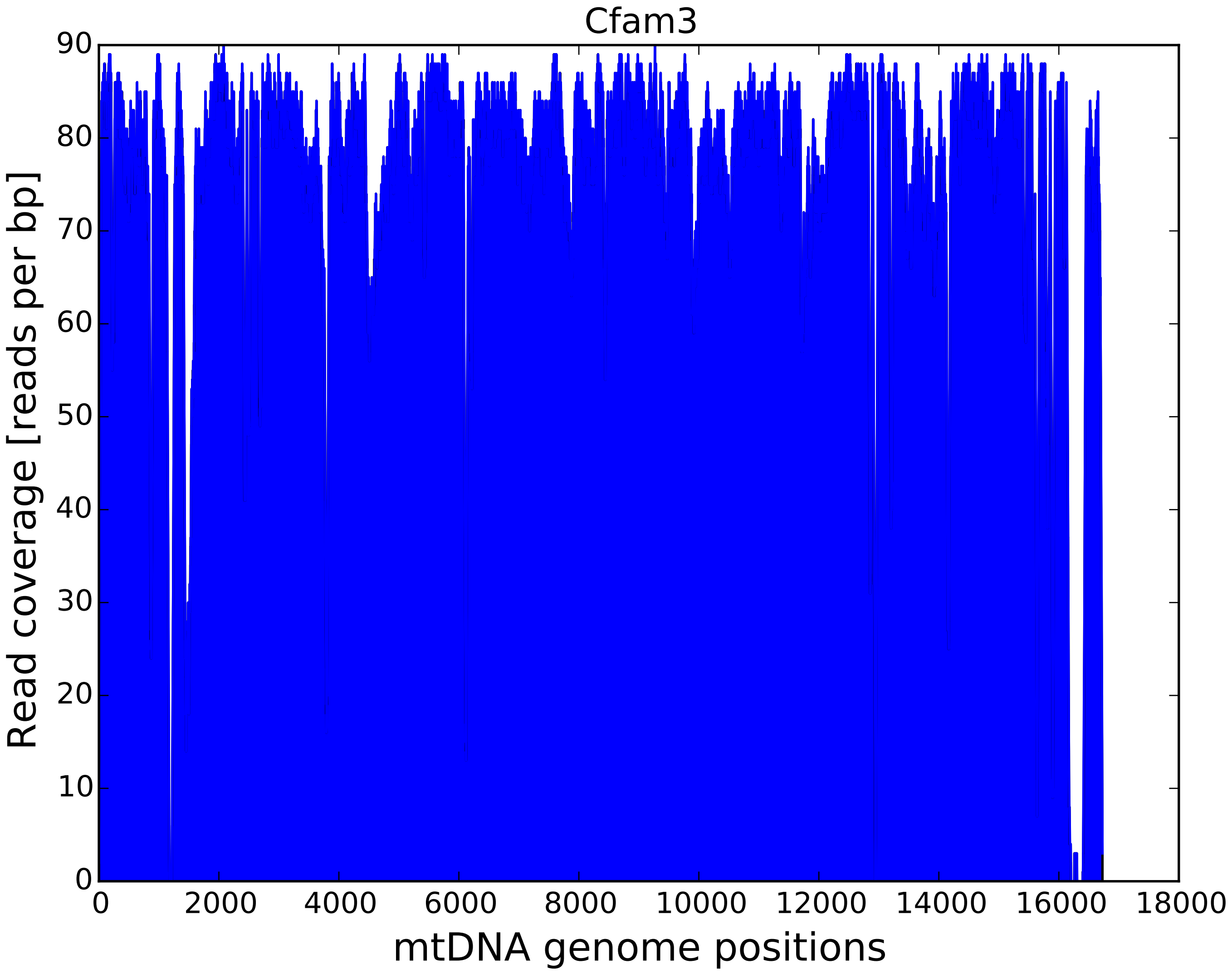

Cfam3
Read coverage [reads per bp]
mtDNA genome positions
0
10
20
30
40
50
60
70
80
90
2000
4000
6000
8000
10000
12000
14000
16000
18000

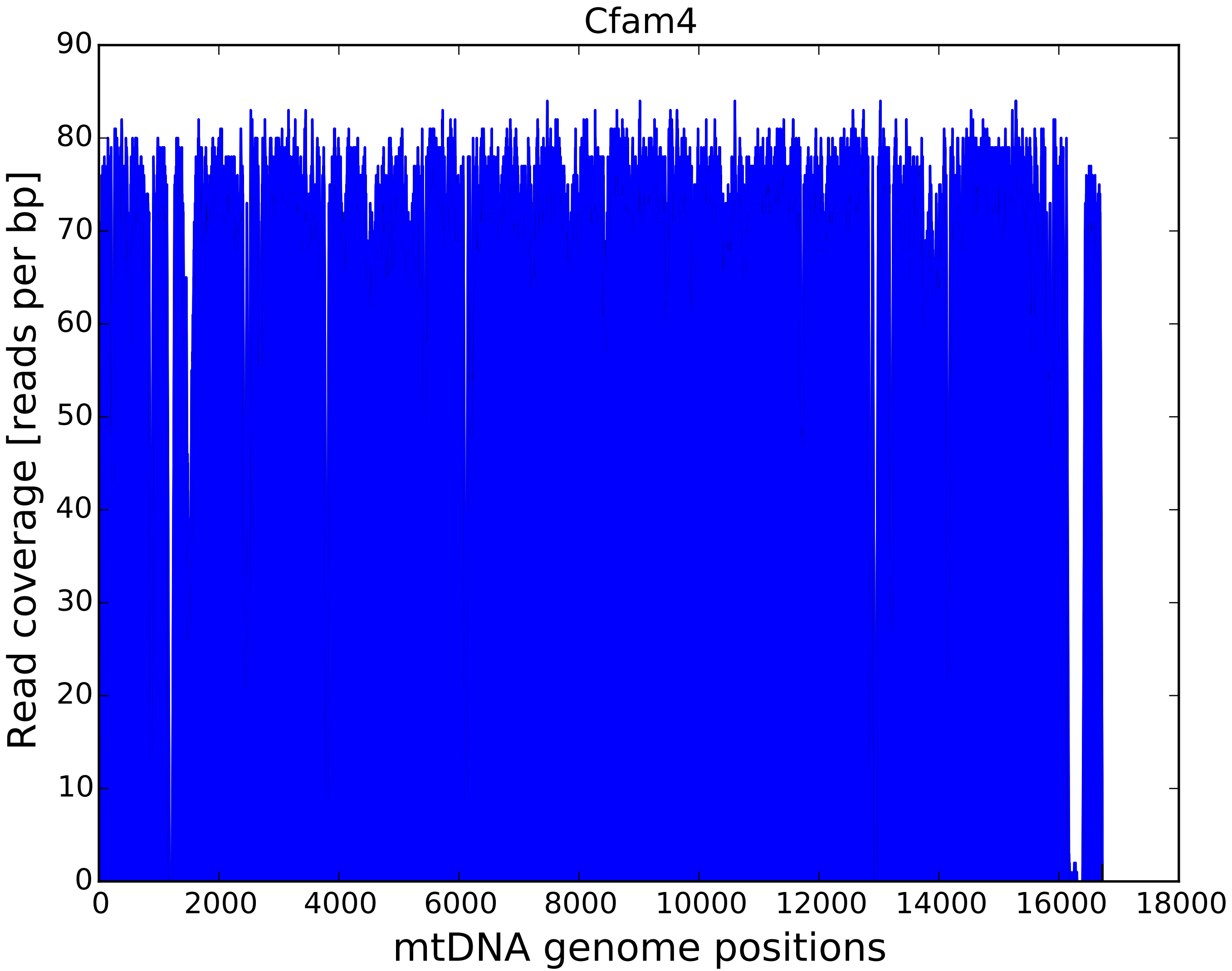

Cfam4
Read coverage [reads per bp]
mtDNA genome positions
0
10
20
30
40
50
60
70
80
90
0
2000
4000
6000
8000
10000
12000
14000
16000
18000

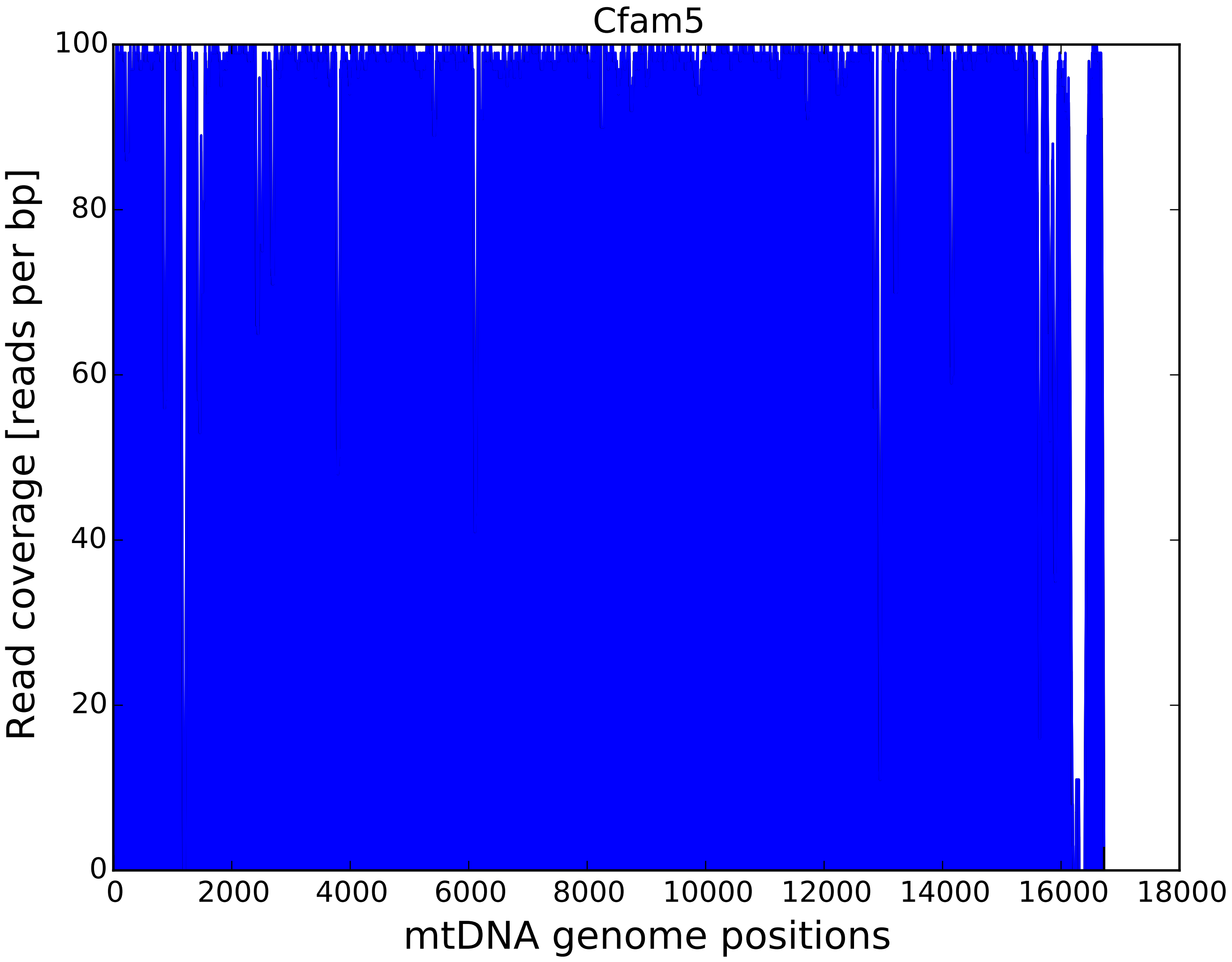

Cfam5
Read coverage [reads per bp]
mtDNA genome positions
0
20
40
60
80
100
0
2000
4000
6000
8000
10000
12000
14000
16000
18000

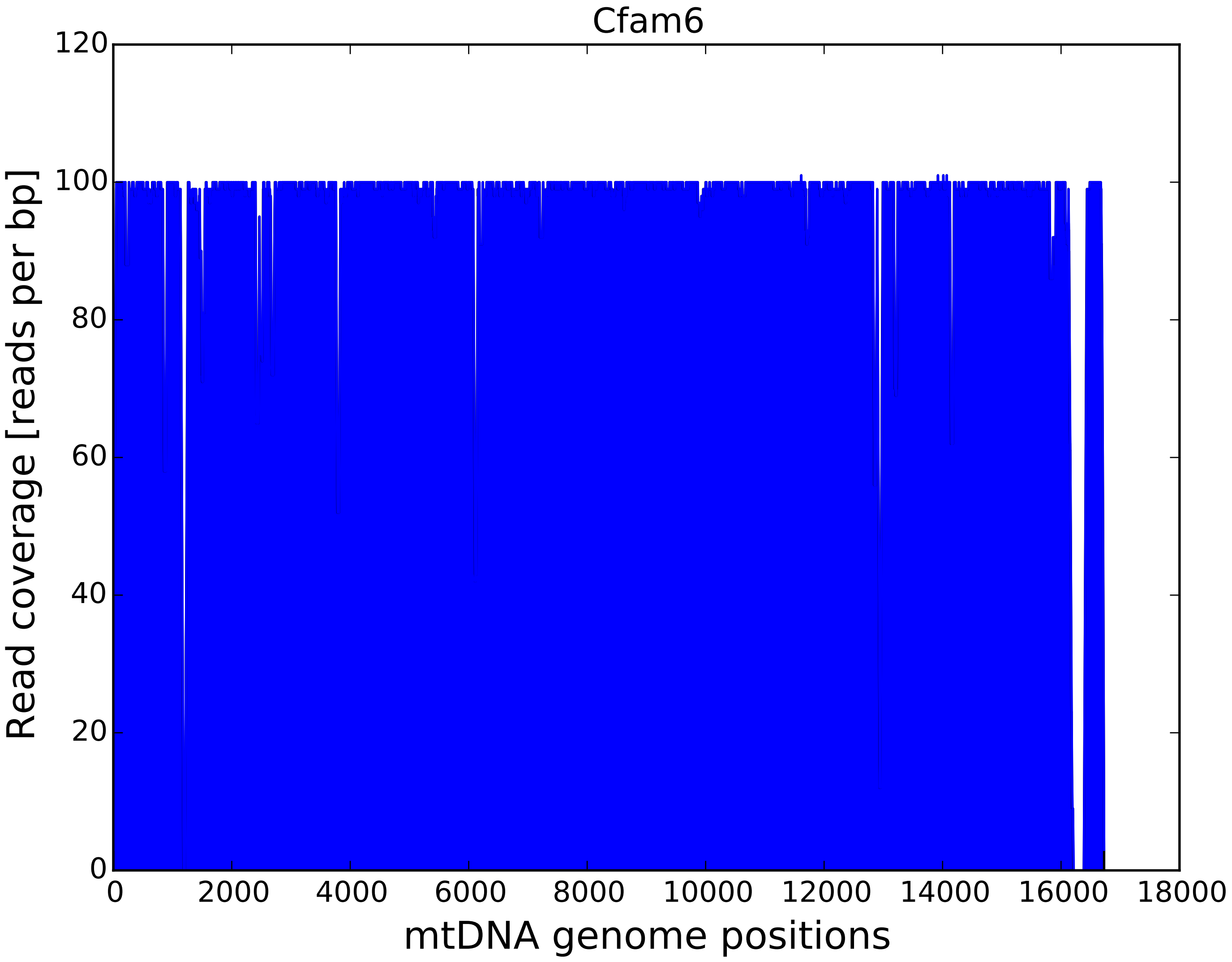

Cfam6
Read coverage [reads per bp]
120
100
80
60
40
20
0
0
2000
4000
6000
8000
10000
12000
14000
16000
18000
mtDNA genome positions

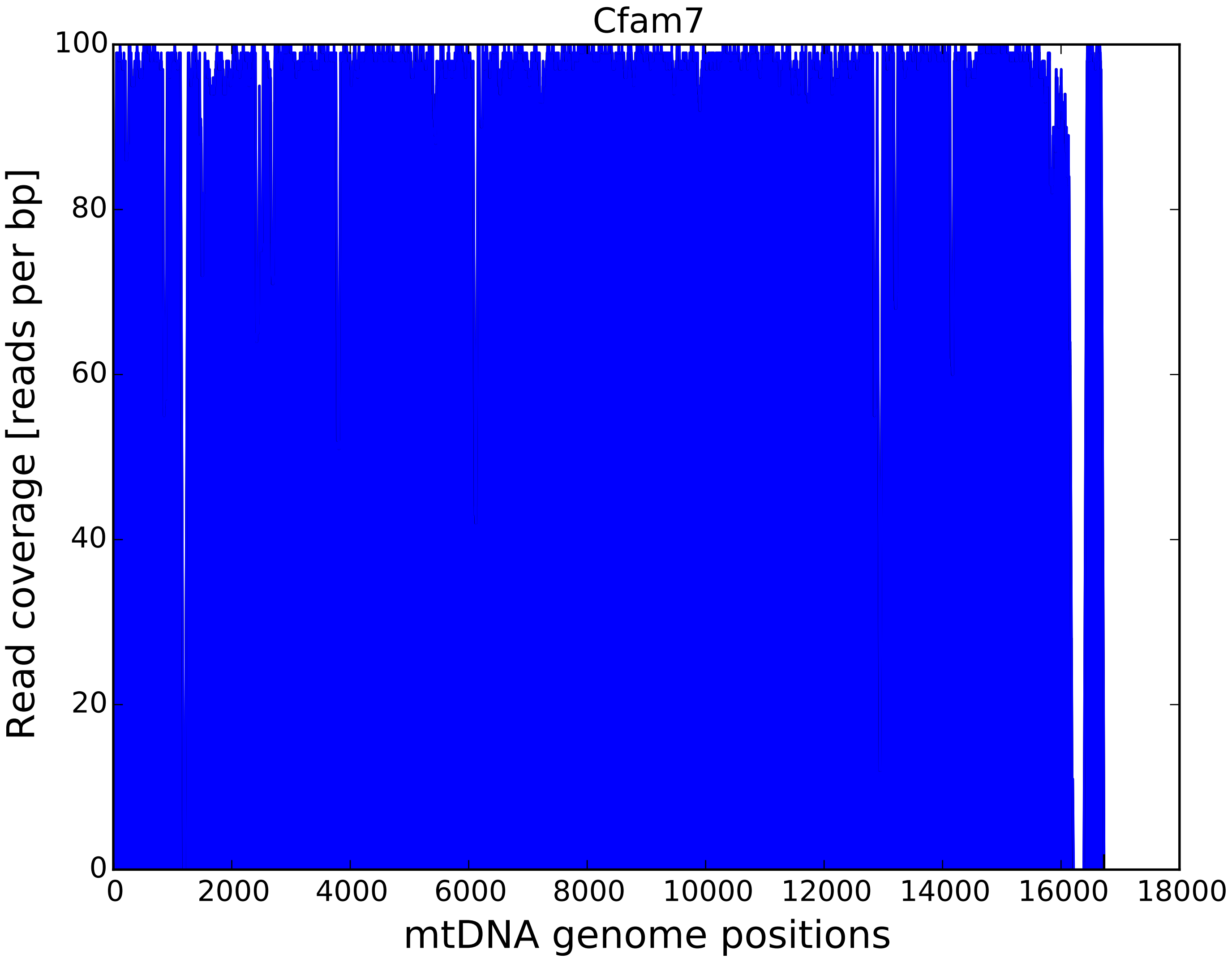
Cfam7
Read coverage [reads per bp]
mtDNA genome positions
0
20
40
60
80
100
0
2000
4000
6000
8000
10000
12000
14000
16000
18000

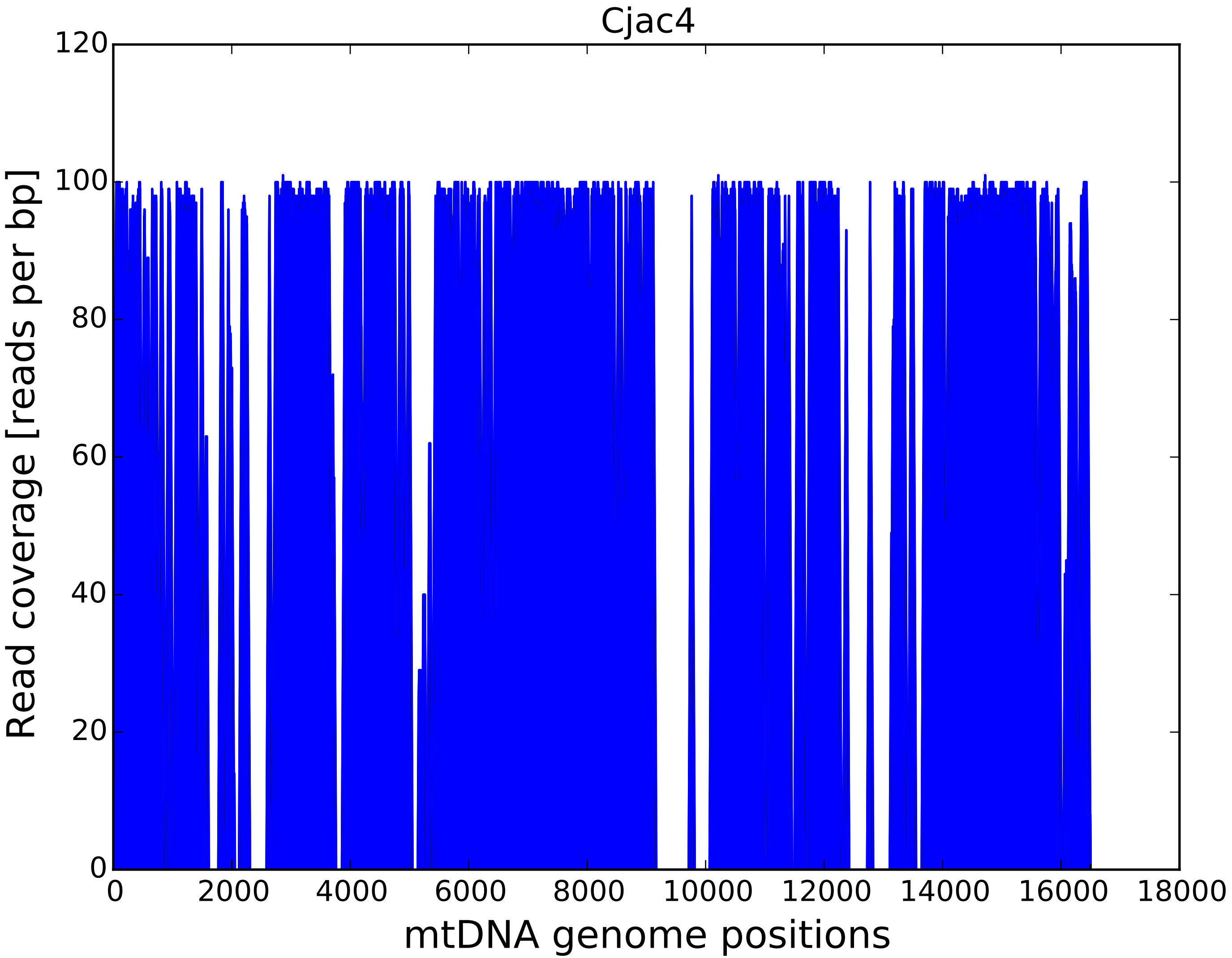
Cjac4
Read coverage [reads per bp]
mtDNA genome positions
0
20
40
60
80
100
120
0
2000
4000
6000
8000
10000
12000
14000
16000
18000

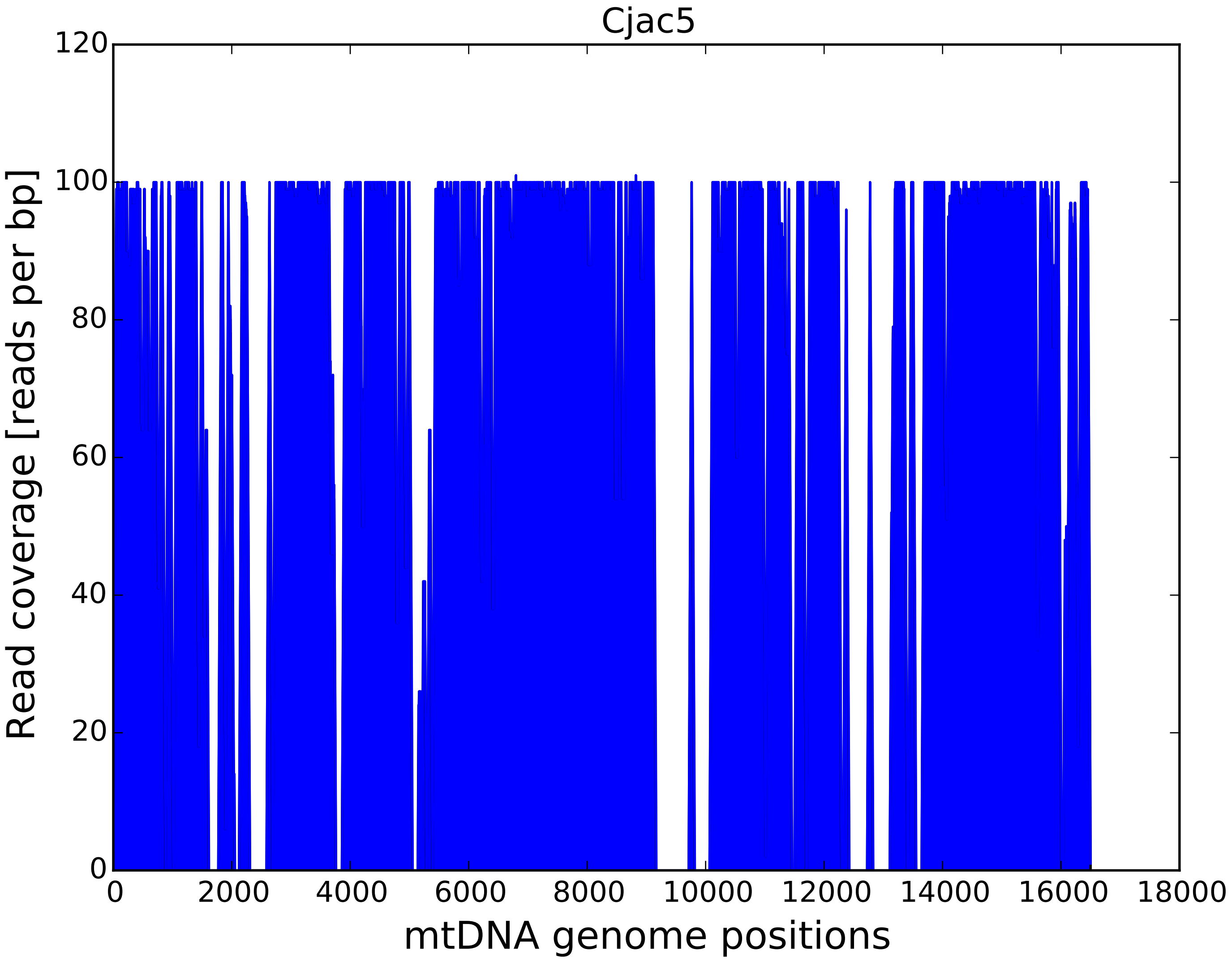
Cjac5
Read coverage [reads per bp]
mtDNA genome positions
0
20
40
60
80
100
120
0
2000
4000
6000
8000
10000
12000
14000
16000
18000

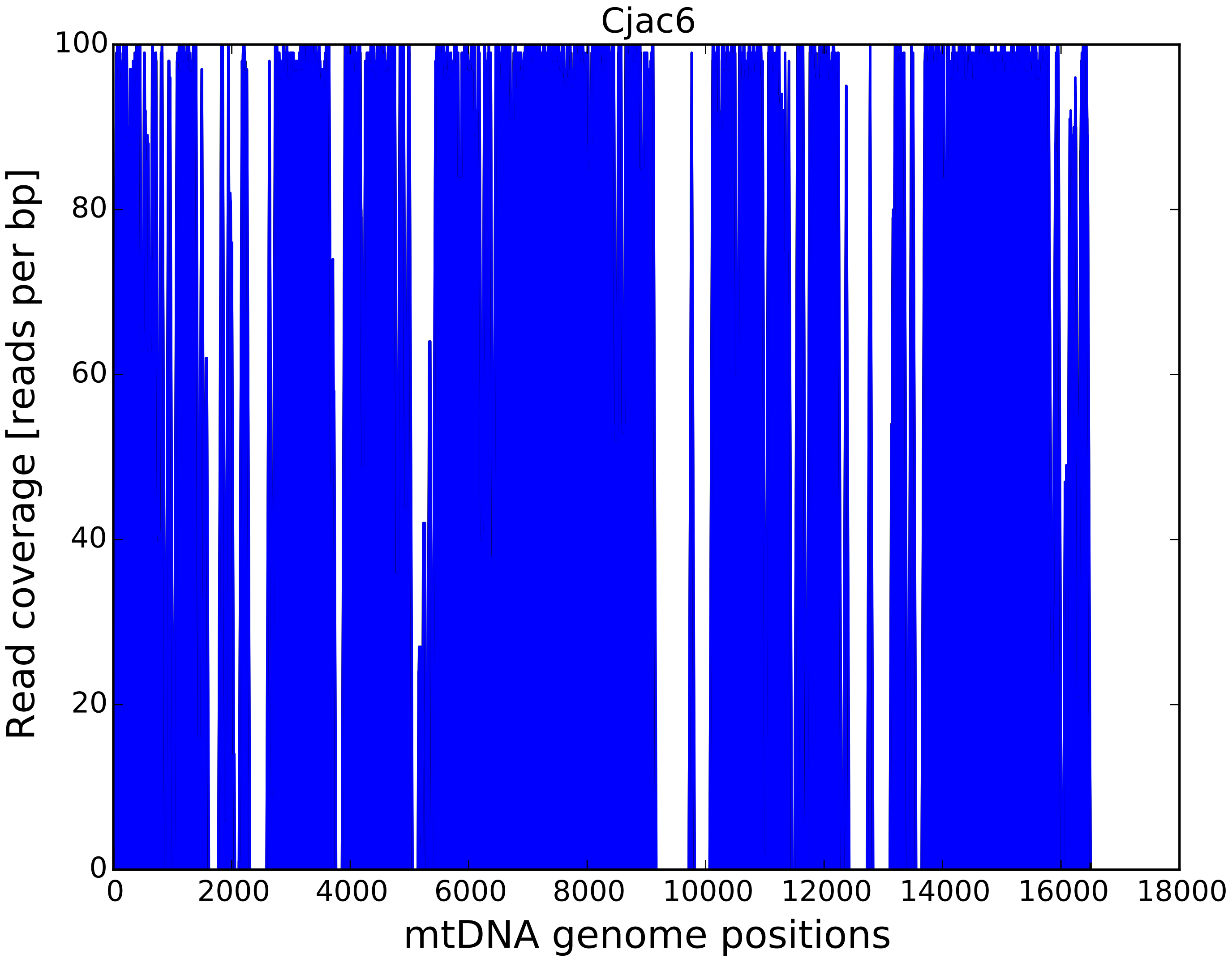
Cjac6
Read coverage [reads per bp]
mtDNA genome positions
0
20
40
60
80
100
0
2000
4000
6000
8000
10000
12000
14000
16000
18000

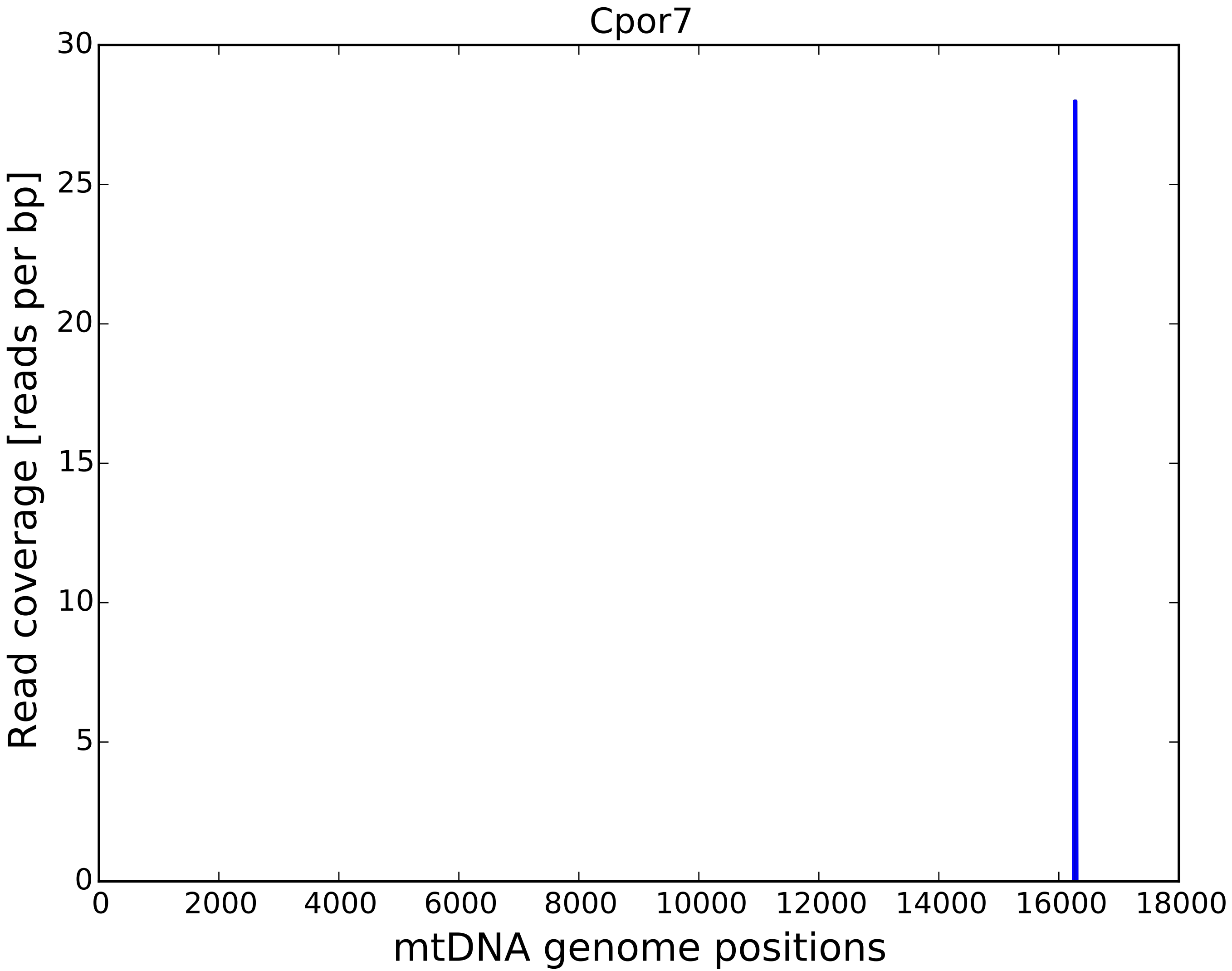
Cpor7
Read coverage [reads per bp]
mtDNA genome positions
0
5
10
15
20
25
30
0
2000
4000
6000
8000
10000
12000
14000
16000
18000

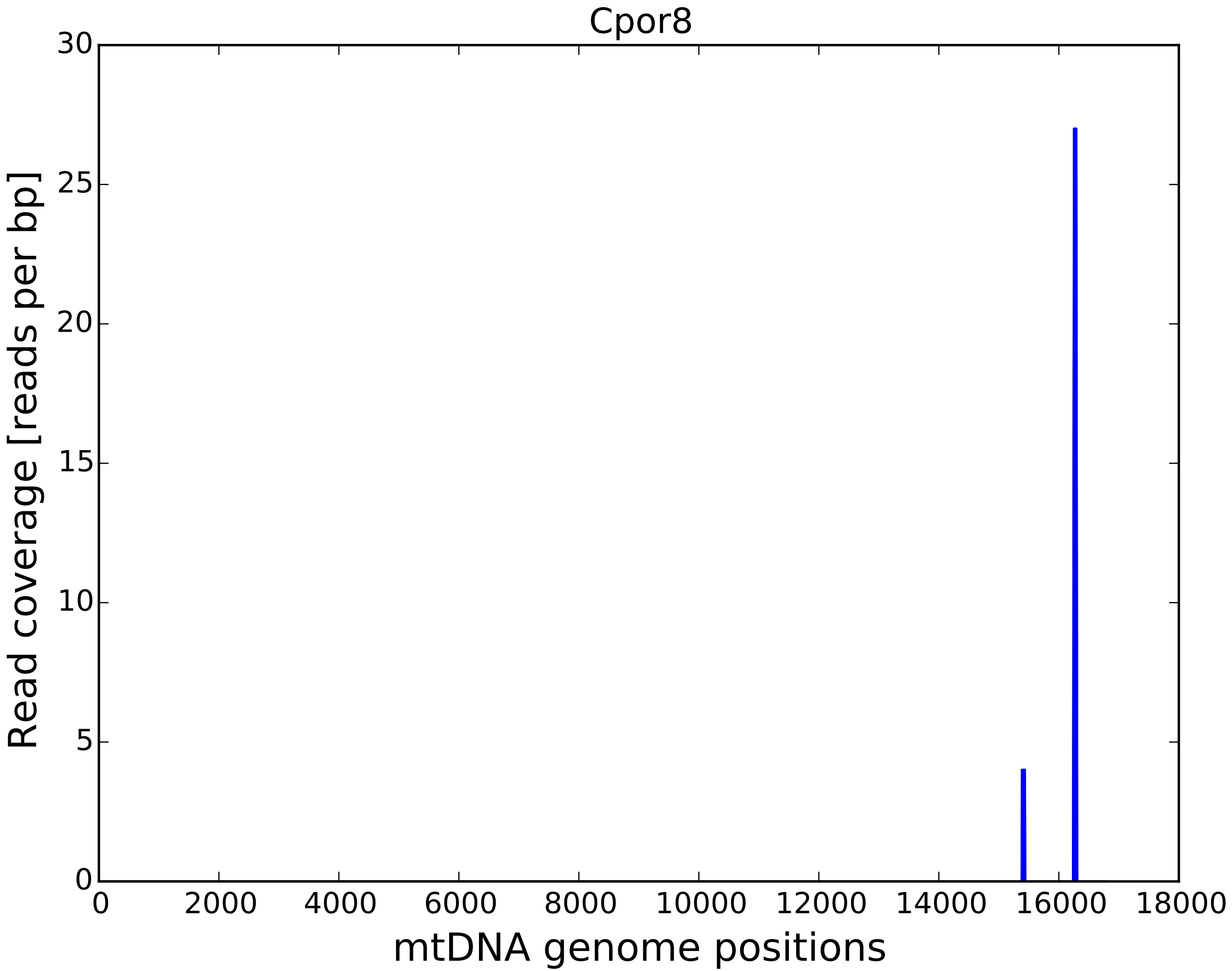

Cpor8
Read coverage [reads per bp]
mtDNA genome positions
0
5
10
15
20
25
30
0
2000
4000
6000
8000
10000
12000
14000
16000
18000

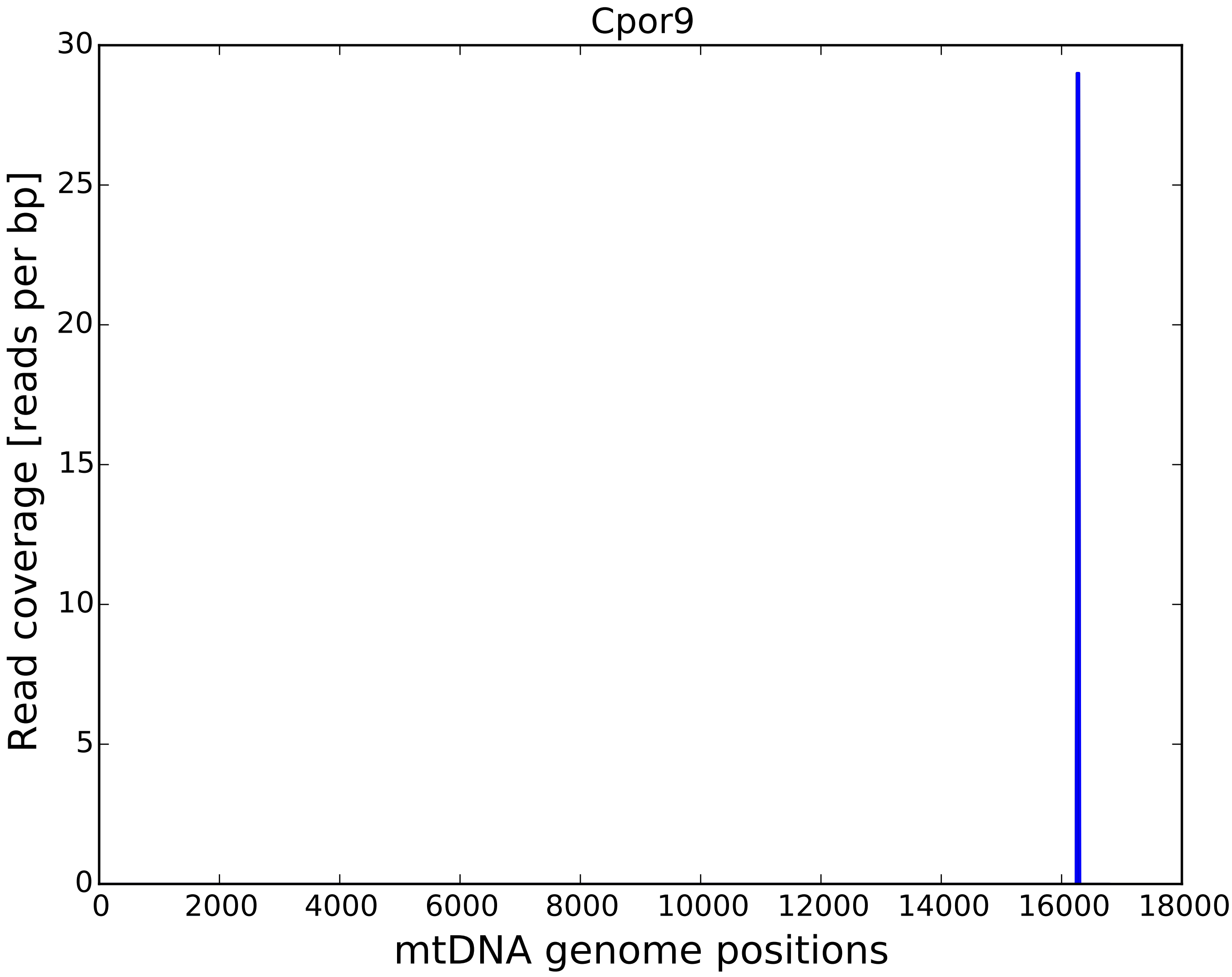
Cpor9
Read coverage [reads per bp]
mtDNA genome positions
0
5
10
15
20
25
30
0
2000
4000
6000
8000
10000
12000
14000
16000
18000

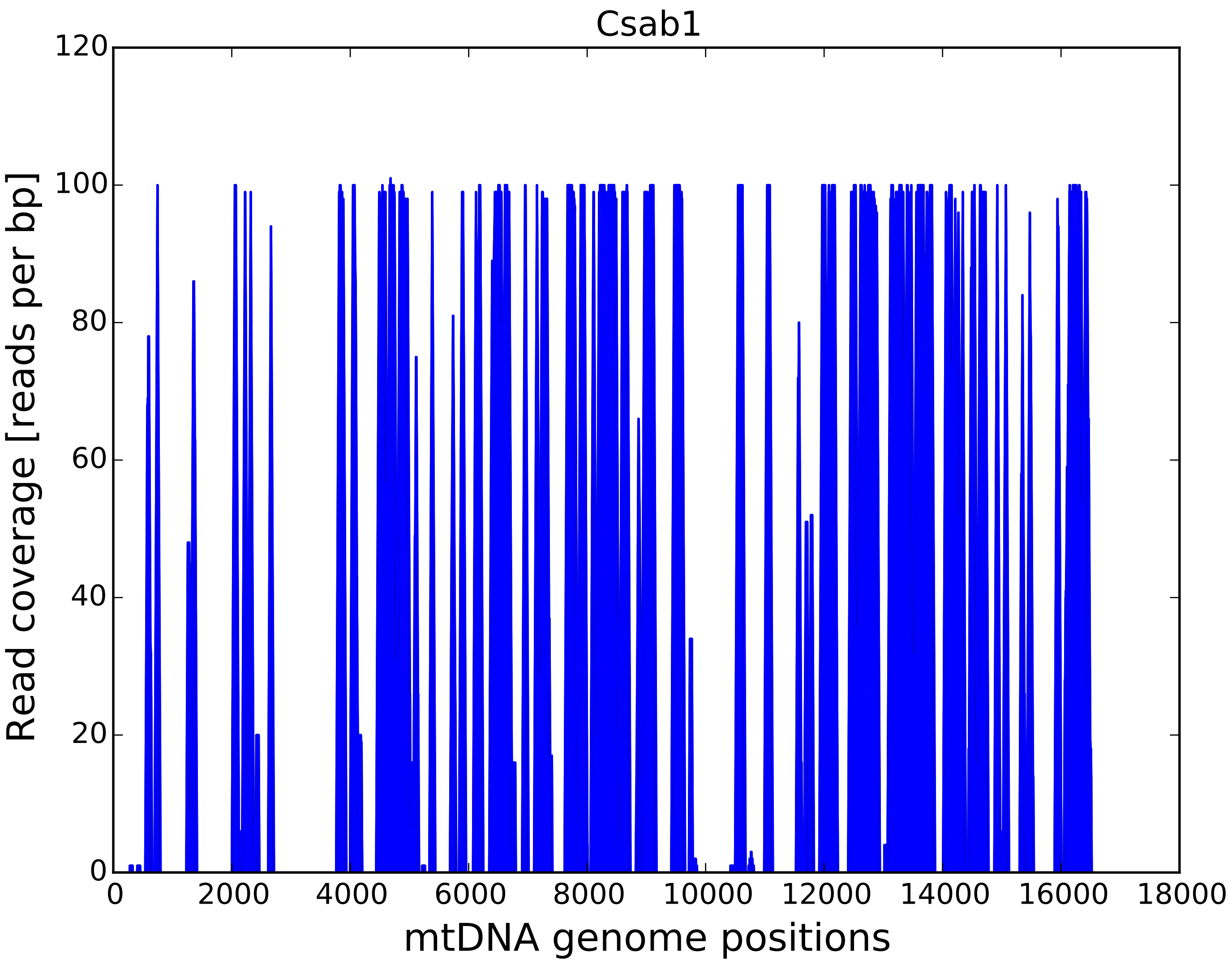

Csab1
Read coverage [reads per bp]
mtDNA genome positions
0
20
40
60
80
100
120
0
2000
4000
6000
8000
10000
12000
14000
16000
18000

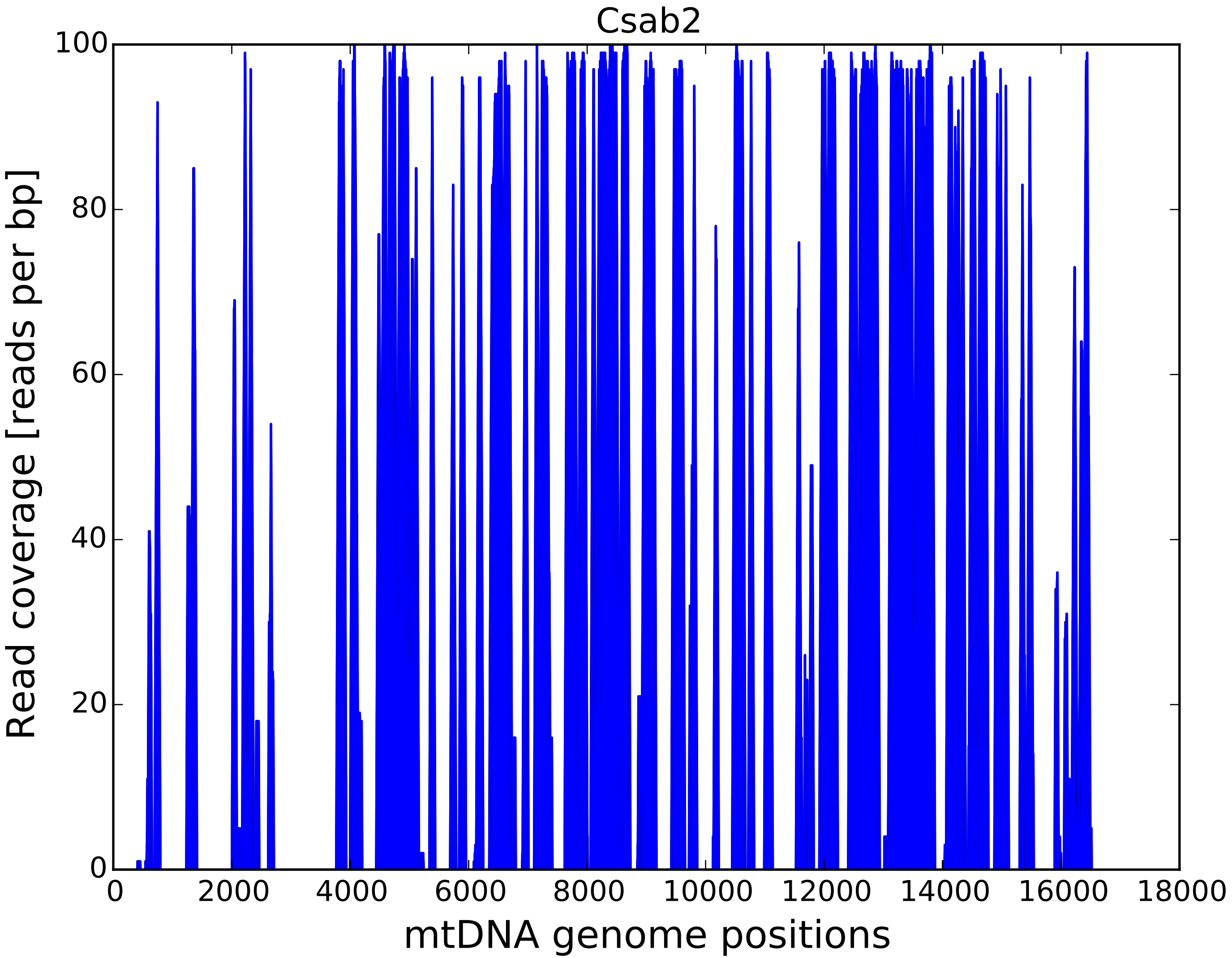
Csab2
Read coverage [reads per bp]
mtDNA genome positions
0
20
40
60
80
100
0
2000
4000
6000
8000
10000
12000
14000
16000
18000

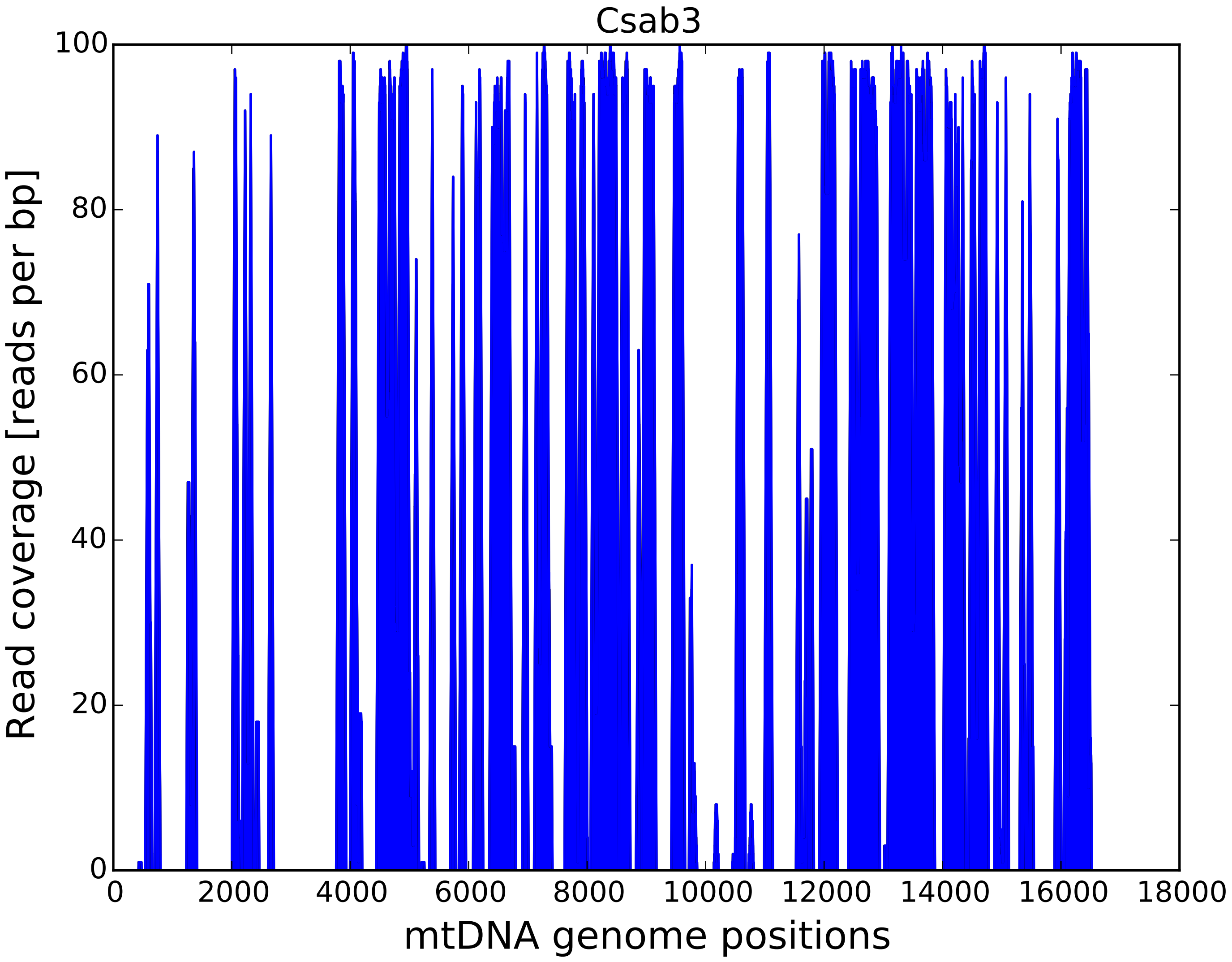
Csab3
Read coverage [reads per bp]
0
20
40
60
80
100
0
2000
4000
6000
8000
10000
12000
14000
16000
18000
mtDNA genome positions

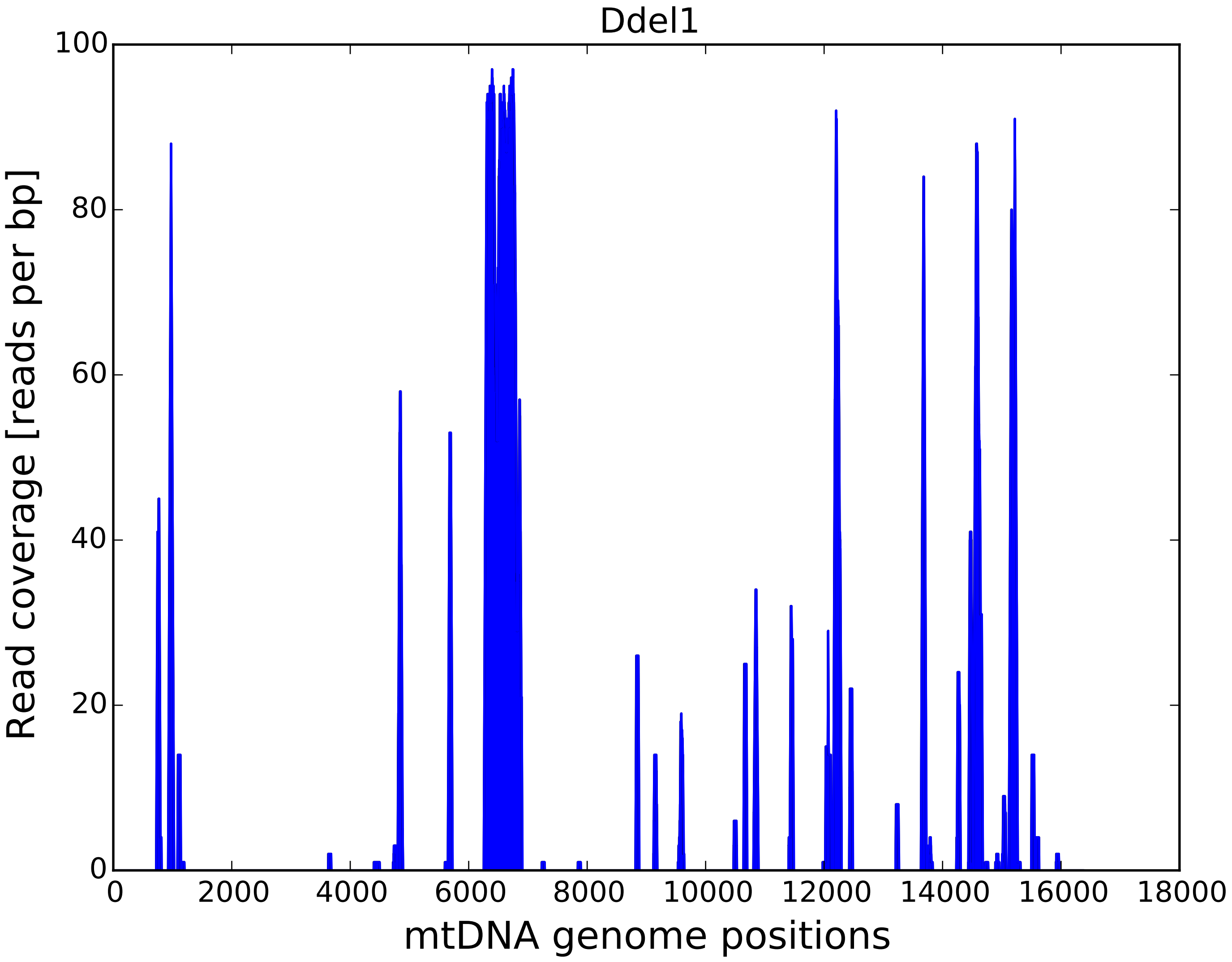

Ddel1
Read coverage [reads per bp]
mtDNA genome positions
0
20
40
60
80
100
0
2000
4000
6000
8000
10000
12000
14000
16000
18000

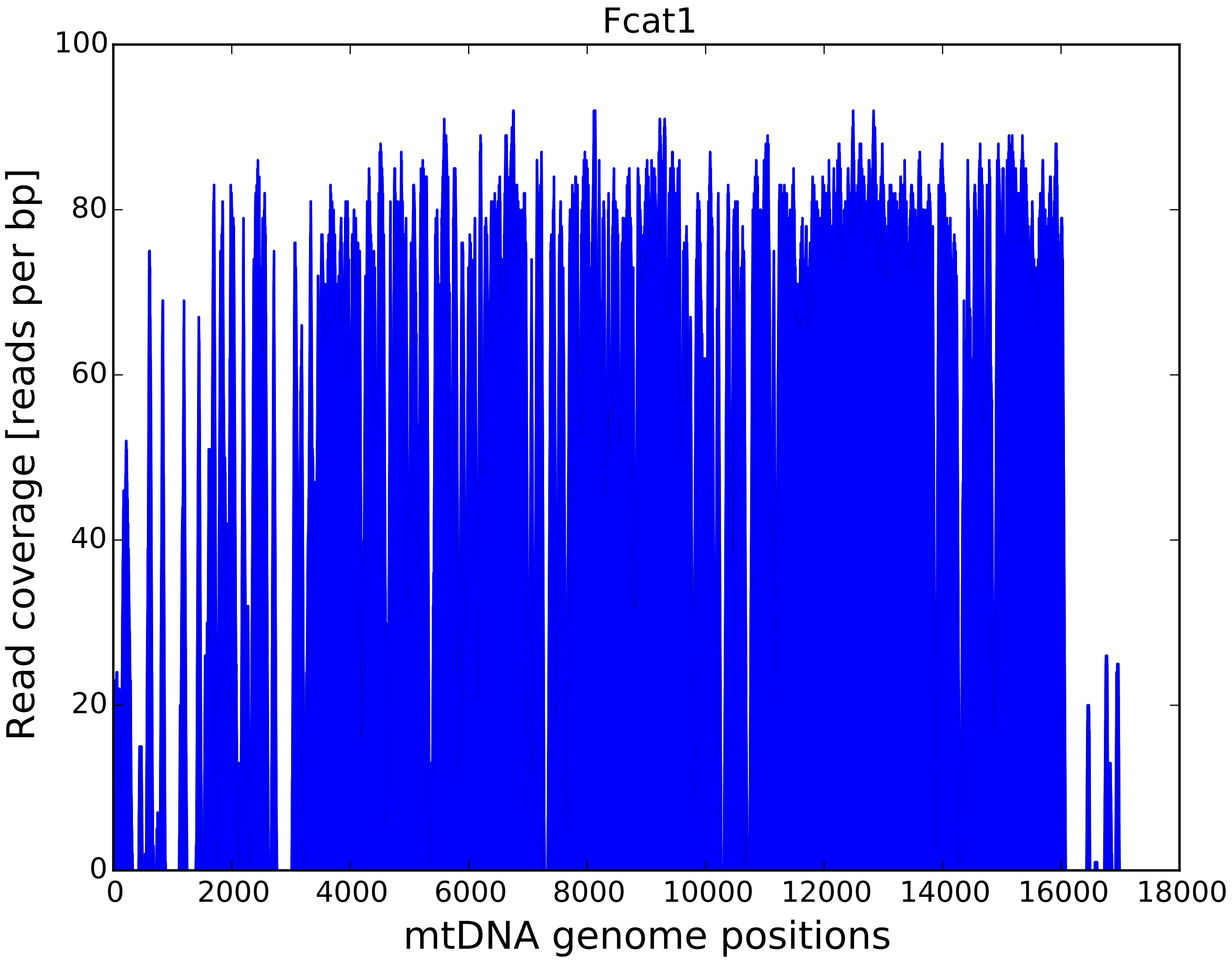

Fcat1
Read coverage [reads per bp]
0
20
40
60
80
100
0
2000
4000
6000
8000
10000
12000
14000
16000
18000
mtDNA genome positions

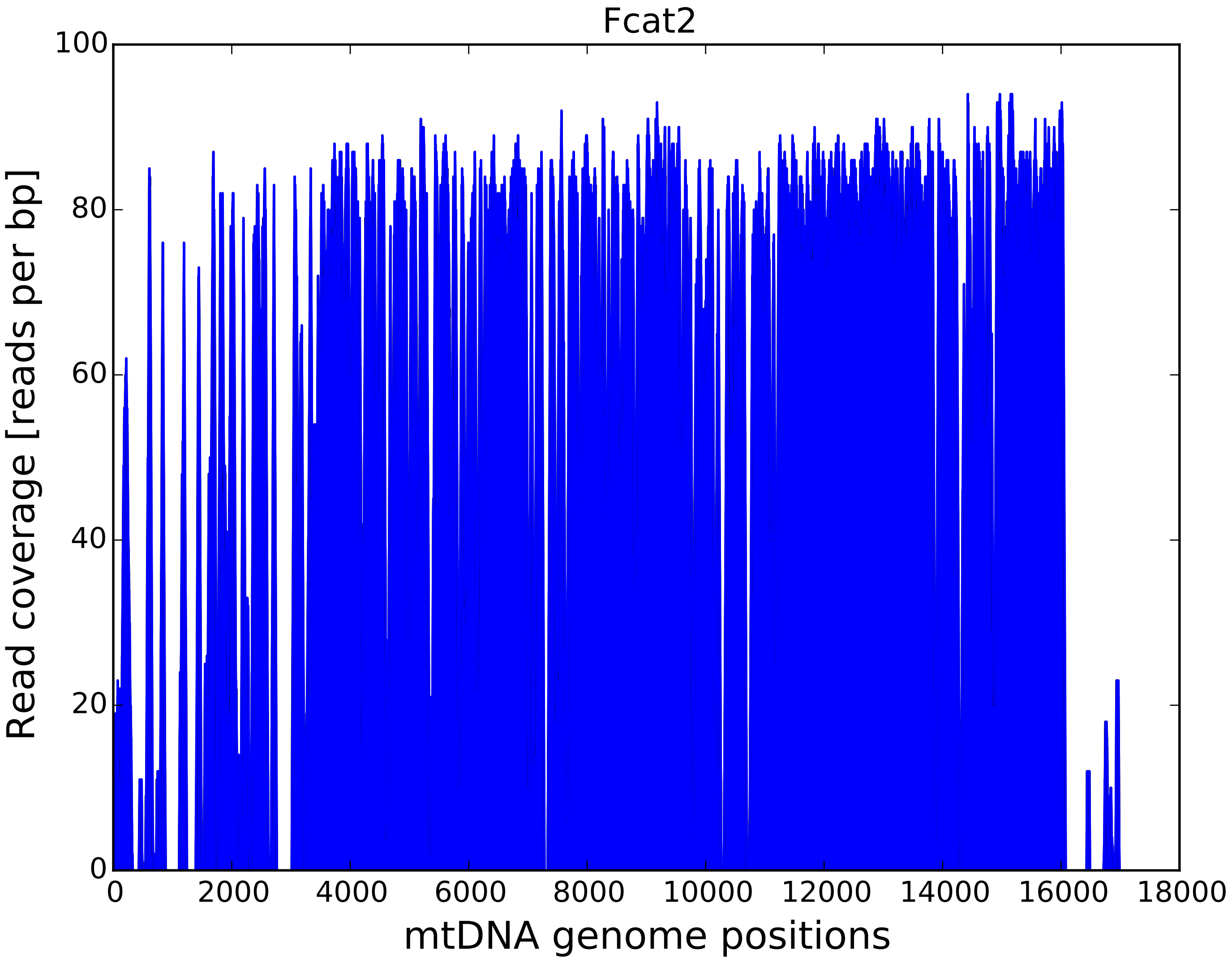
Fcat2
Read coverage [reads per bp]
mtDNA genome positions
0
20
40
60
80
100
0
2000
4000
6000
8000
10000
12000
14000
16000
18000

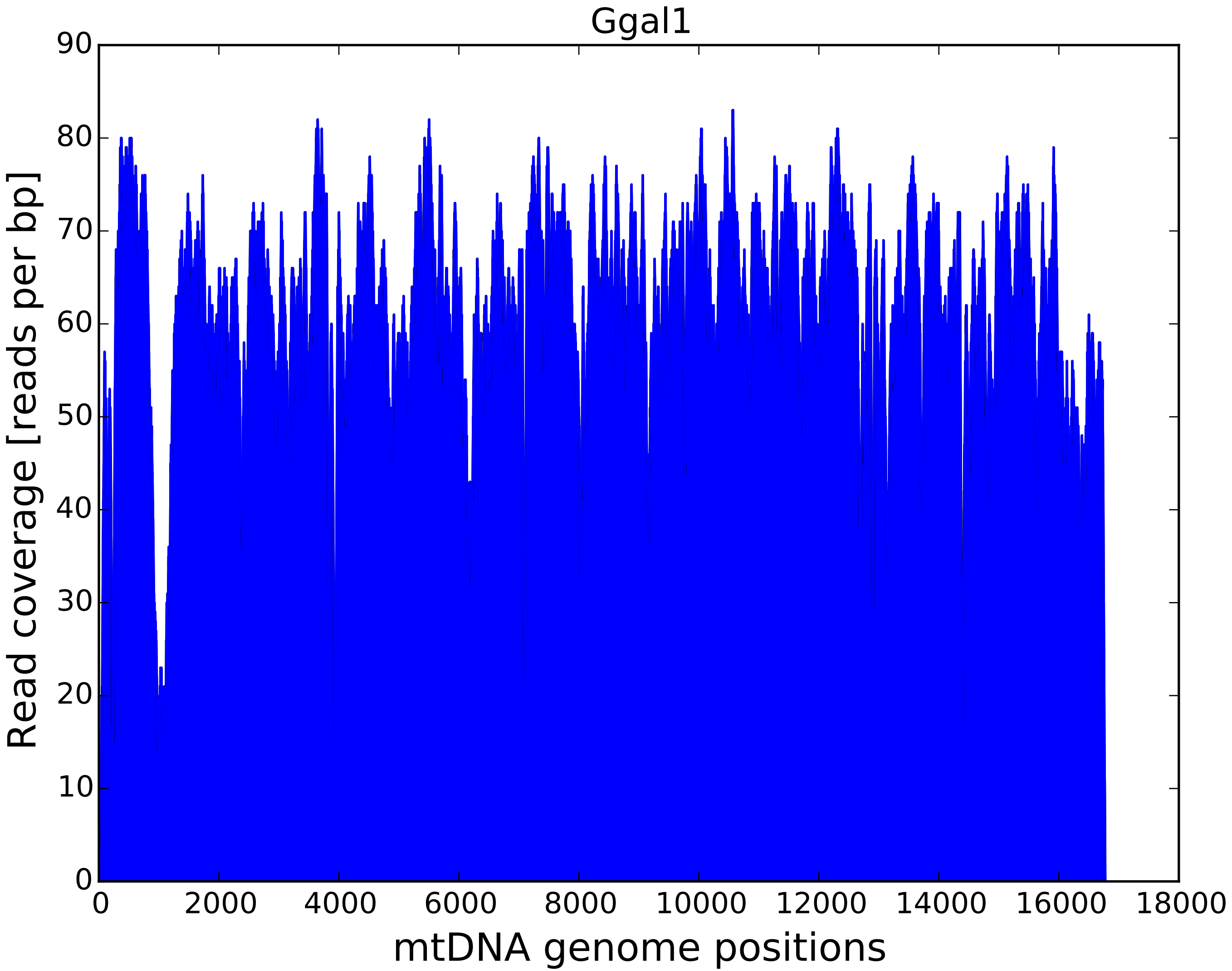
Ggal1
Read coverage [reads per bp]
mtDNA genome positions
0
10
20
30
40
50
60
70
80
90
0
2000
4000
6000
8000
10000
12000
14000
16000
18000

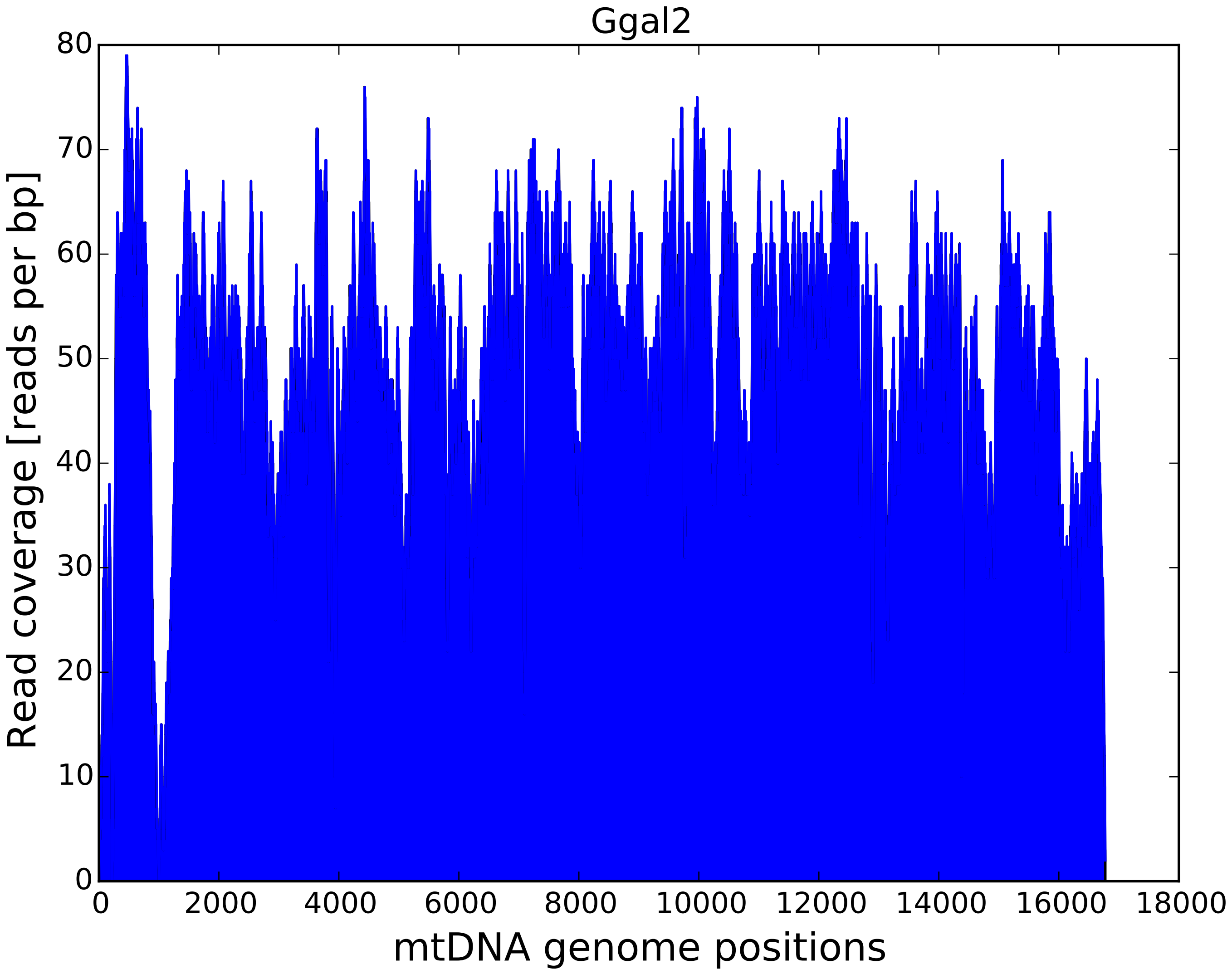

Ggal2
Read coverage [reads per bp]
mtDNA genome positions
0
10
20
30
40
50
60
70
80
0
2000
4000
6000
8000
10000
12000
14000
16000
18000

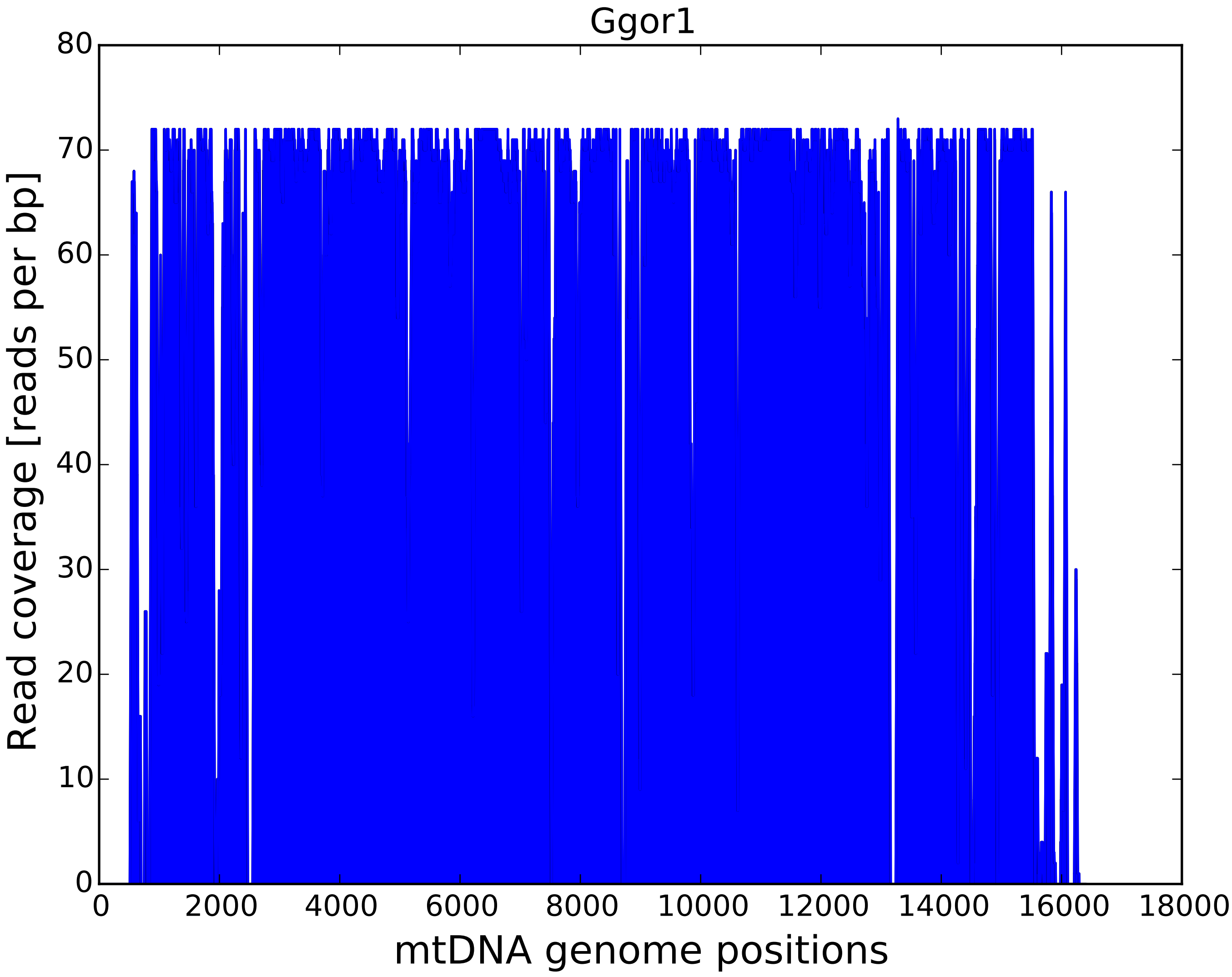
Ggor1
Read coverage [reads per bp]
mtDNA genome positions
0
10
20
30
40
50
60
70
80
0
2000
4000
6000
8000
10000
12000
14000
16000
18000

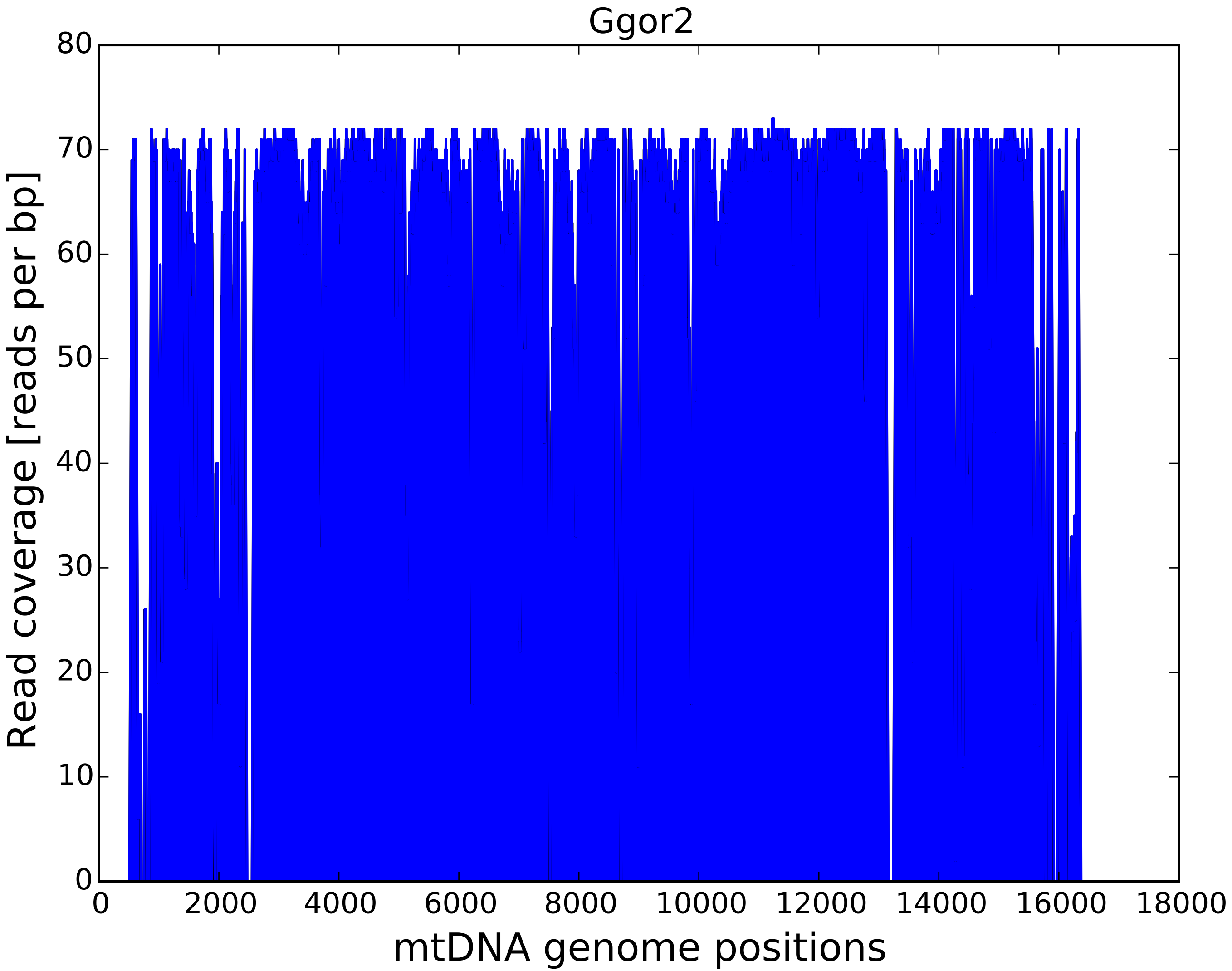

Ggor2
Read coverage [reads per bp]
mtDNA genome positions
0
10
20
30
40
50
60
70
80
0
2000
4000
6000
8000
10000
12000
14000
16000
18000

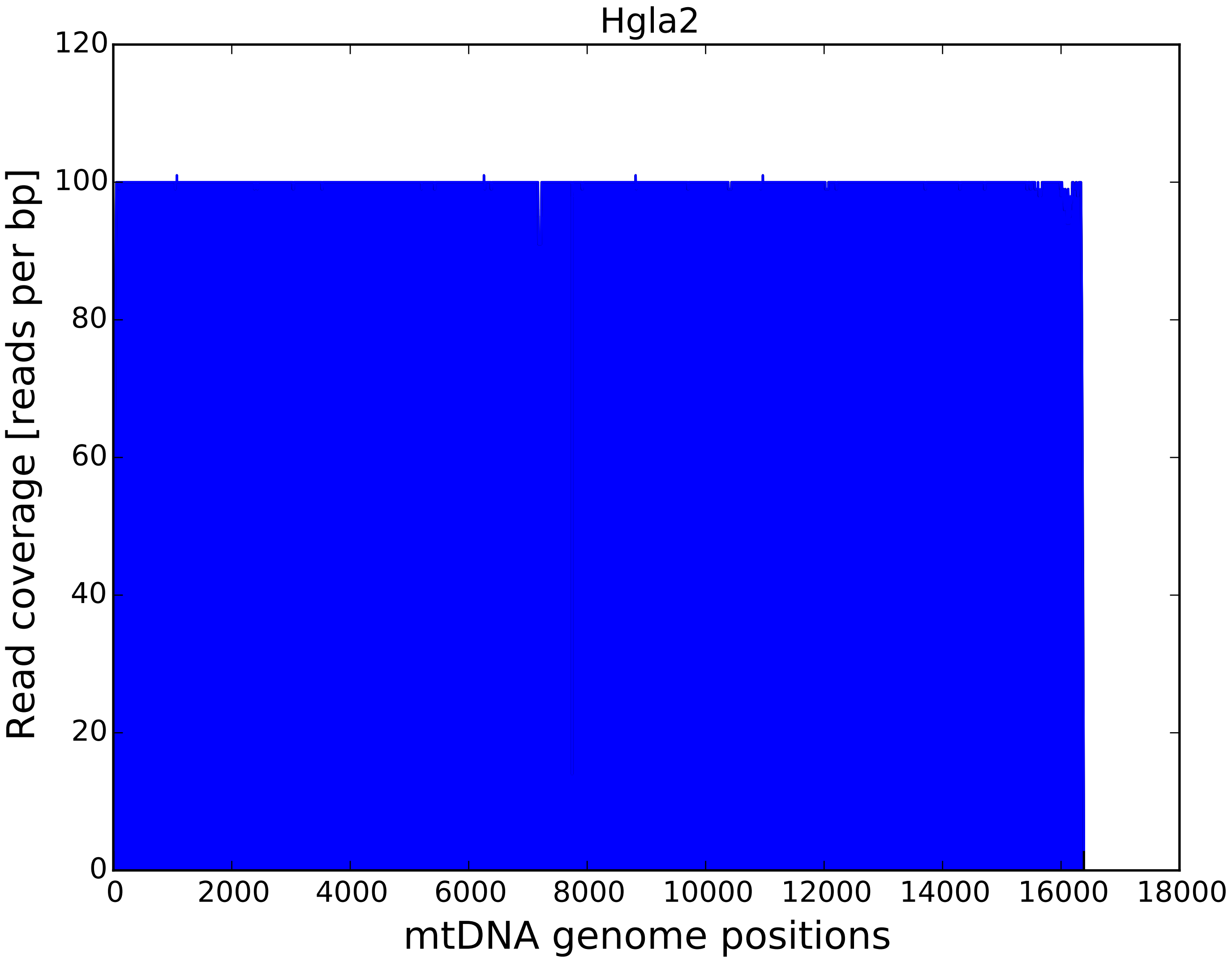

Hgla2
Read coverage [reads per bp]
mtDNA genome positions
0
20
40
60
80
100
120
0
2000
4000
6000
8000
10000
12000
14000
16000
18000

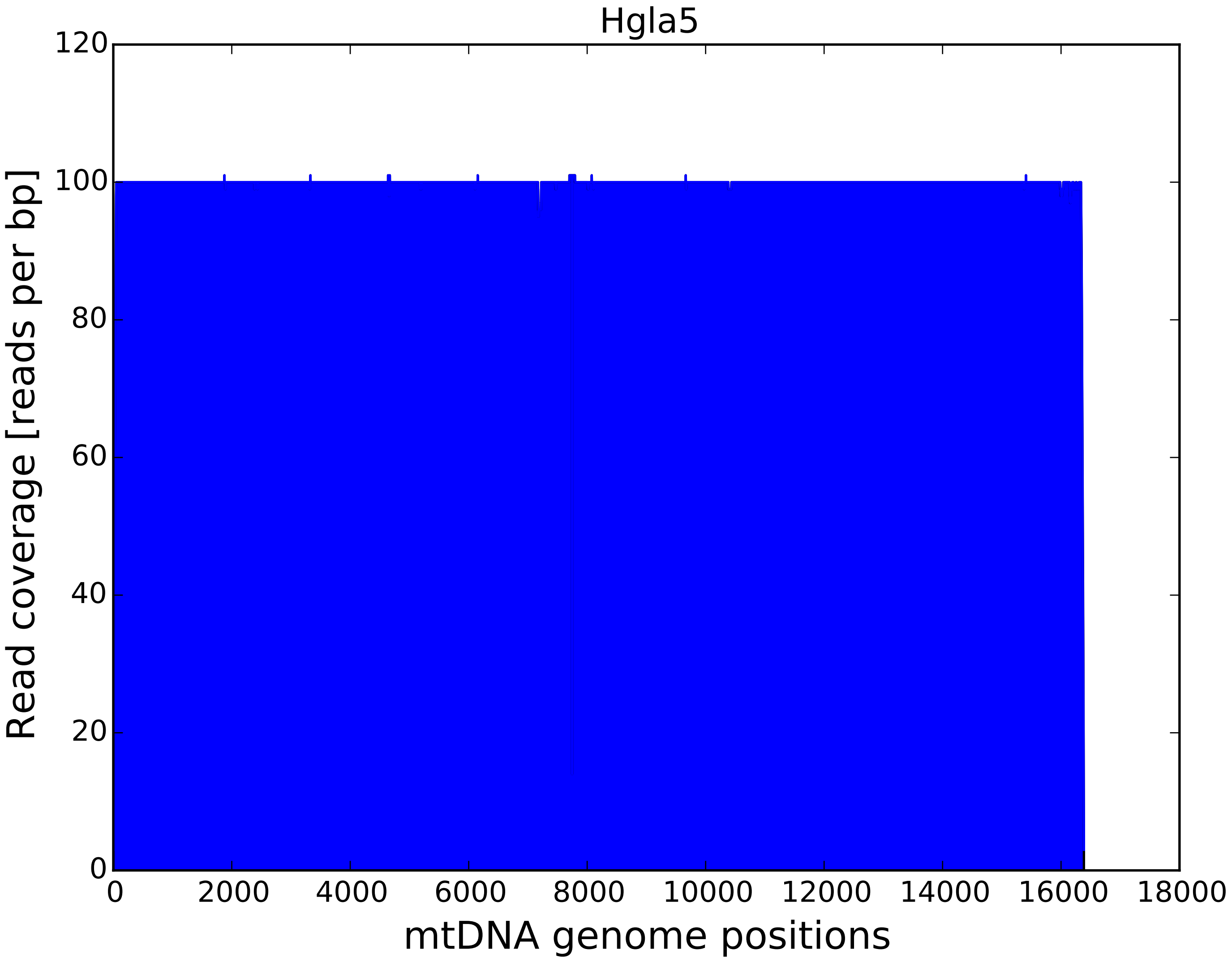

Hgla5
Read coverage [reads per bp]
0
20
40
60
80
100
120
0
2000
4000
6000
8000
10000
12000
14000
16000
18000
mtDNA genome positions

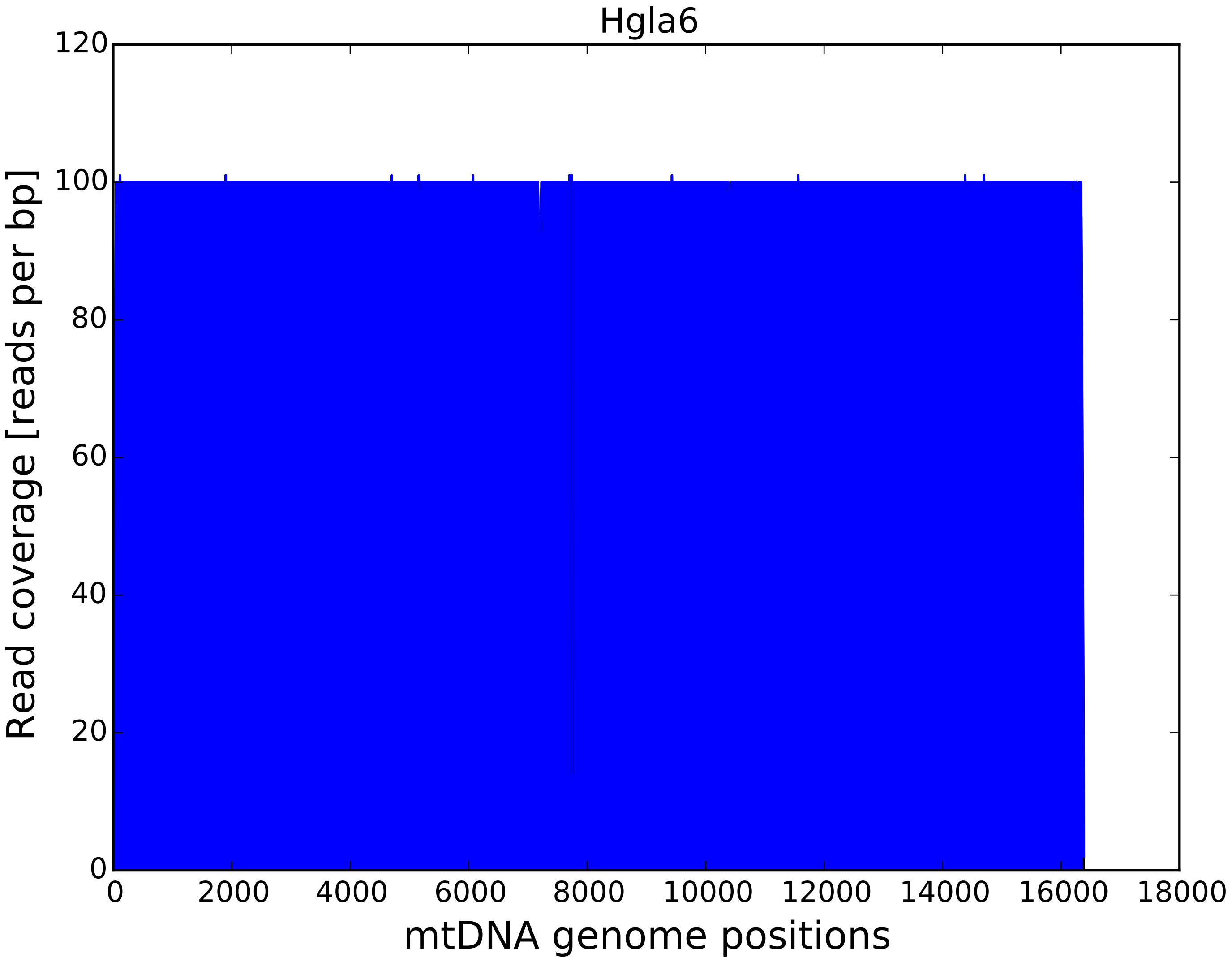
Hgla6
Read coverage [reads per bp]
mtDNA genome positions
0
20
40
60
80
100
120
0
2000
4000
6000
8000
10000
12000
14000
16000
18000

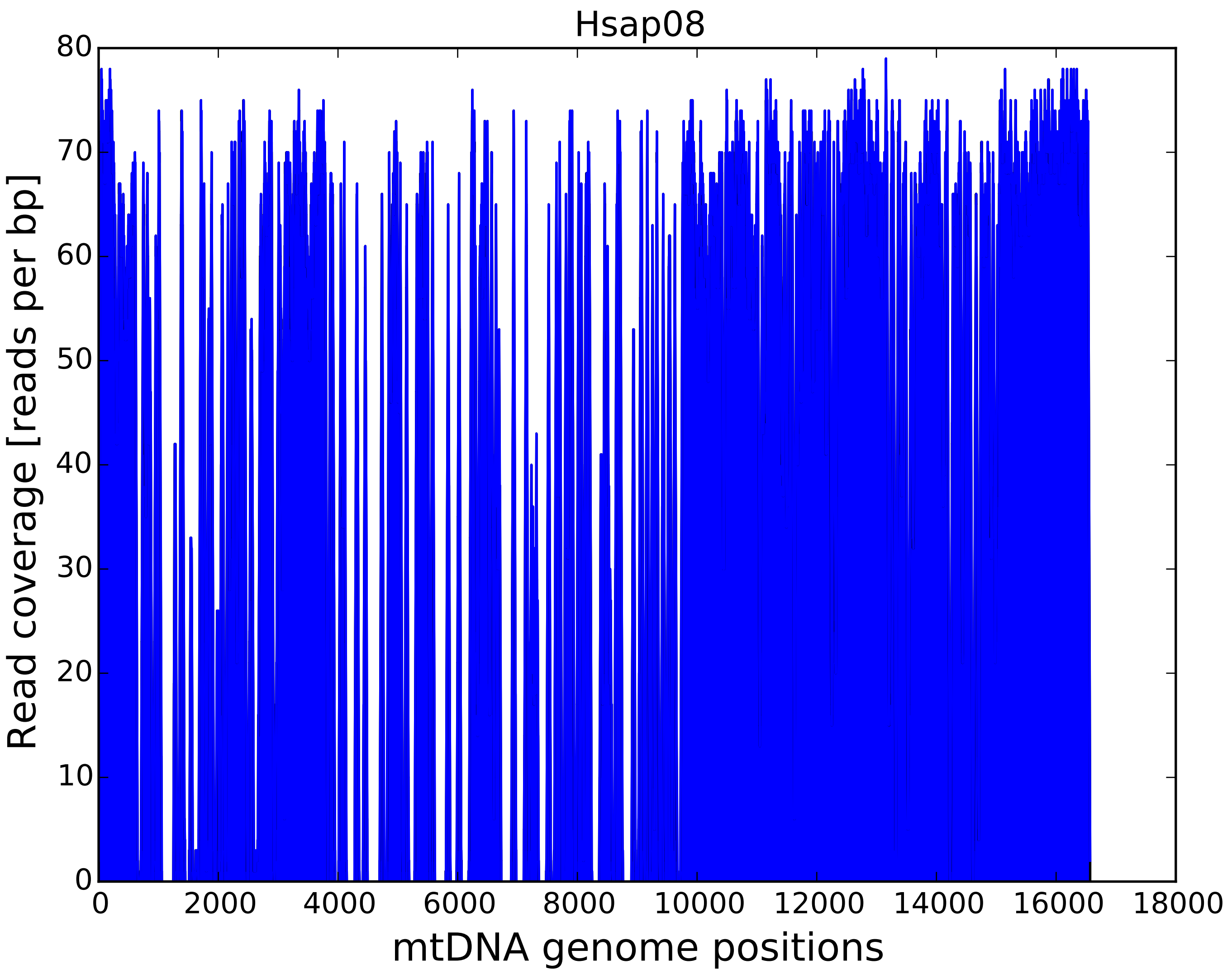
Hsap08
Read coverage [reads per bp]
mtDNA genome positions
0
10
20
30
40
50
60
70
80
0
2000
4000
6000
8000
10000
12000
14000
16000
18000

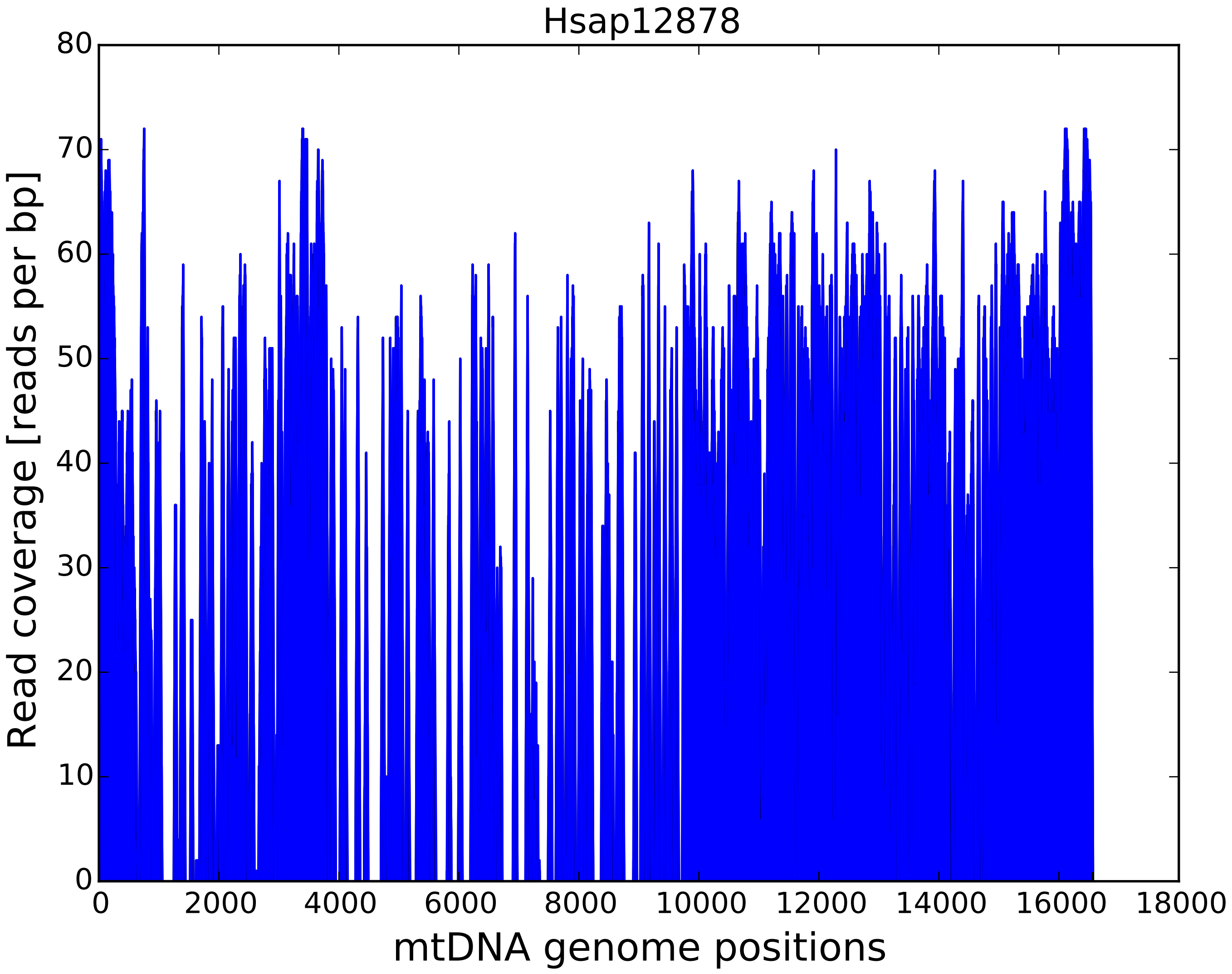

Hsap12878
Read coverage [reads per bp]
mtDNA genome positions
0
10
20
30
40
50
60
70
80
0
2000
4000
6000
8000
10000
12000
14000
16000
18000

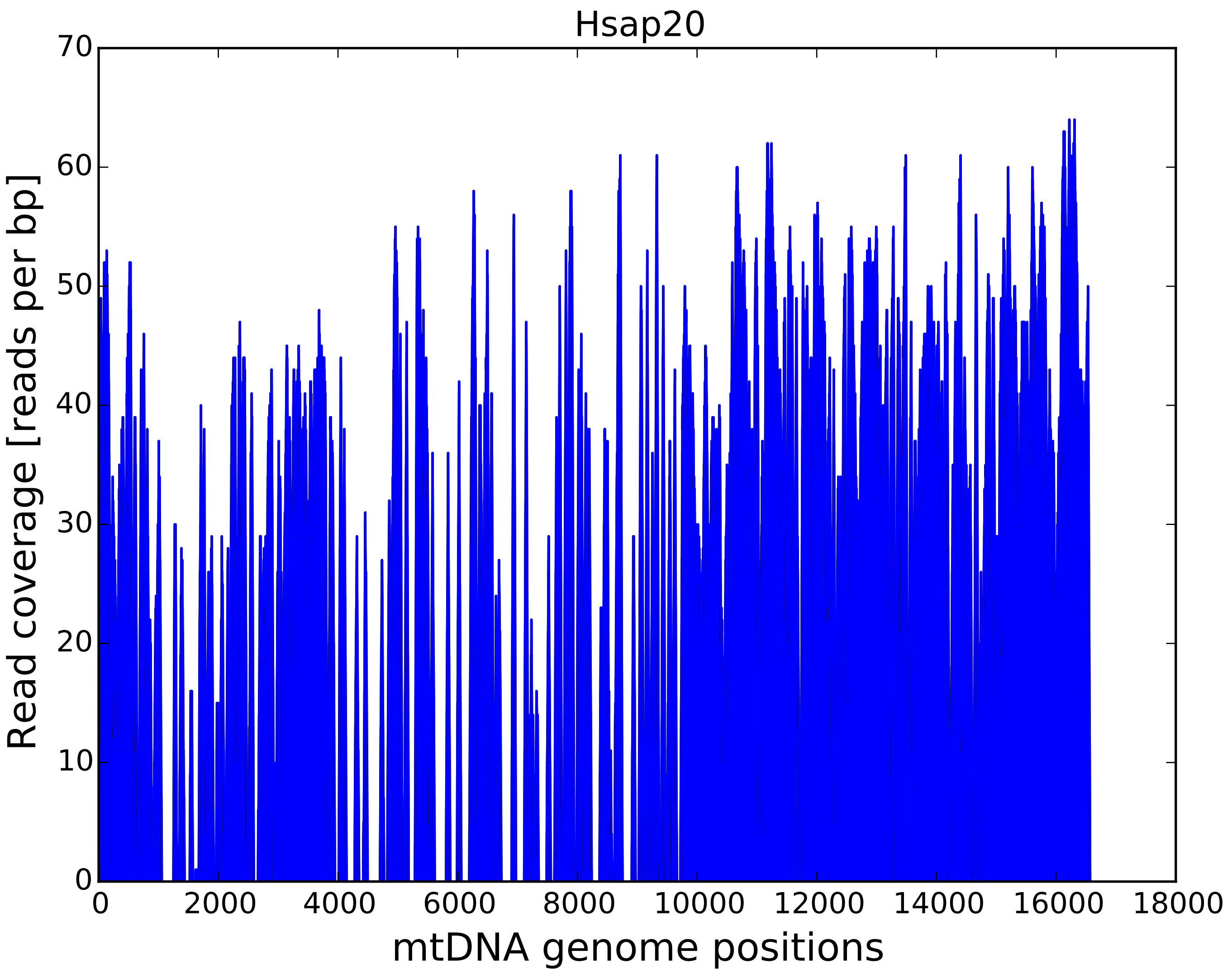

Hsap20
Read coverage [reads per bp]
mtDNA genome positions
0
10
20
30
40
50
60
70
0
2000
4000
6000
8000
10000
12000
14000
16000
18000

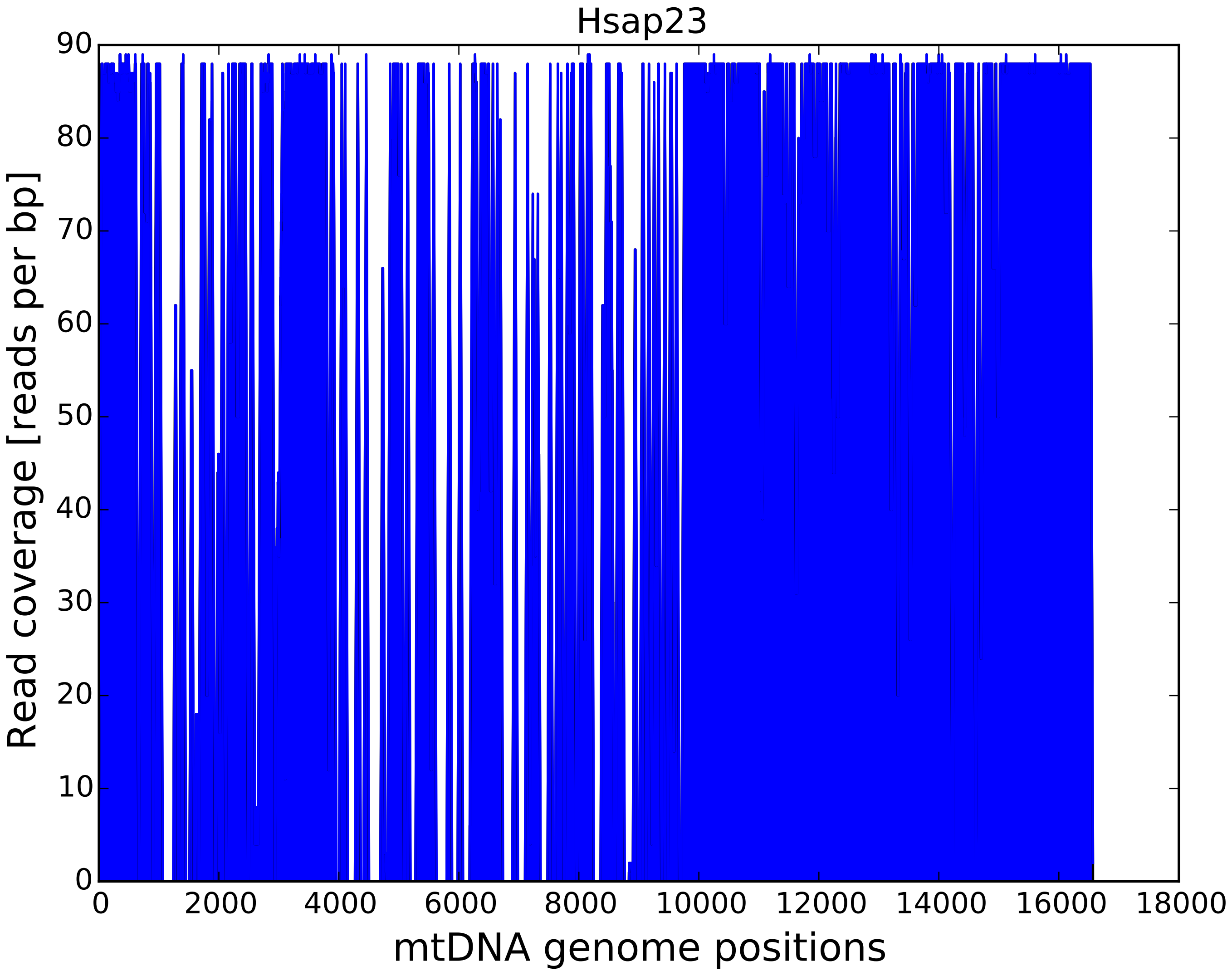

Hsap23
Read coverage [reads per bp]
mtDNA genome positions
0
10
20
30
40
50
60
70
80
90
0
2000
4000
6000
8000
10000
12000
14000
16000
18000

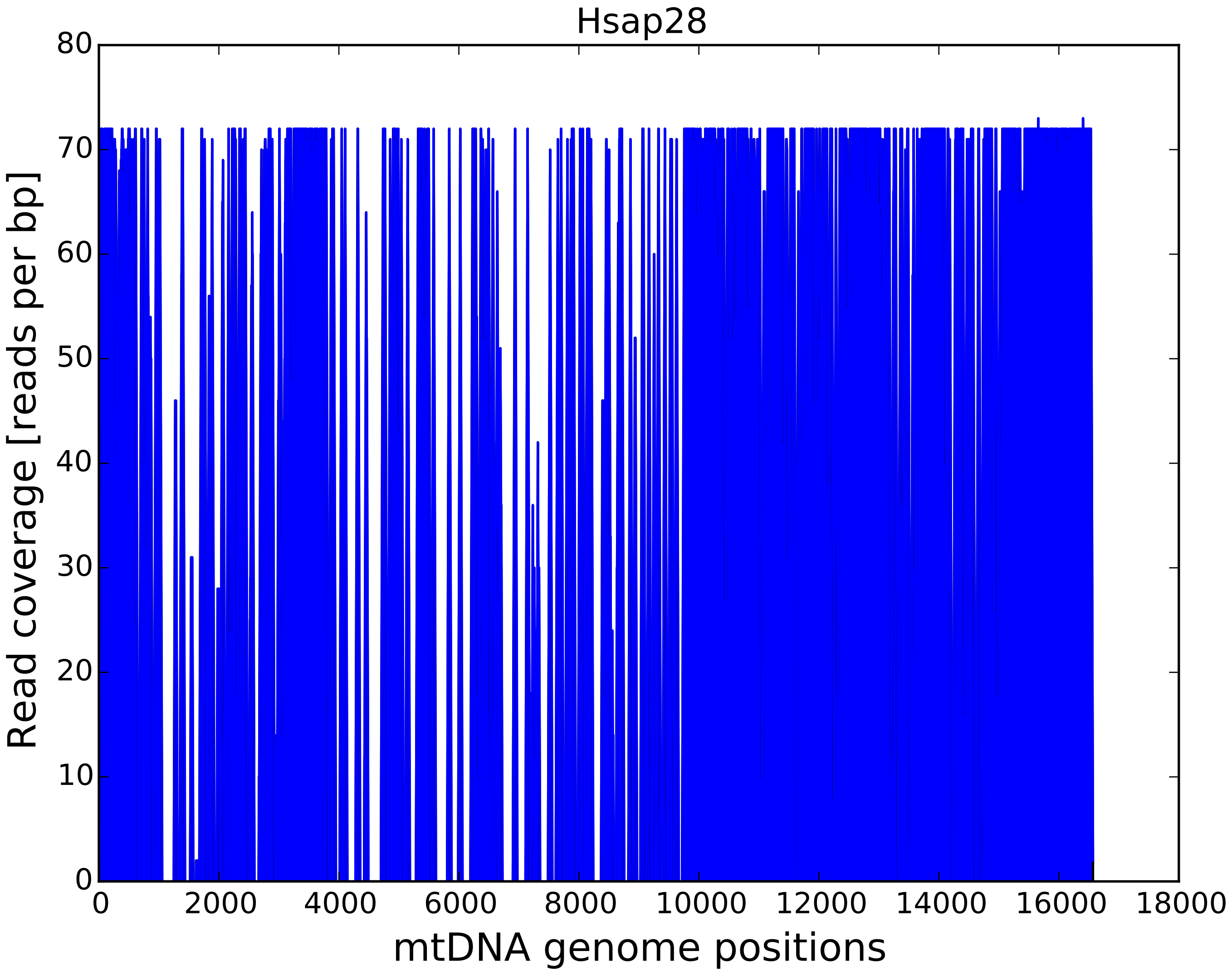

Hsap28
Read coverage [reads per bp]
mtDNA genome positions
0
10
20
30
40
50
60
70
80
0
2000
4000
6000
8000
10000
12000
14000
16000
18000

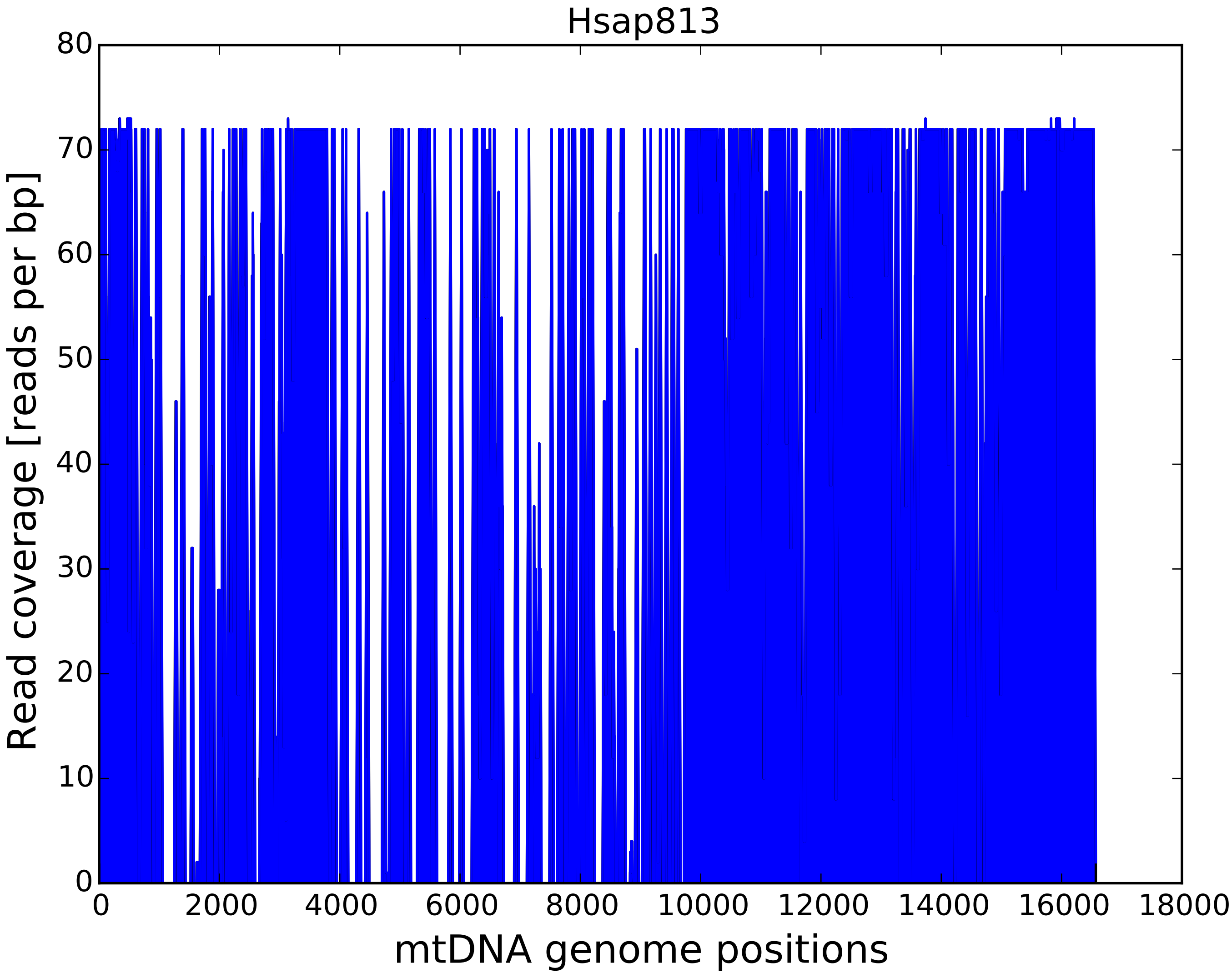

Hsap813
Read coverage [reads per bp]
mtDNA genome positions
0
10
20
30
40
50
60
70
80
0
2000
4000
6000
8000
10000
12000
14000
16000
18000

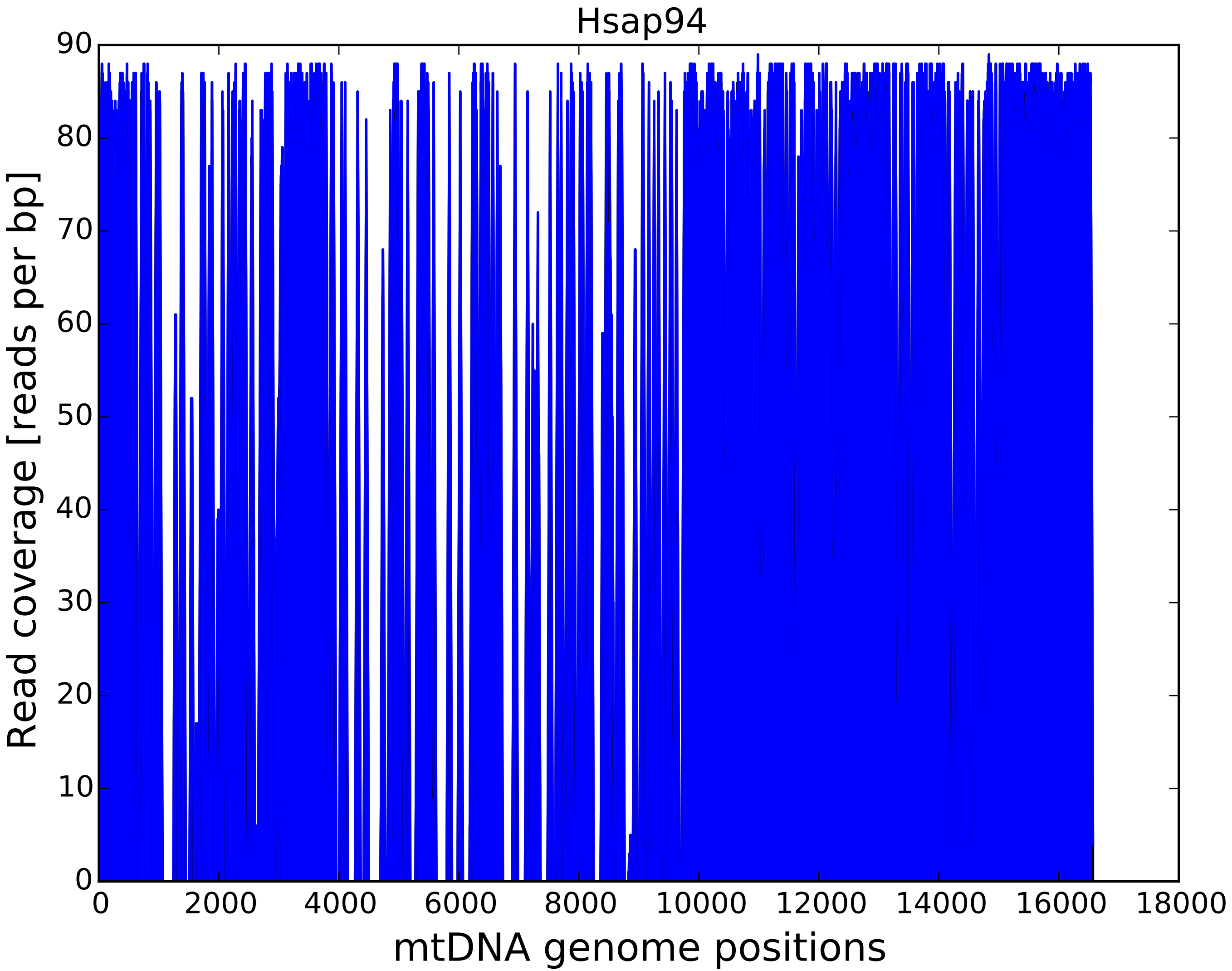

Hsap94
Read coverage [reads per bp]
mtDNA genome positions
0
10
20
30
40
50
60
70
80
90
0
2000
4000
6000
8000
10000
12000
14000
16000
18000

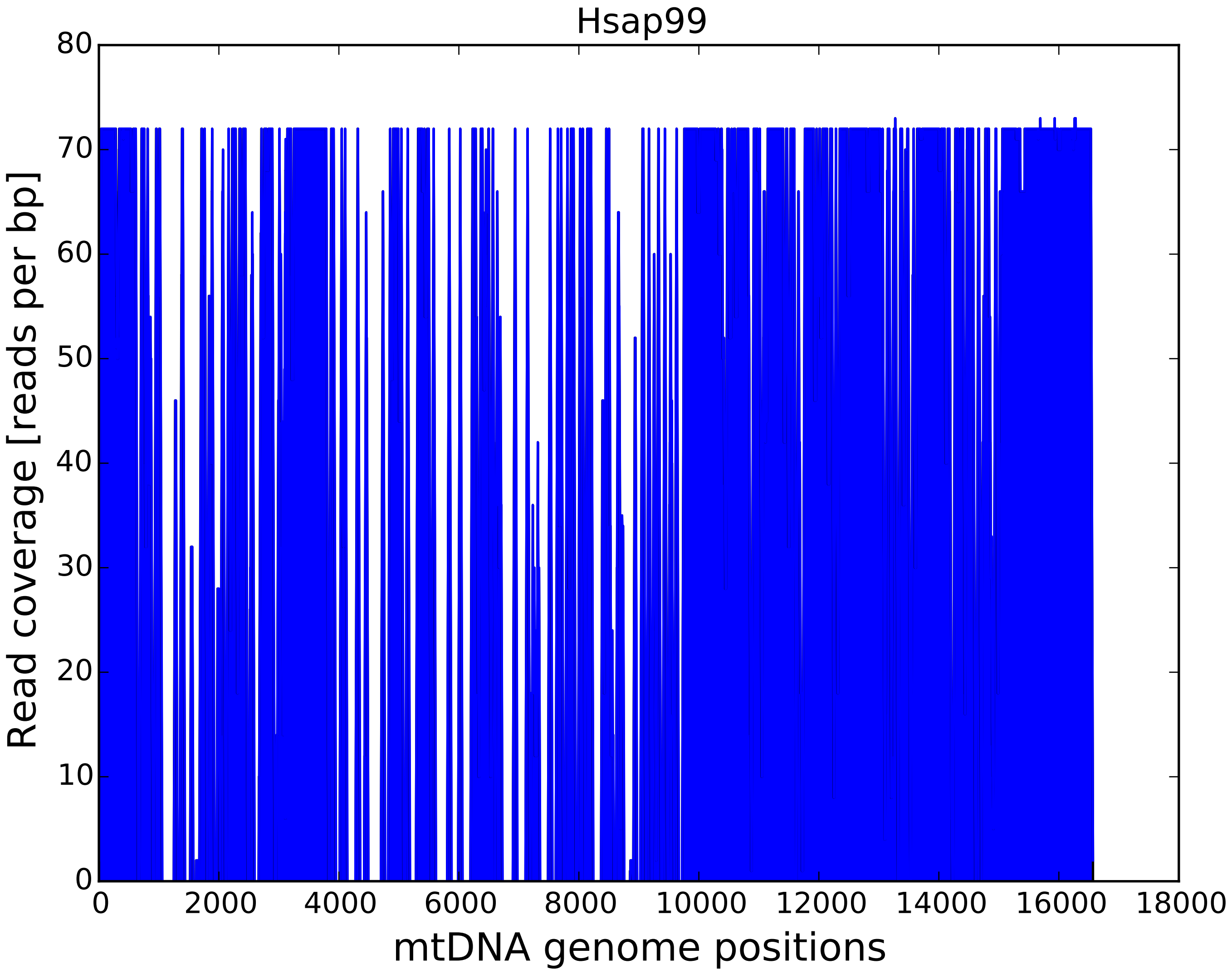
Hsap99
Read coverage [reads per bp]
mtDNA genome positions
0
10
20
30
40
50
60
70
80
0
2000
4000
6000
8000
10000
12000
14000
16000
18000

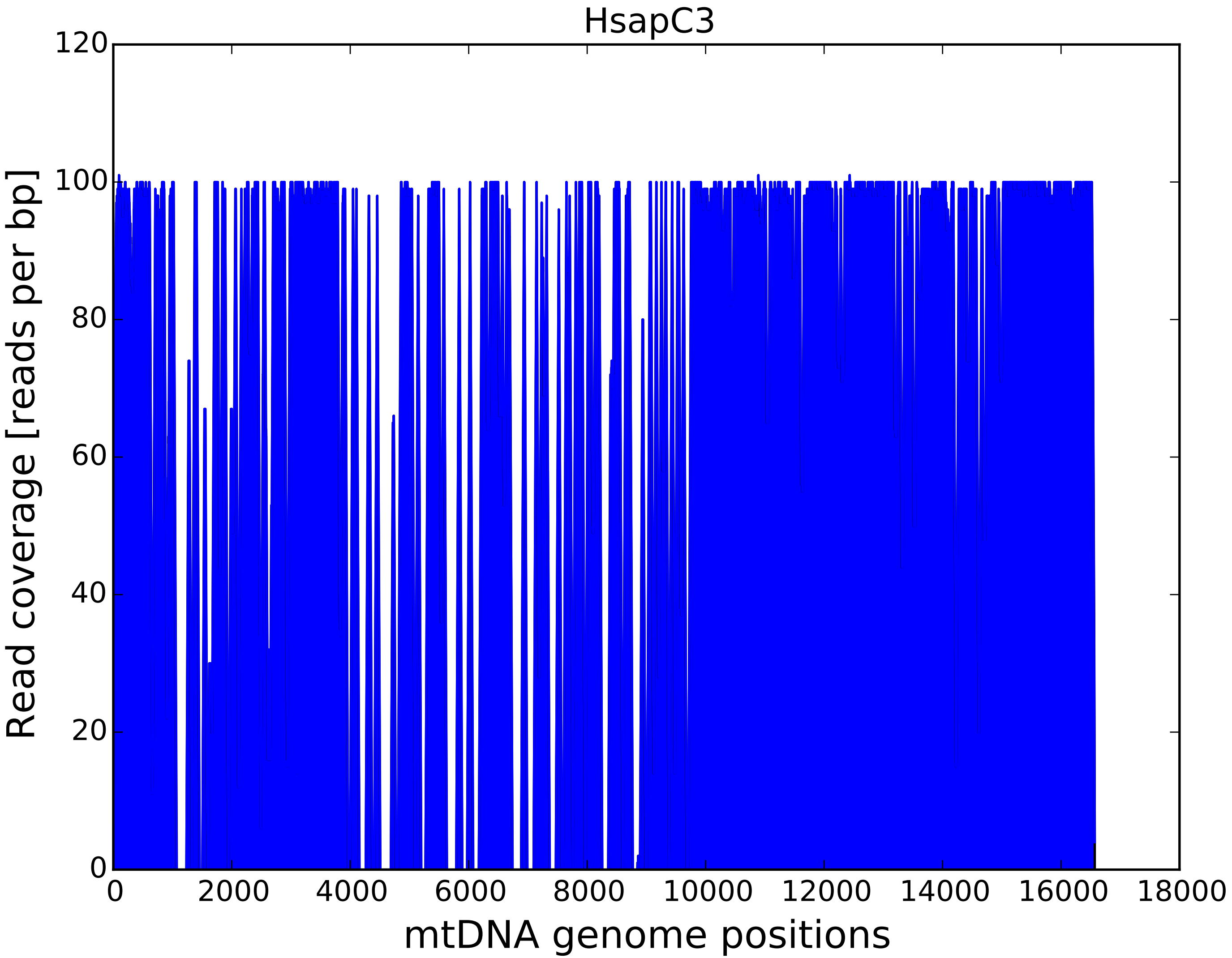

HsapC3
Read coverage [reads per bp]
mtDNA genome positions
0
20
40
60
80
100
120
0
2000
4000
6000
8000
10000
12000
14000
16000
18000

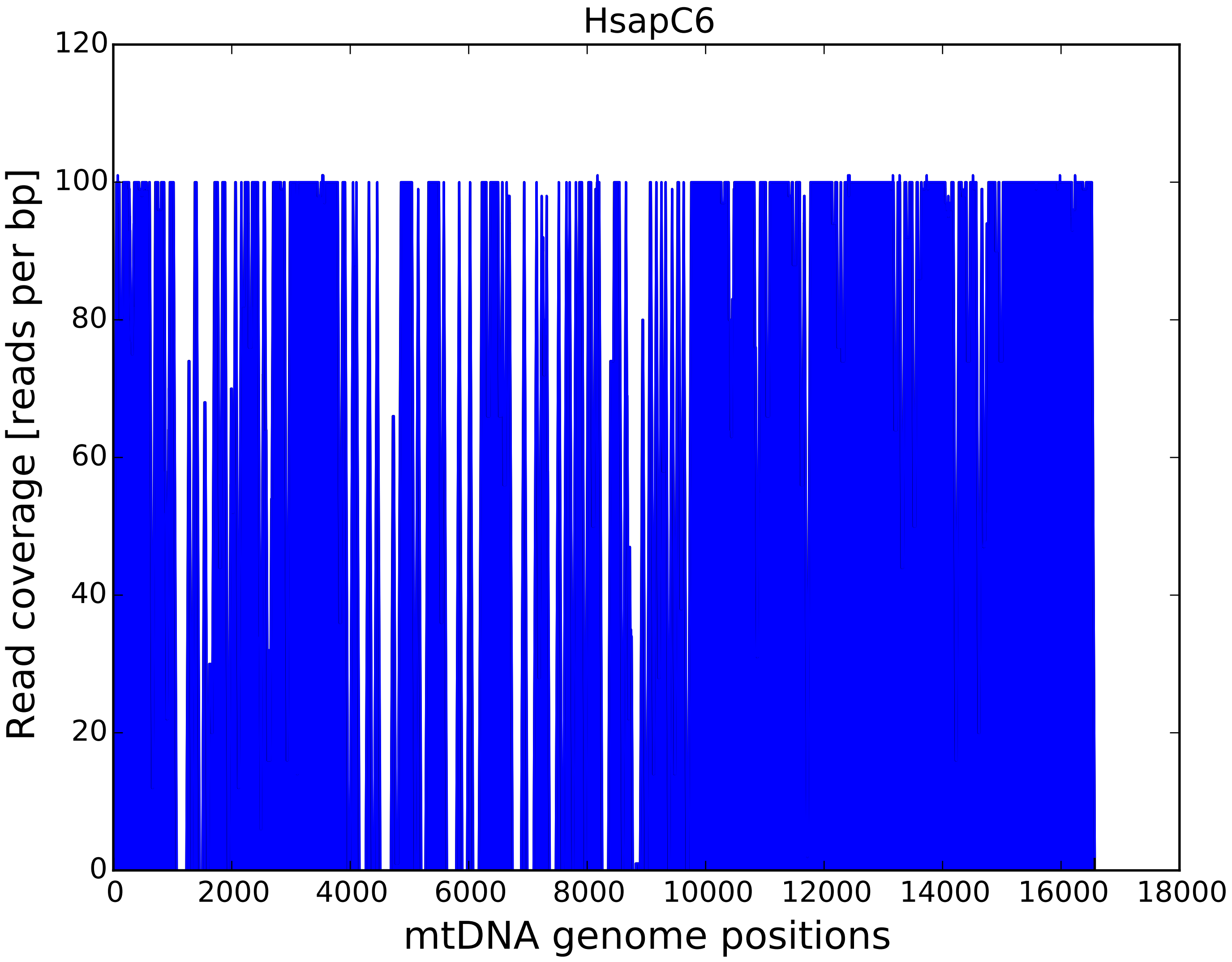

HsapC6
Read coverage [reads per bp]
mtDNA genome positions
0
20
40
60
80
100
120
0
2000
4000
6000
8000
10000
12000
14000
16000
18000

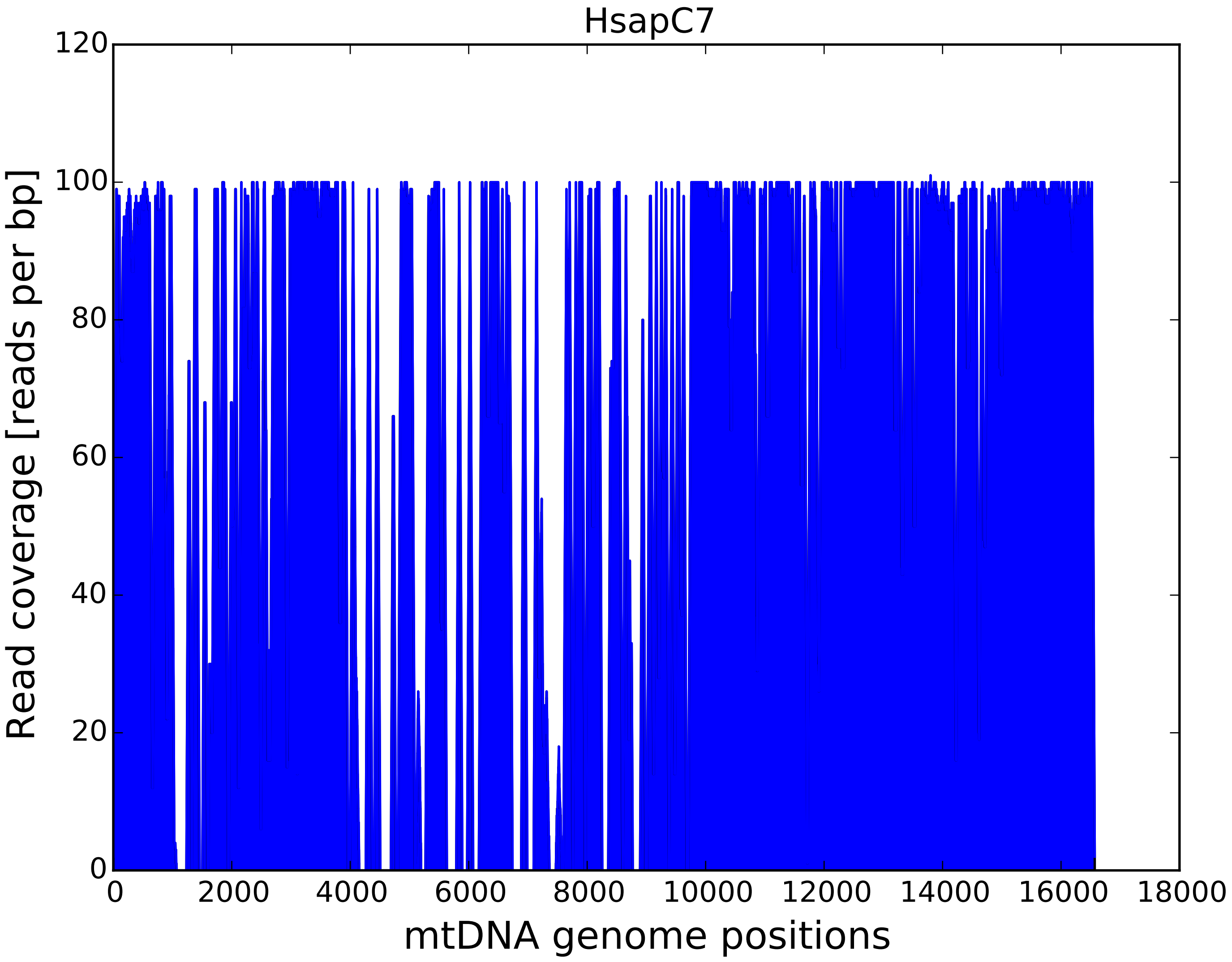

HsapC7
Read coverage [reads per bp]
mtDNA genome positions
0
20
40
60
80
100
120
2000
4000
6000
8000
10000
12000
14000
16000
18000

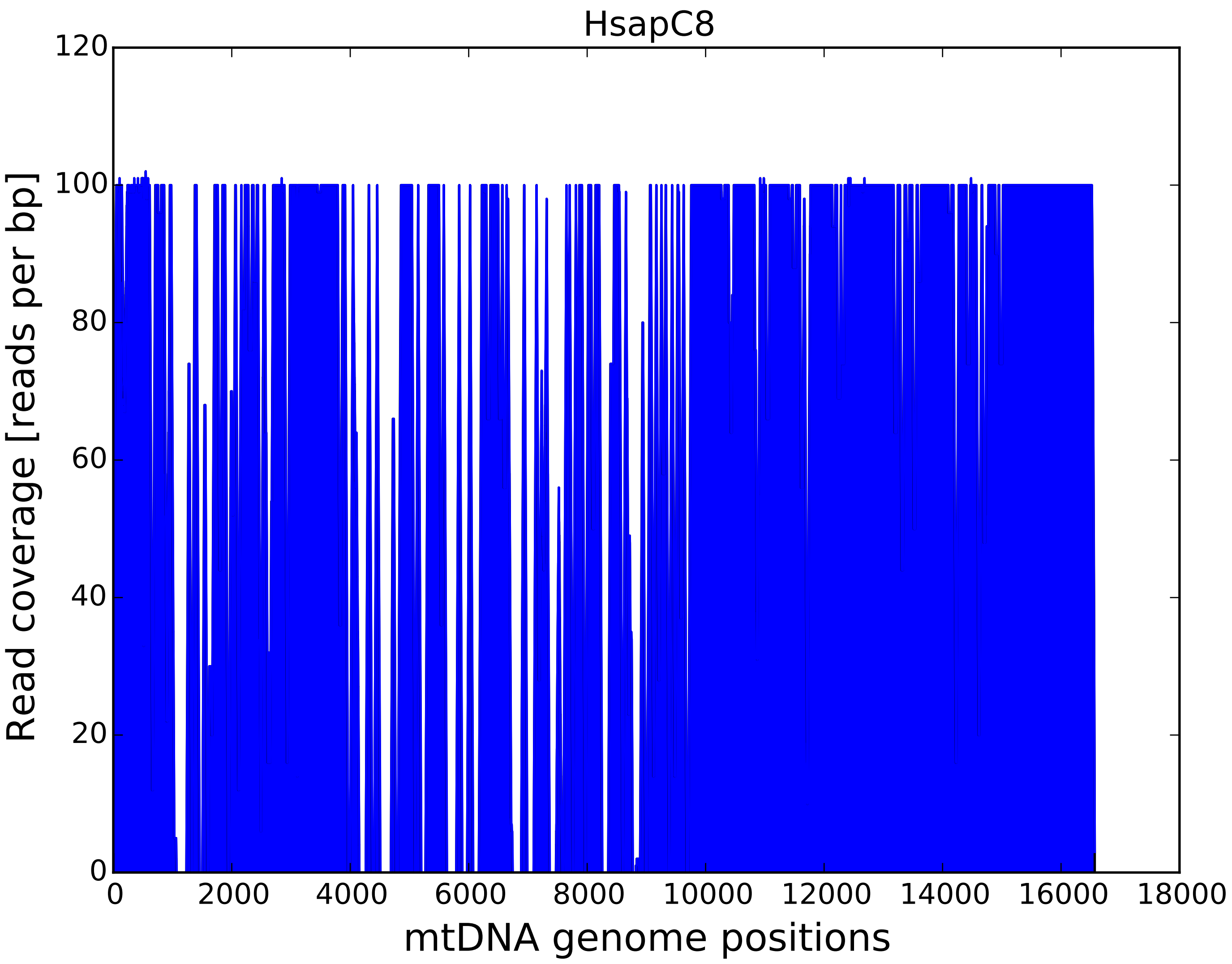

HsapC8
Read coverage [reads per bp]
mtDNA genome positions
0
20
40
60
80
100
120
0
2000
4000
6000
8000
10000
12000
14000
16000
18000

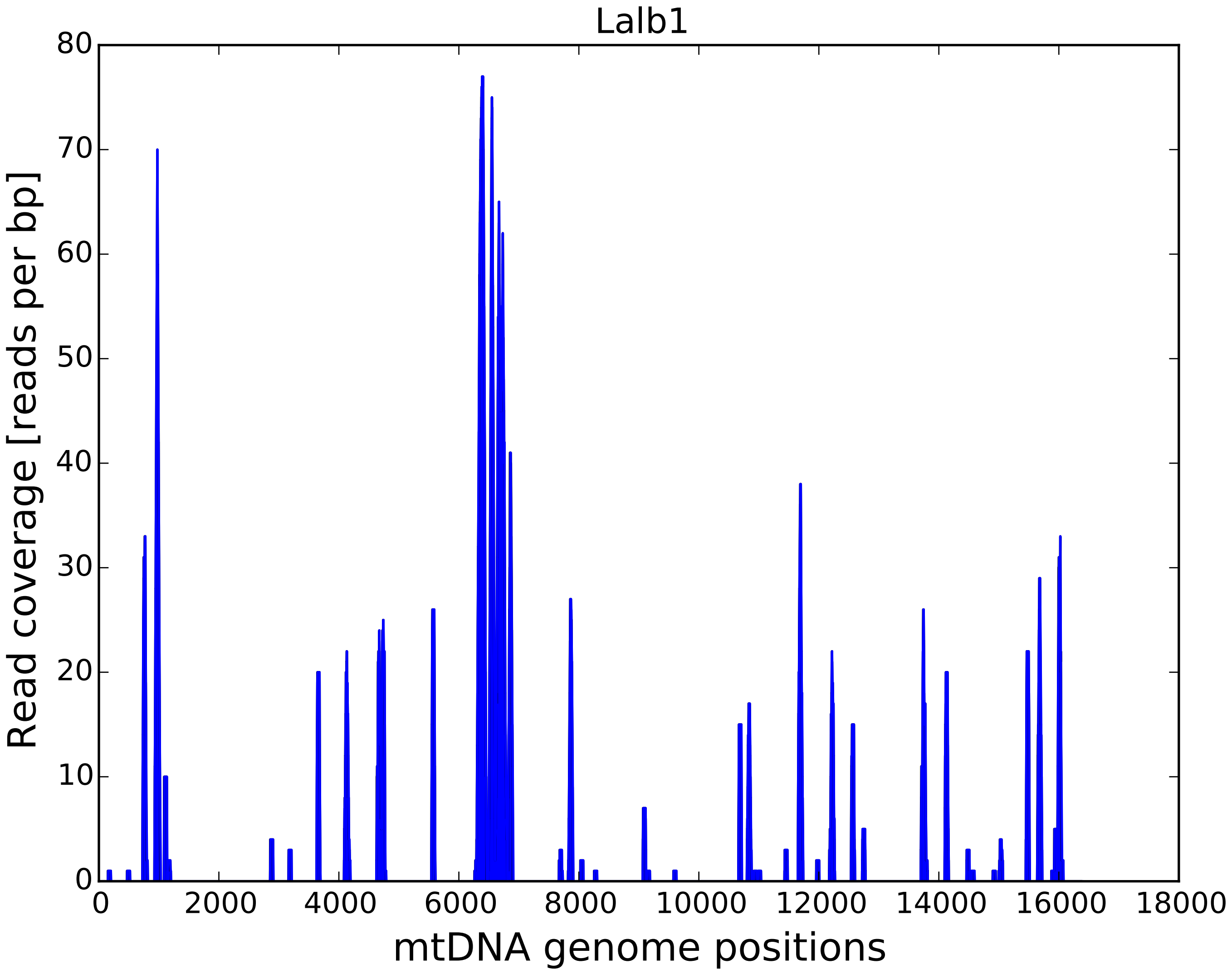
Lalb1
Read coverage [reads per bp]
mtDNA genome positions
0
10
20
30
40
50
60
70
80
0
2000
4000
6000
8000
10000
12000
14000
16000
18000

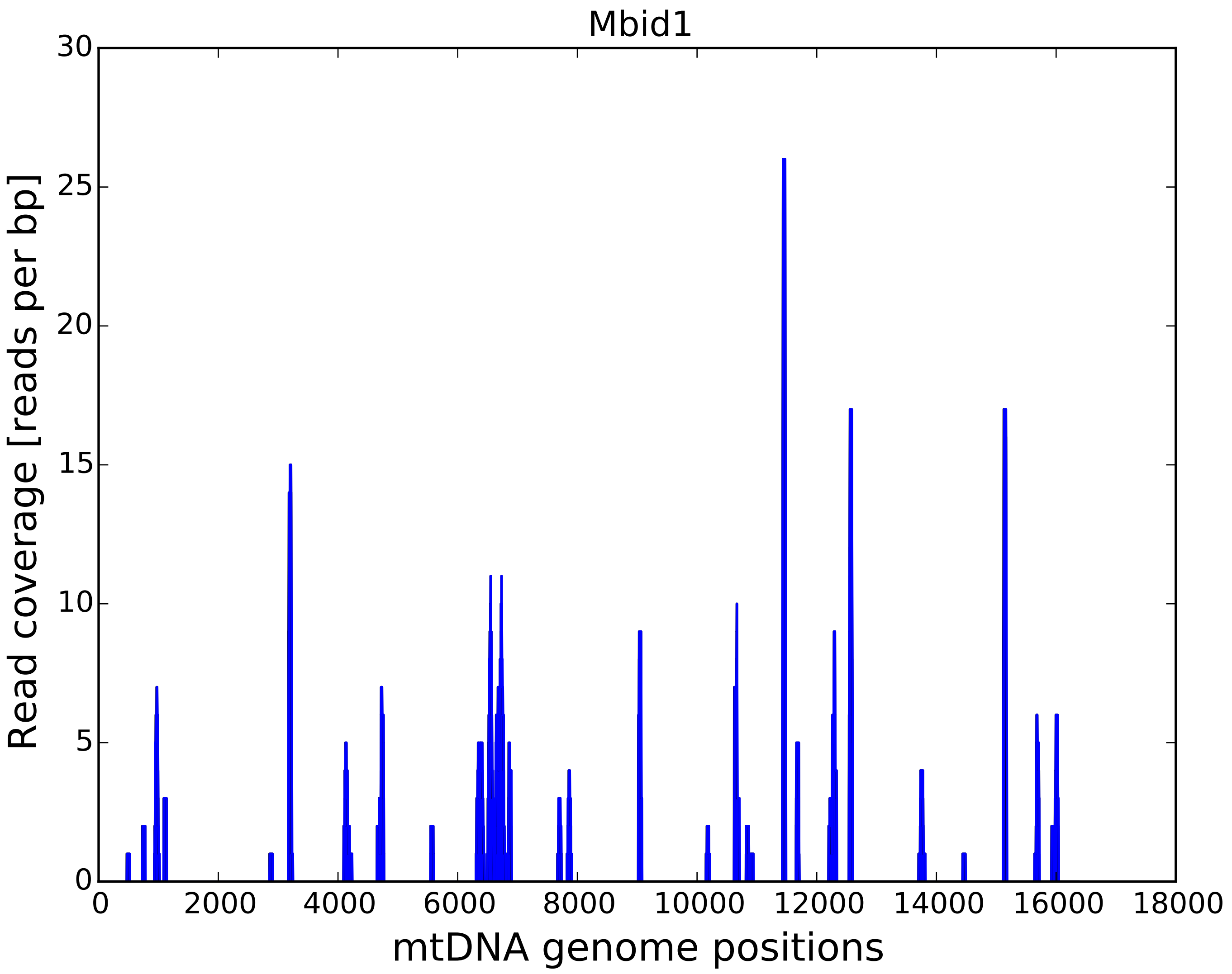

Mbid1
Read coverage [reads per bp]
mtDNA genome positions
0
5
10
15
20
25
30
0
2000
4000
6000
8000
10000
12000
14000
16000
18000

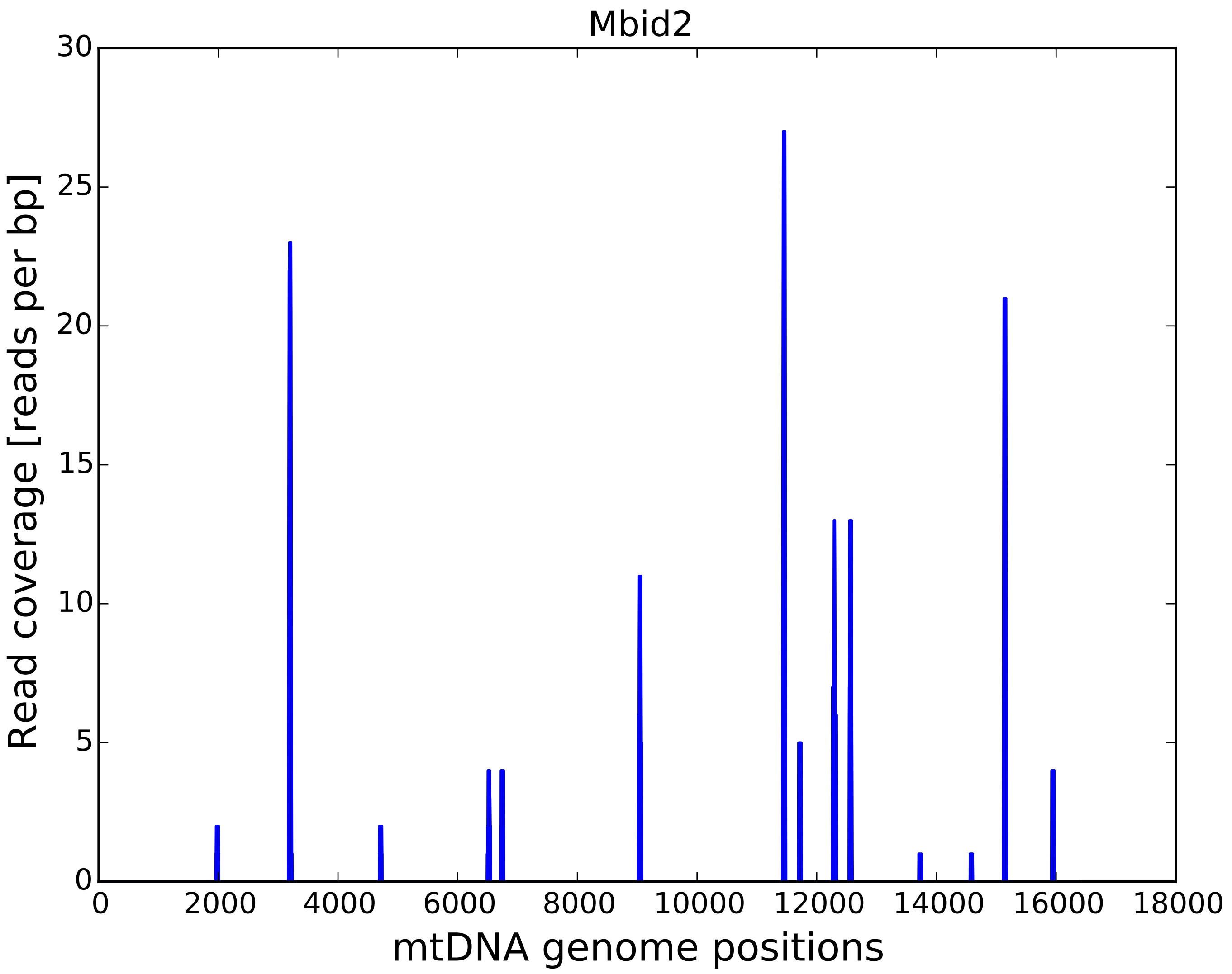

Mbid2
Read coverage [reads per bp]
mtDNA genome positions
0
5
10
15
20
25
30
0
2000
4000
6000
8000
10000
12000
14000
16000
18000

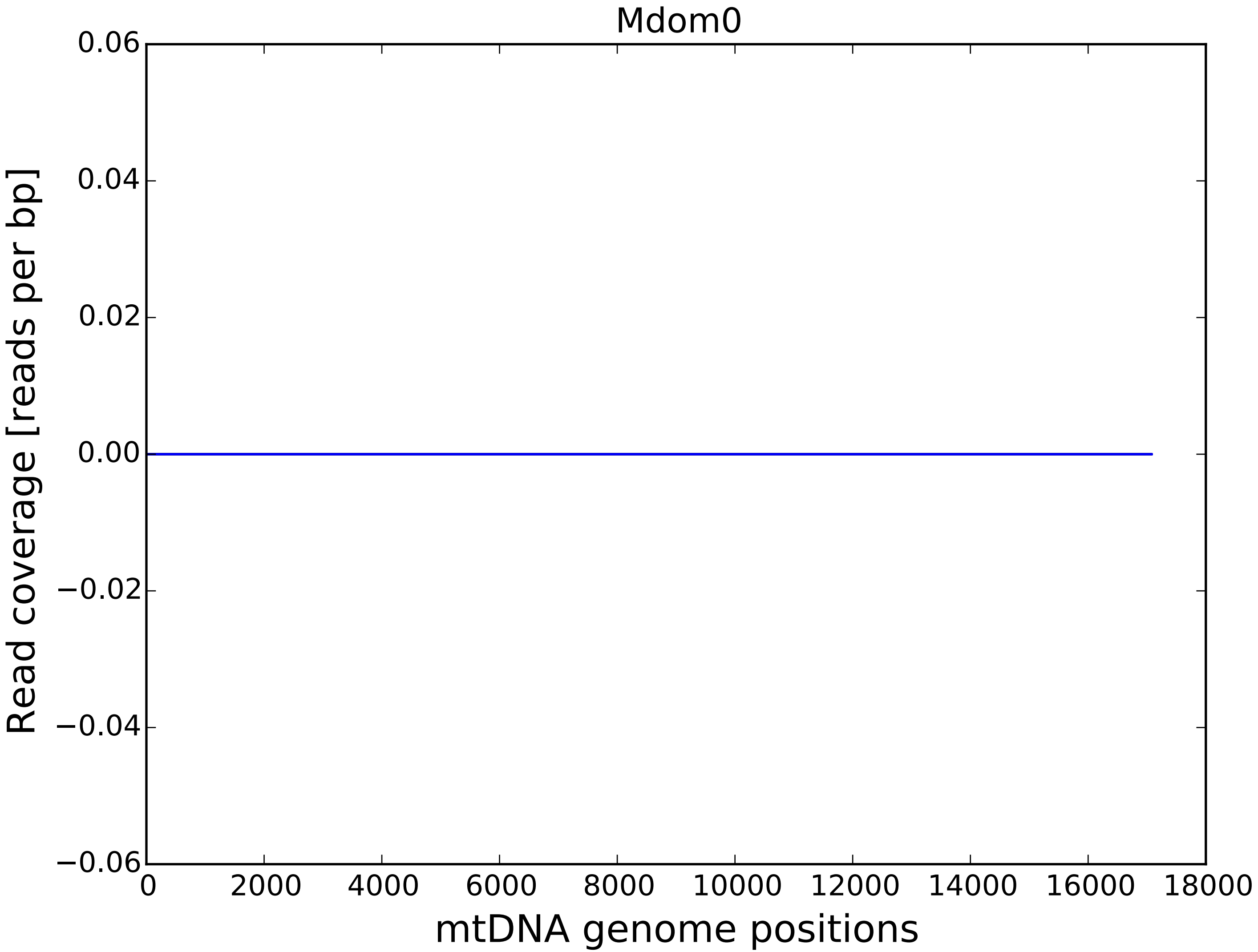
Mdom0
Read coverage [reads per bp]
mtDNA genome positions
0.06
0.04
0.02
0.00
−0.02
−0.04
−0.06
0
2000
4000
6000
8000
10000
12000
14000
16000
18000

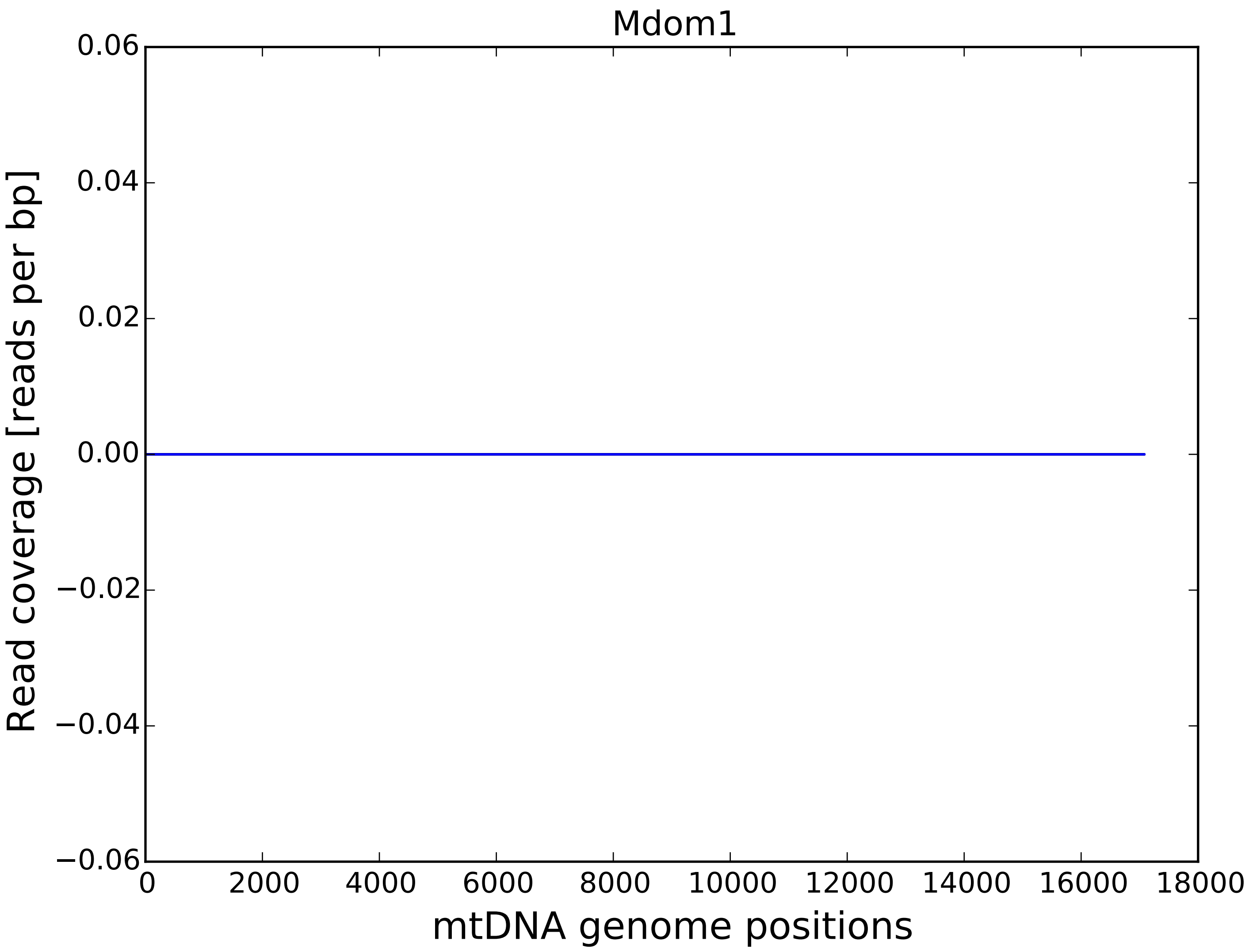
Mdom1
Read coverage [reads per bp]
0.06
0.04
0.02
0.00
−0.02
−0.04
−0.06
0
2000
4000
6000
8000
10000
12000
14000
16000
18000
mtDNA genome positions

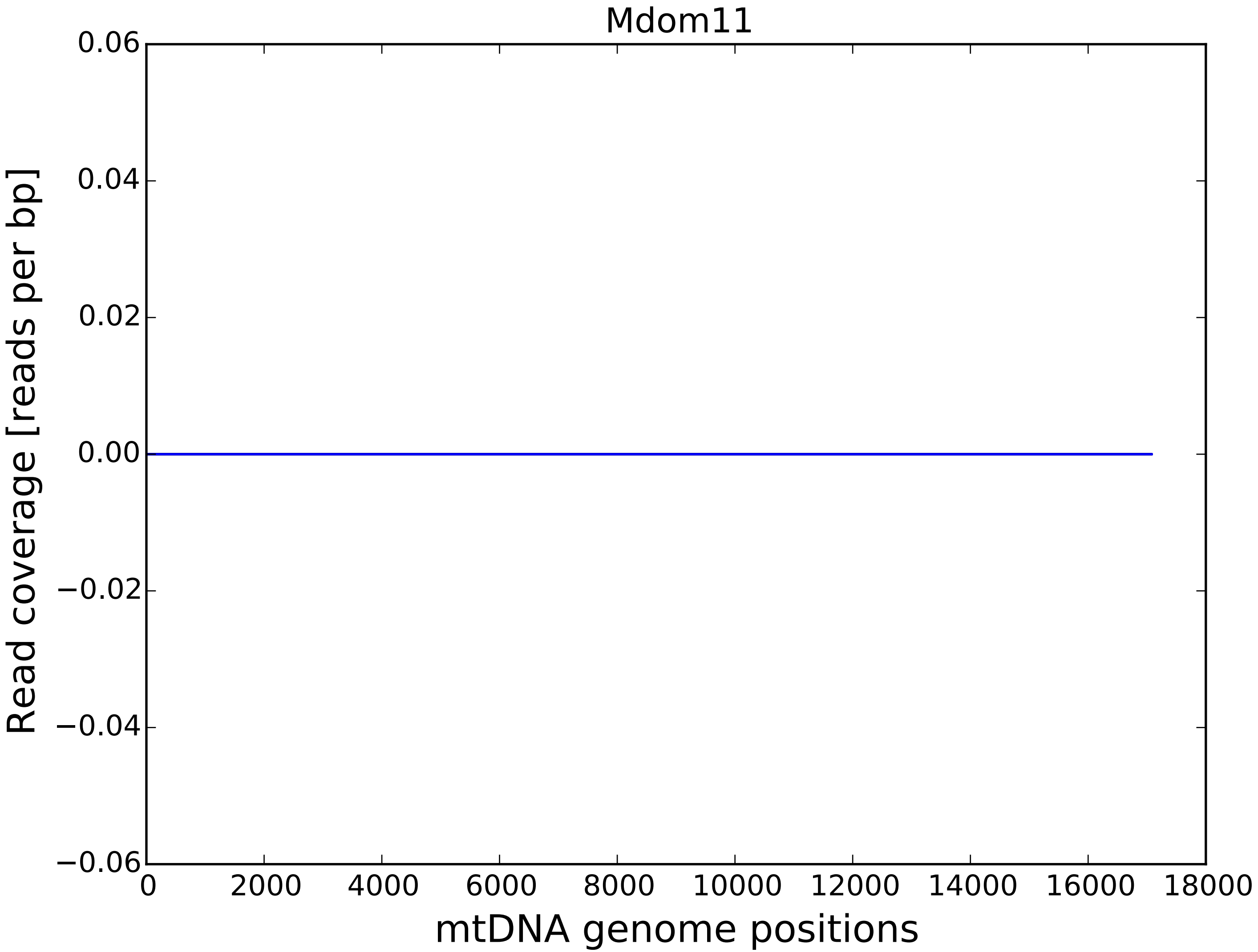

Mdom11
Read coverage [reads per bp]
0.06
0.04
0.02
0.00
−0.02
−0.04
−0.06
0
2000
4000
6000
8000
10000
12000
14000
16000
18000
mtDNA genome positions

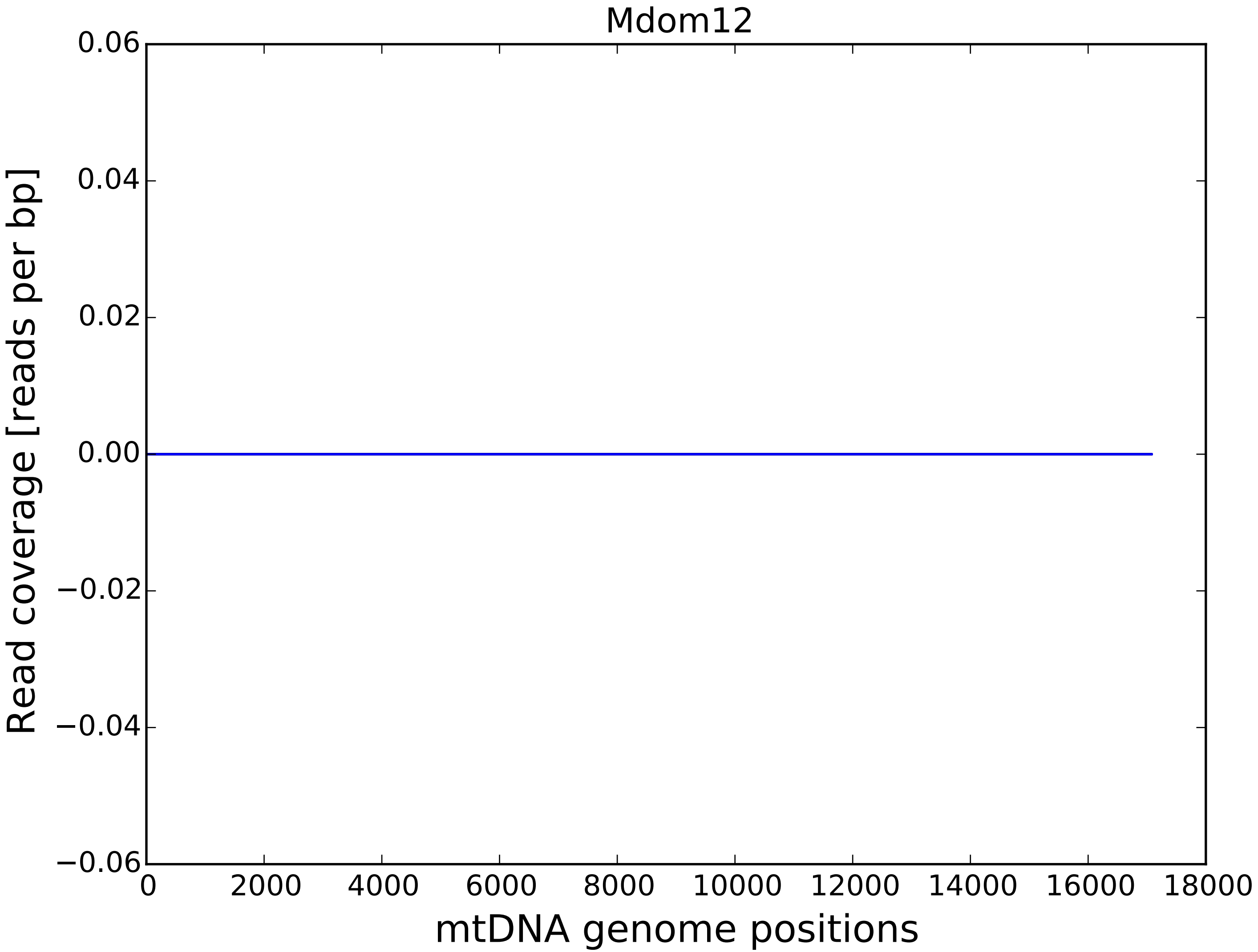

Mdom12
Read coverage [reads per bp]
0.06
0.04
0.02
0.00
−0.02
−0.04
−0.06
0
2000
4000
6000
8000
10000
12000
14000
16000
18000
mtDNA genome positions

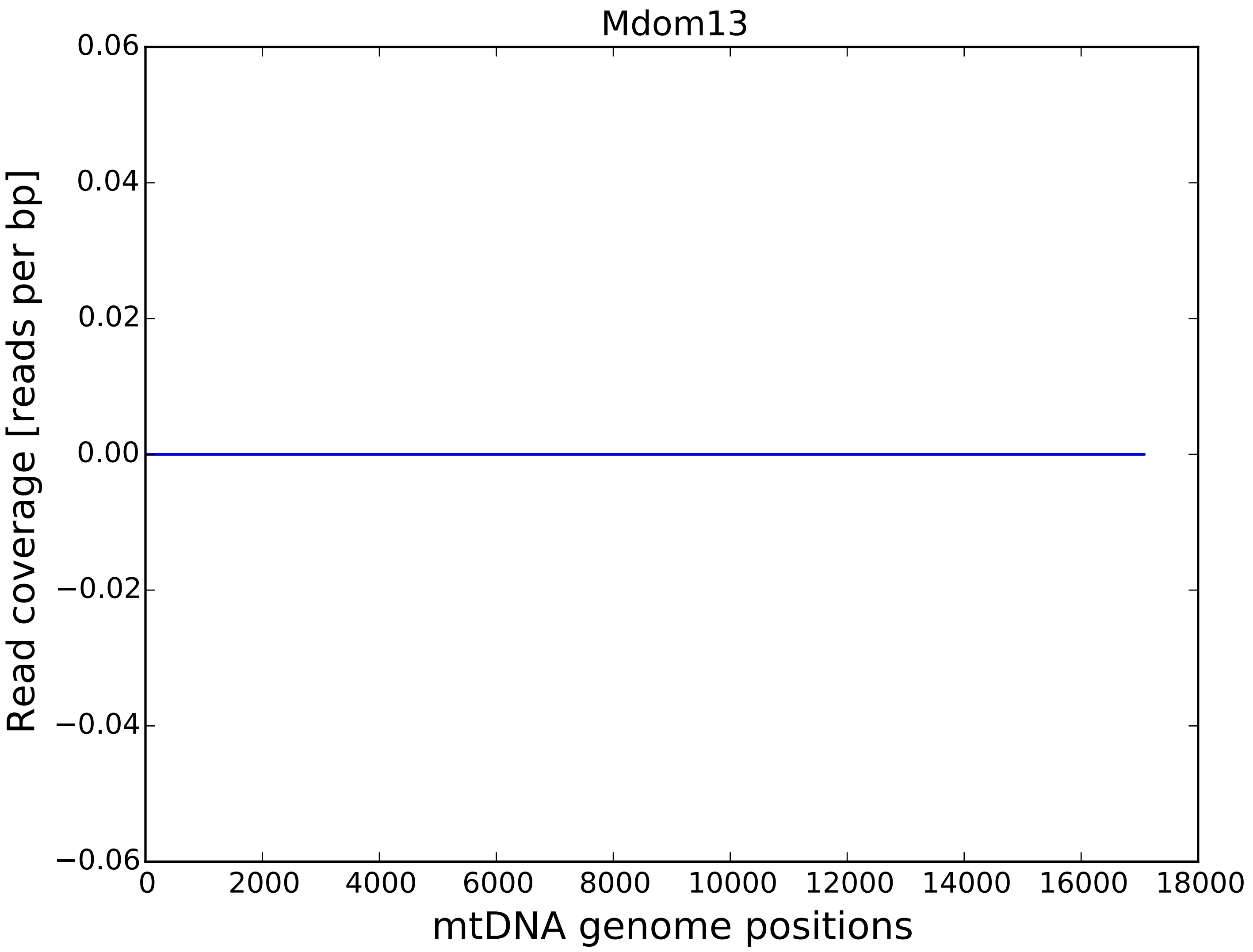
Mdom13
Read coverage [reads per bp]
0.06
0.04
0.02
0.00
−0.02
−0.04
−0.06
0
2000
4000
6000
8000
10000
12000
14000
16000
18000
mtDNA genome positions

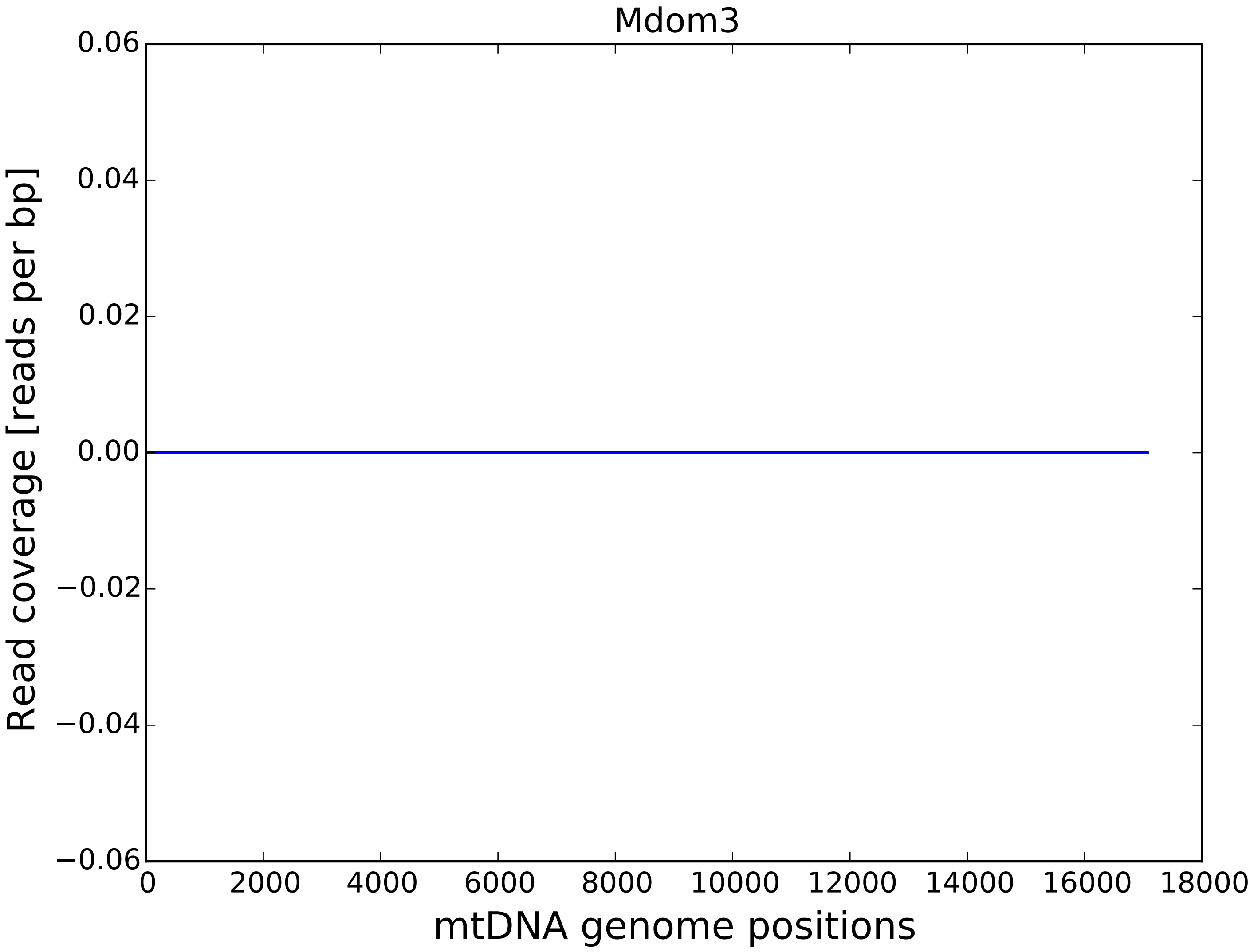
Mdom3
Read coverage [reads per bp]
0.06
0.04
0.02
0.00
−0.02
−0.04
−0.06
0
2000
4000
6000
8000
10000
12000
14000
16000
18000
mtDNA genome positions

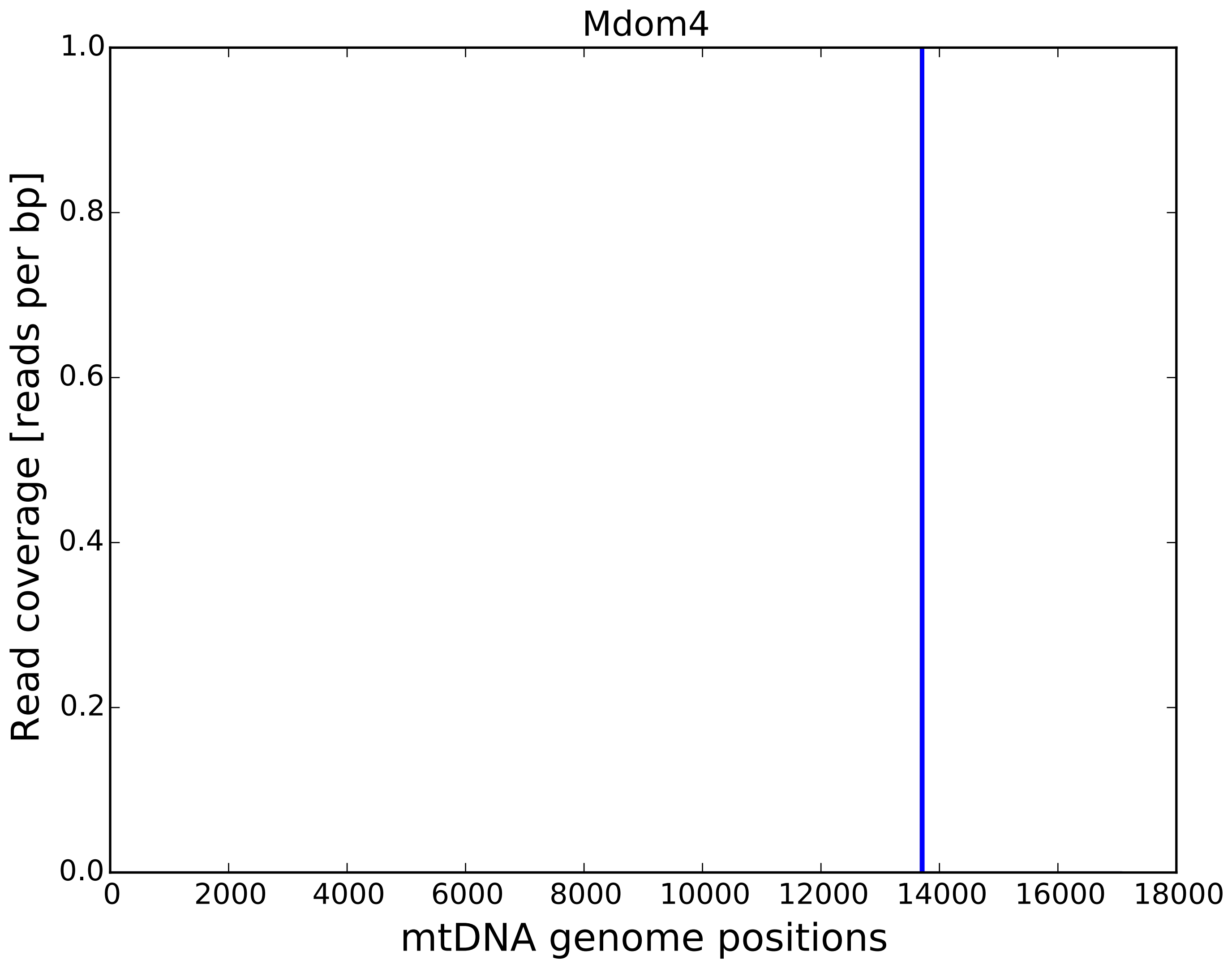

Mdom4
Read coverage [reads per bp]
mtDNA genome positions
0.0
0.2
0.4
0.6
0.8
1.0
0
2000
4000
6000
8000
10000
12000
14000
16000
18000

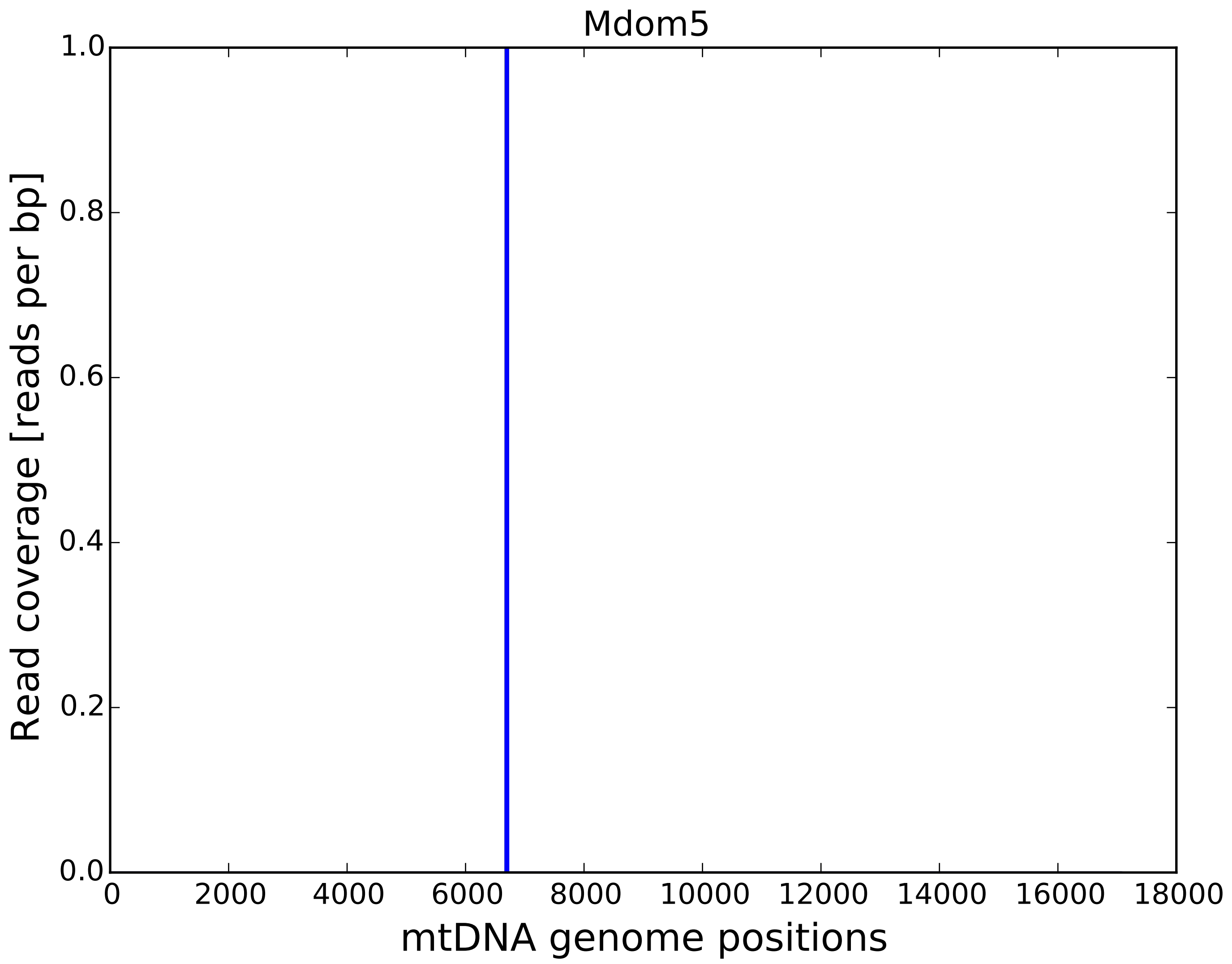
Mdom5
Read coverage [reads per bp]
mtDNA genome positions
0.0
0.2
0.4
0.6
0.8
1.0
0
2000
4000
6000
8000
10000
12000
14000
16000
18000

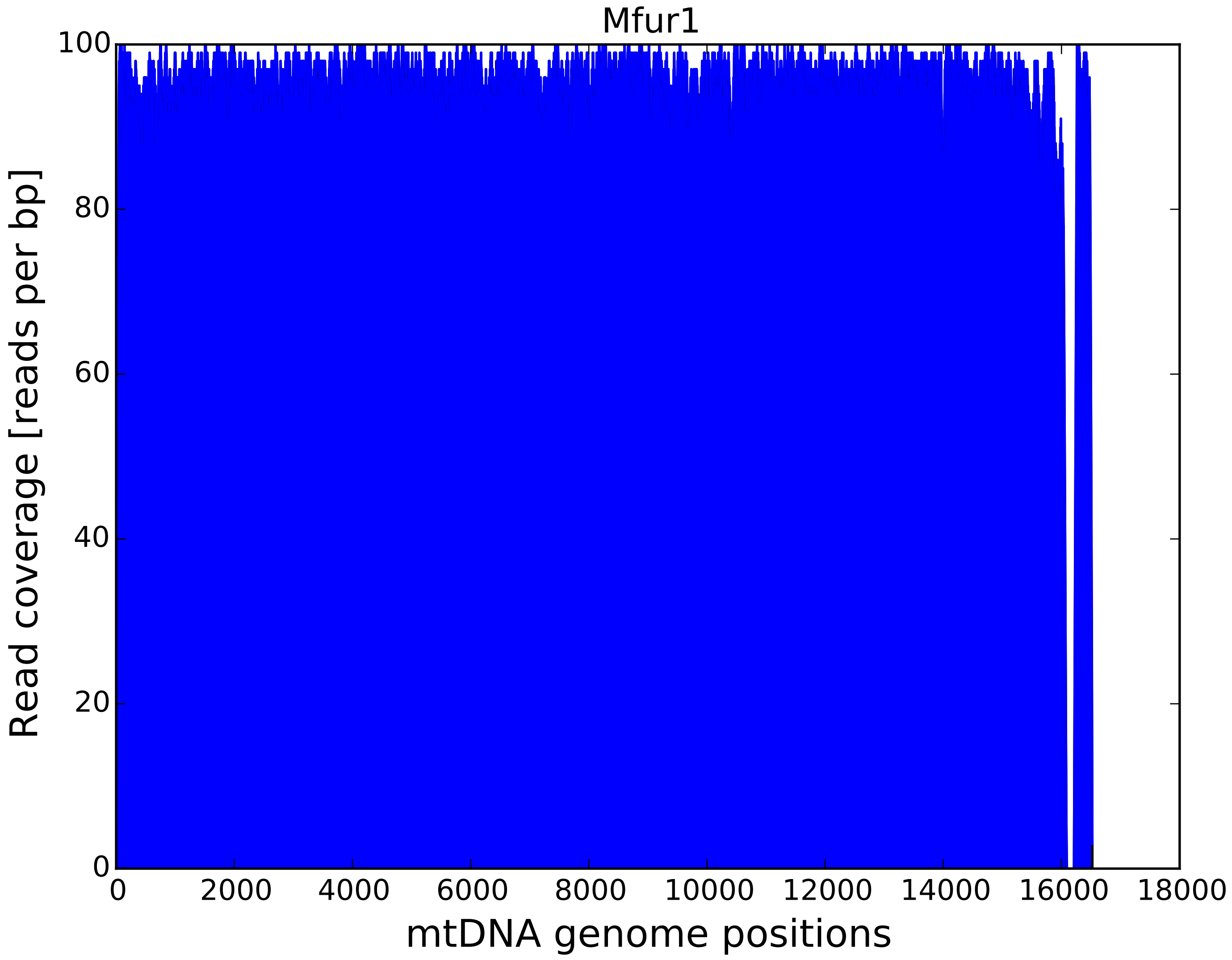

Mfur1
Read coverage [reads per bp]
100
80
60
40
20
0
0
2000
4000
6000
8000
10000
12000
14000
16000
18000
mtDNA genome positions

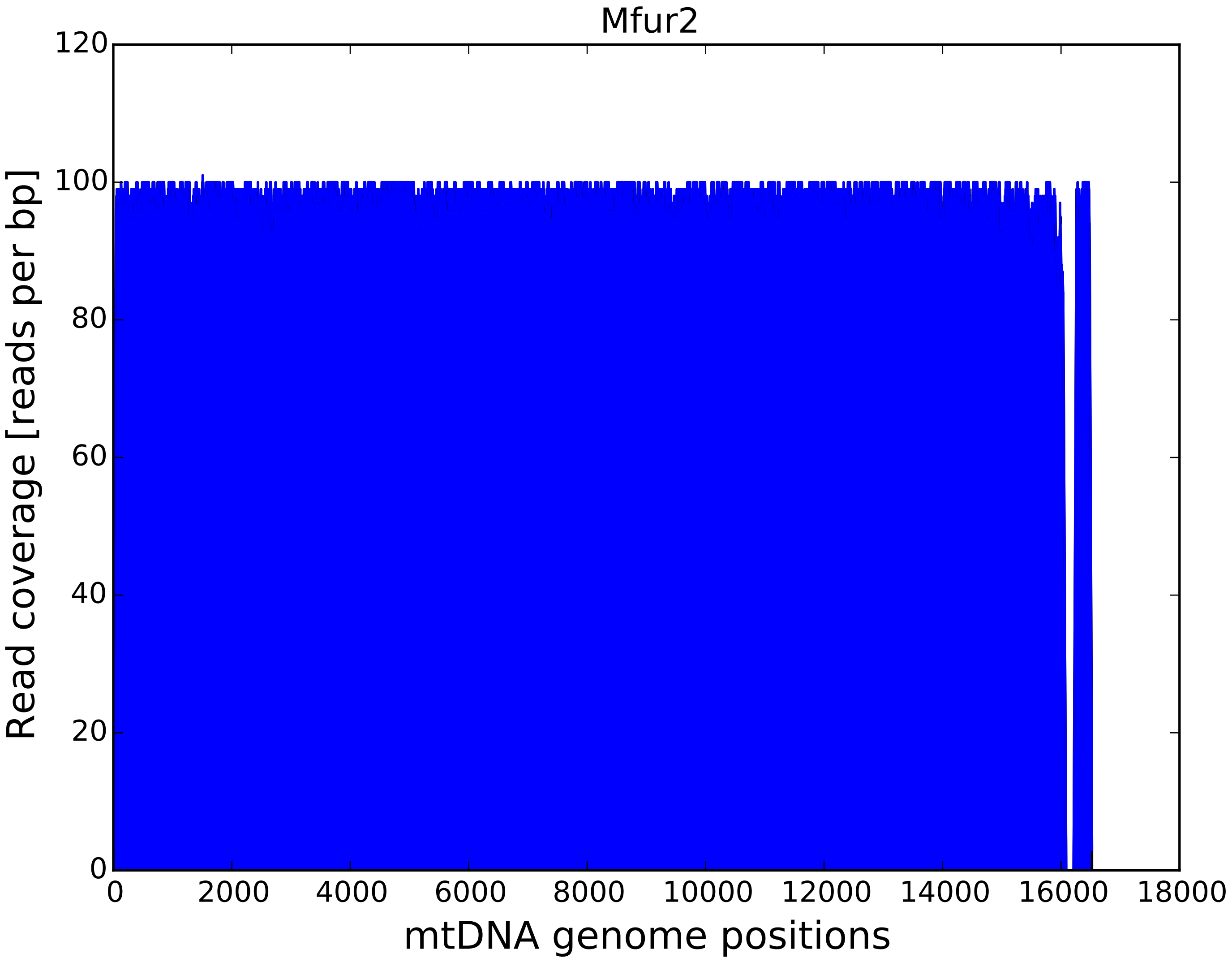

Mfur2
Read coverage [reads per bp]
mtDNA genome positions
0
20
40
60
80
100
120
0
2000
4000
6000
8000
10000
12000
14000
16000
18000

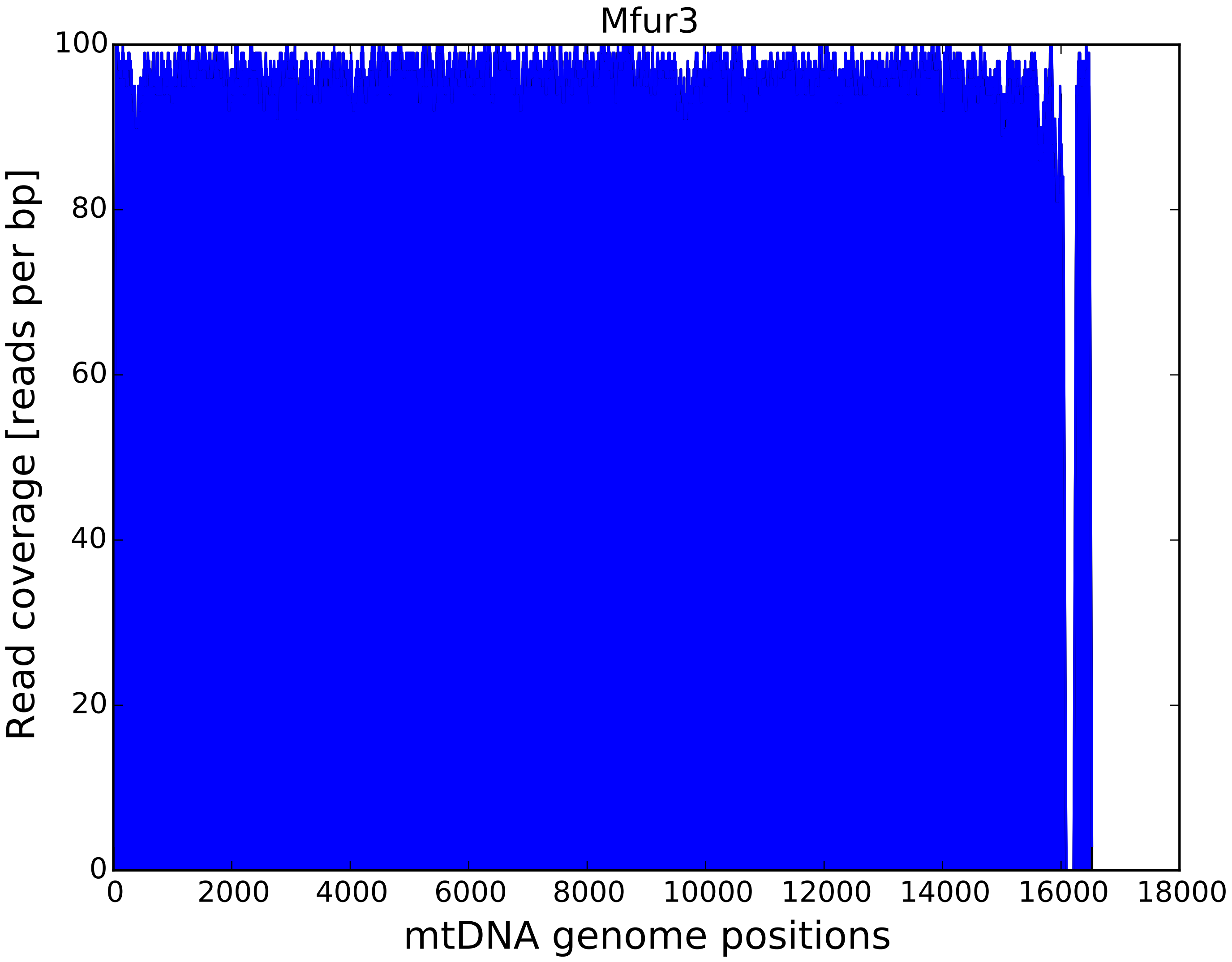

Mfur3
Read coverage [reads per bp]
0
20
40
60
80
100
0
2000
4000
6000
8000
10000
12000
14000
16000
18000
mtDNA genome positions

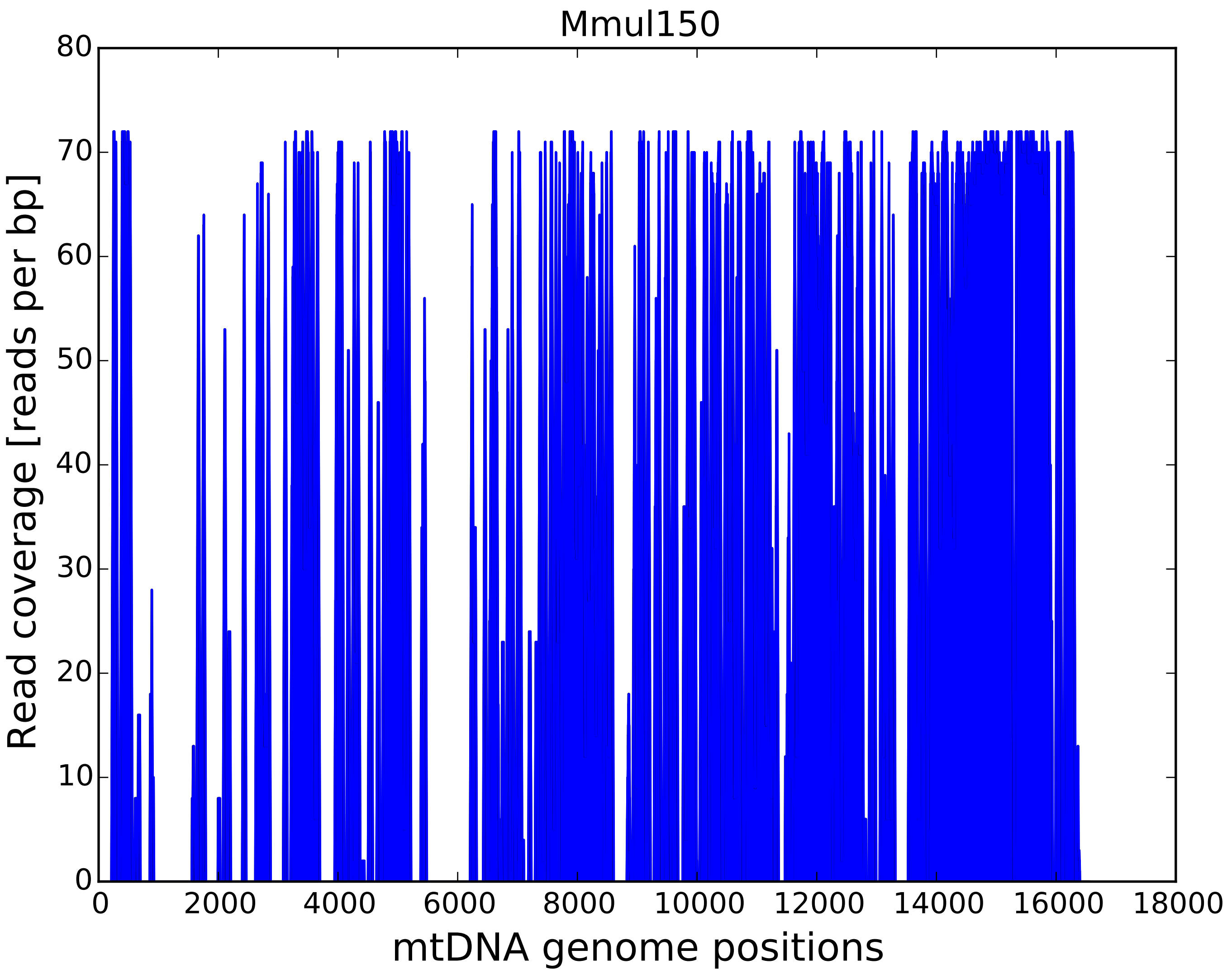

Mmul150
Read coverage [reads per bp]
0
10
20
30
40
50
60
70
80
0
2000
4000
6000
8000
10000
12000
14000
16000
18000
mtDNA genome positions

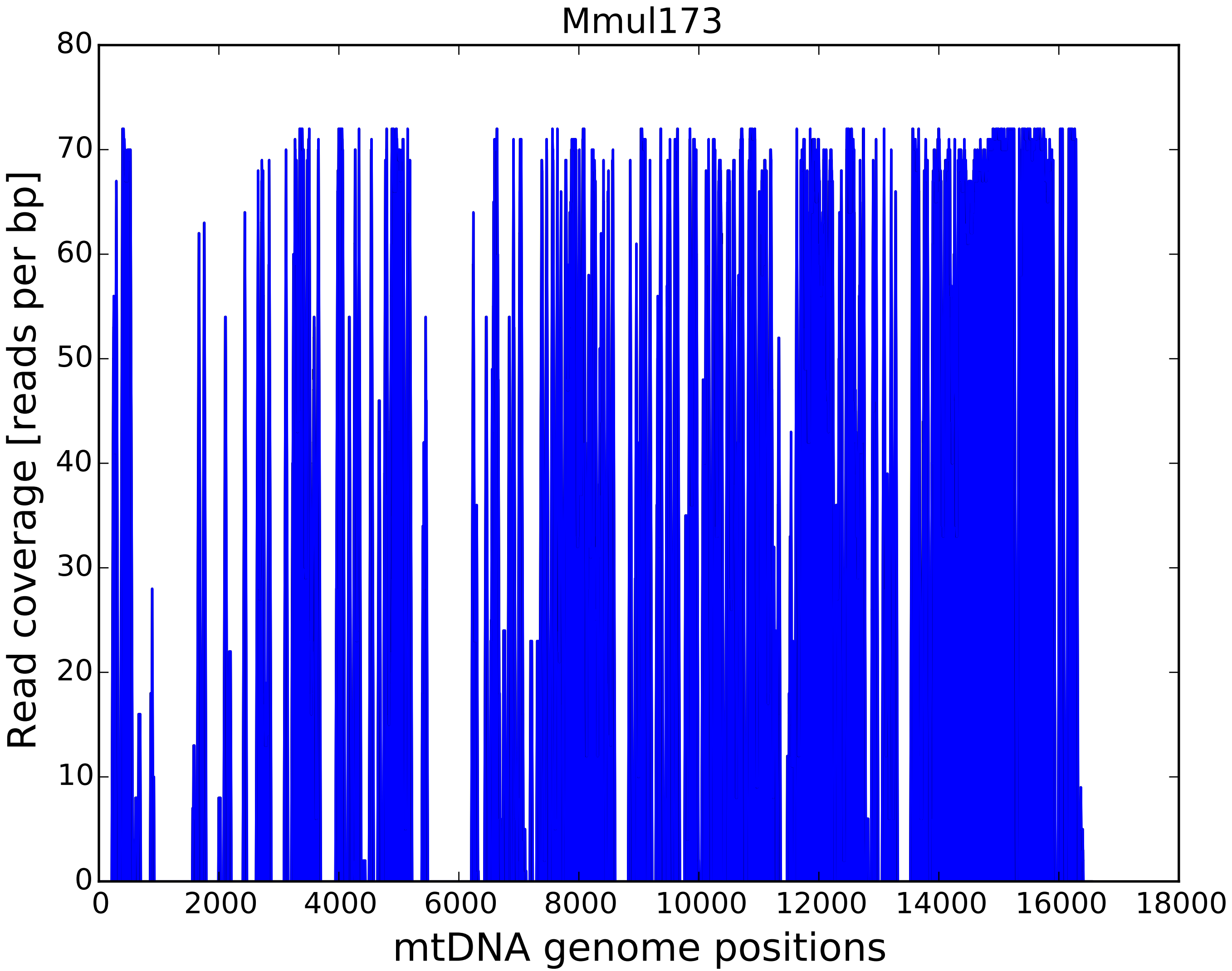

Mmul173
Read coverage [reads per bp]
mtDNA genome positions
0
10
20
30
40
50
60
70
80
0
2000
4000
6000
8000
10000
12000
14000
16000
18000

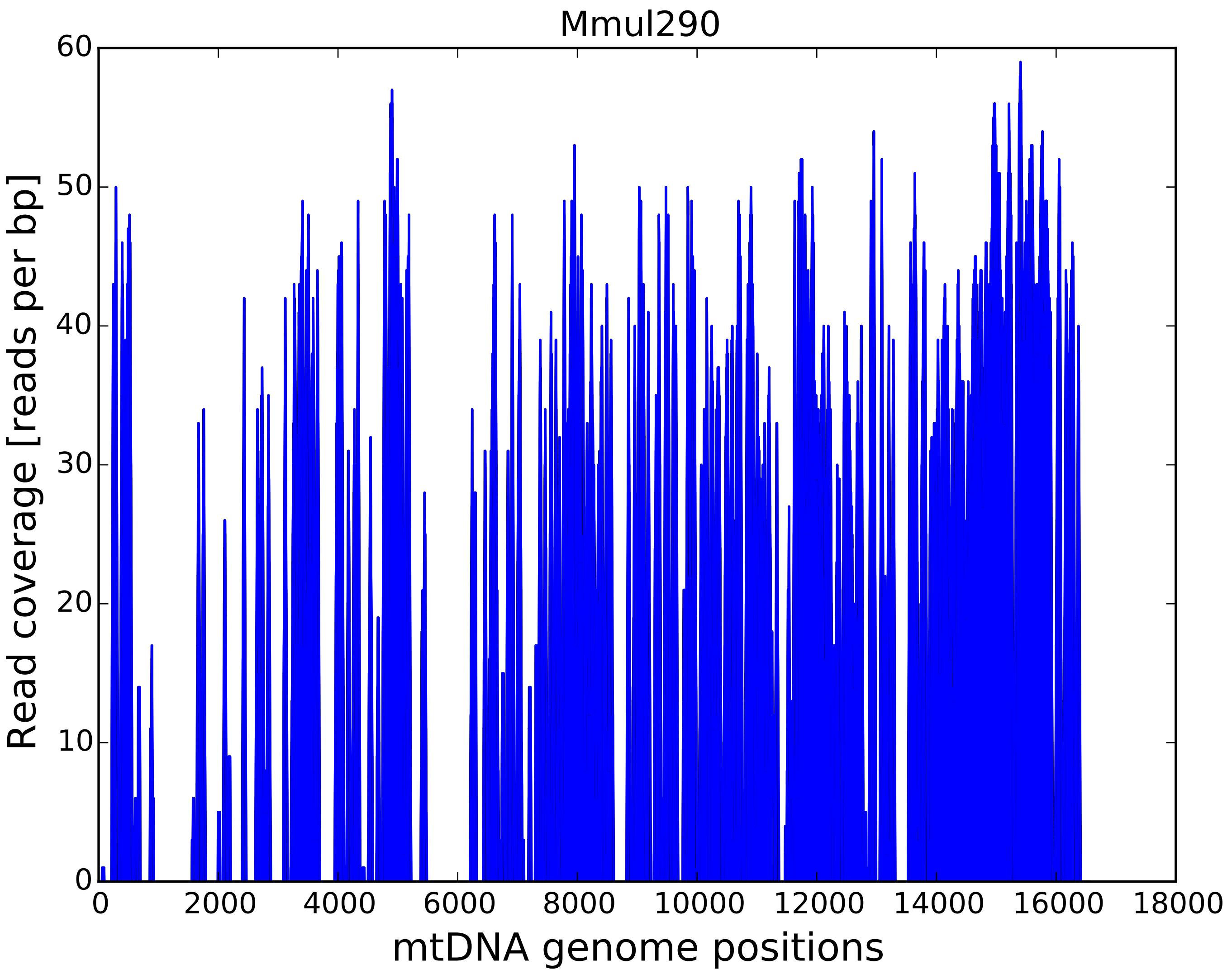

Mmul290
Read coverage [reads per bp]
mtDNA genome positions
0
10
20
30
40
50
60
0
2000
4000
6000
8000
10000
12000
14000
16000
18000

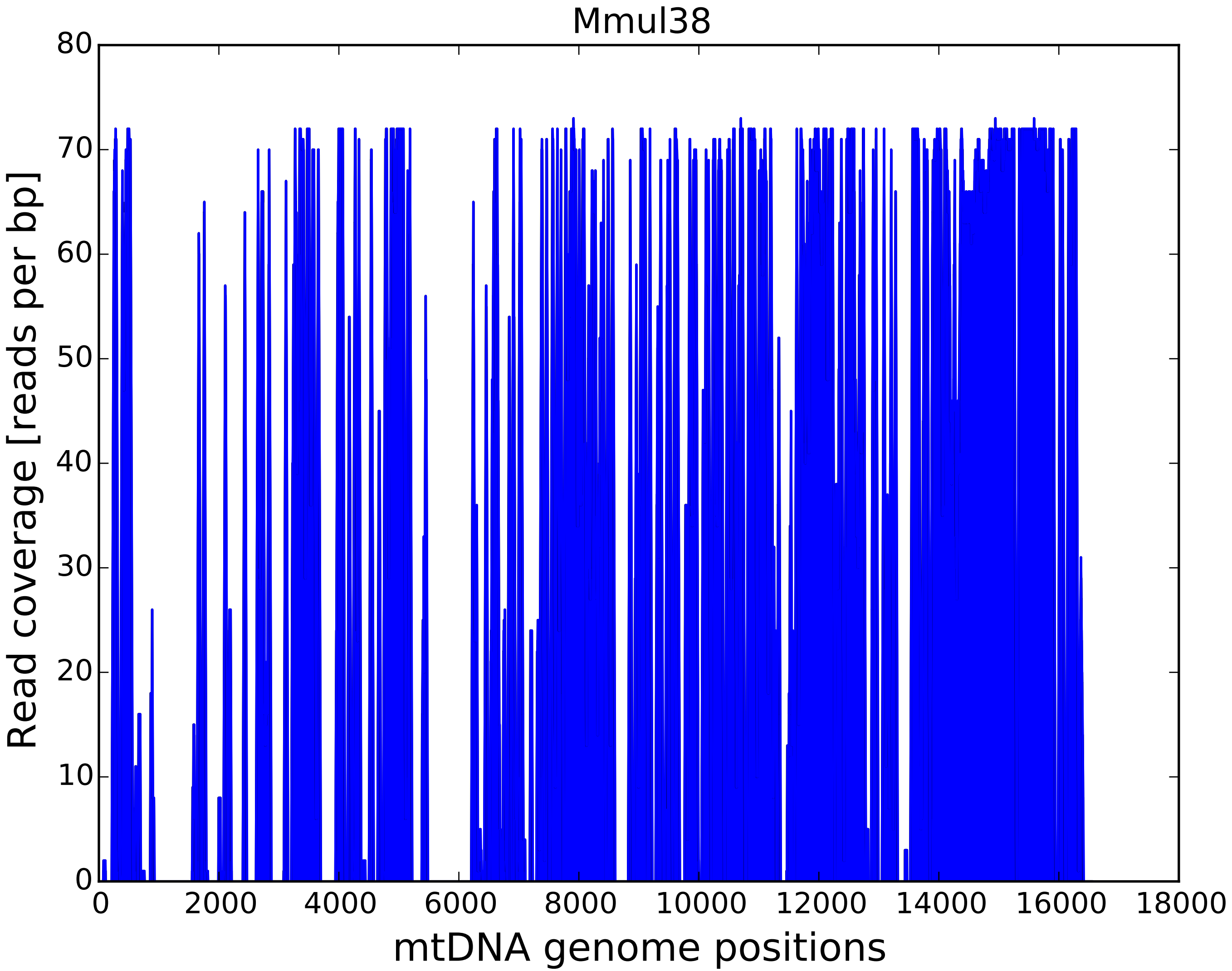

Mmul38
Read coverage [reads per bp]
mtDNA genome positions
0
10
20
30
40
50
60
70
80
0
2000
4000
6000
8000
10000
12000
14000
16000
18000

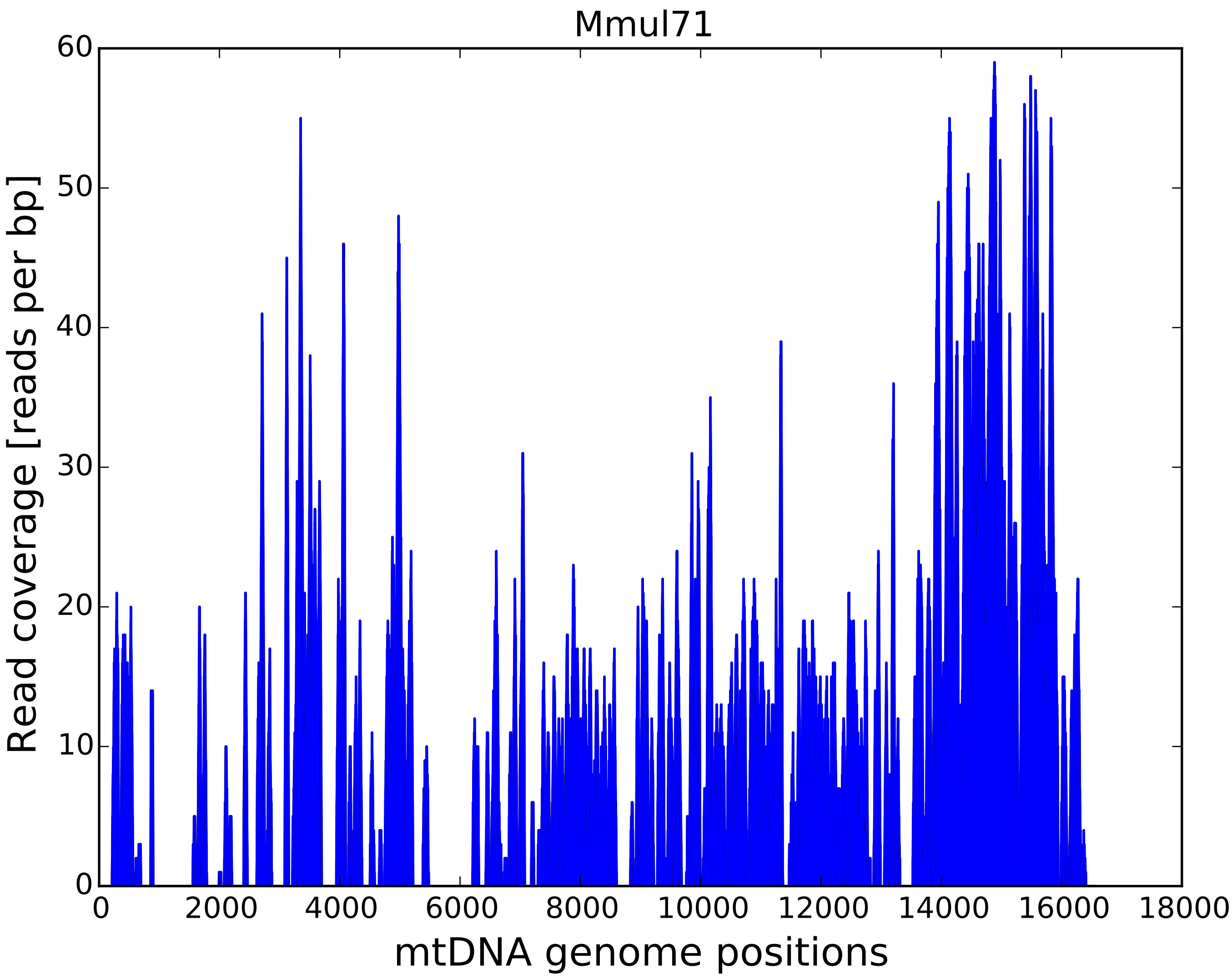
Mmul71
Read coverage [reads per bp]
mtDNA genome positions
0
10
20
30
40
50
60
0
2000
4000
6000
8000
10000
12000
14000
16000
18000

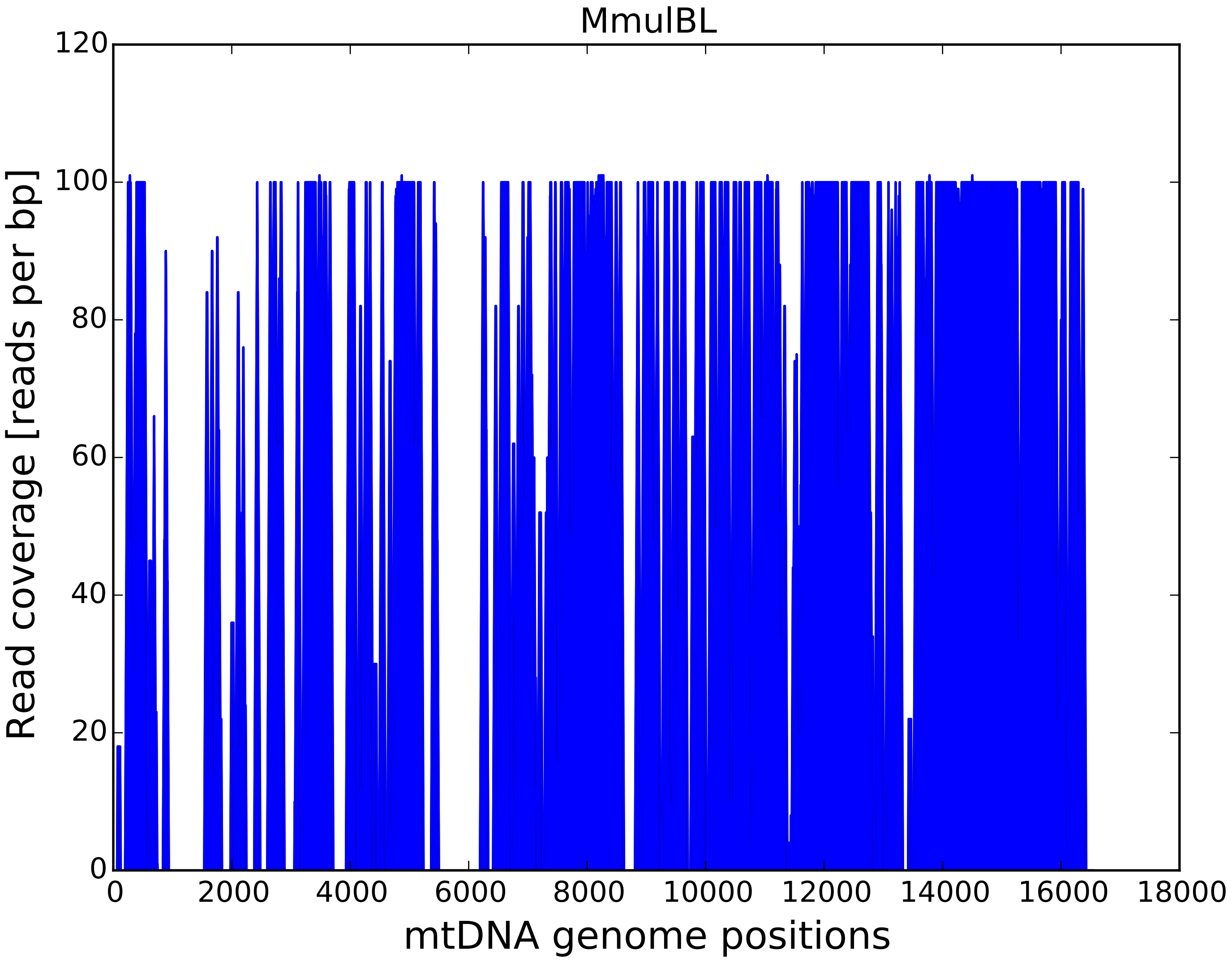
MmulBL
Read coverage [reads per bp]
mtDNA genome positions
0
20
40
60
80
100
120
0
2000
4000
6000
8000
10000
12000
14000
16000
18000

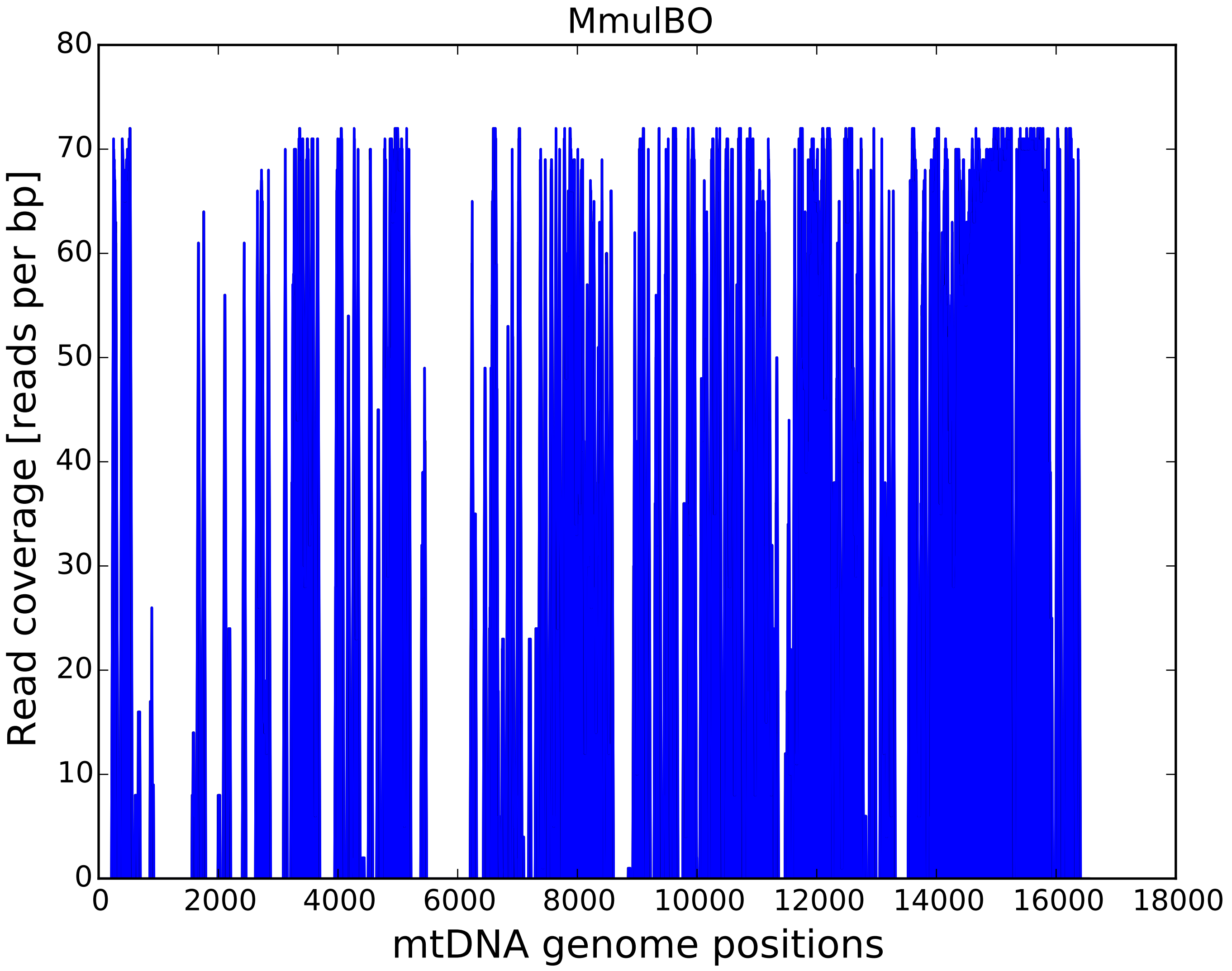

MmulBO
Read coverage [reads per bp]
mtDNA genome positions
0
10
20
30
40
50
60
70
80
0
2000
4000
6000
8000
10000
12000
14000
16000
18000

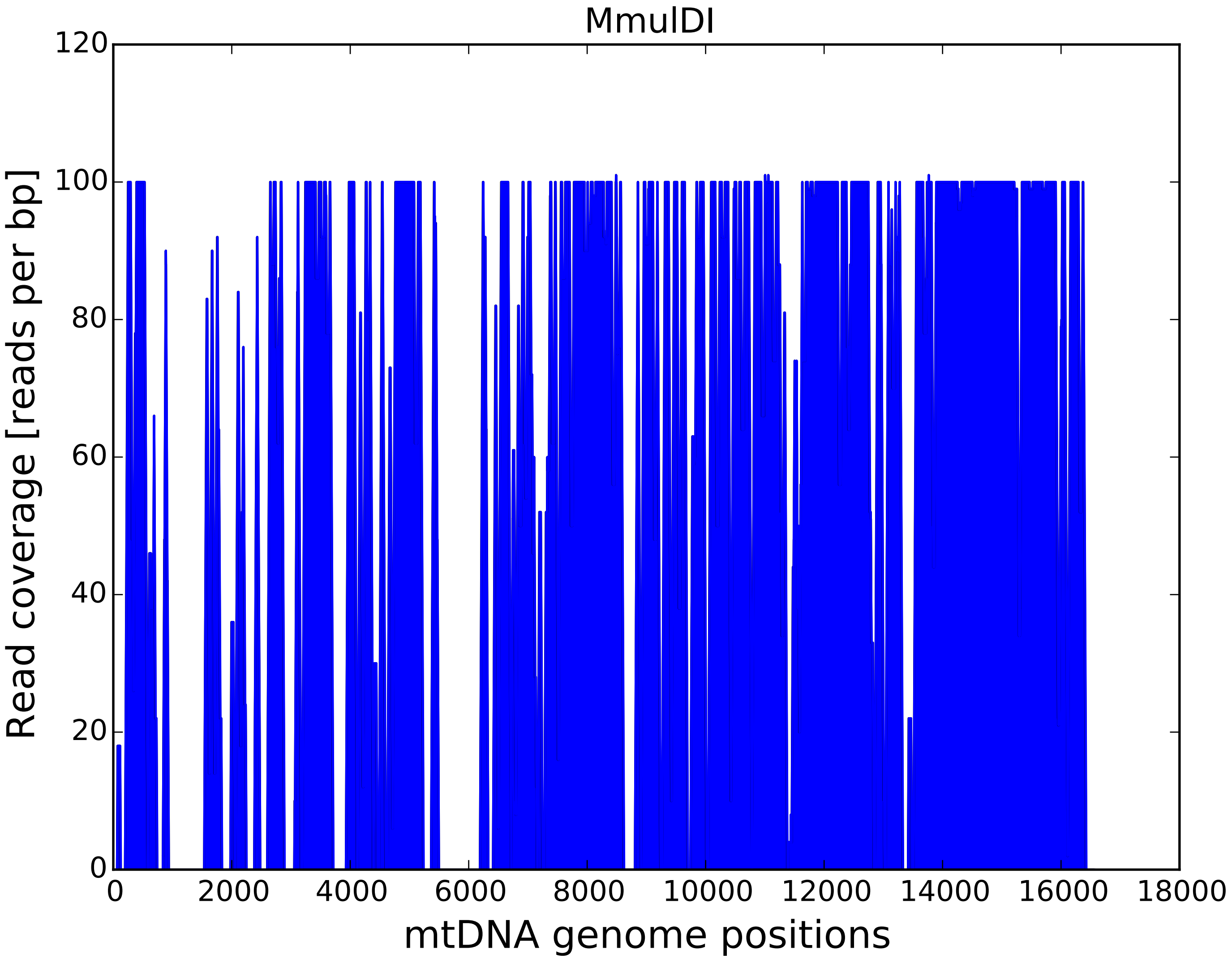

MmulDI
Read coverage [reads per bp]
mtDNA genome positions
0
20
40
60
80
100
120
0
2000
4000
6000
8000
10000
12000
14000
16000
18000

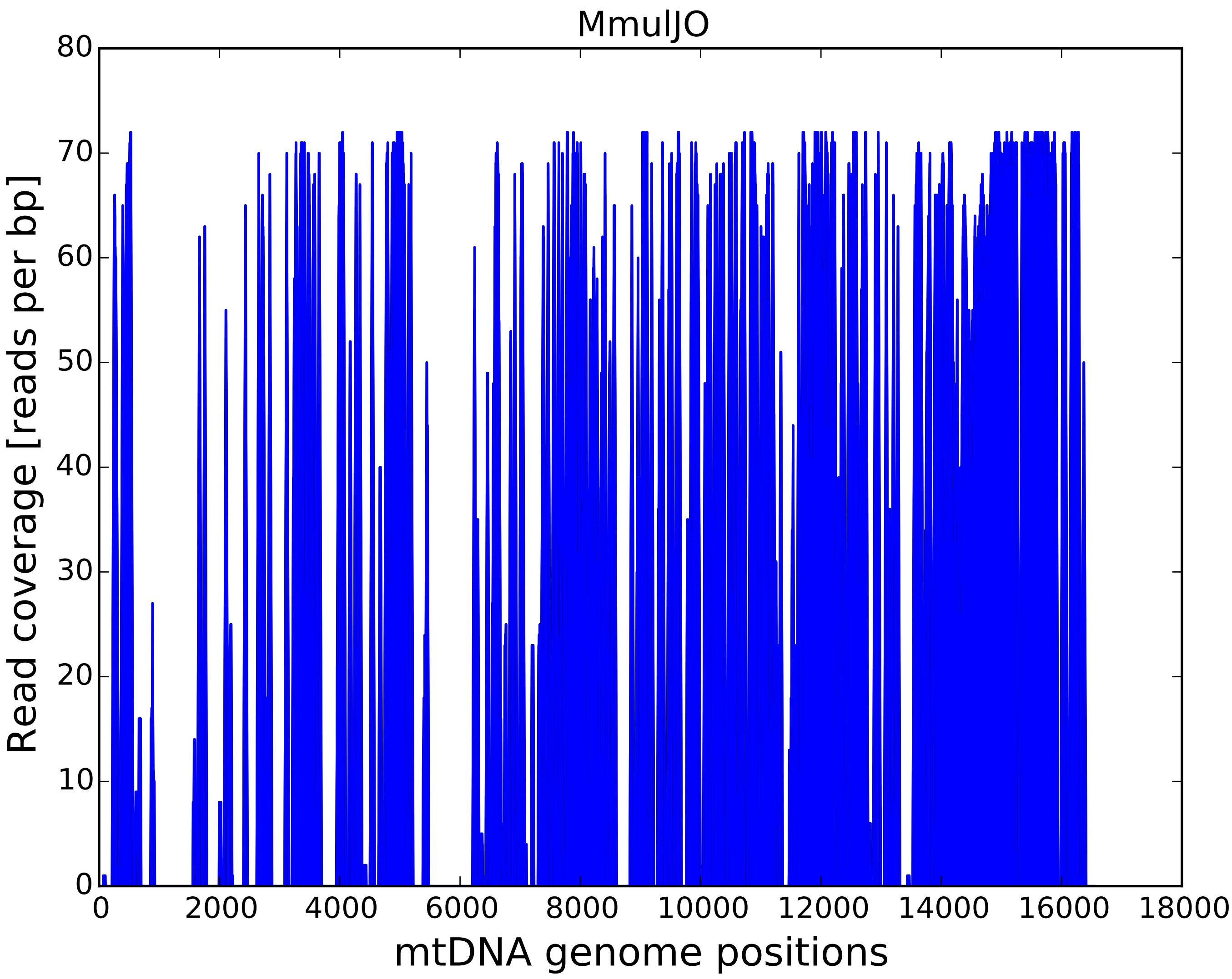
MmulJO
Read coverage [reads per bp]
mtDNA genome positions
0
10
20
30
40
50
60
70
80
0
2000
4000
6000
8000
10000
12000
14000
16000
18000

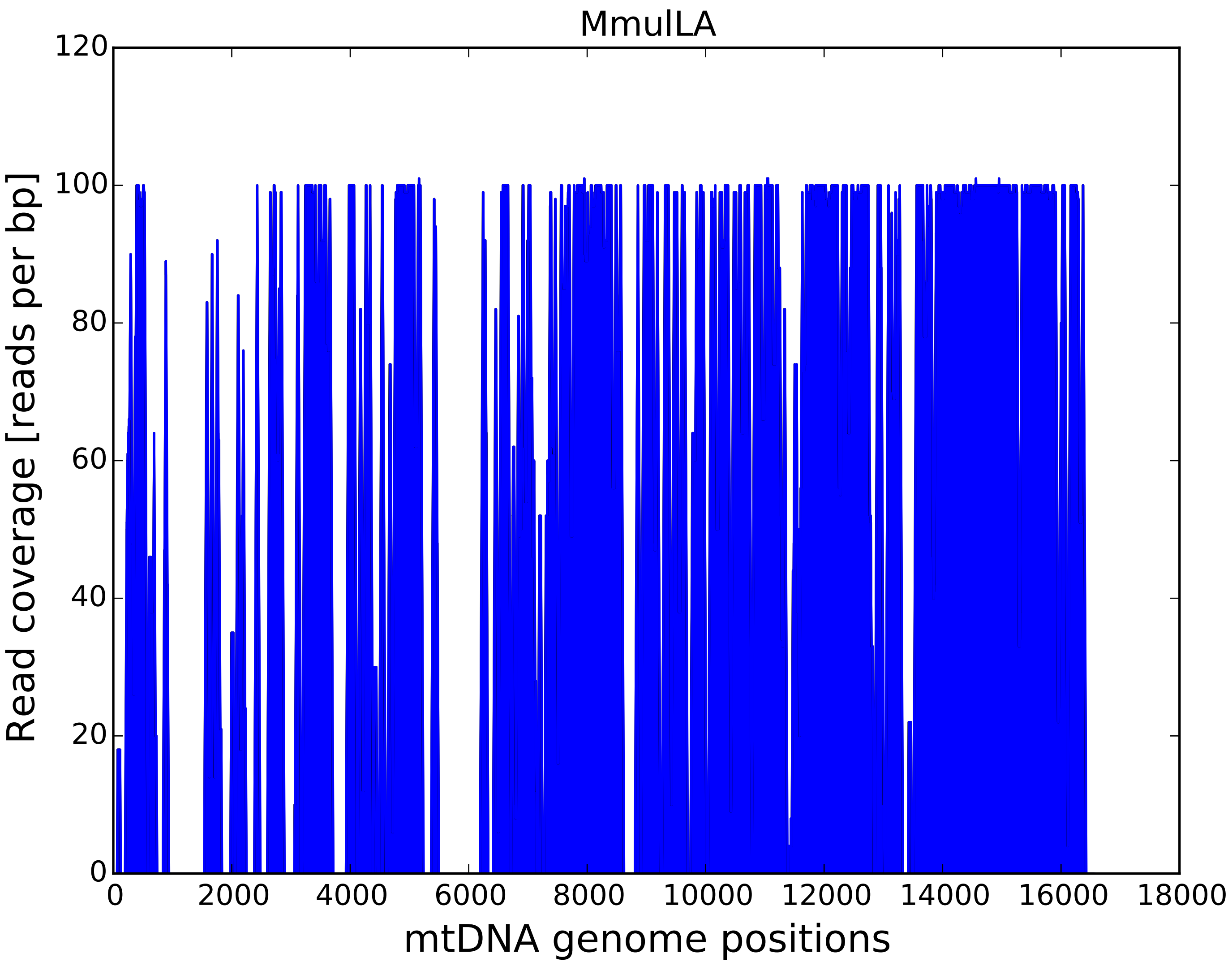
MmulLA
Read coverage [reads per bp]
mtDNA genome positions
0
20
40
60
80
100
120
0
2000
4000
6000
8000
10000
12000
14000
16000
18000

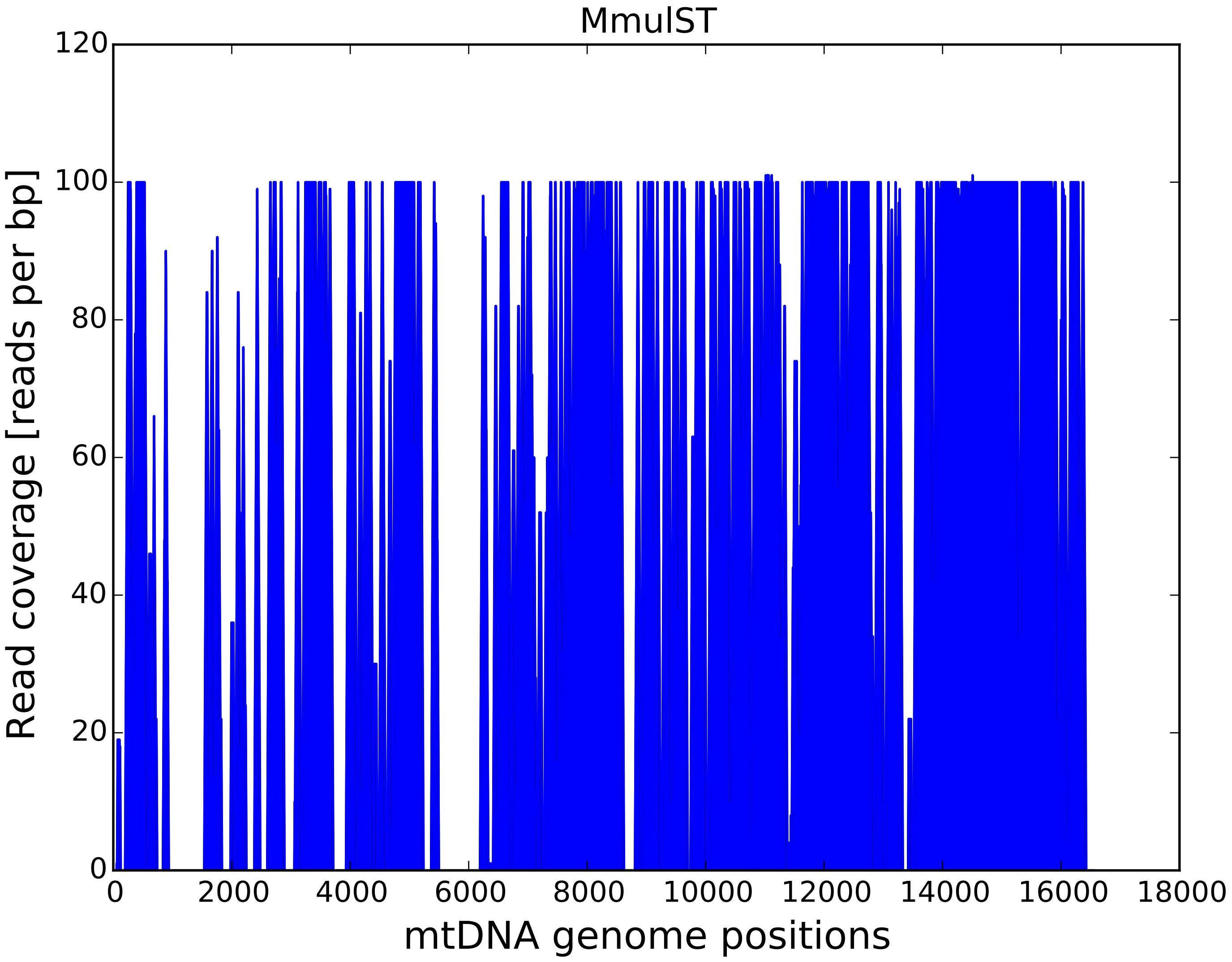

MmulST
Read coverage [reads per bp]
mtDNA genome positions
0
20
40
60
80
100
120
0
2000
4000
6000
8000
10000
12000
14000
16000
18000

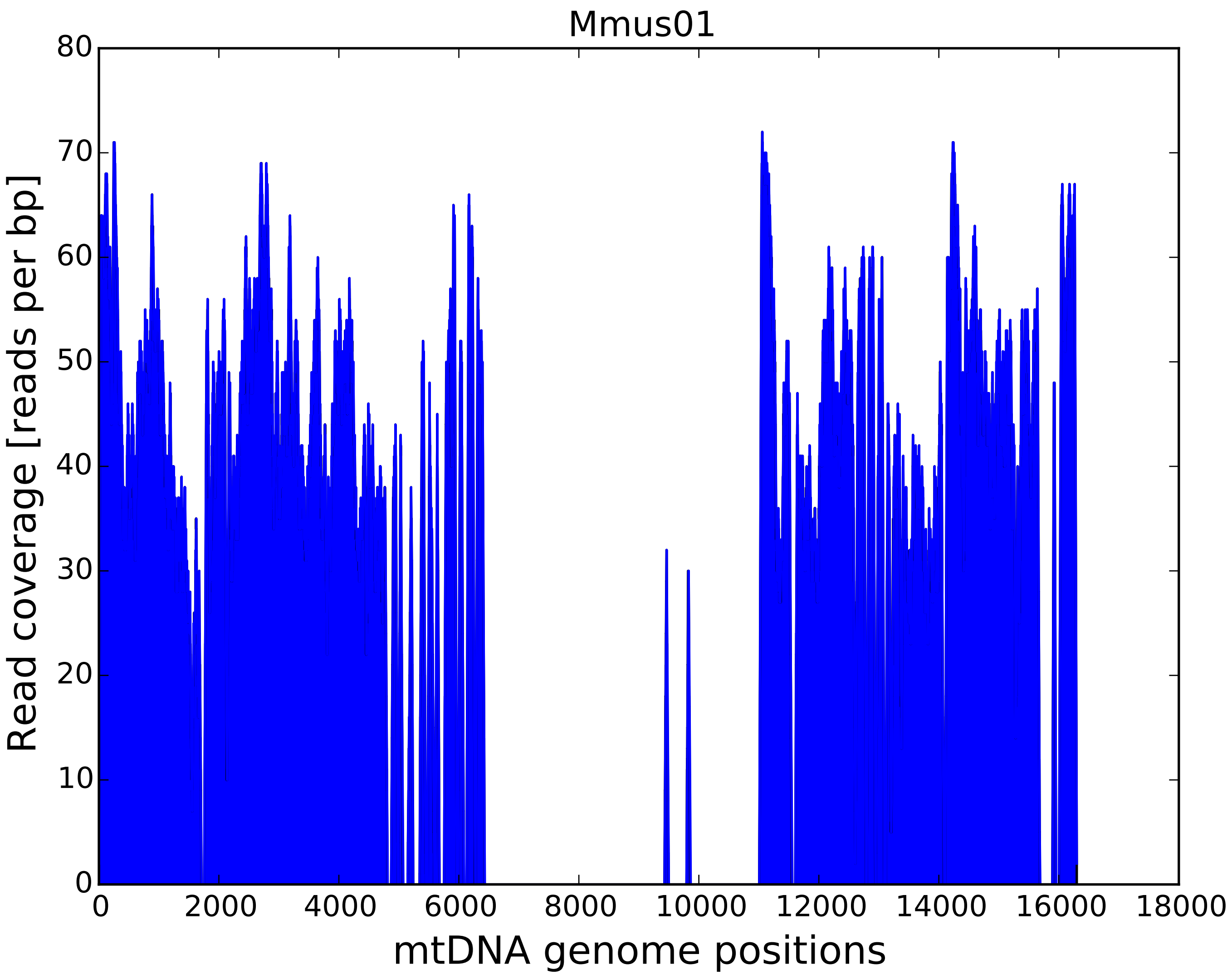

Mmus01
Read coverage [reads per bp]
mtDNA genome positions
0
10
20
30
40
50
60
70
80
0
2000
4000
6000
8000
10000
12000
14000
16000
18000

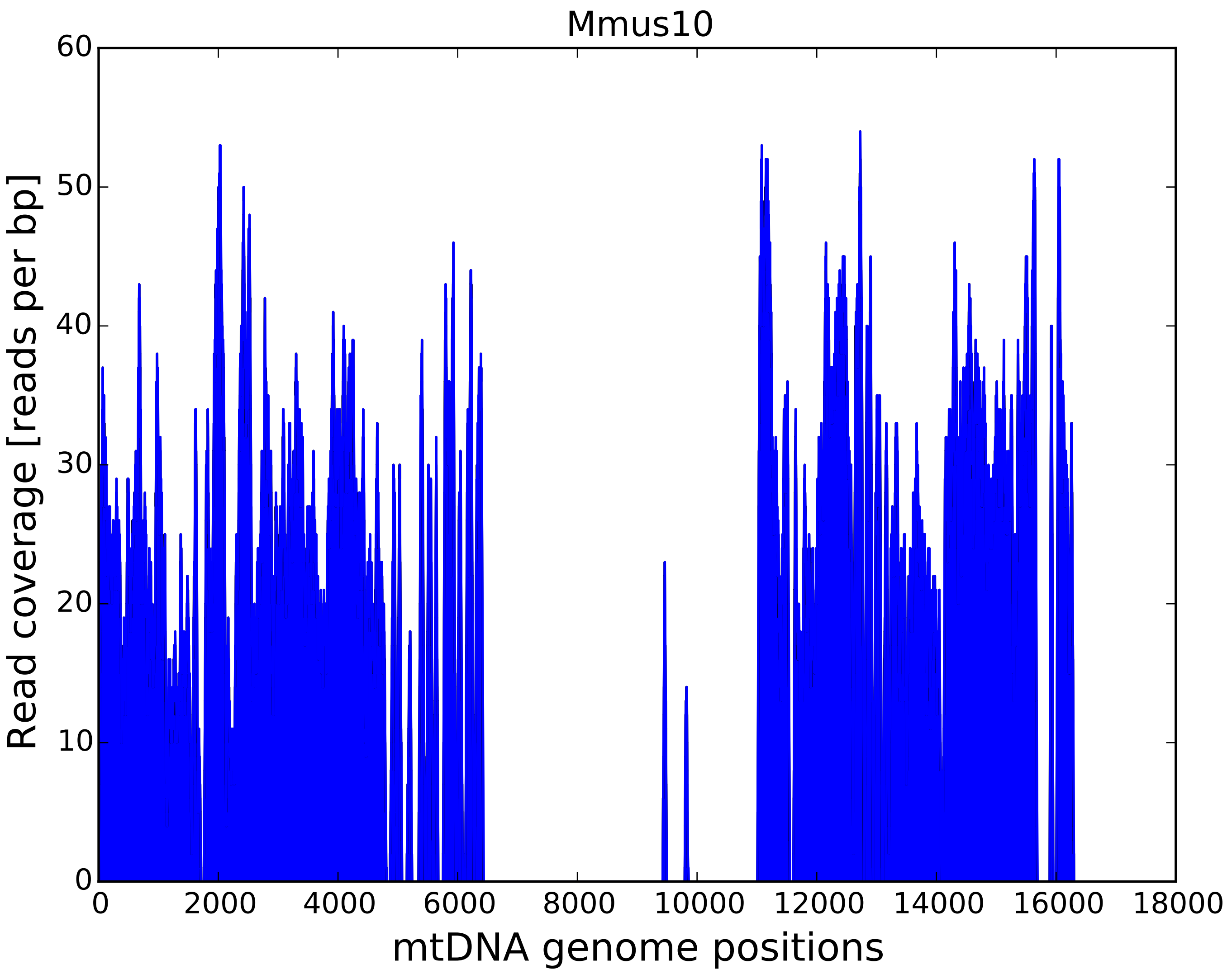
Mmus10
Read coverage [reads per bp]
mtDNA genome positions
0
10
20
30
40
50
60
0
2000
4000
6000
8000
10000
12000
14000
16000
18000

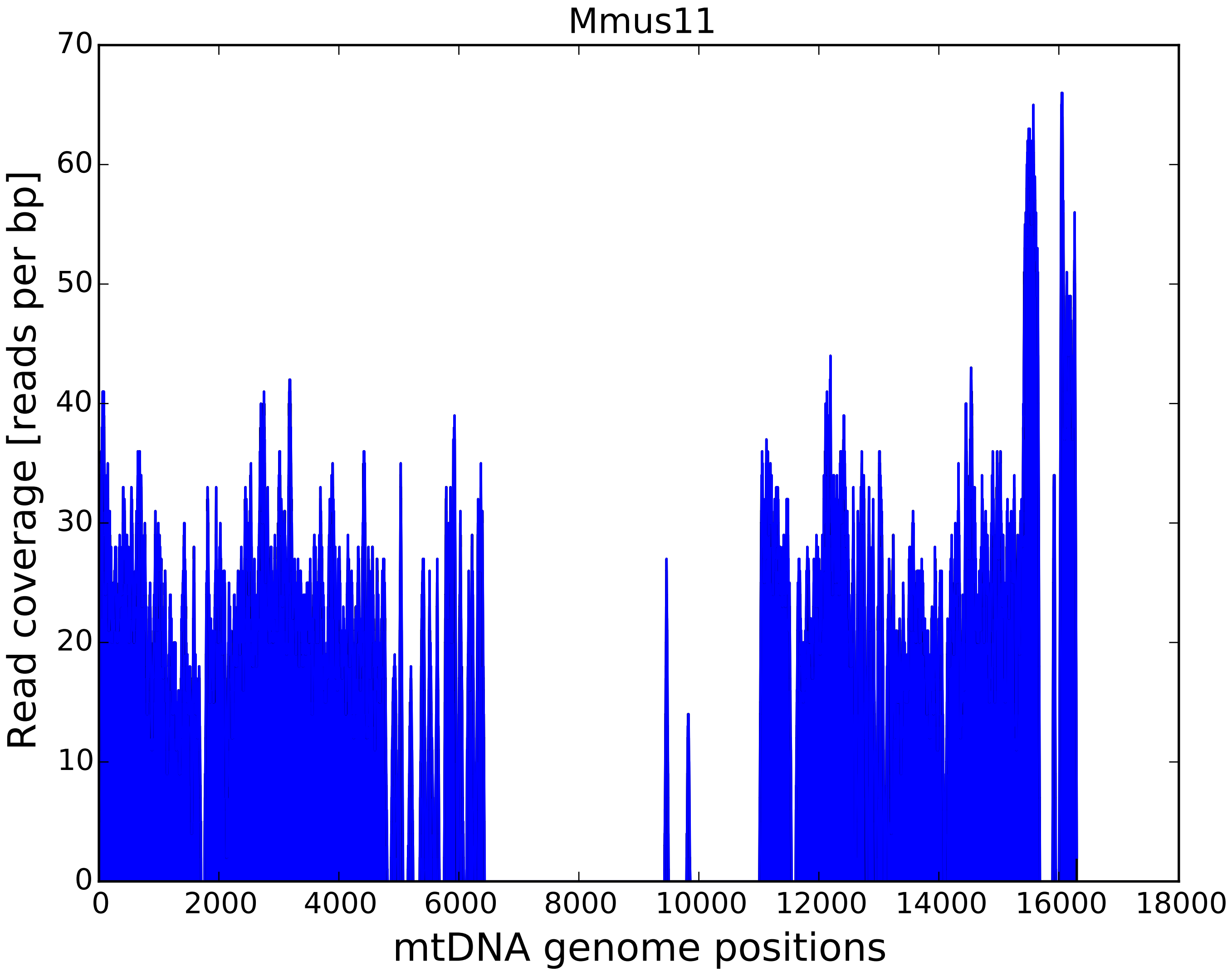

Mmus11
Read coverage [reads per bp]
mtDNA genome positions
0
10
20
30
40
50
60
70
0
2000
4000
6000
8000
10000
12000
14000
16000
18000

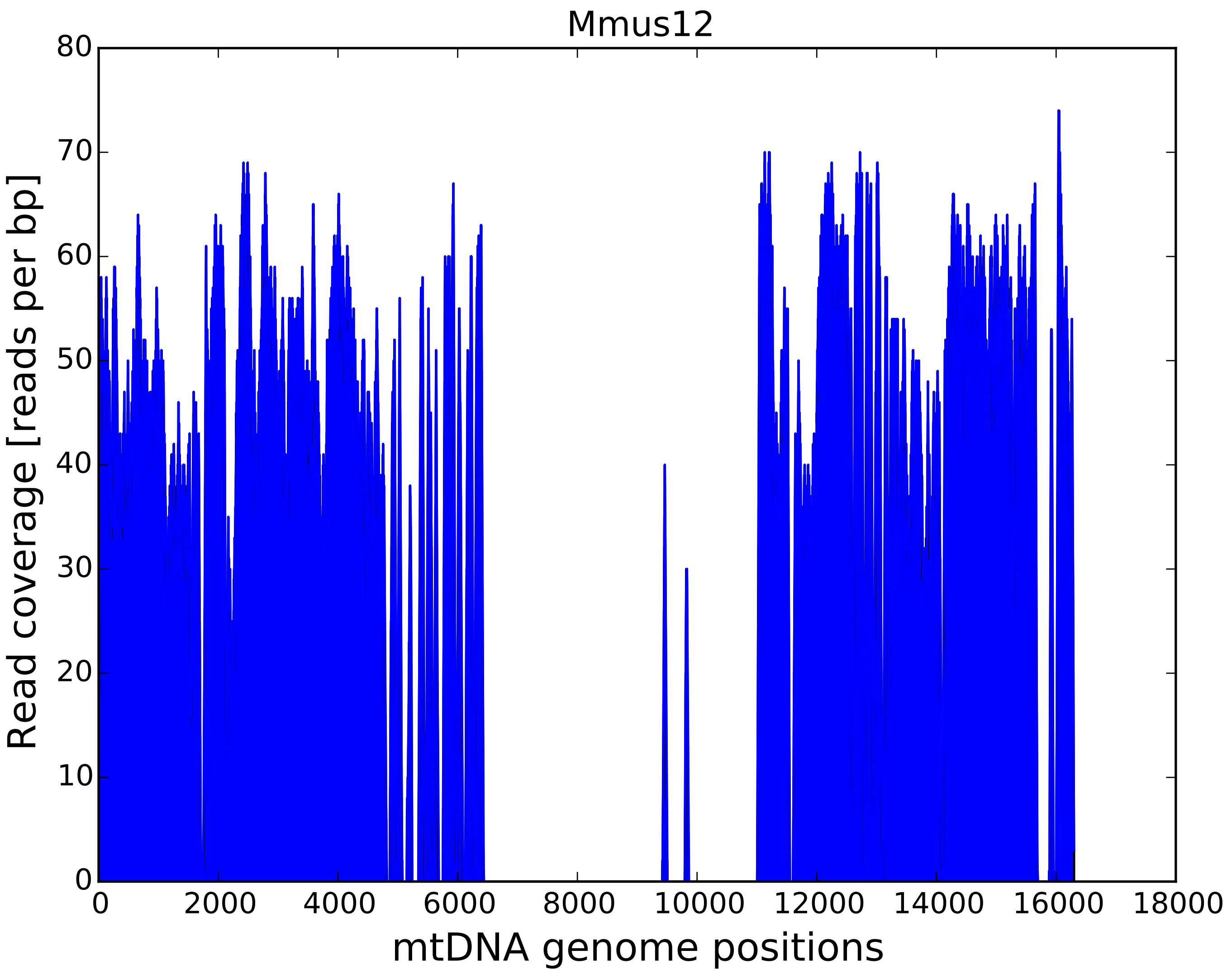
Mmus12
Read coverage [reads per bp]
mtDNA genome positions
0
10
20
30
40
50
60
70
80
0
2000
4000
6000
8000
10000
12000
14000
16000
18000

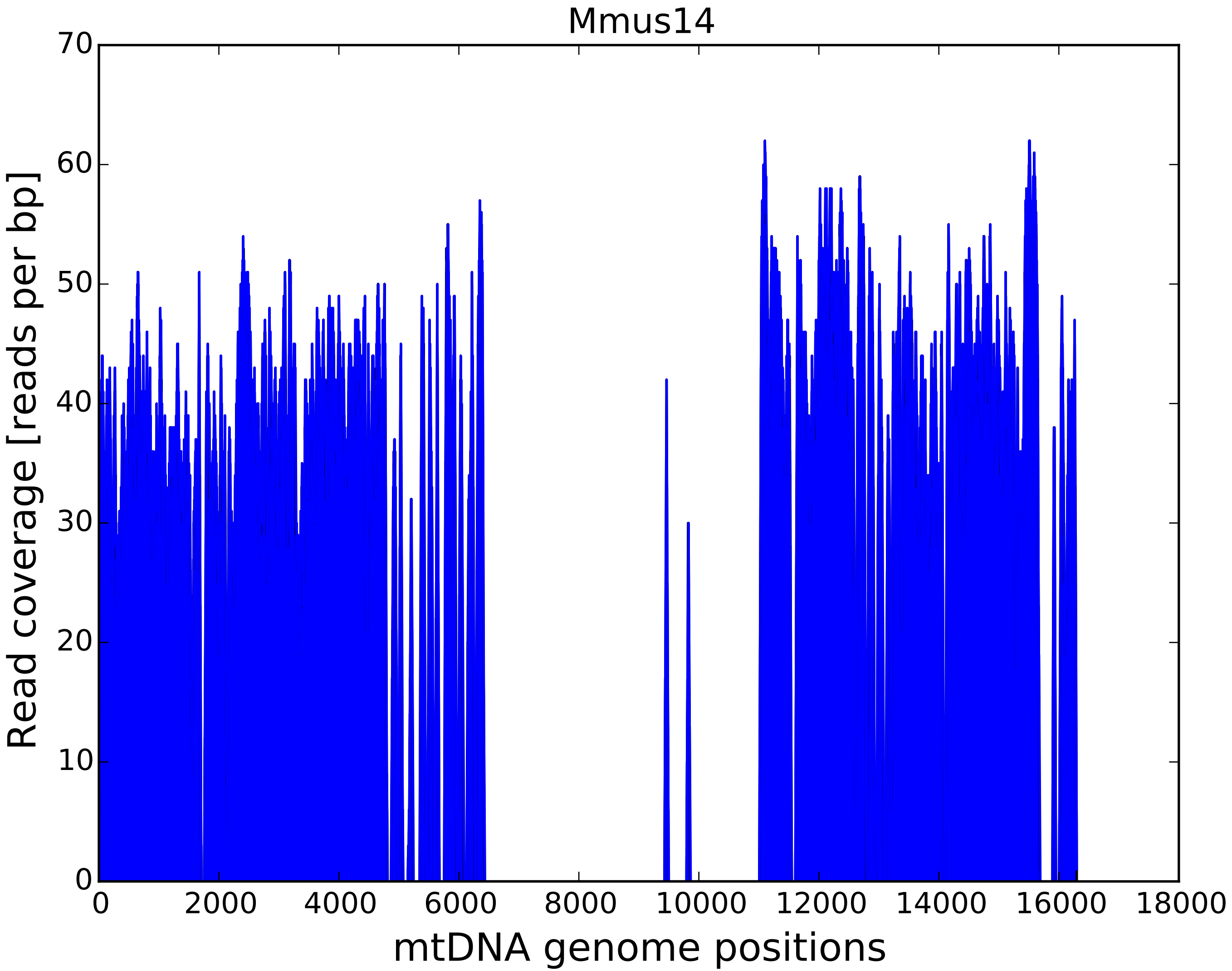

Mmus14
Read coverage [reads per bp]
mtDNA genome positions
0
10
20
30
40
50
60
70
0
2000
4000
6000
8000
10000
12000
14000
16000
18000

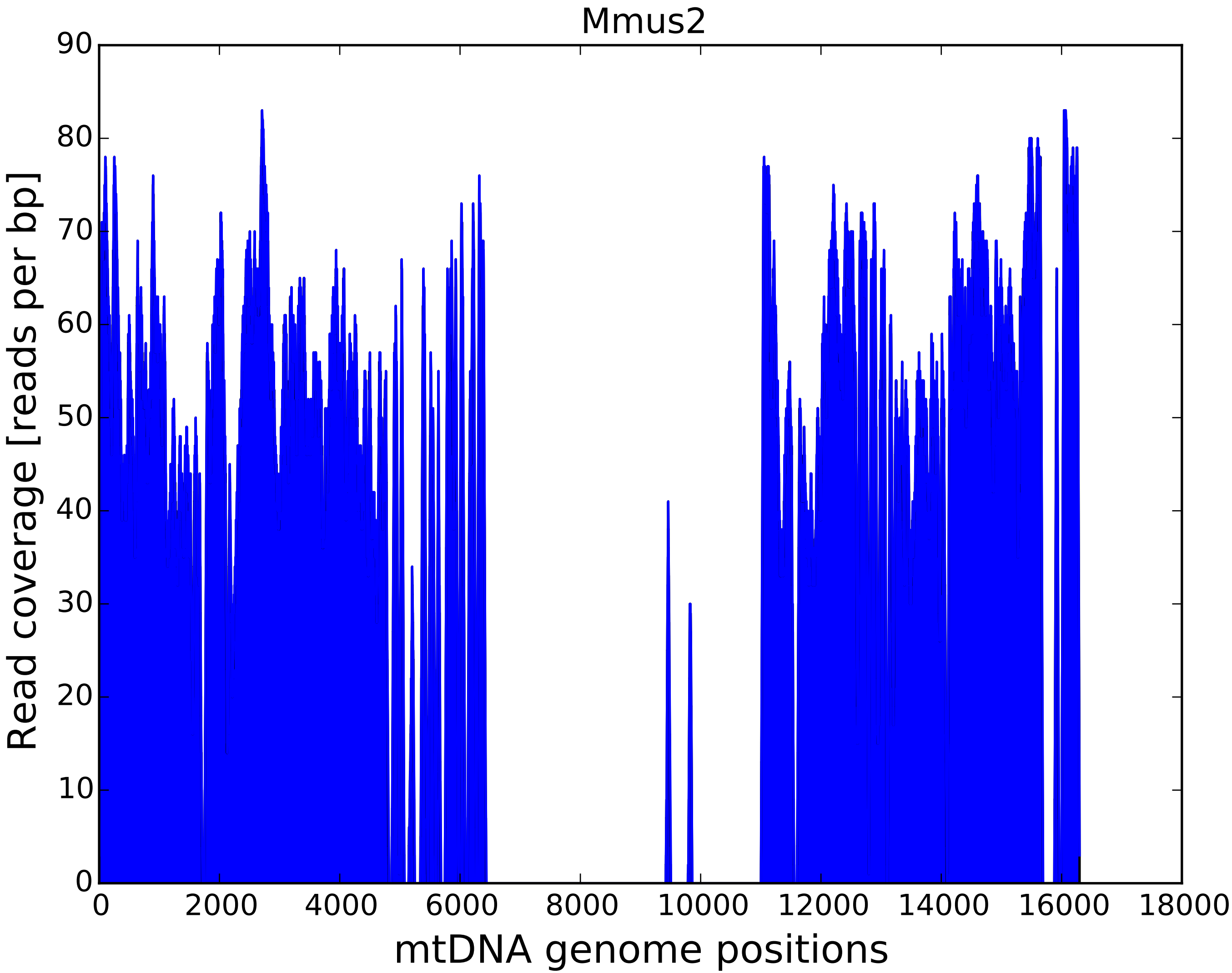

Mmus2
Read coverage [reads per bp]
mtDNA genome positions
0
10
20
30
40
50
60
70
80
90
0
2000
4000
6000
8000
10000
12000
14000
16000
18000

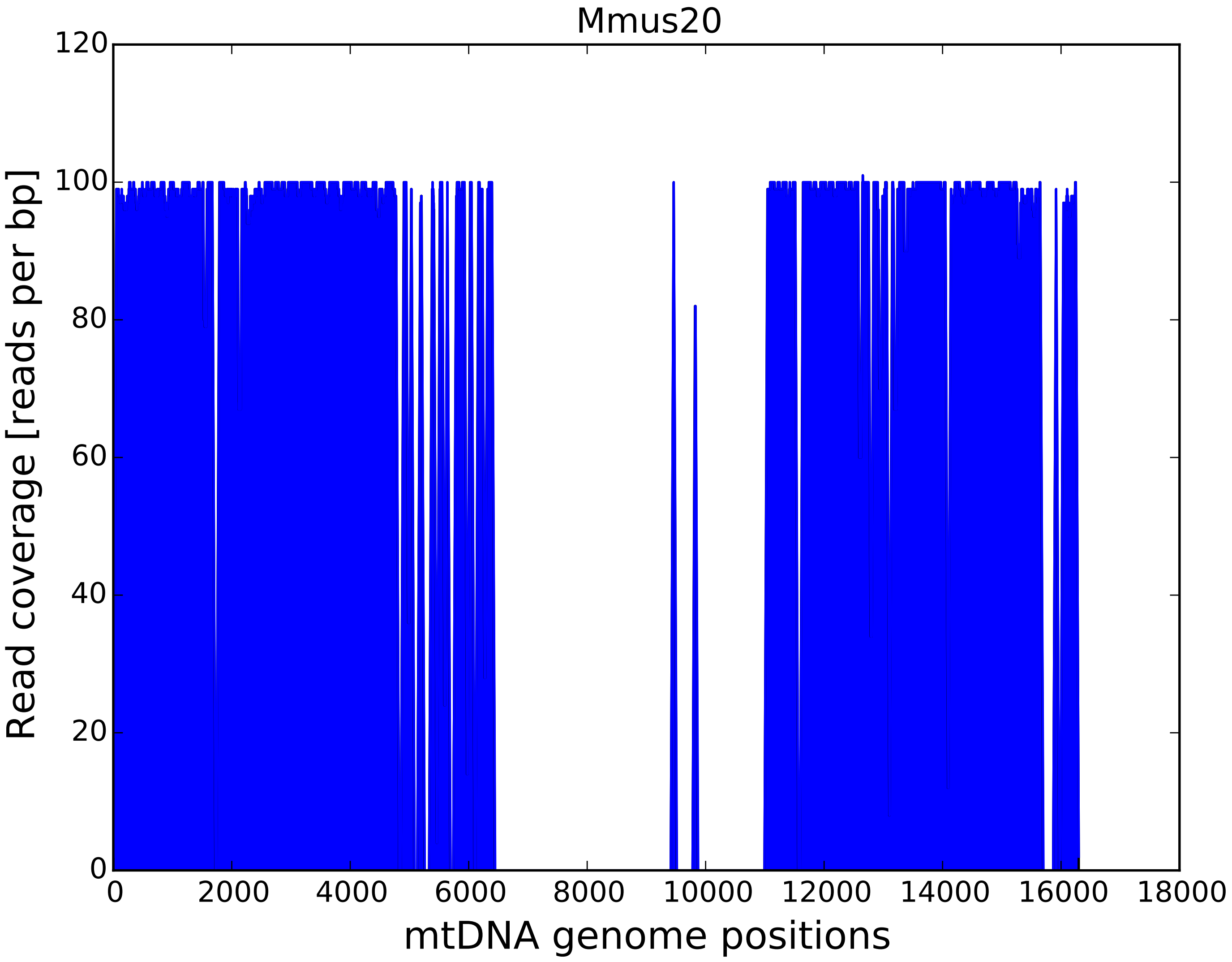

Mmus20
Read coverage [reads per bp]
mtDNA genome positions
0
20
40
60
80
100
120
0
2000
4000
6000
8000
10000
12000
14000
16000
18000

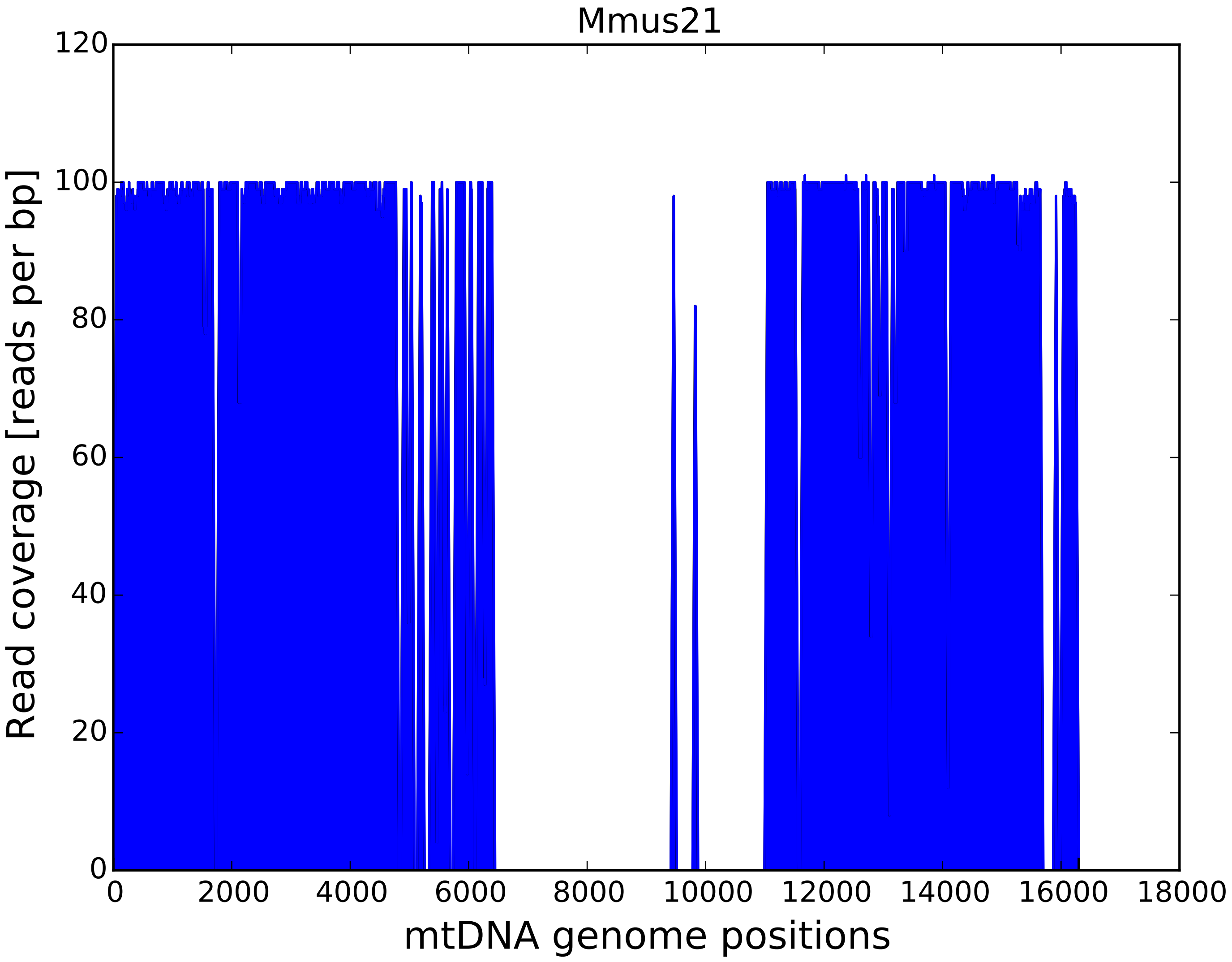

Mmus21
Read coverage [reads per bp]
mtDNA genome positions
0
20
40
60
80
100
120
2000
4000
6000
8000
10000
12000
14000
16000
18000

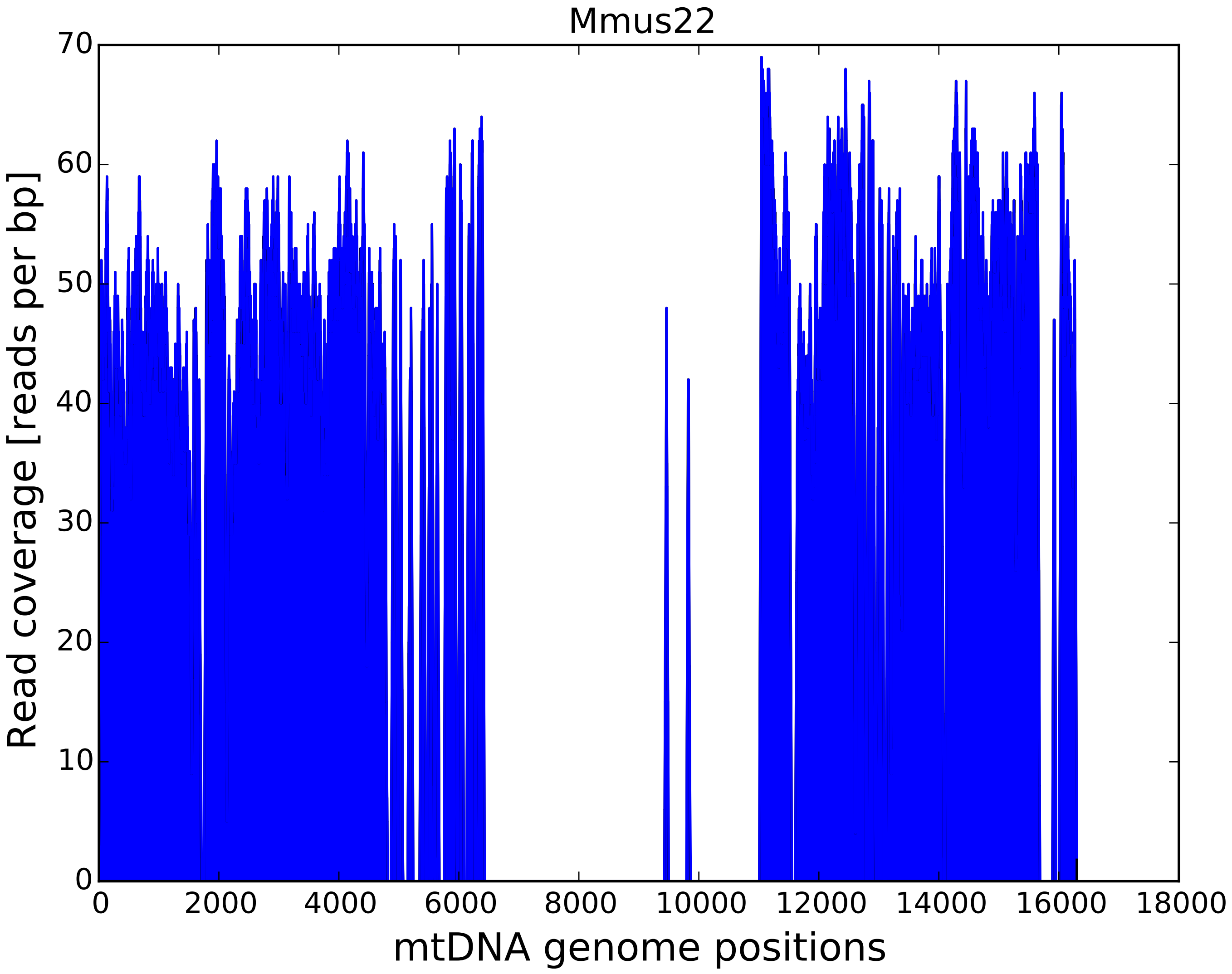
Mmus22
Read coverage [reads per bp]
mtDNA genome positions
0
10
20
30
40
50
60
70
0
2000
4000
6000
8000
10000
12000
14000
16000
18000

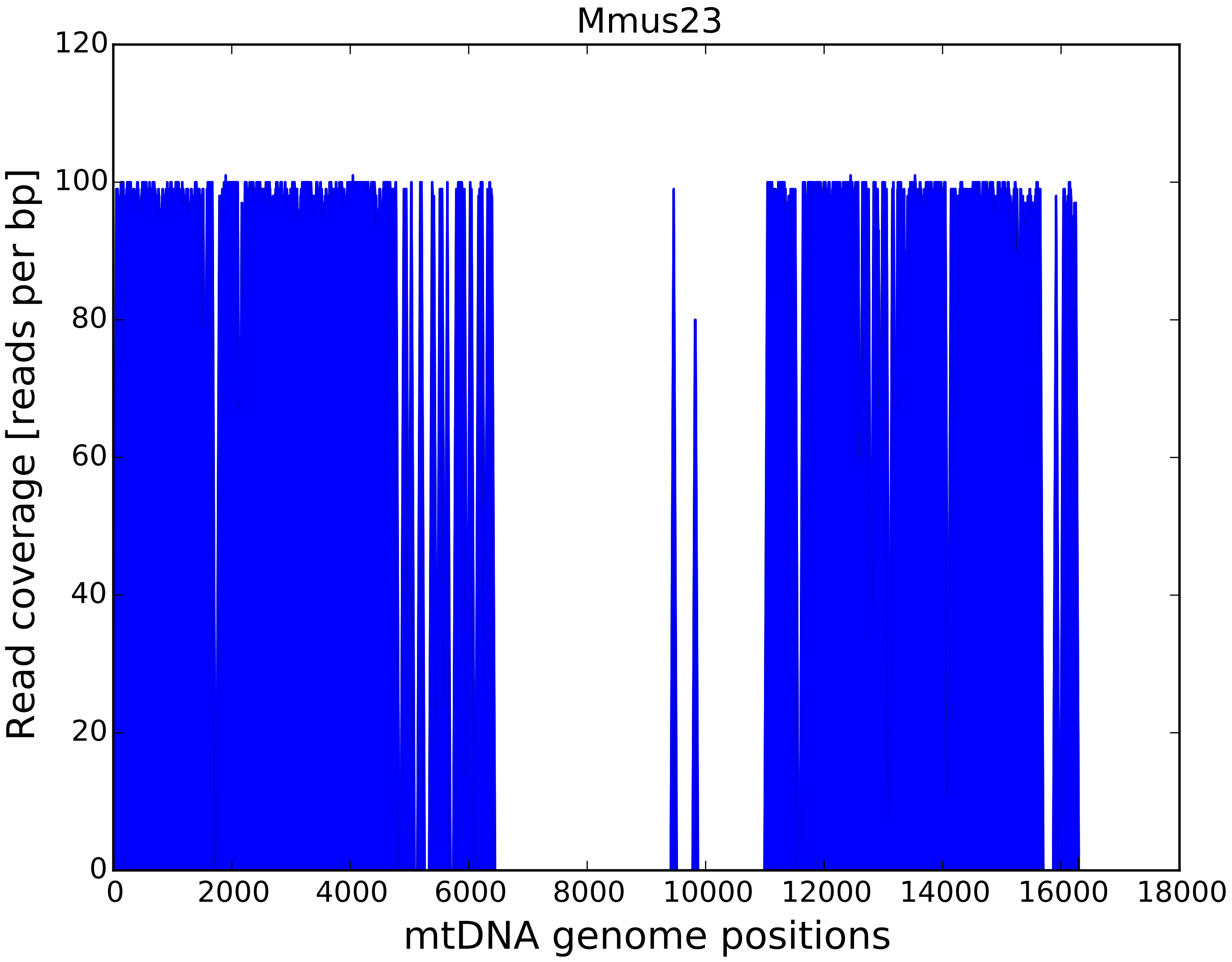

Mmus23
Read coverage [reads per bp]
mtDNA genome positions
0
20
40
60
80
100
120
0
2000
4000
6000
8000
10000
12000
14000
16000
18000

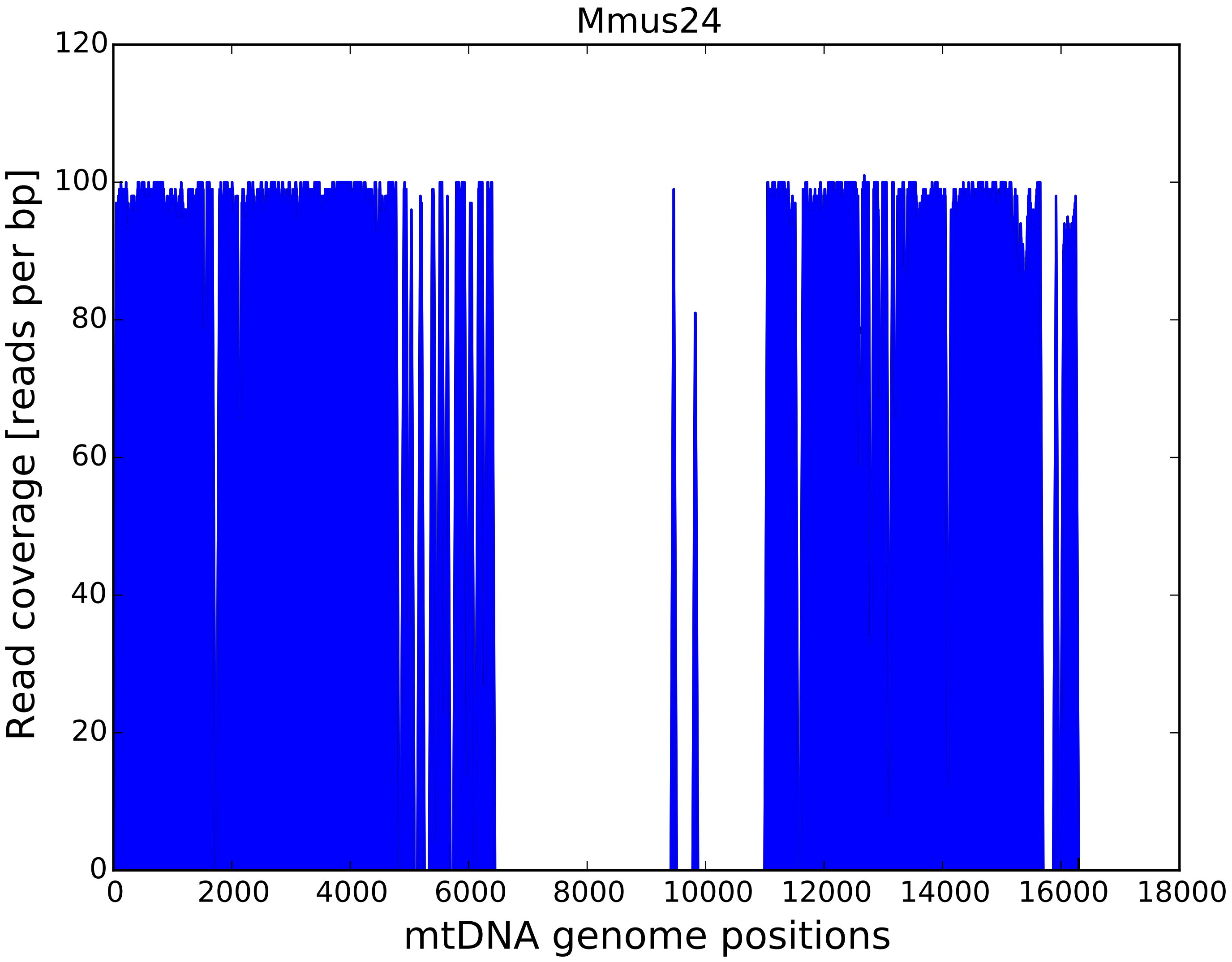

Mmus24
Read coverage [reads per bp]
mtDNA genome positions
0
20
40
60
80
100
120
0
2000
4000
6000
8000
10000
12000
14000
16000
18000

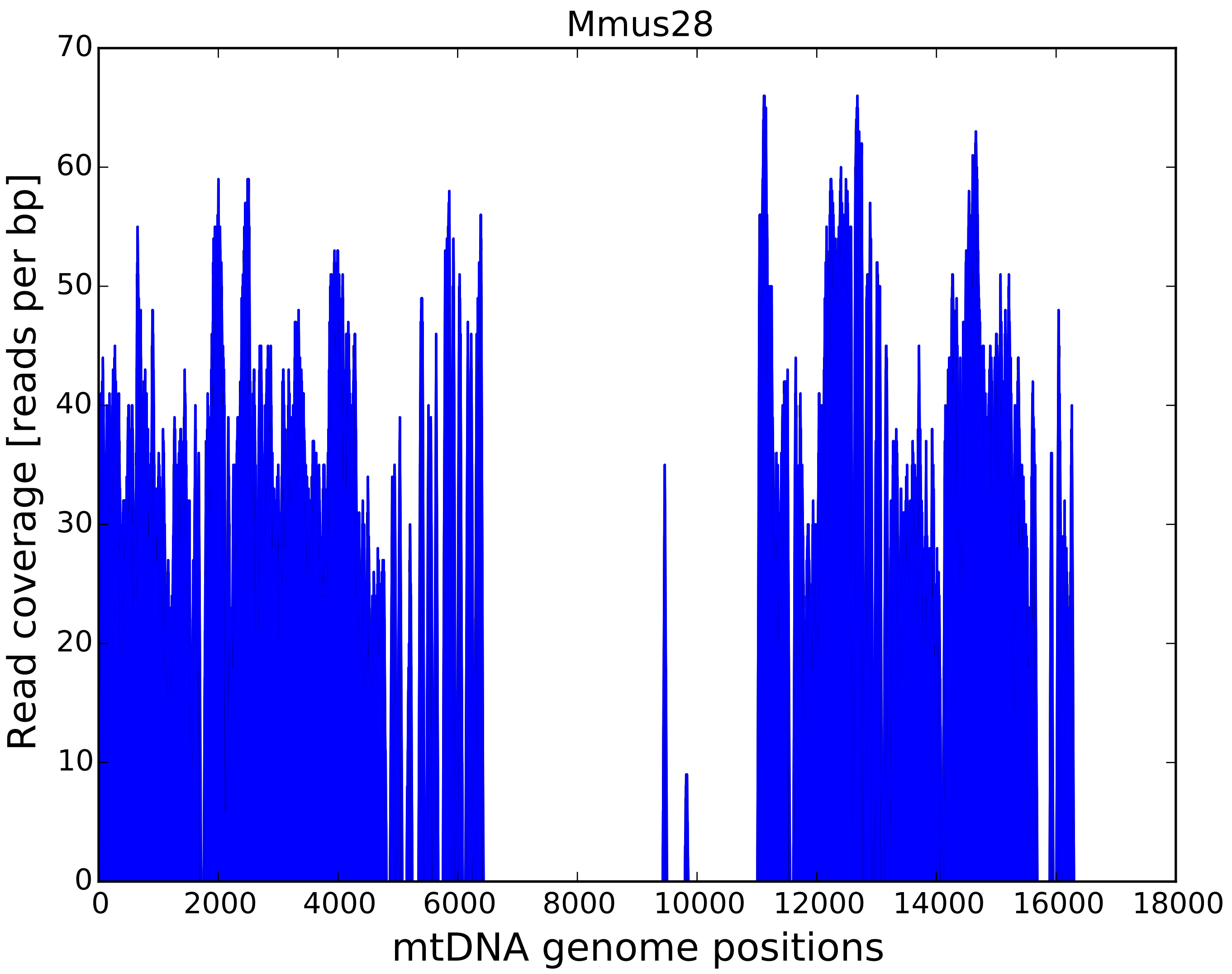
Mmus28
Read coverage [reads per bp]
mtDNA genome positions
0
10
20
30
40
50
60
70
0
2000
4000
6000
8000
10000
12000
14000
16000
18000

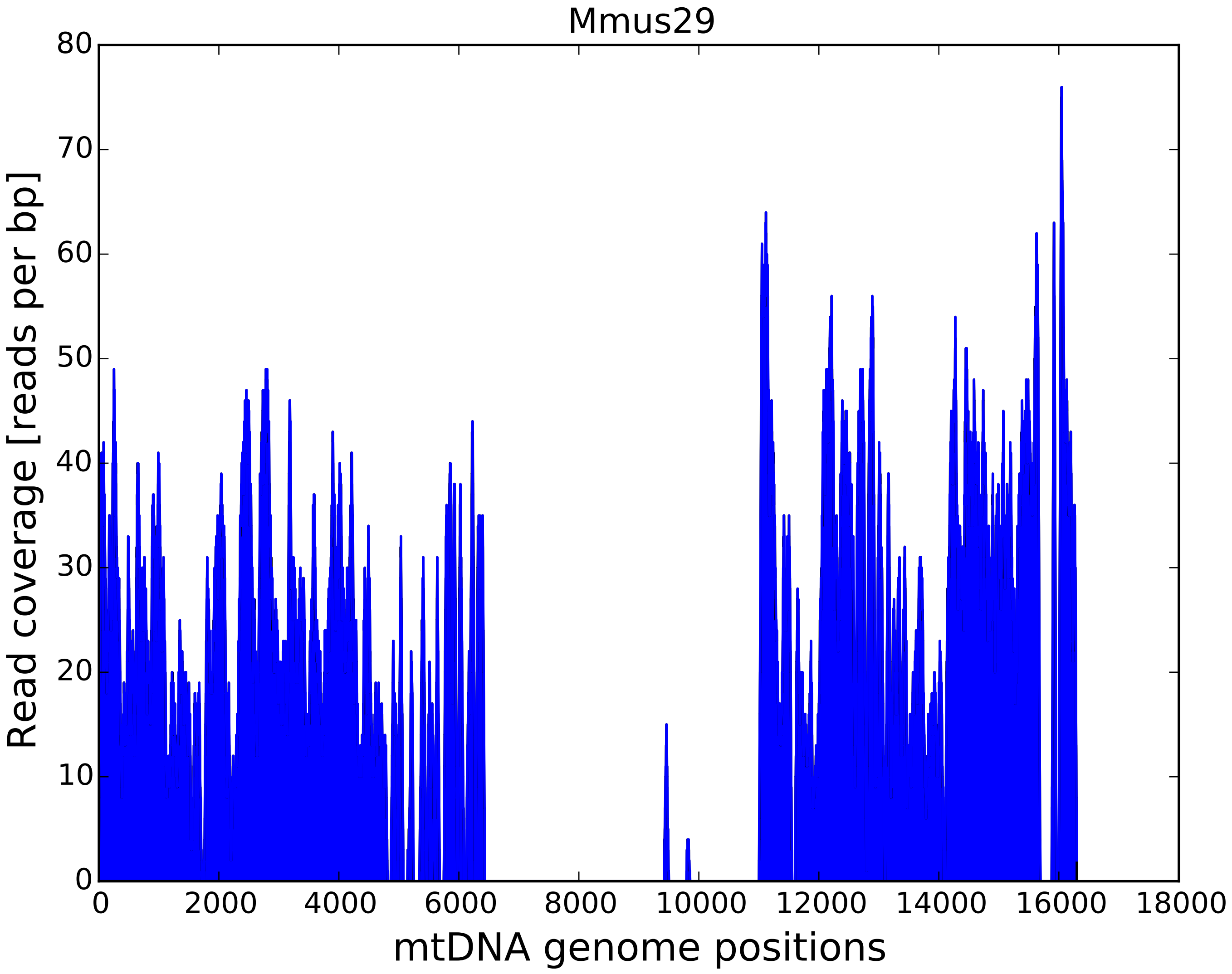

Mmus29
Read coverage [reads per bp]
mtDNA genome positions
0
10
20
30
40
50
60
70
80
0
2000
4000
6000
8000
10000
12000
14000
16000
18000

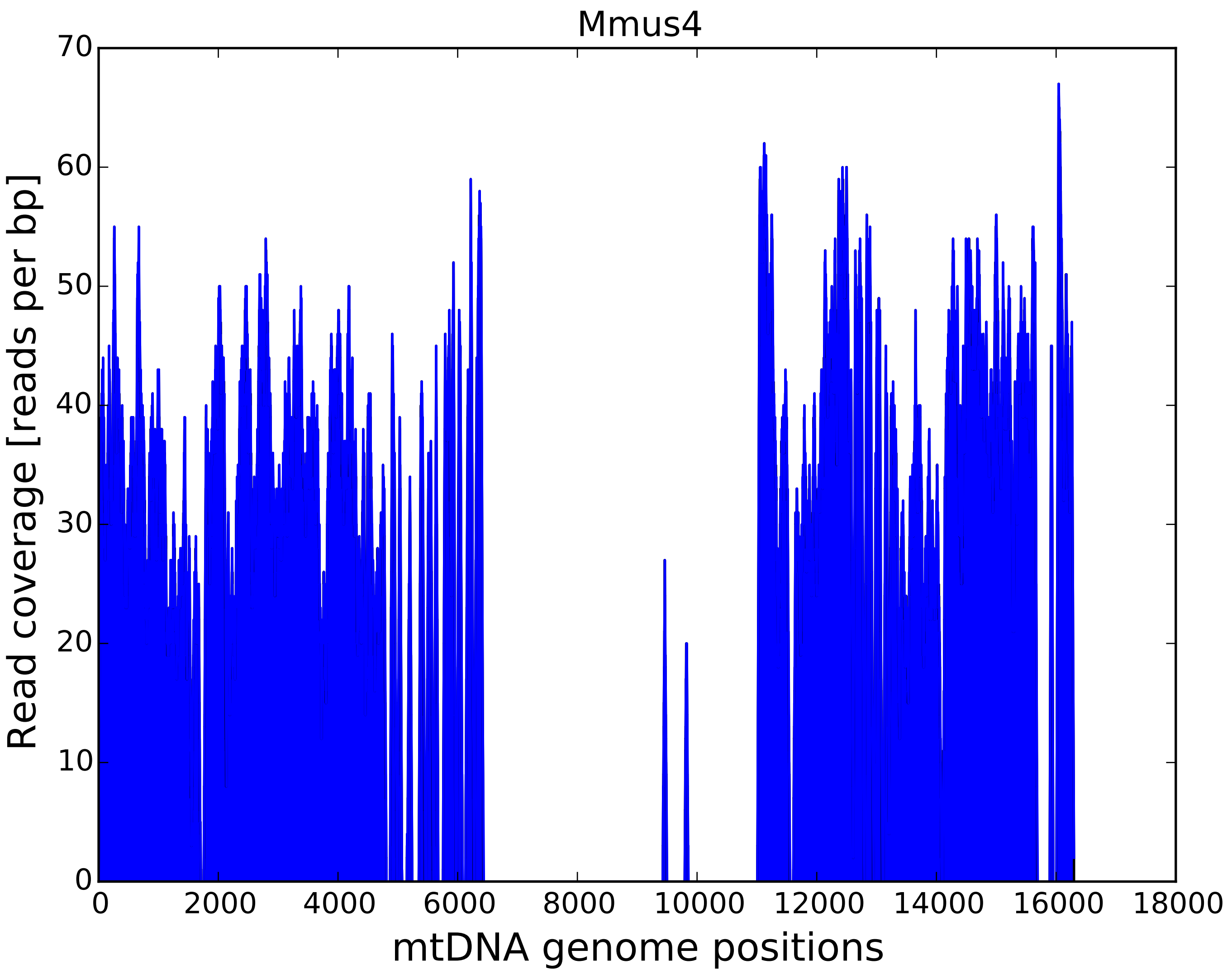

Mmus4
Read coverage [reads per bp]
mtDNA genome positions
0
10
20
30
40
50
60
70
0
2000
4000
6000
8000
10000
12000
14000
16000
18000

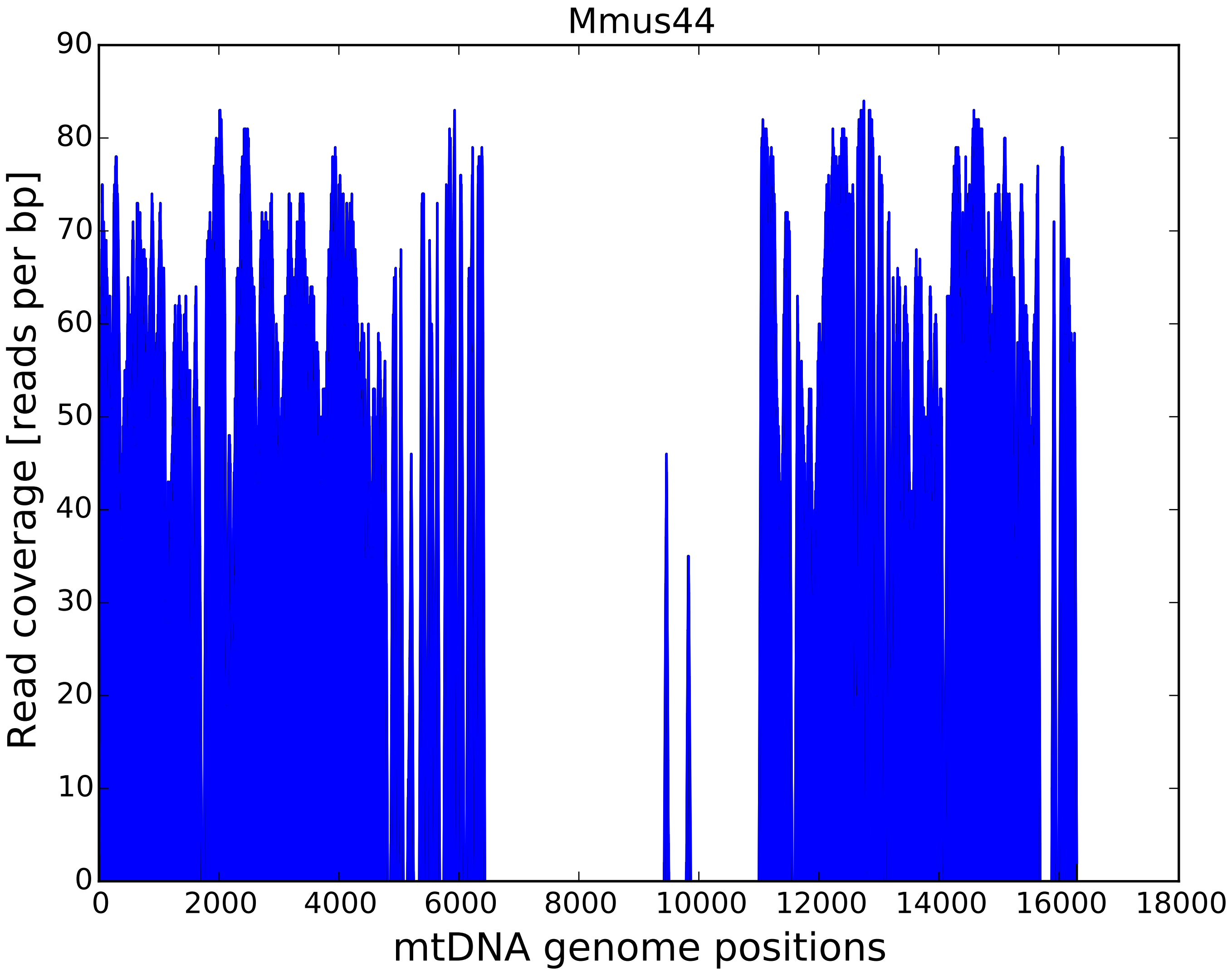

Mmus44
Read coverage [reads per bp]
mtDNA genome positions
0
10
20
30
40
50
60
70
80
90
0
2000
4000
6000
8000
10000
12000
14000
16000
18000

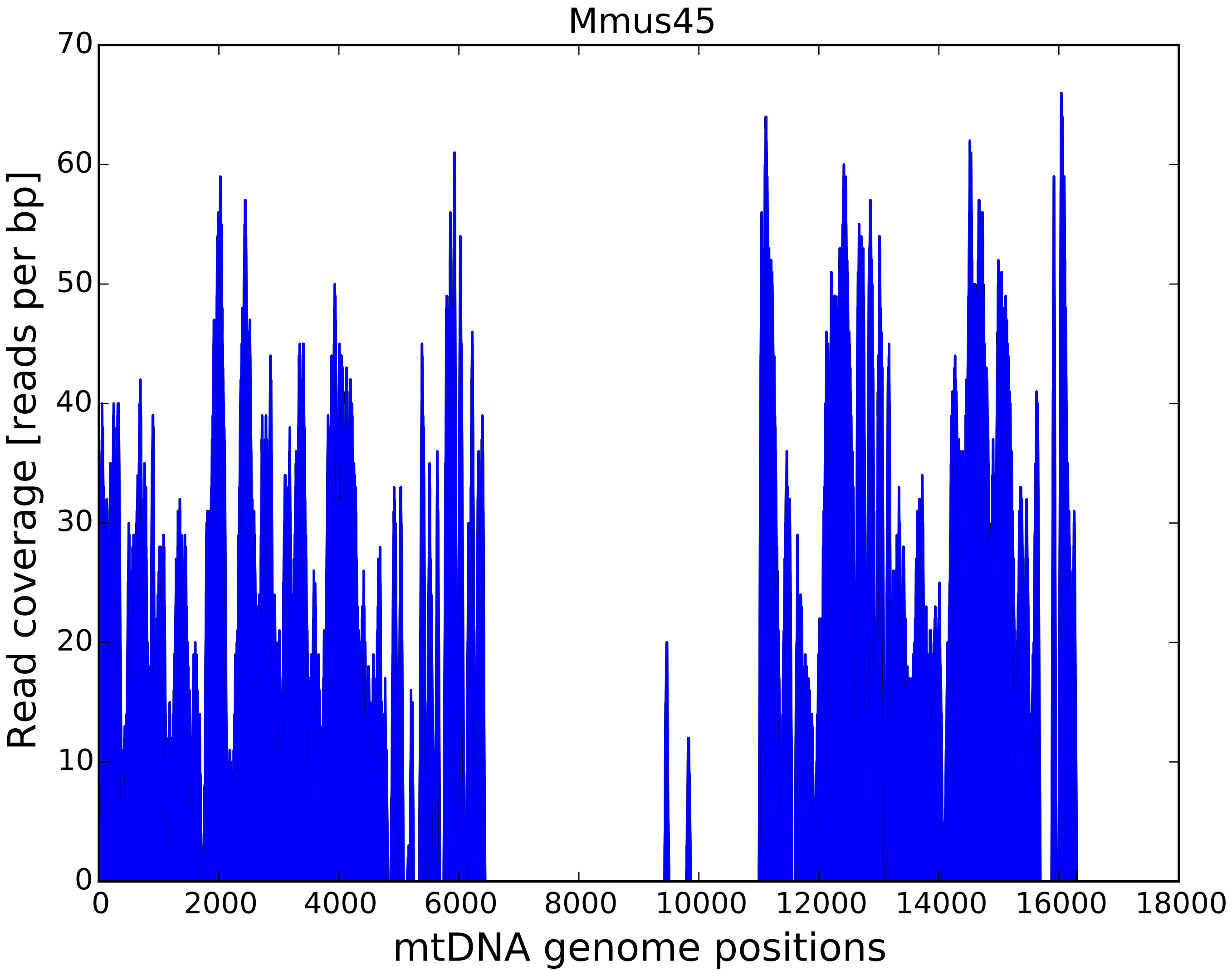

Mmus45
Read coverage [reads per bp]
mtDNA genome positions
0
10
20
30
40
50
60
70
0
2000
4000
6000
8000
10000
12000
14000
16000
18000

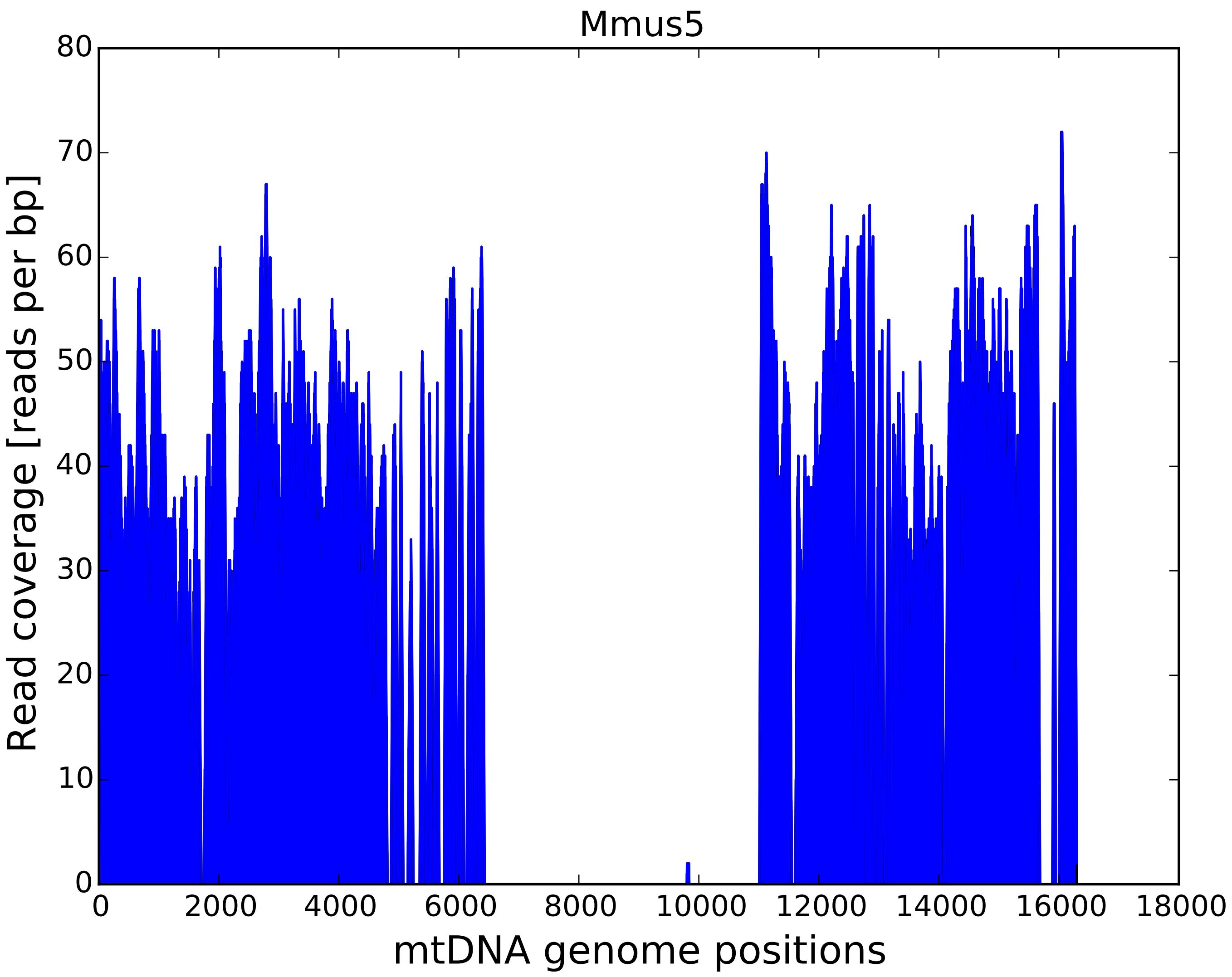
Mmus5
Read coverage [reads per bp]
mtDNA genome positions
0
10
20
30
40
50
60
70
80
0
2000
4000
6000
8000
10000
12000
14000
16000
18000

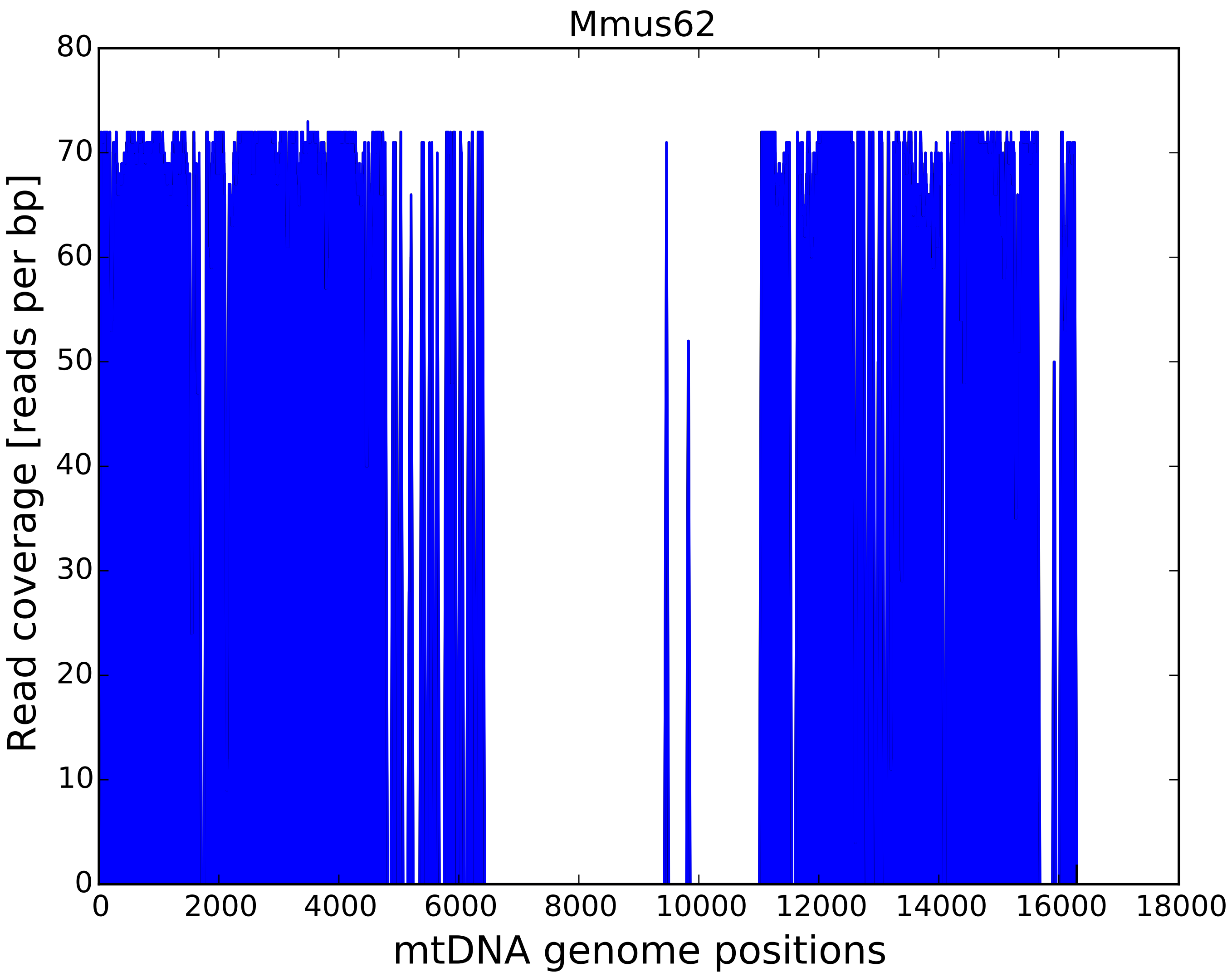

Mmus62
Read coverage [reads per bp]
mtDNA genome positions
0
10
20
30
40
50
60
70
80
0
2000
4000
6000
8000
10000
12000
14000
16000
18000

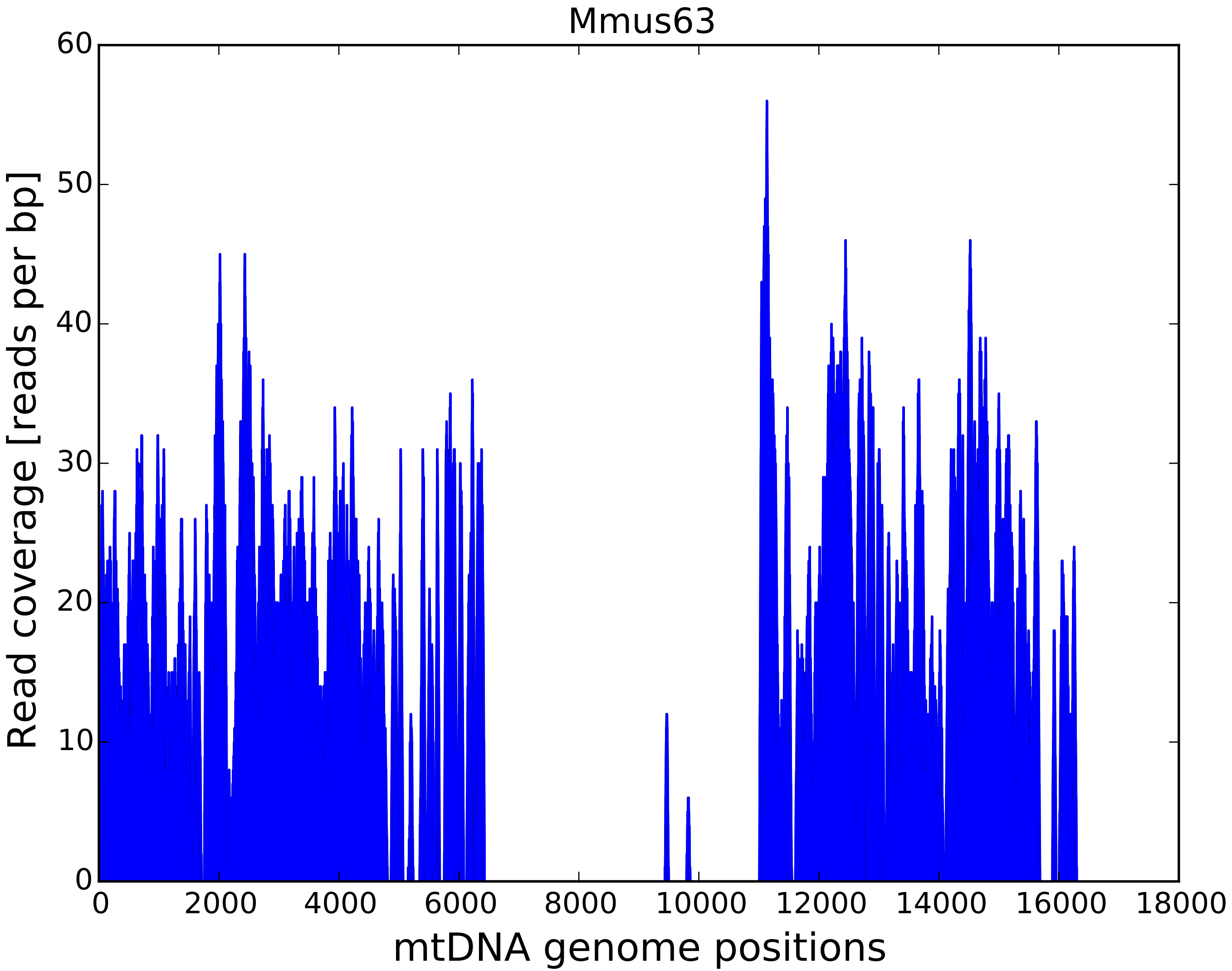

Mmus63
Read coverage [reads per bp]
mtDNA genome positions
0
10
20
30
40
50
60
2000
4000
6000
8000
10000
12000
14000
16000
18000

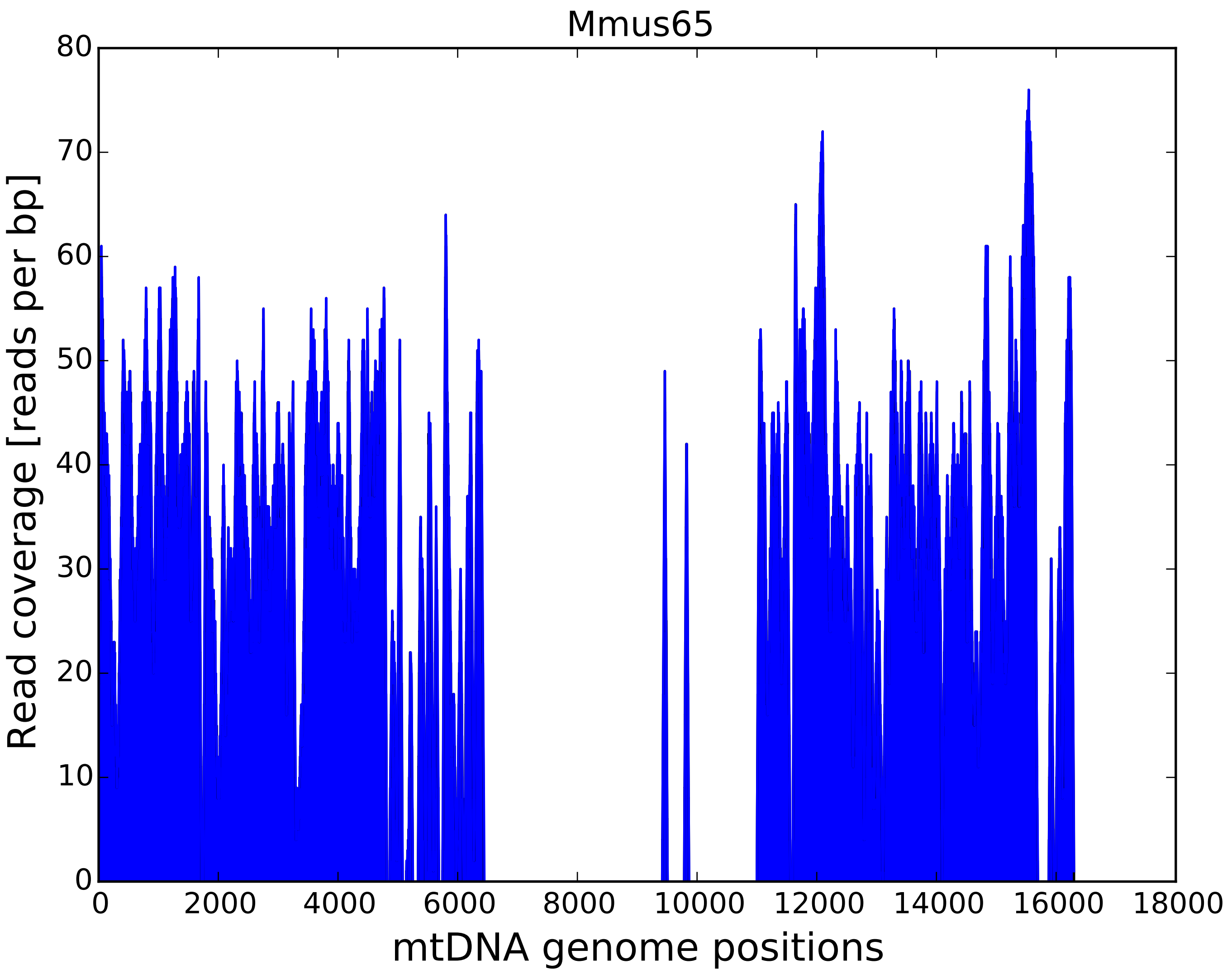

Mmus65
Read coverage [reads per bp]
mtDNA genome positions
0
10
20
30
40
50
60
70
80
0
2000
4000
6000
8000
10000
12000
14000
16000
18000

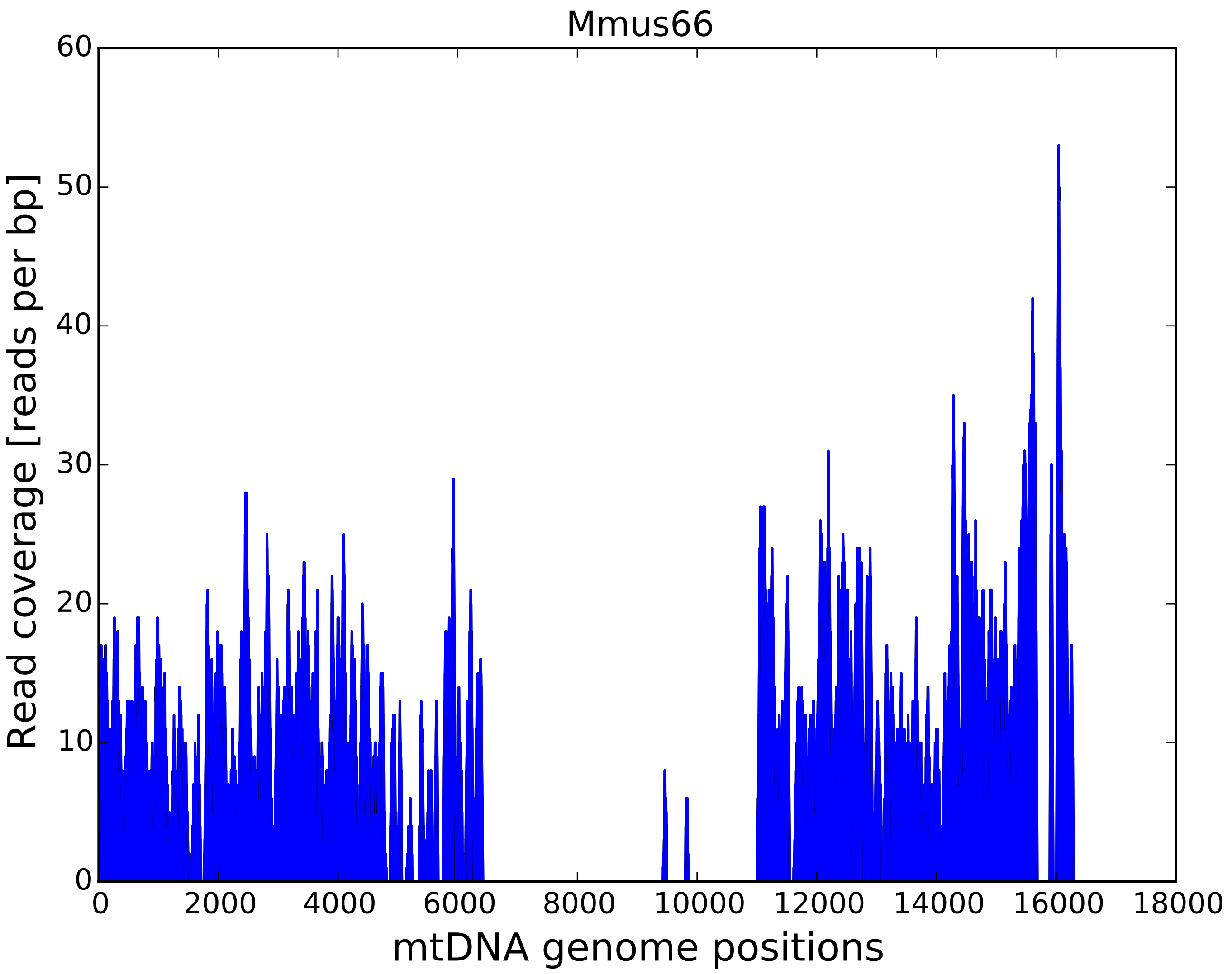

Mmus66
Read coverage [reads per bp]
mtDNA genome positions
0
10
20
30
40
50
60
0
2000
4000
6000
8000
10000
12000
14000
16000
18000

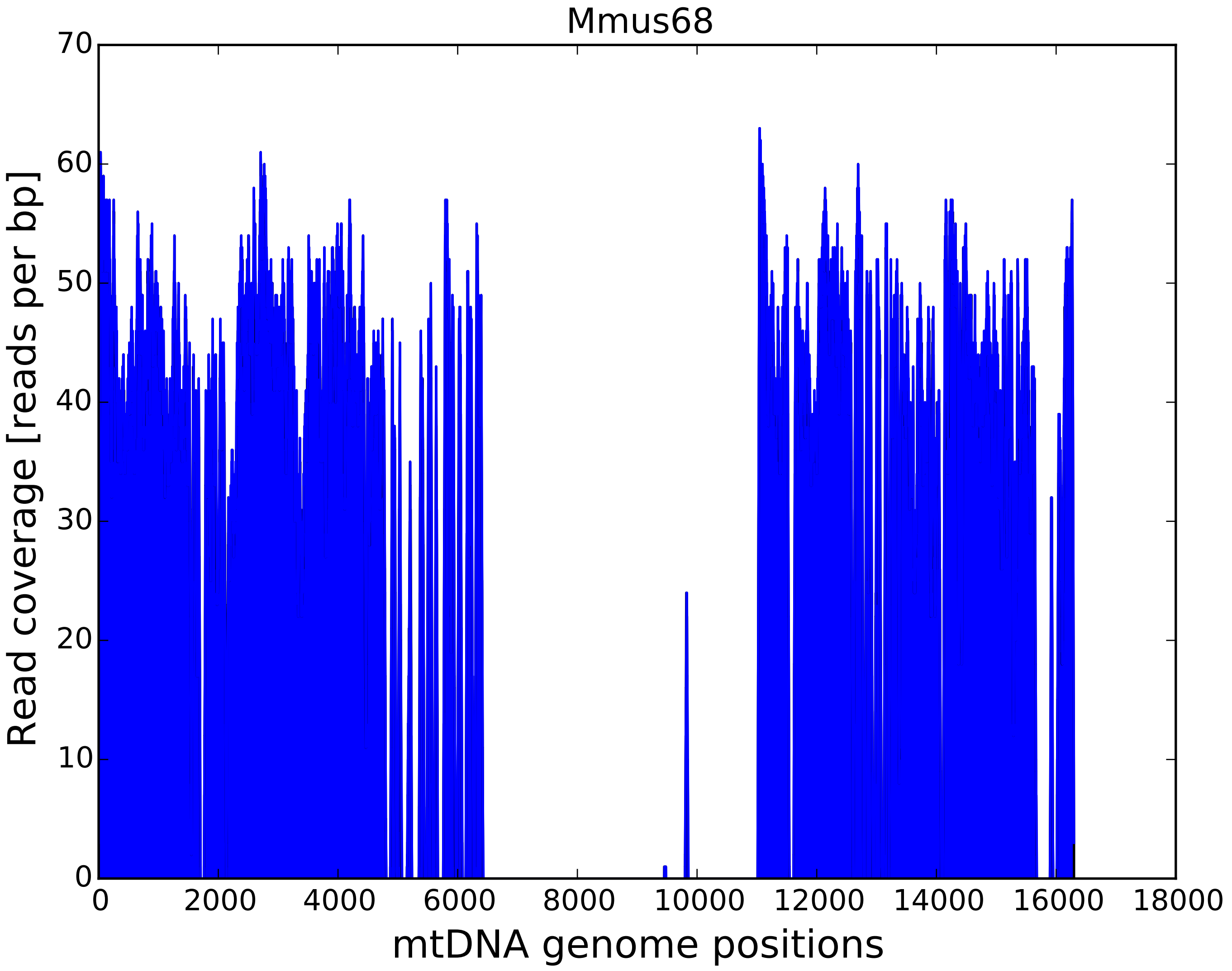

Mmus68
Read coverage [reads per bp]
mtDNA genome positions
0
10
20
30
40
50
60
70
0
2000
4000
6000
8000
10000
12000
14000
16000
18000

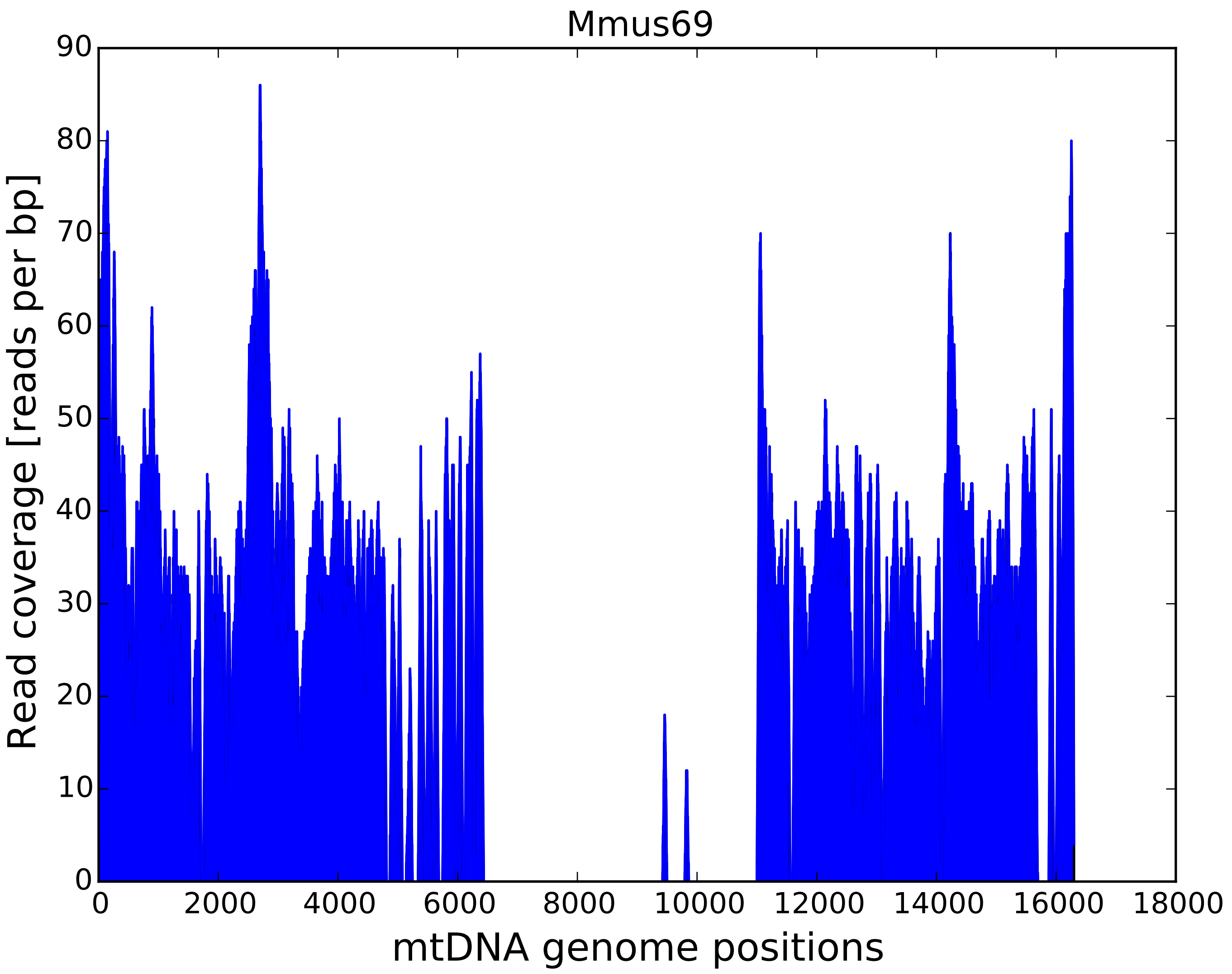

Mmus69
Read coverage [reads per bp]
mtDNA genome positions
0
10
20
30
40
50
60
70
80
90
0
2000
4000
6000
8000
10000
12000
14000
16000
18000

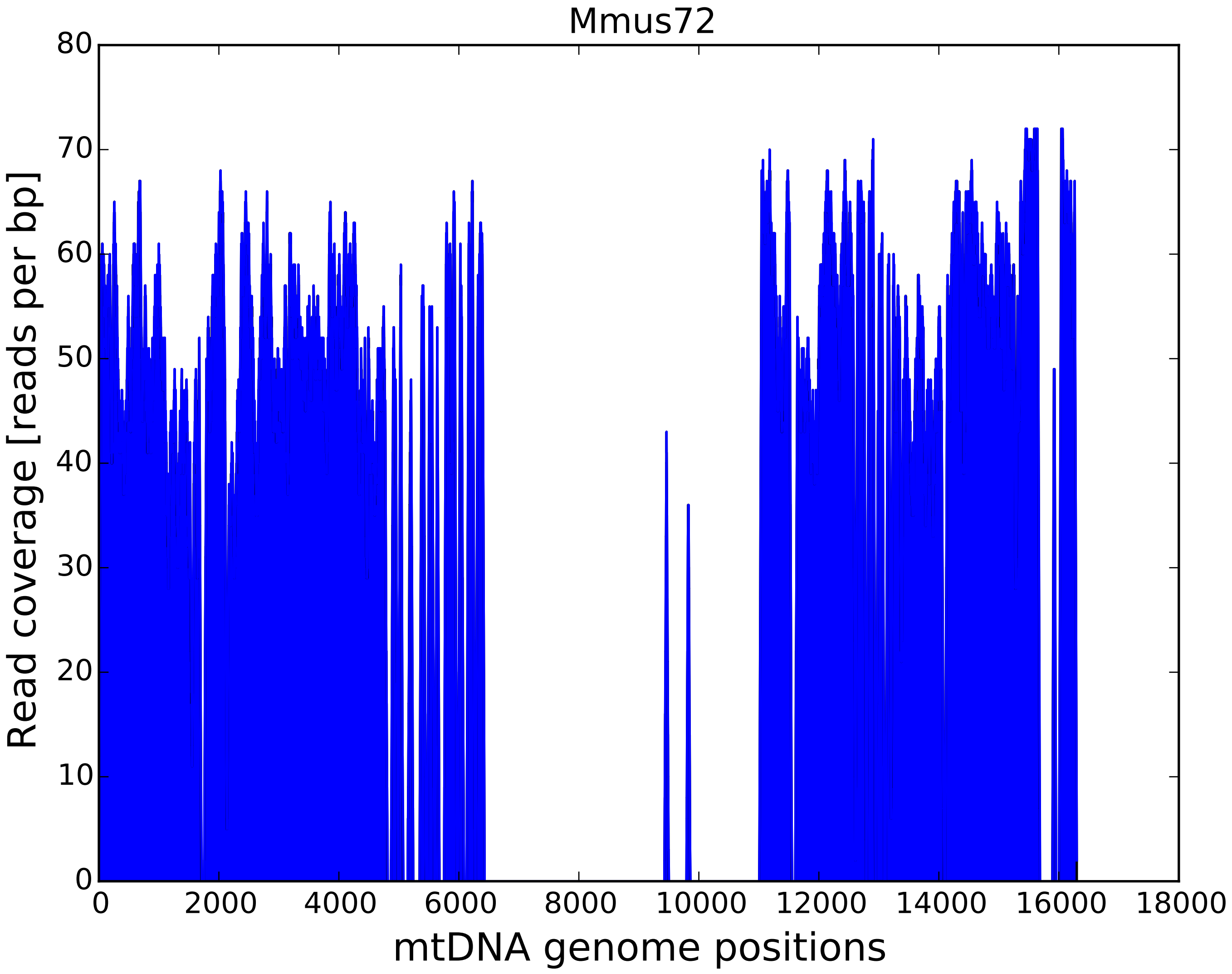

Mmus72
Read coverage [reads per bp]
mtDNA genome positions
0
10
20
30
40
50
60
70
80
0
2000
4000
6000
8000
10000
12000
14000
16000
18000

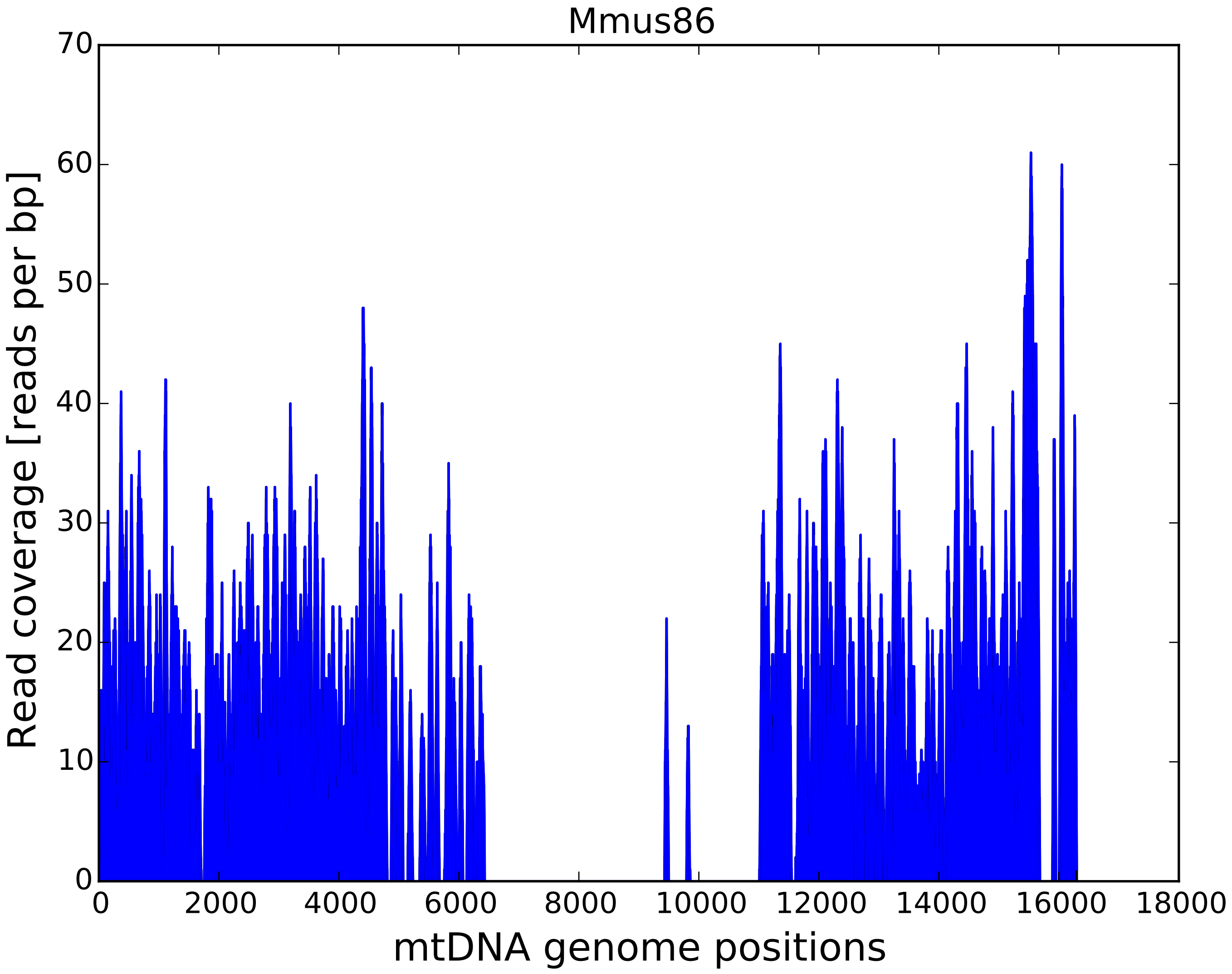
Mmus86
Read coverage [reads per bp]
mtDNA genome positions
0
10
20
30
40
50
60
70
0
2000
4000
6000
8000
10000
12000
14000
16000
18000

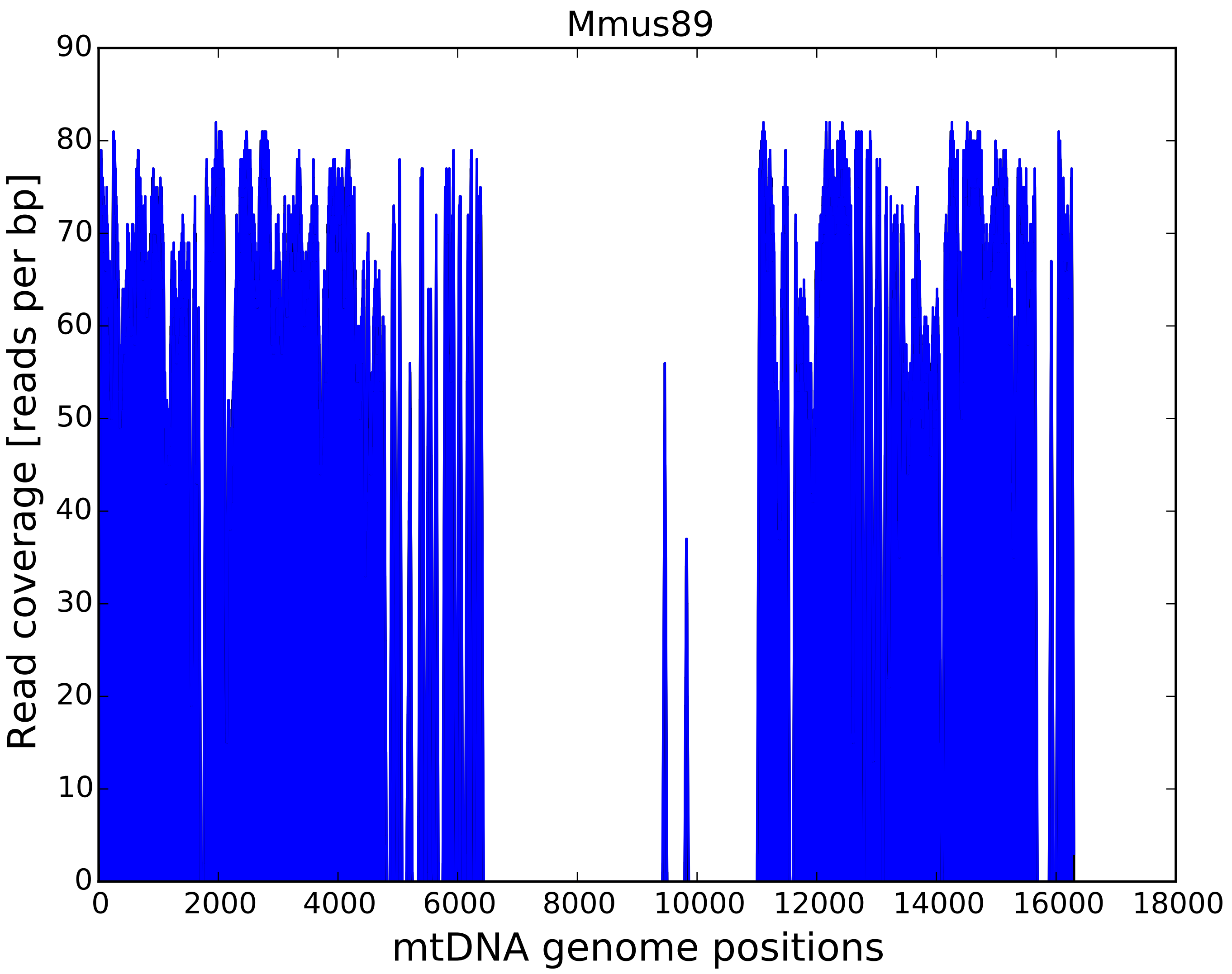

Mmus89
Read coverage [reads per bp]
mtDNA genome positions
0
10
20
30
40
50
60
70
80
90
0
2000
4000
6000
8000
10000
12000
14000
16000
18000

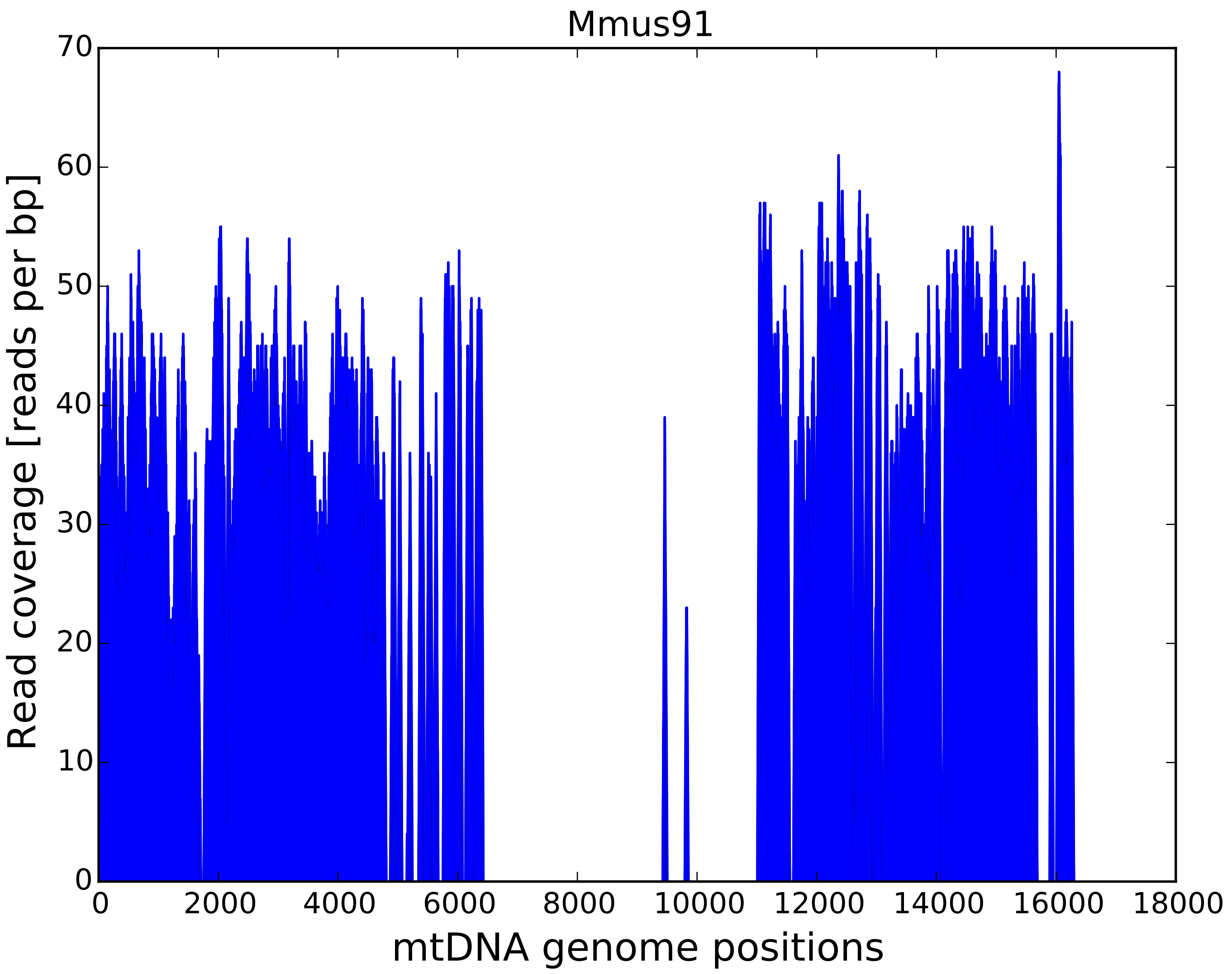
Mmus91
Read coverage [reads per bp]
mtDNA genome positions
0
10
20
30
40
50
60
70
0
2000
4000
6000
8000
10000
12000
14000
16000
18000

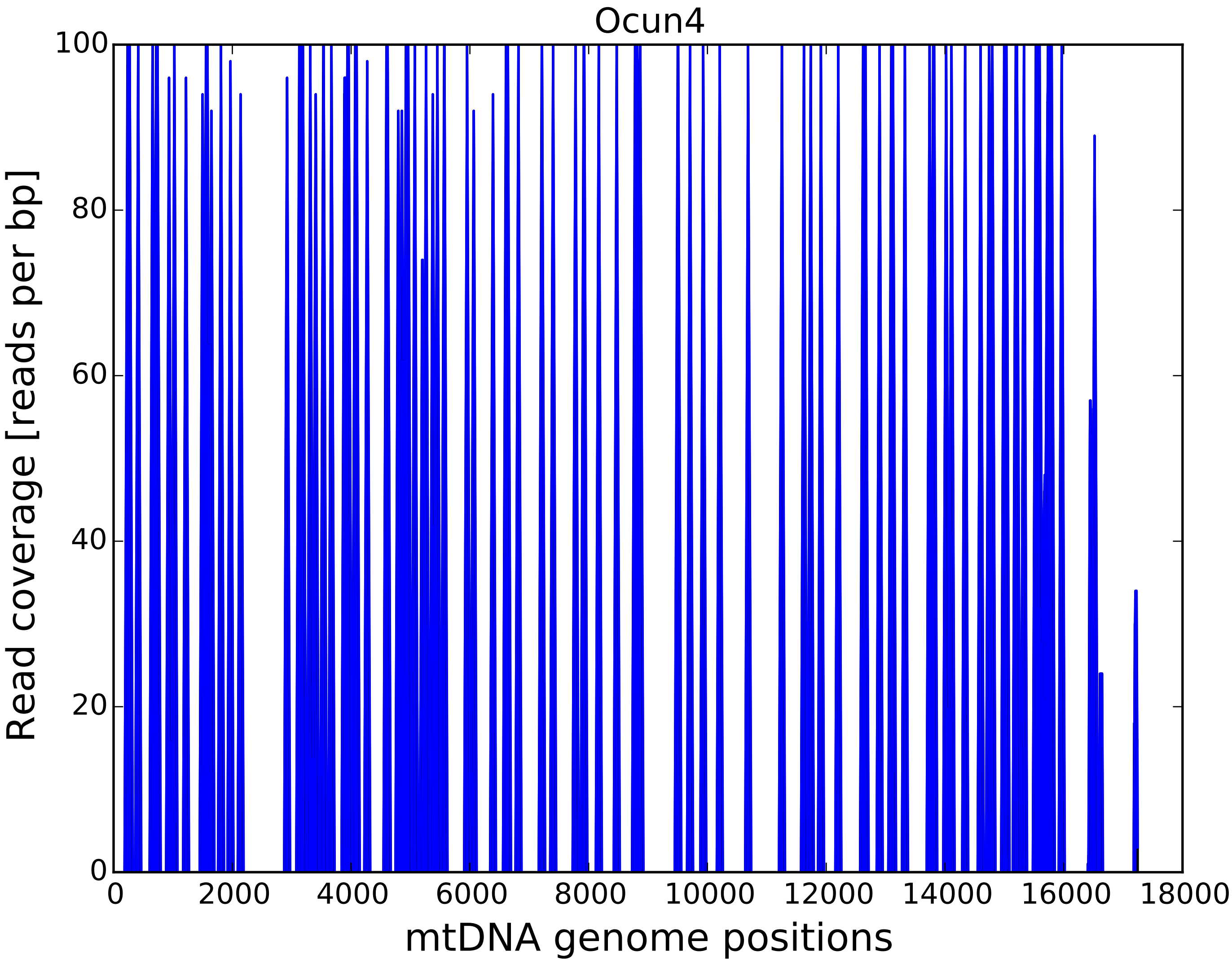
Ocun4
Read coverage [reads per bp]
mtDNA genome positions
0
20
40
60
80
100
0
2000
4000
6000
8000
10000
12000
14000
16000
18000

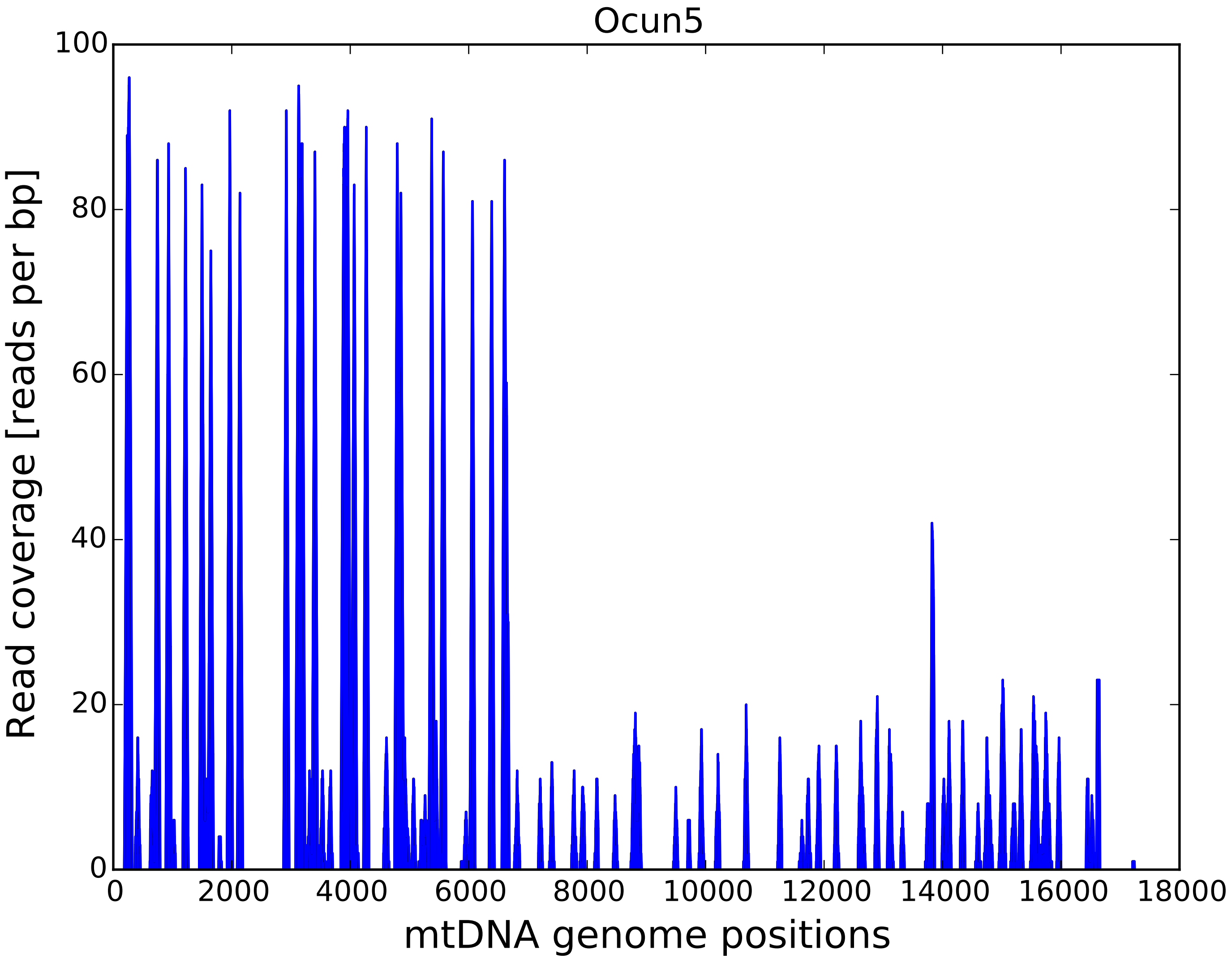

Ocun5
Read coverage [reads per bp]
mtDNA genome positions
0
20
40
60
80
100
0
2000
4000
6000
8000
10000
12000
14000
16000
18000

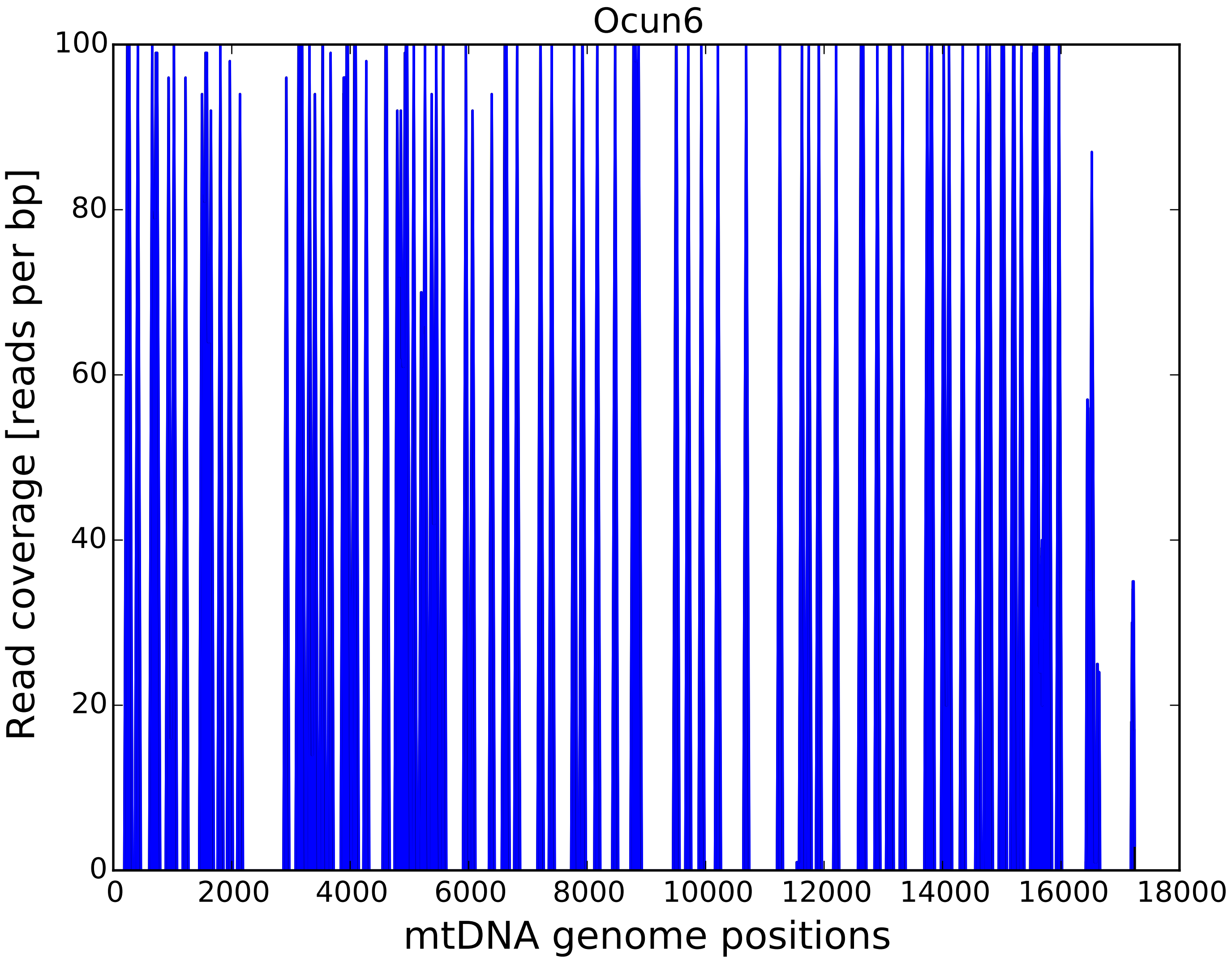
Ocun6
Read coverage [reads per bp]
mtDNA genome positions
0
20
40
60
80
100
0
2000
4000
6000
8000
10000
12000
14000
16000
18000

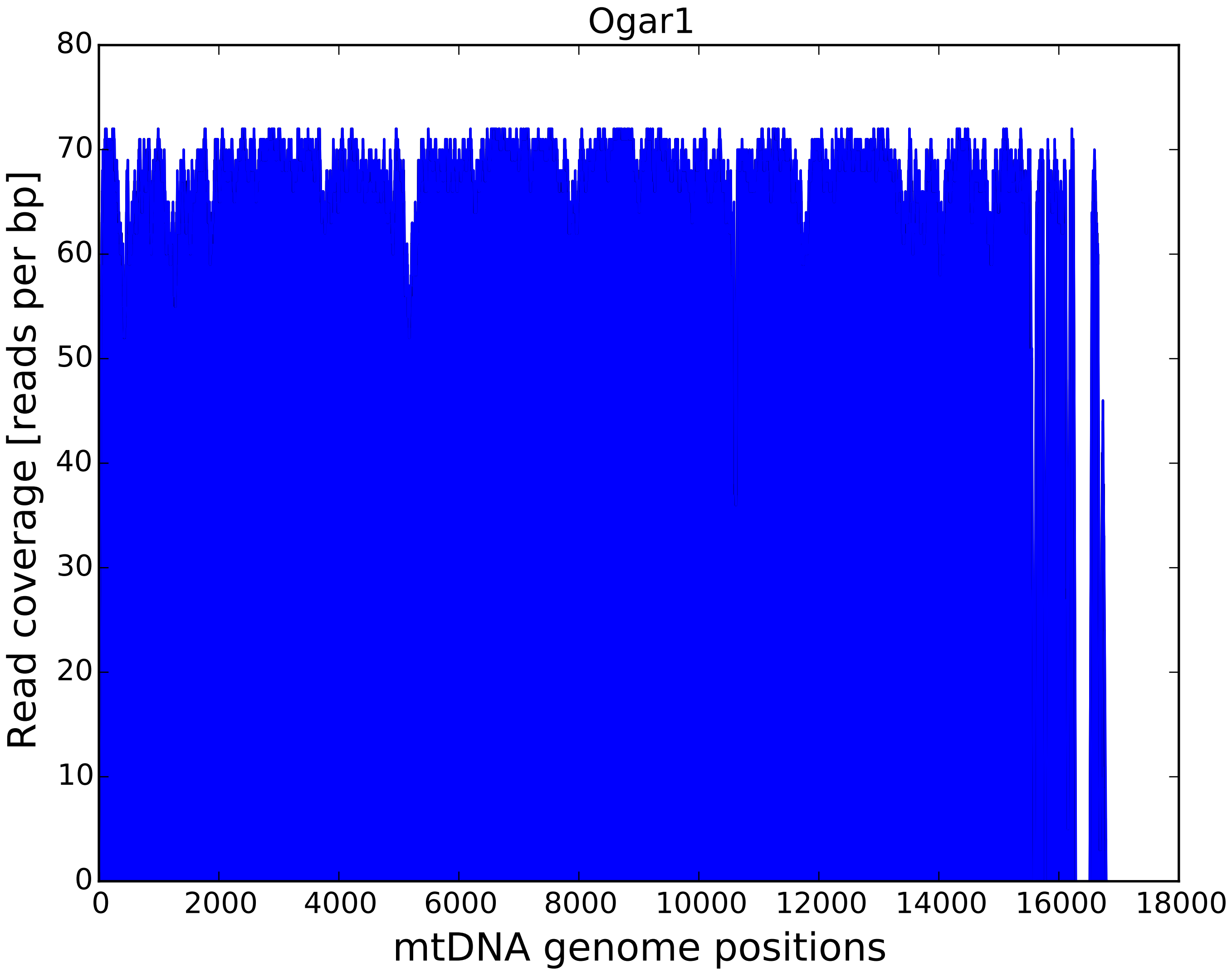

Ogar1
Read coverage [reads per bp]
mtDNA genome positions
0
10
20
30
40
50
60
70
80
0
2000
4000
6000
8000
10000
12000
14000
16000
18000

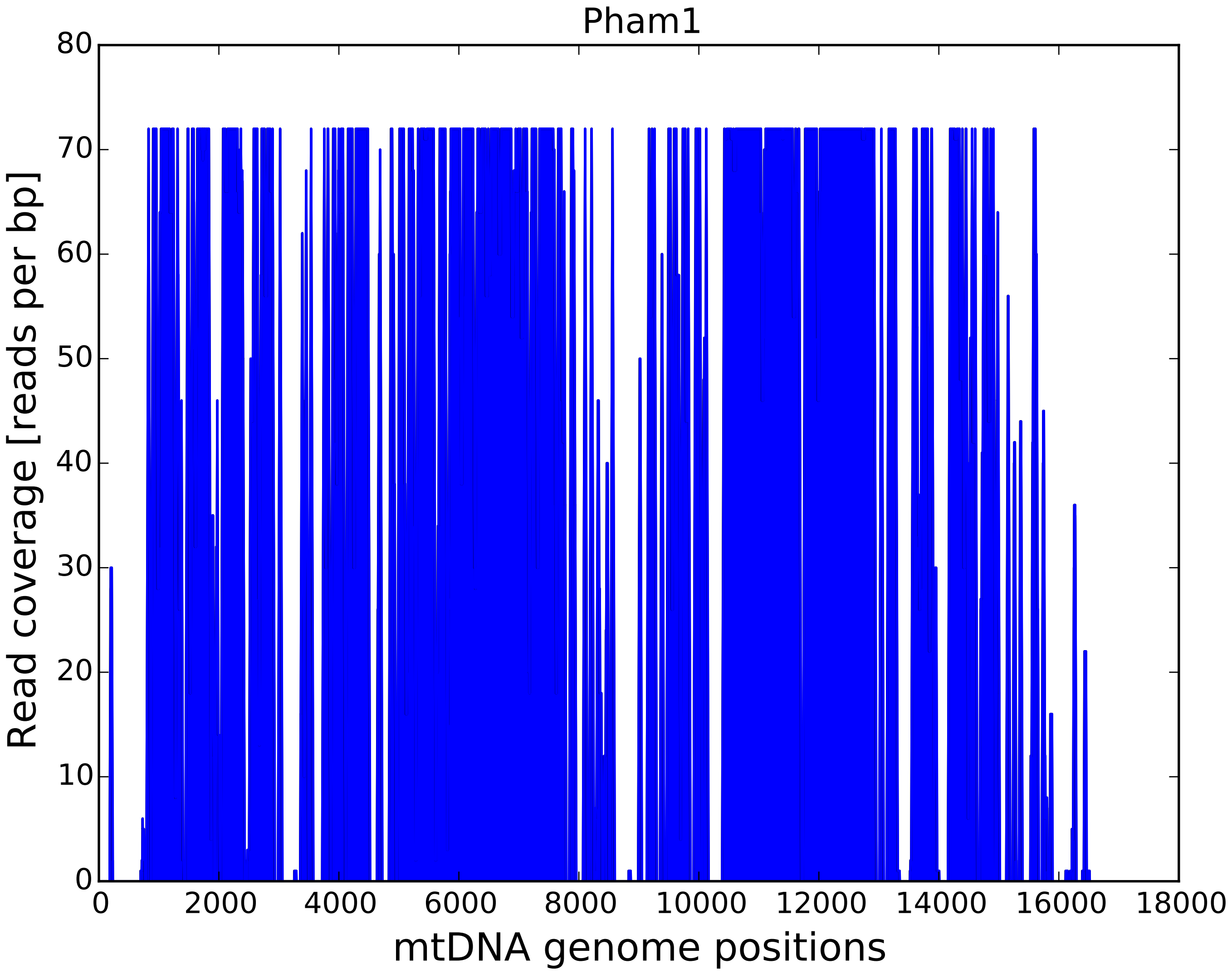
Pham1
Read coverage [reads per bp]
mtDNA genome positions
0
10
20
30
40
50
60
70
80
0
2000
4000
6000
8000
10000
12000
14000
16000
18000

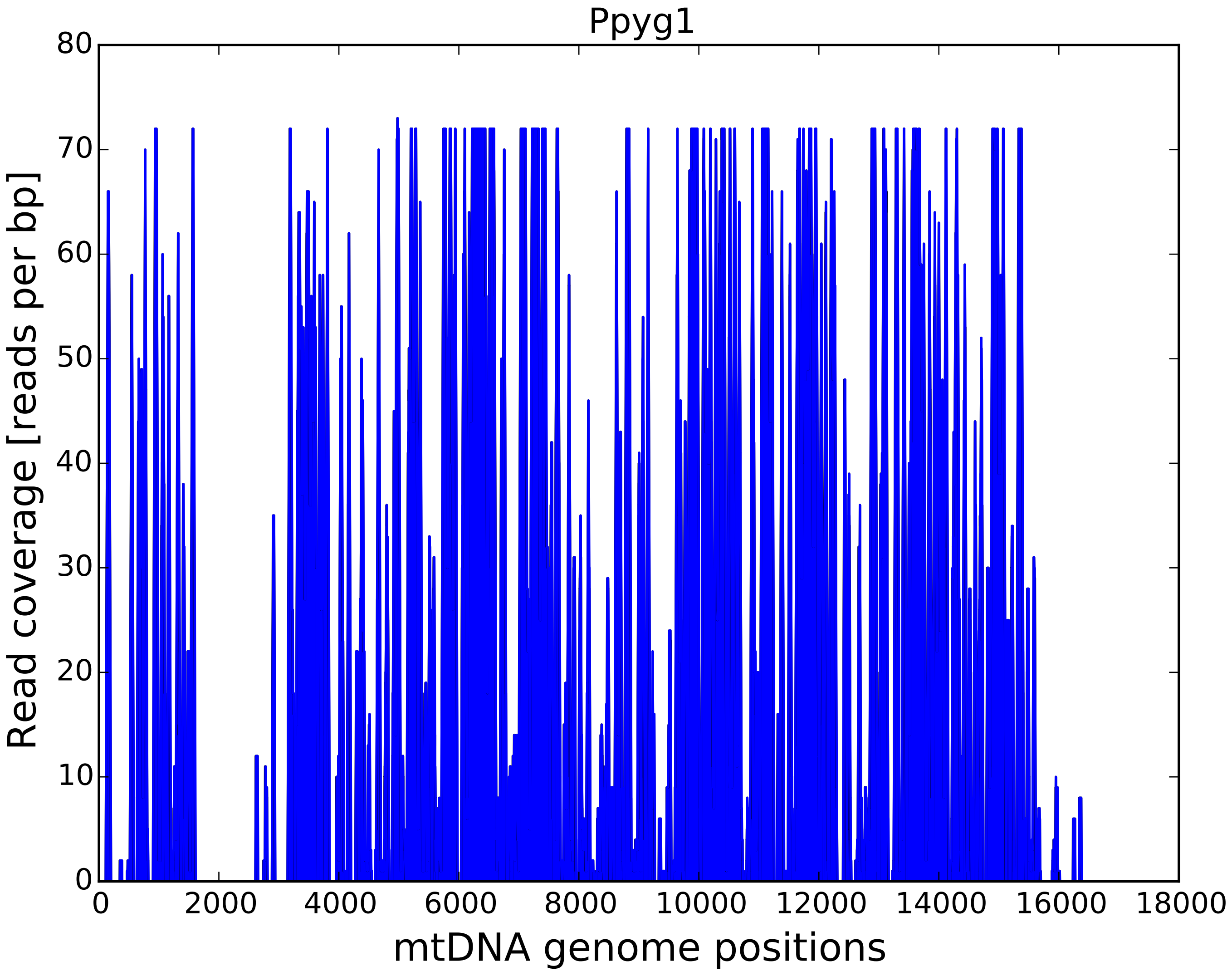

Ppyg1
Read coverage [reads per bp]
mtDNA genome positions
0
10
20
30
40
50
60
70
80
0
2000
4000
6000
8000
10000
12000
14000
16000
18000

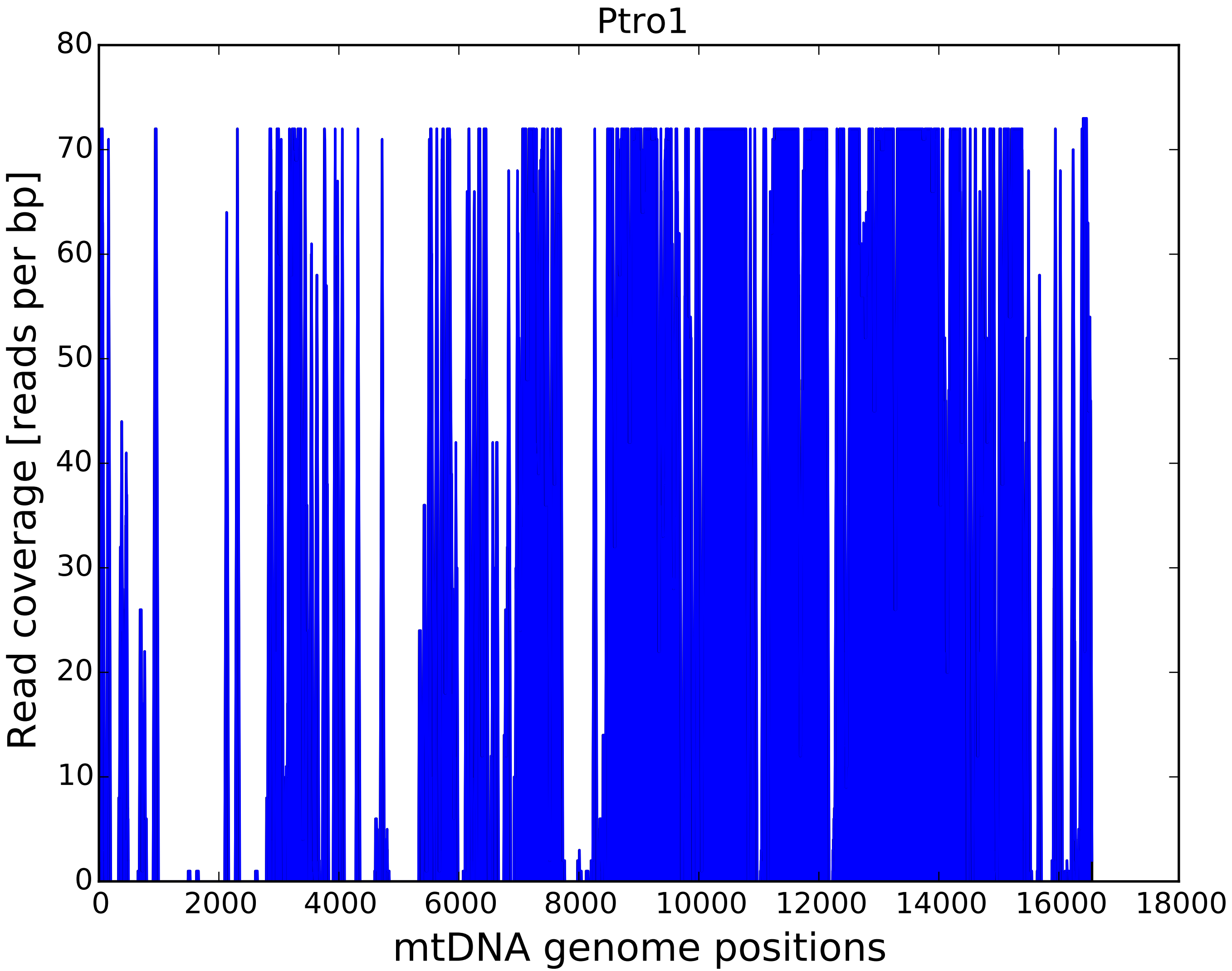

Ptro1
Read coverage [reads per bp]
mtDNA genome positions
0
10
20
30
40
50
60
70
80
0
2000
4000
6000
8000
10000
12000
14000
16000
18000

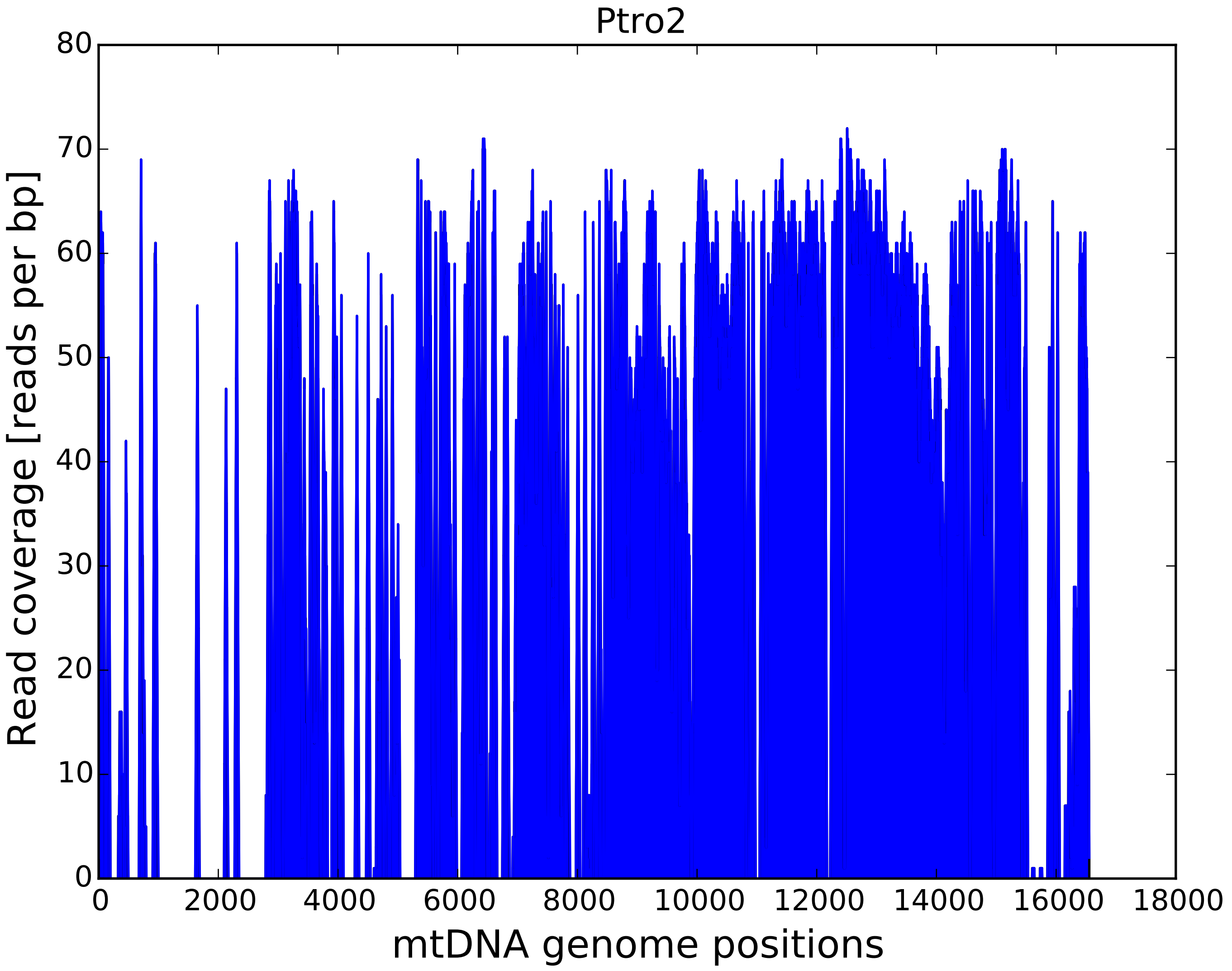

Ptro2
Read coverage [reads per bp]
mtDNA genome positions
0
10
20
30
40
50
60
70
80
0
2000
4000
6000
8000
10000
12000
14000
16000
18000

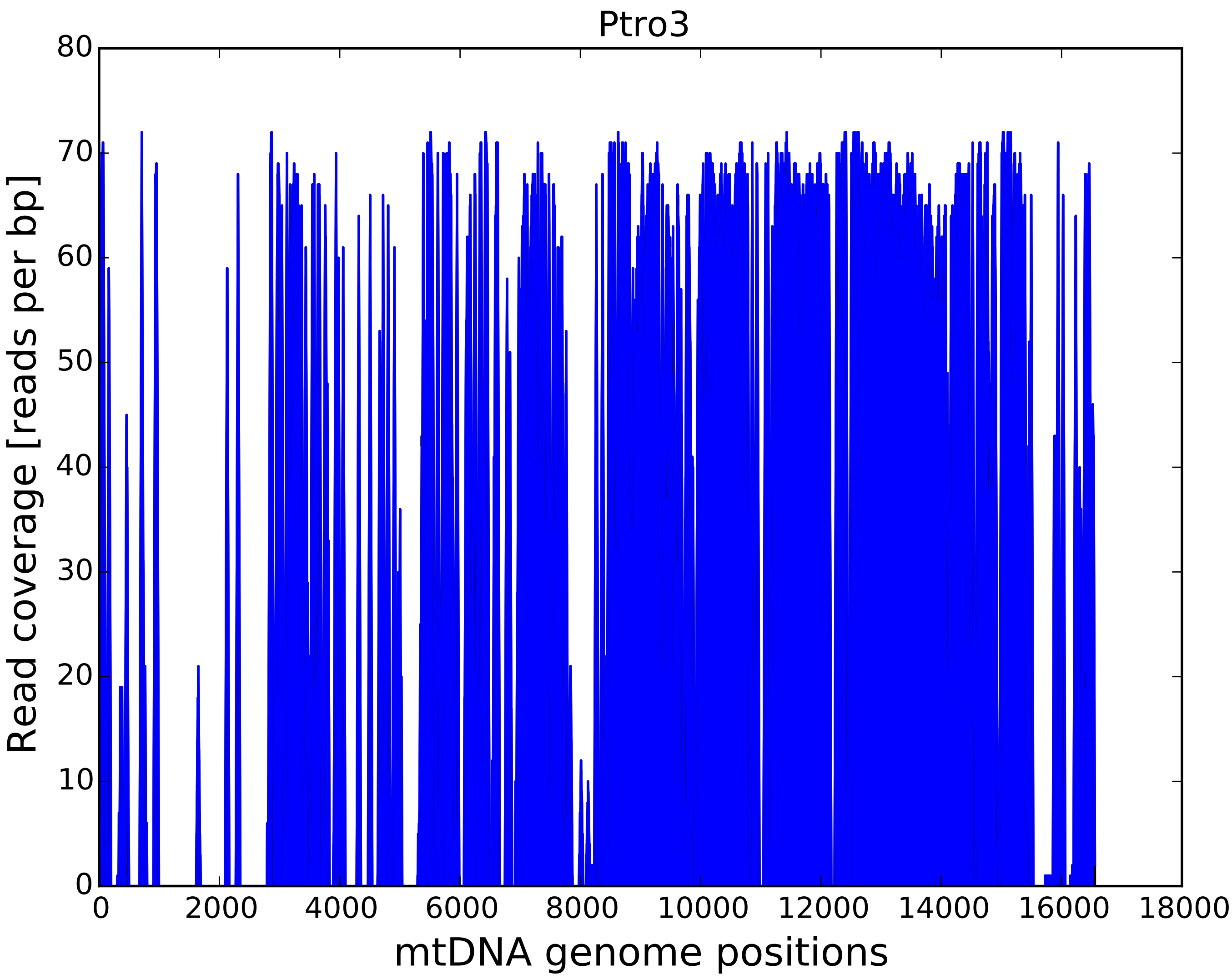
Ptro3
Read coverage [reads per bp]
mtDNA genome positions
0
10
20
30
40
50
60
70
80
2000
4000
6000
8000
10000
12000
14000
16000
18000

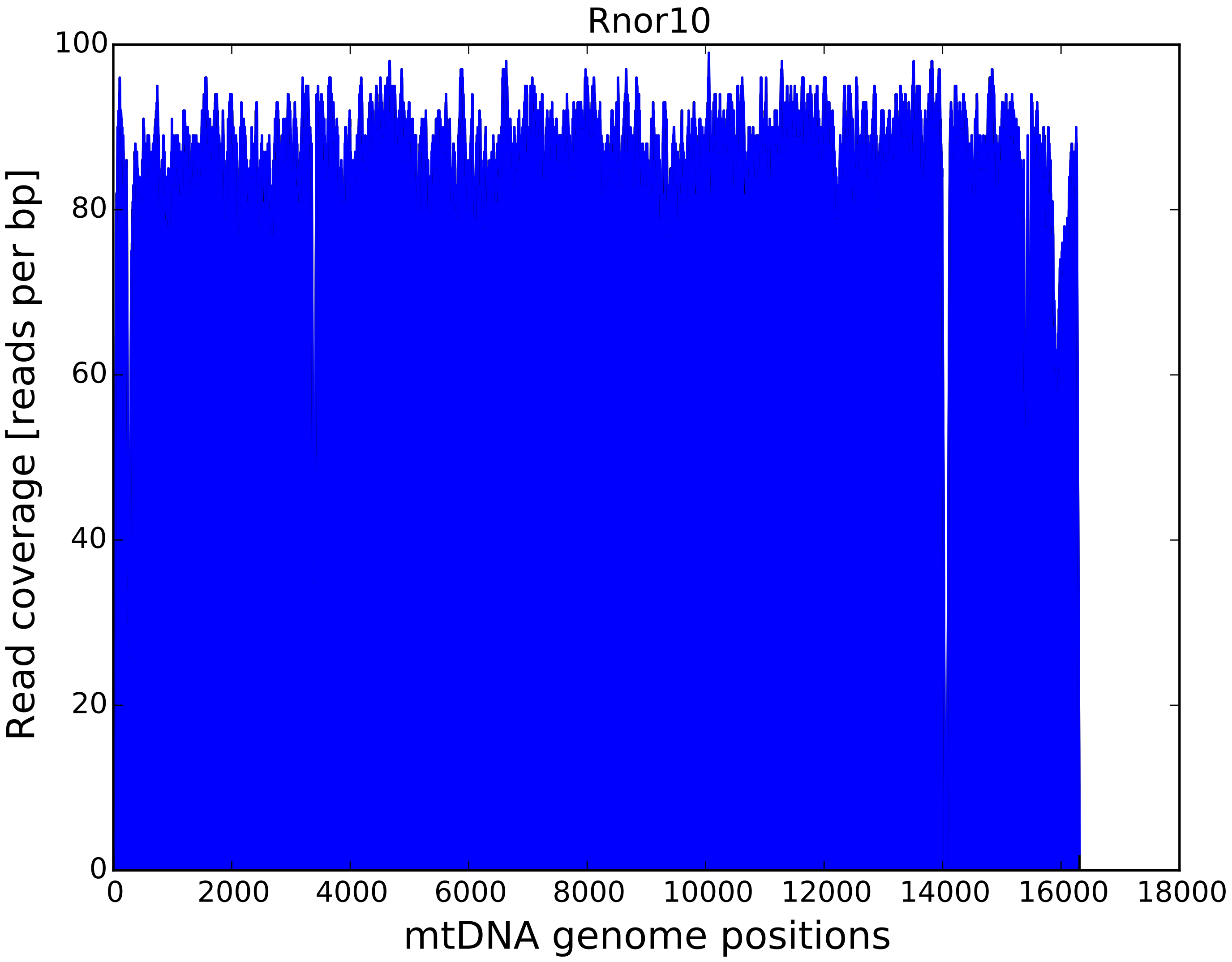

Rnor10
Read coverage [reads per bp]
mtDNA genome positions
0
20
40
60
80
100
0
2000
4000
6000
8000
10000
12000
14000
16000
18000

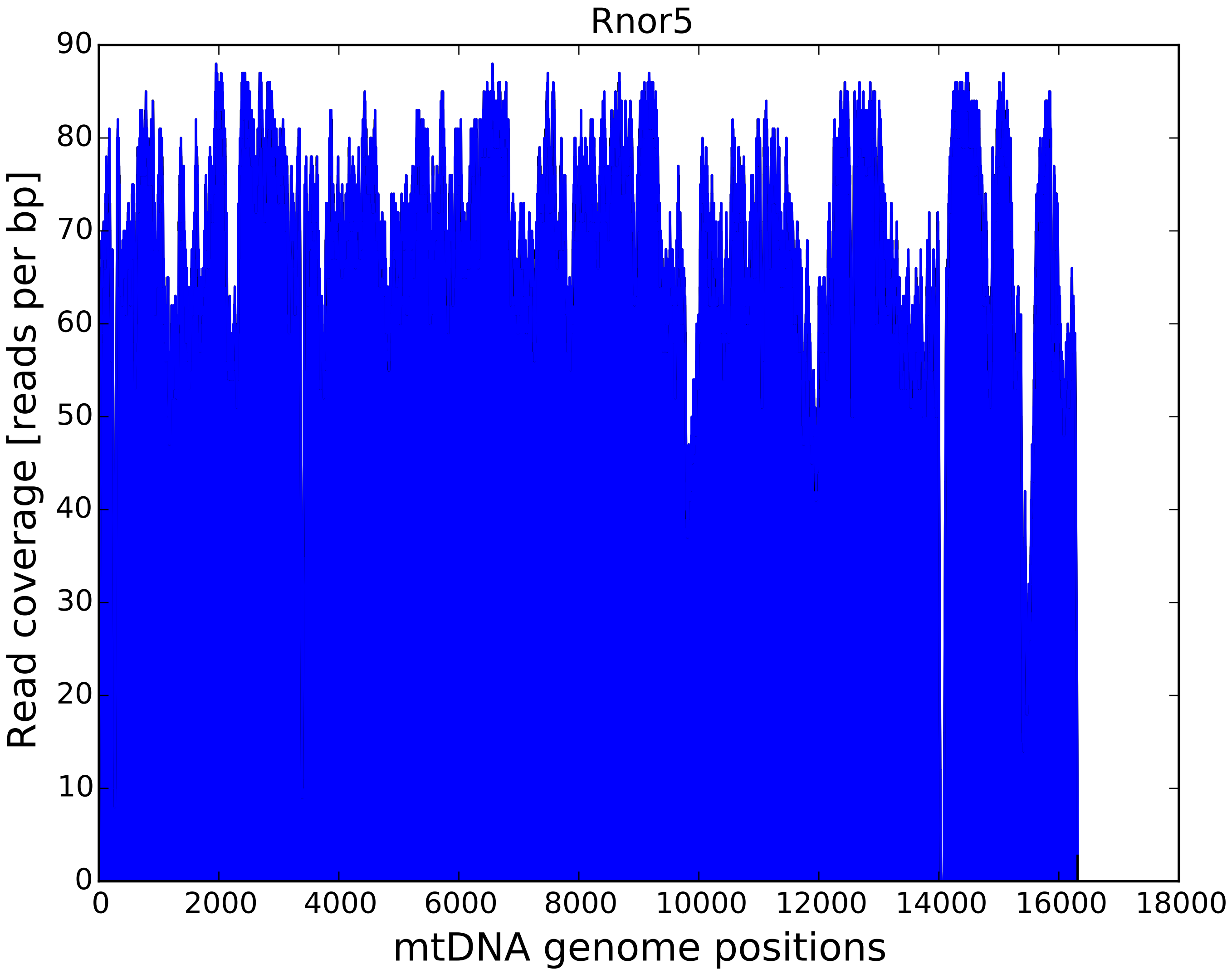
Rnor5
Read coverage [reads per bp]
mtDNA genome positions
0
10
20
30
40
50
60
70
80
90
0
2000
4000
6000
8000
10000
12000
14000
16000
18000

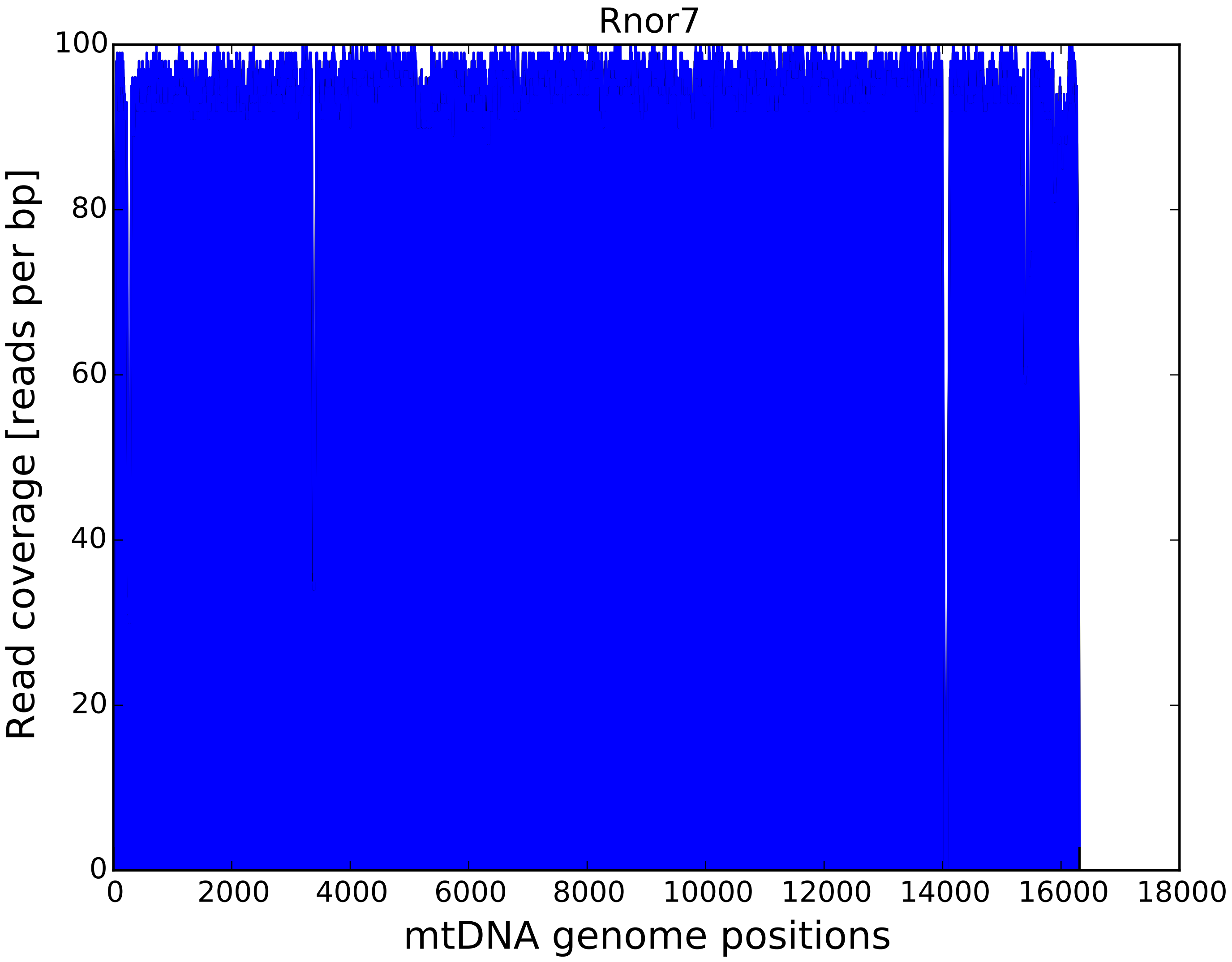
Rnor7
Read coverage [reads per bp]
mtDNA genome positions
0
20
40
60
80
100
0
2000
4000
6000
8000
10000
12000
14000
16000
18000

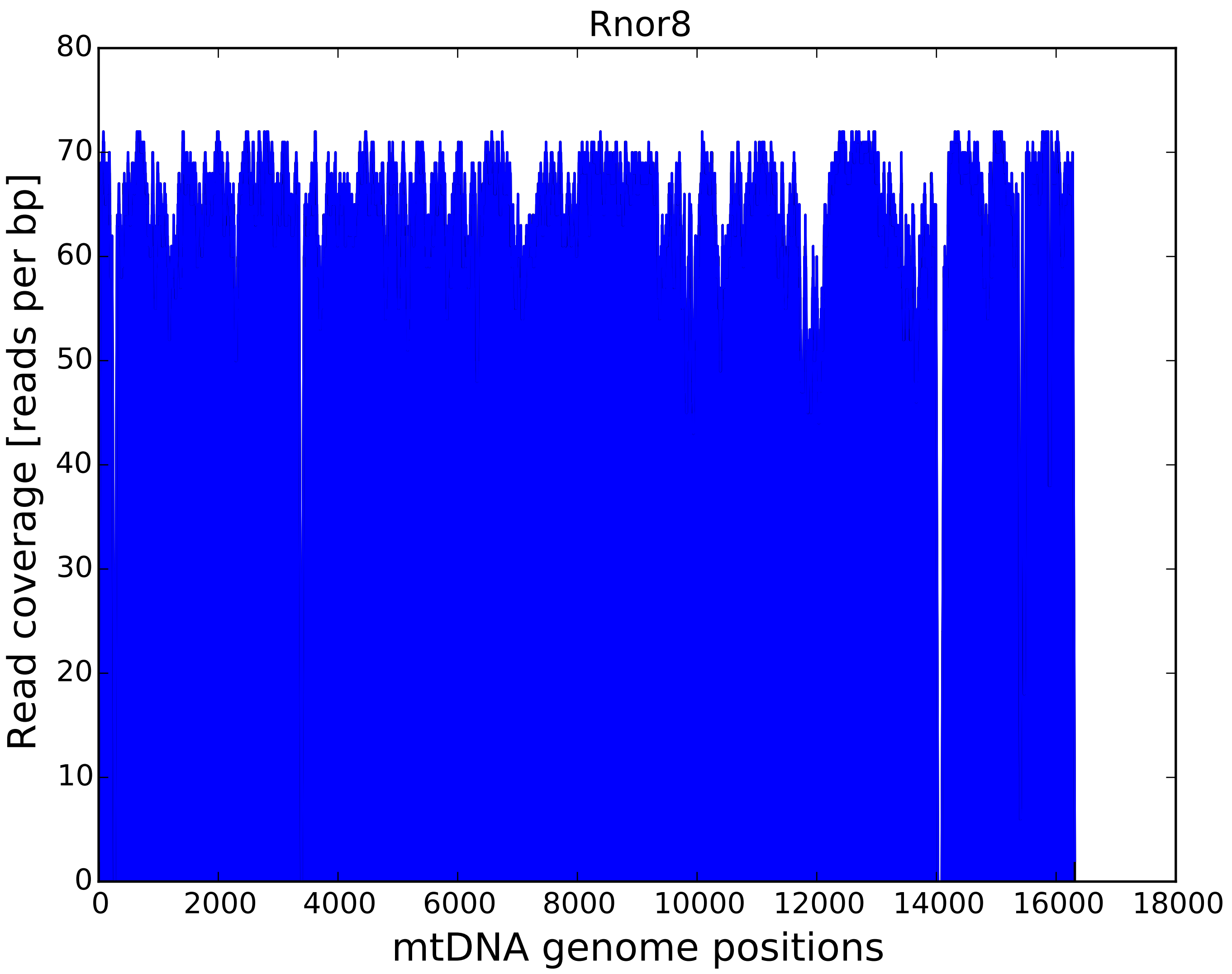
Rnor8
Read coverage [reads per bp]
mtDNA genome positions
0
10
20
30
40
50
60
70
80
0
2000
4000
6000
8000
10000
12000
14000
16000
18000

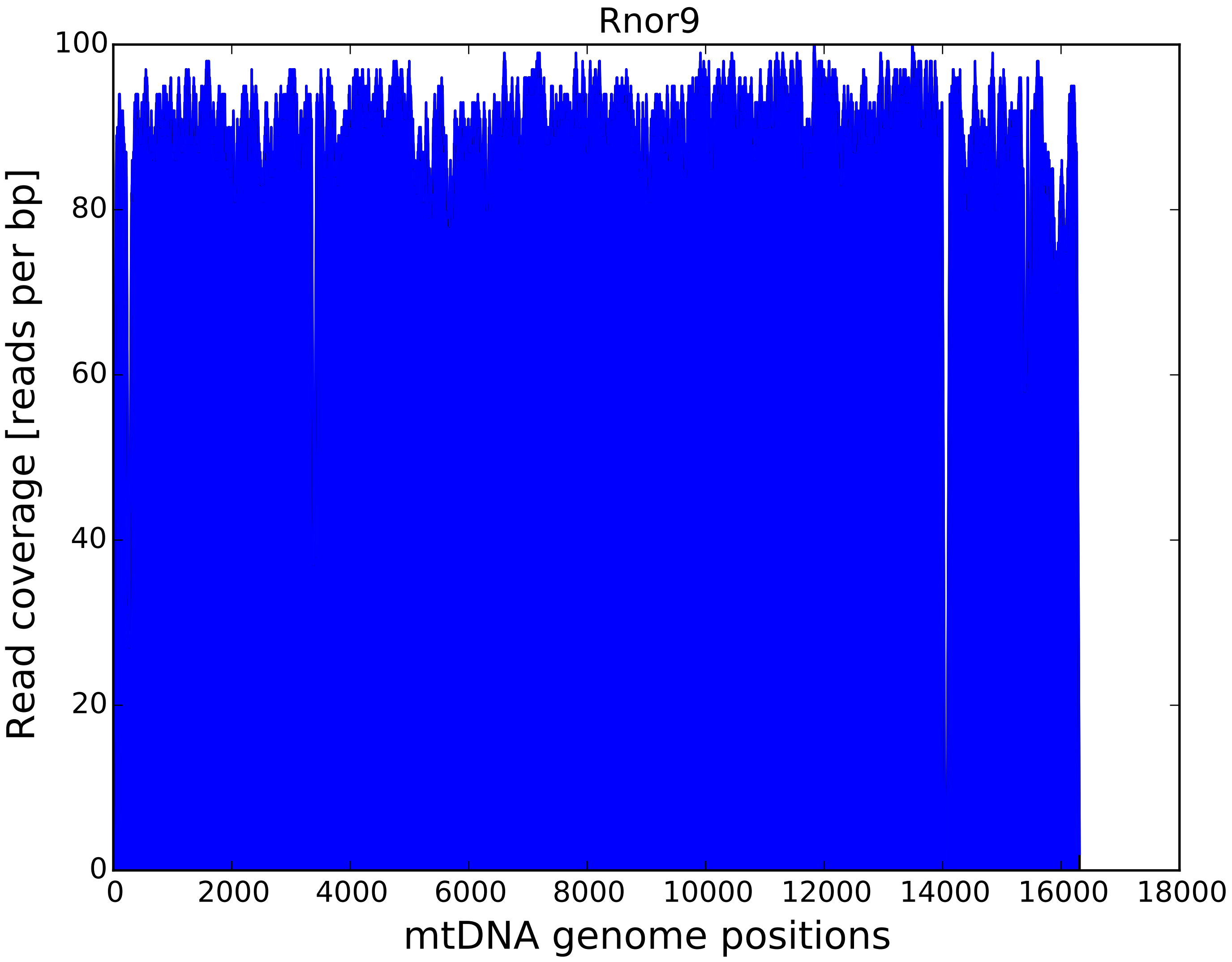

Rnor9
Read coverage [reads per bp]
mtDNA genome positions
0
20
40
60
80
100
0
2000
4000
6000
8000
10000
12000
14000
16000
18000

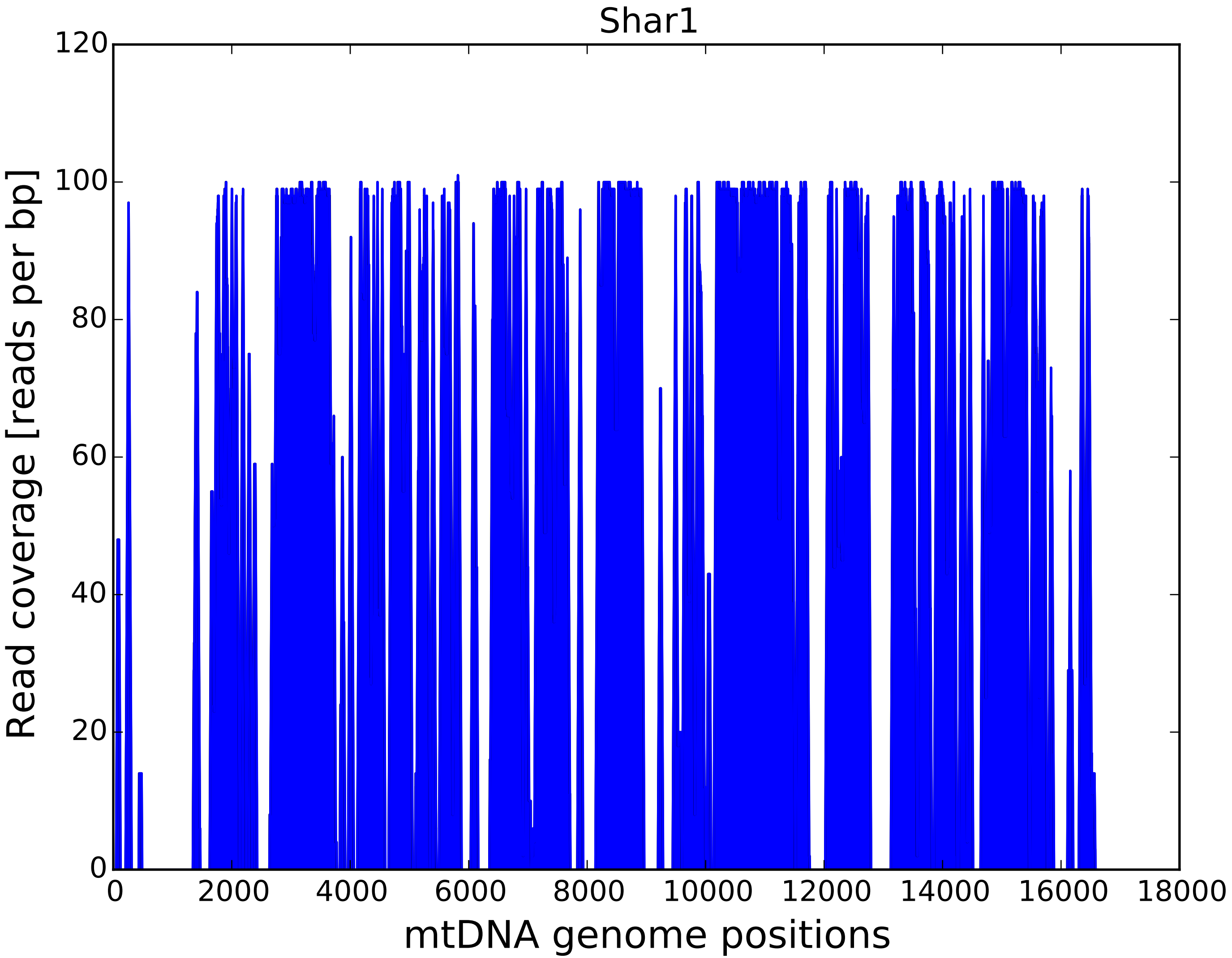
Shar1
Read coverage [reads per bp]
mtDNA genome positions
0
20
40
60
80
100
120
0
2000
4000
6000
8000
10000
12000
14000
16000
18000

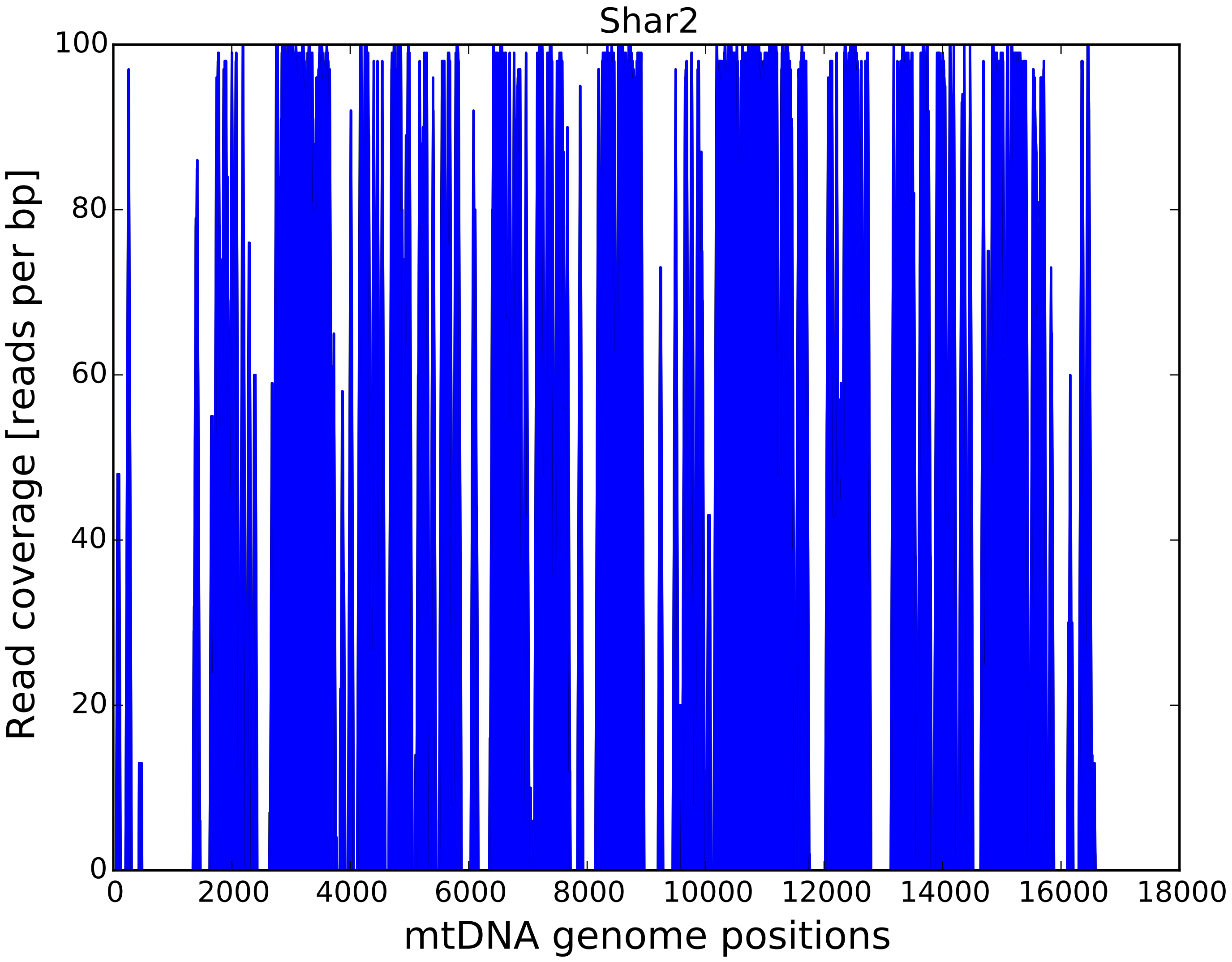

Shar2
Read coverage [reads per bp]
mtDNA genome positions
0
20
40
60
80
100
0
2000
4000
6000
8000
10000
12000
14000
16000
18000

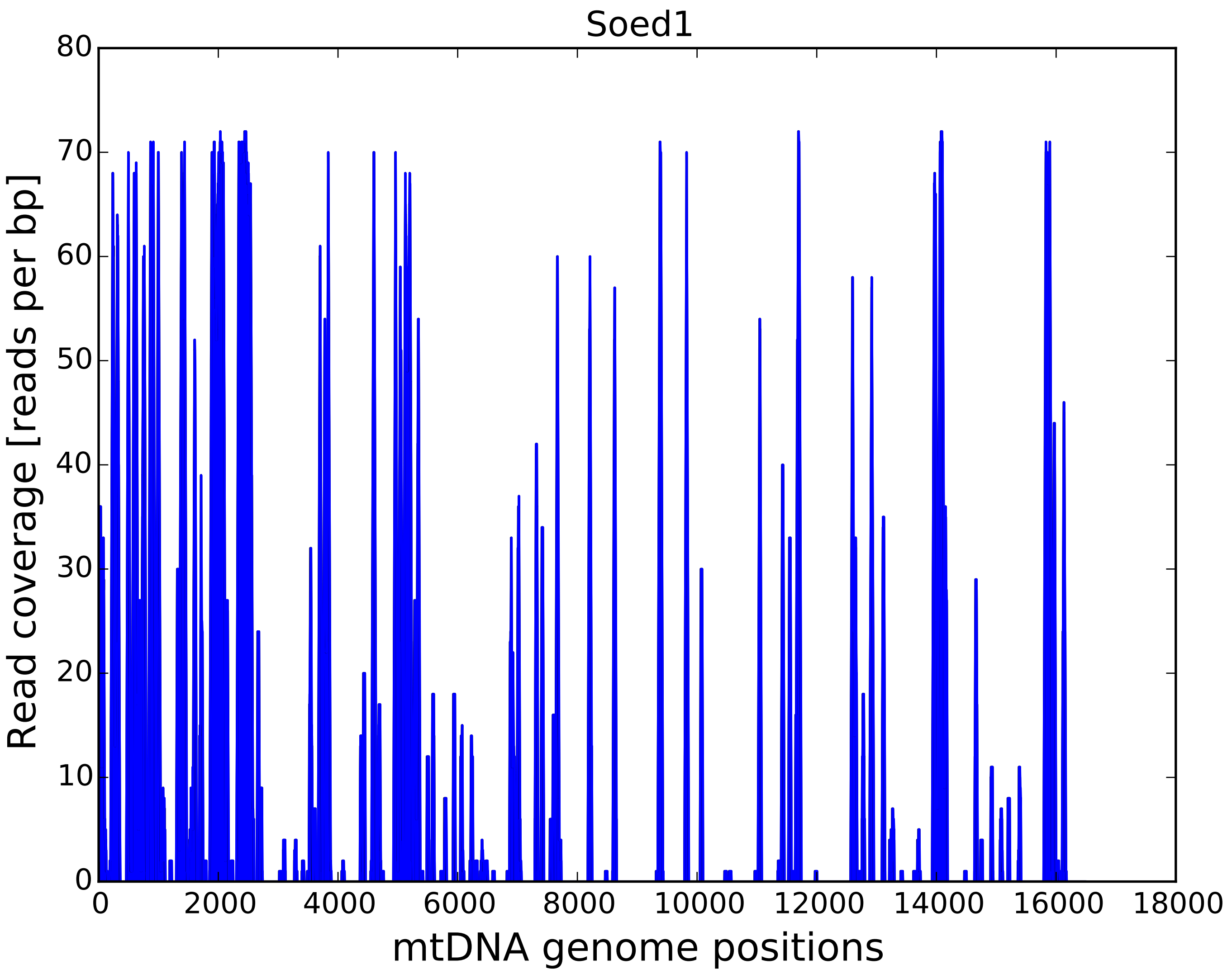
Soed1
Read coverage [reads per bp]
mtDNA genome positions
0
10
20
30
40
50
60
70
80
0
2000
4000
6000
8000
10000
12000
14000
16000
18000

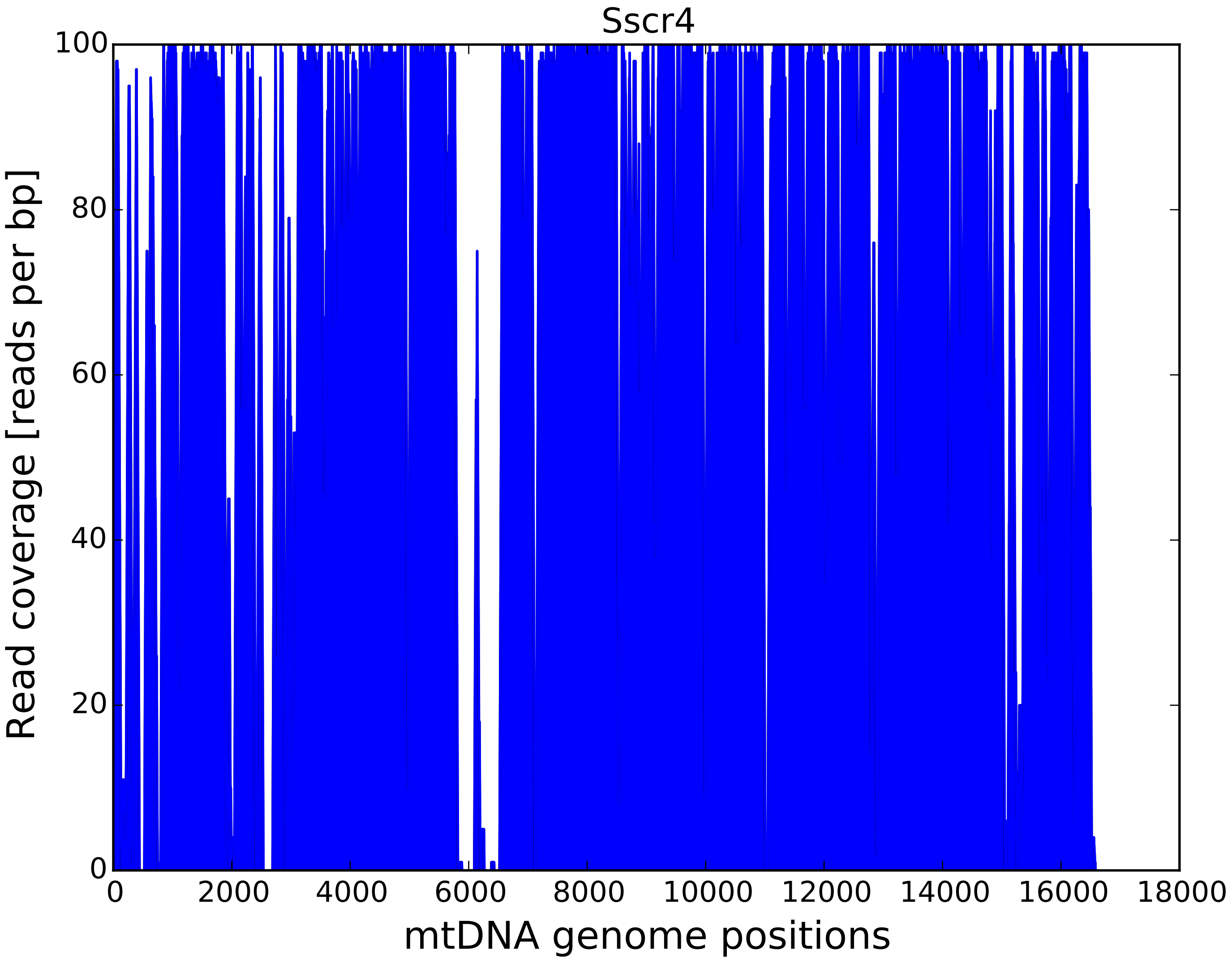
Sscr4
Read coverage [reads per bp]
mtDNA genome positions
0
20
40
60
80
100
0
2000
4000
6000
8000
10000
12000
14000
16000
18000

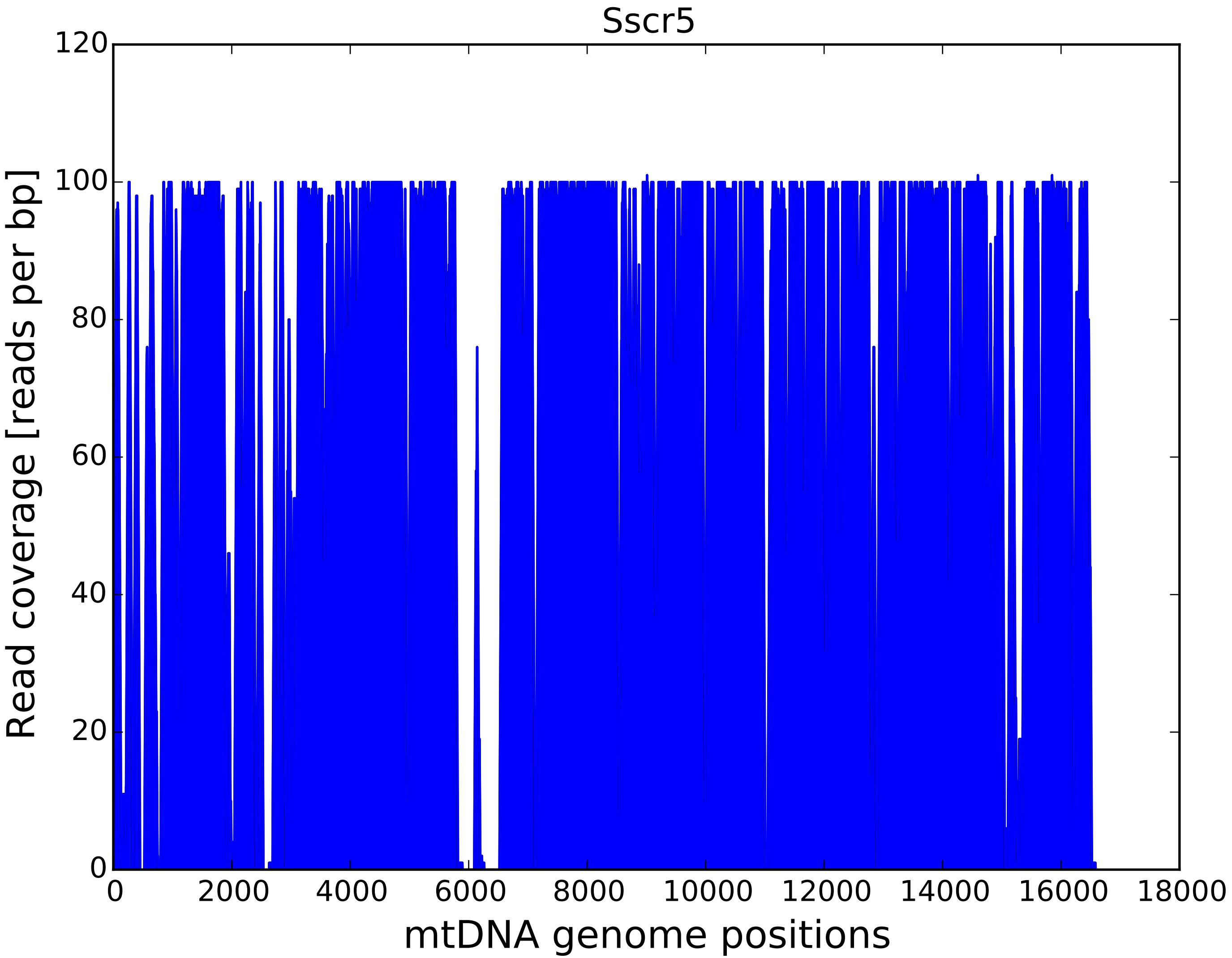
Sscr5
Read coverage [reads per bp]
mtDNA genome positions
0
20
40
60
80
100
120
0
2000
4000
6000
8000
10000
12000
14000
16000
18000

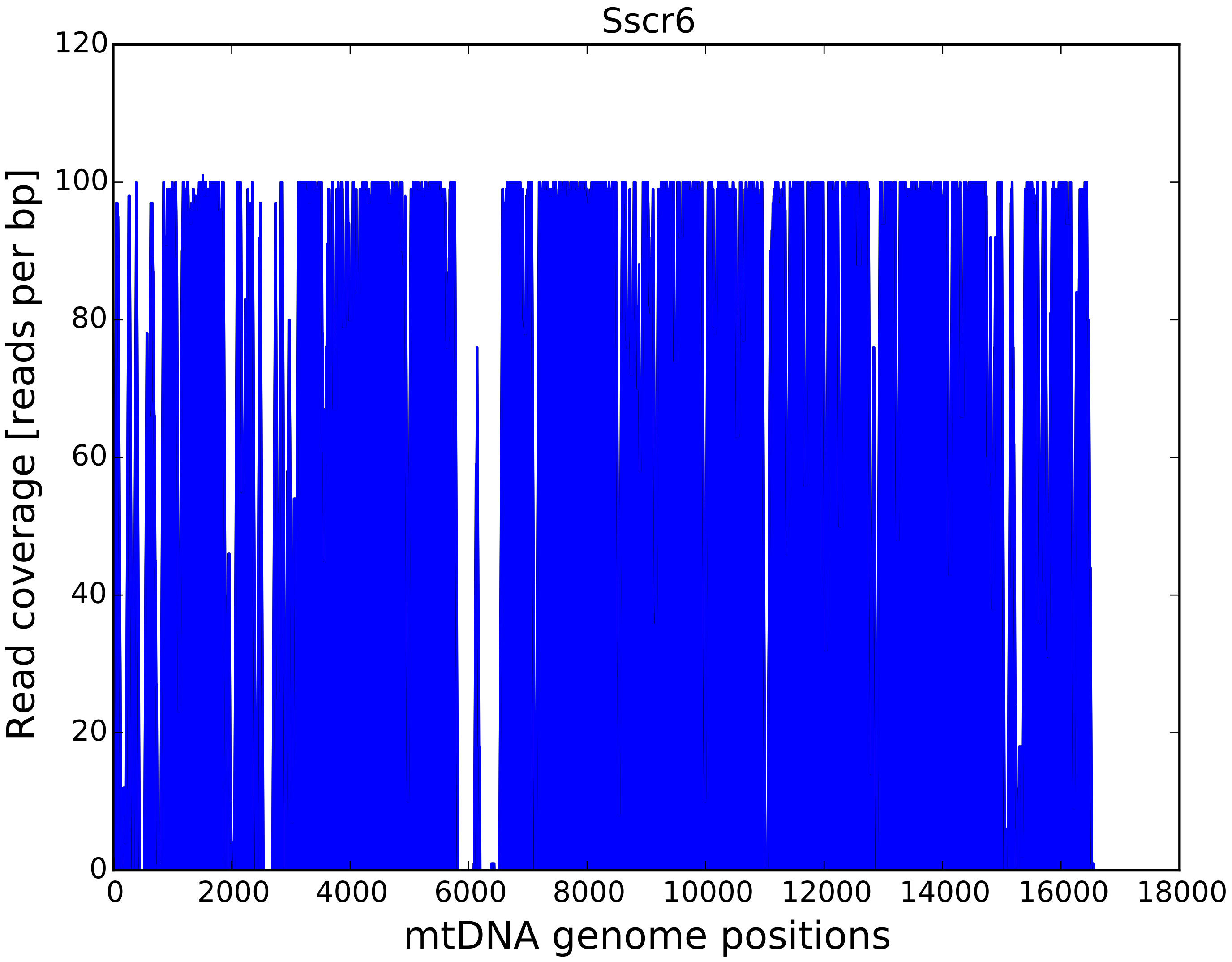
Sscr6
Read coverage [reads per bp]
mtDNA genome positions
0
20
40
60
80
100
120
0
2000
4000
6000
8000
10000
12000
14000
16000
18000

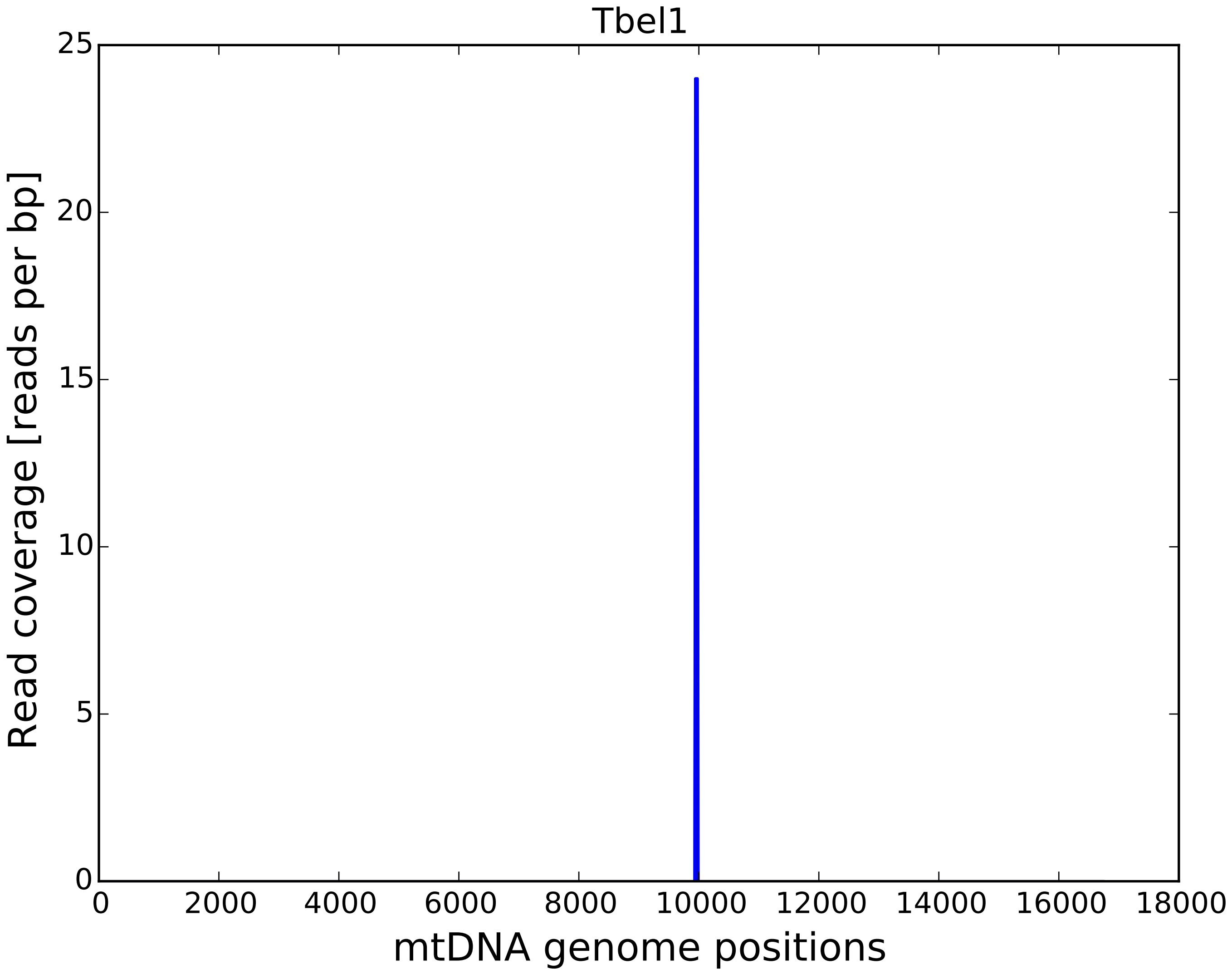
Tbel1
Read coverage [reads per bp]
0
5
10
15
20
25
0
2000
4000
6000
8000
10000
12000
14000
16000
18000
mtDNA genome positions

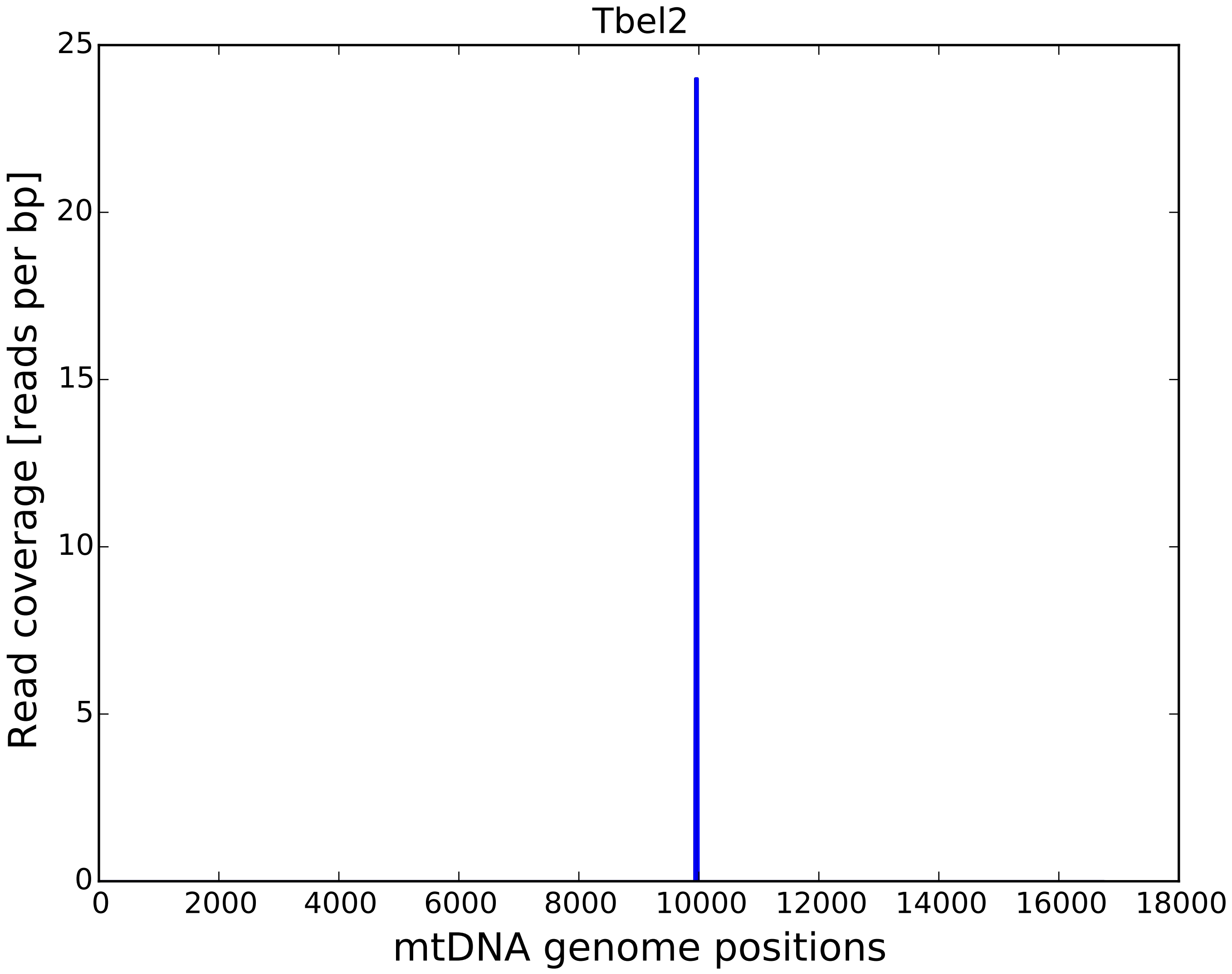

Tbel2
Read coverage [reads per bp]
mtDNA genome positions
0
5
10
15
20
25
0
2000
4000
6000
8000
10000
12000
14000
16000
18000

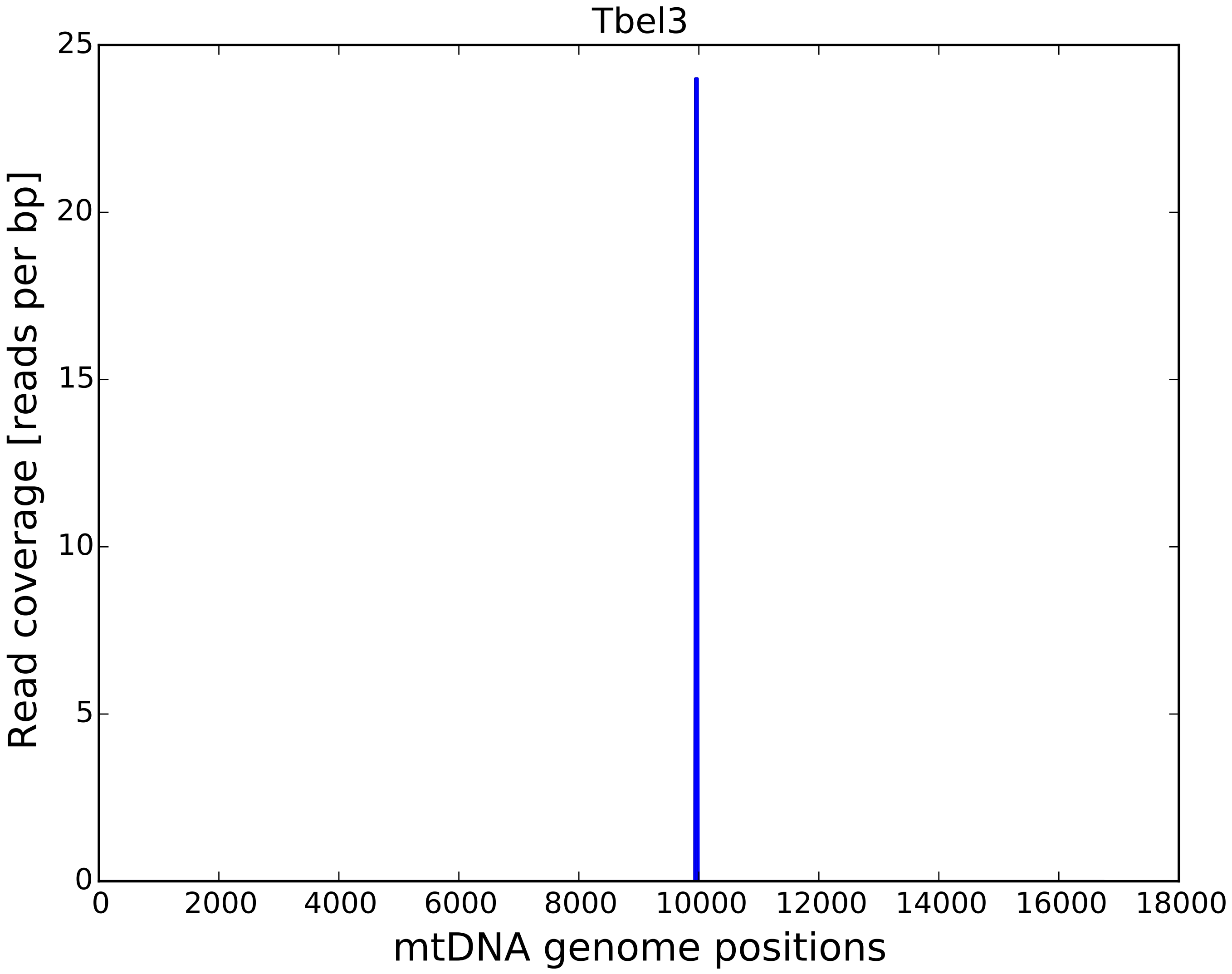

Tbel3
Read coverage [reads per bp]
mtDNA genome positions
0
5
10
15
20
25
0
2000
4000
6000
8000
10000
12000
14000
16000
18000

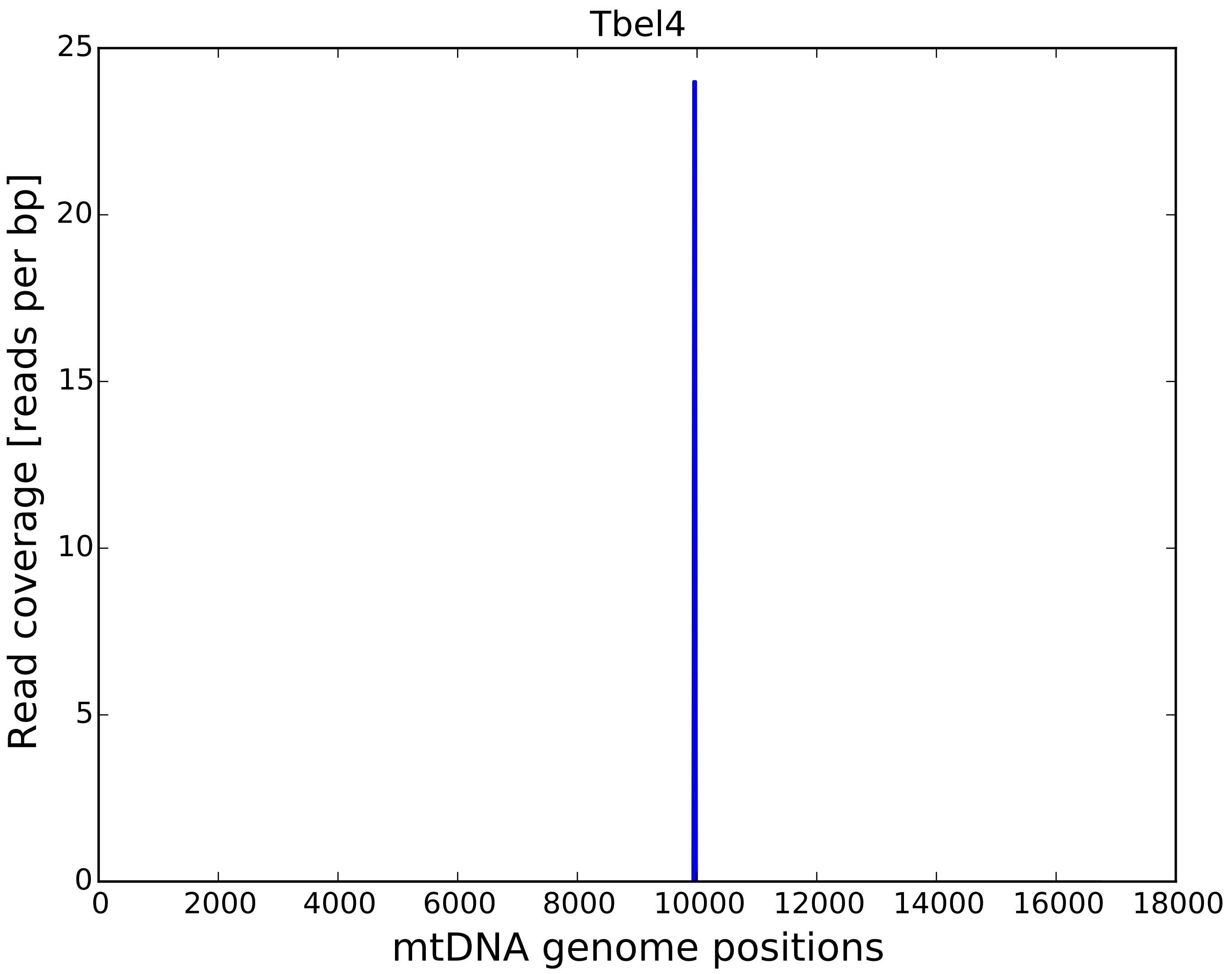
Tbel4
Read coverage [reads per bp]
mtDNA genome positions
0
5
10
15
20
25
0
2000
4000
6000
8000
10000
12000
14000
16000
18000

Figure S2: Read coverage of heteroplasmies

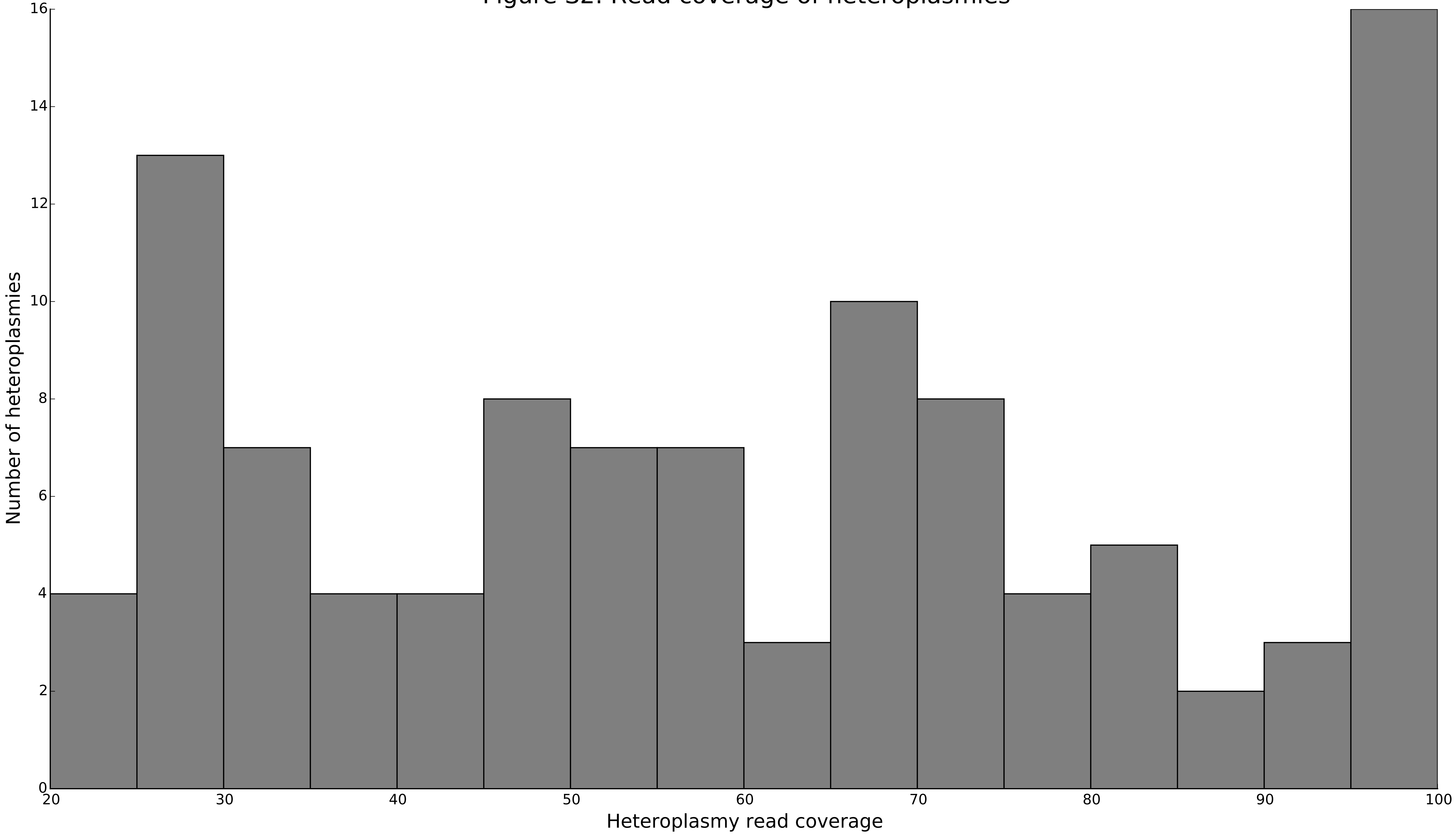

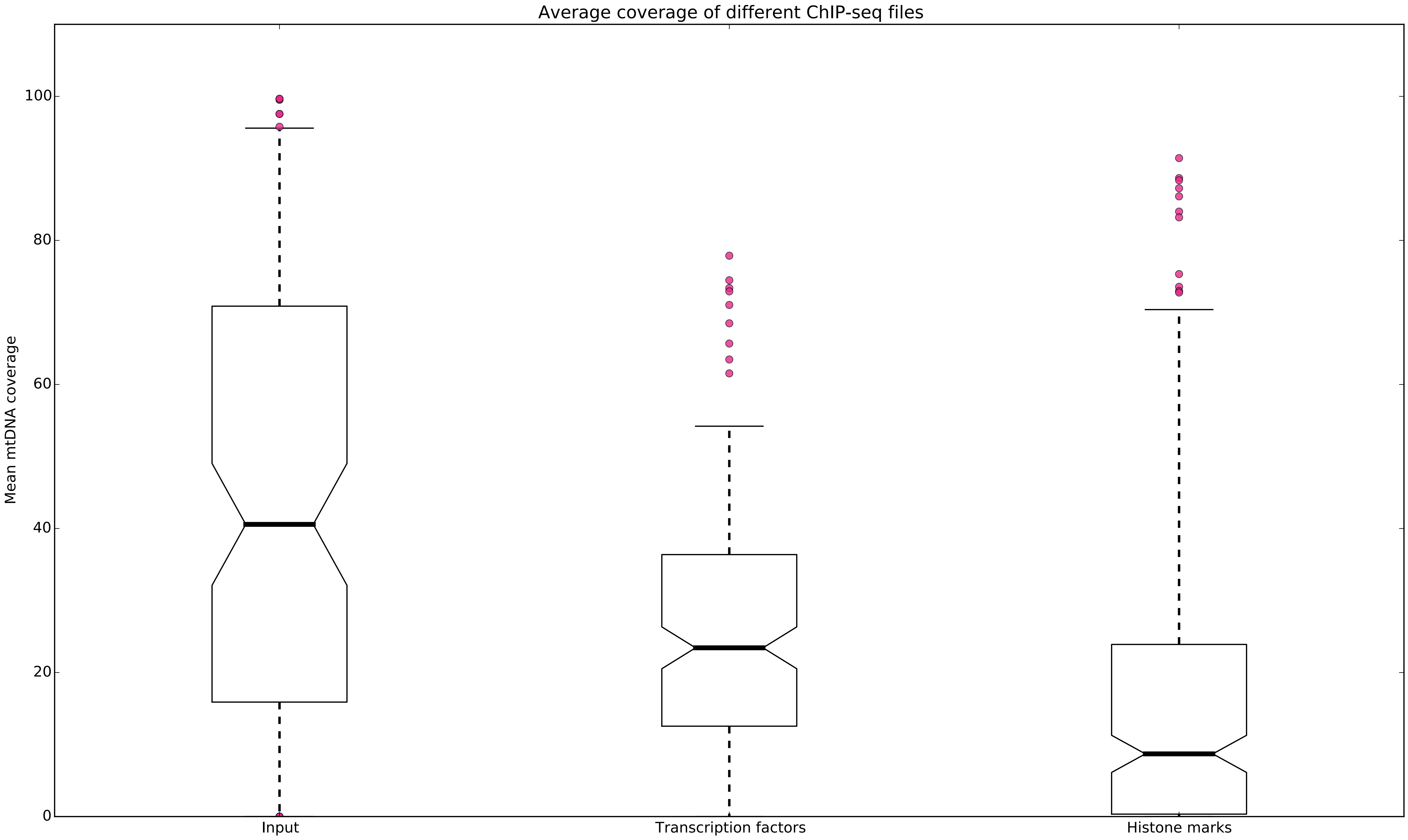
Average coverage of different ChIP-seq files
Mean mtDNA coverage
0
20
40
60
80
100
Input
Transcription factors
Histone marks
